# Bioinformatics Analyses Determined the Distinct CNS and Peripheral Surrogate Biomarker Candidates Between Two Mouse Models for Progressive Multiple Sclerosis

**DOI:** 10.3389/fimmu.2019.00516

**Published:** 2019-03-19

**Authors:** Seiichi Omura, Fumitaka Sato, Nicholas E. Martinez, Ah-Mee Park, Mitsugu Fujita, Nikki J. Kennett, Urška Cvek, Alireza Minagar, J. Steven Alexander, Ikuo Tsunoda

**Affiliations:** ^1^Department of Microbiology, Kindai University Faculty of Medicine, Osakasayama, Japan; ^2^Department of Microbiology and Immunology, Louisiana State University Health Sciences Center-Shreveport, Shreveport, LA, United States; ^3^Department of Pathology, University of Utah, Salt Lake City, UT, United States; ^4^Department of Computer Science, Louisiana State University Shreveport, Shreveport, LA, United States; ^5^Department of Neurology, Louisiana State University Health Sciences Center-Shreveport, Shreveport, LA, United States; ^6^Department of Molecular and Cellular Physiology, Louisiana State University Health Sciences Center-Shreveport, Shreveport, LA, United States

**Keywords:** multi-variate analysis, primary progressive EAE, principal component analysis (PCA), pattern matching, data mining

## Abstract

Previously, we have established two distinct progressive multiple sclerosis (MS) models by induction of experimental autoimmune encephalomyelitis (EAE) with myelin oligodendrocyte glycoprotein (MOG) in two mouse strains. A.SW mice develop ataxia with antibody deposition, but no T cell infiltration, in the central nervous system (CNS), while SJL/J mice develop paralysis with CNS T cell infiltration. In this study, we determined biomarkers contributing to the homogeneity and heterogeneity of two models. Using the CNS and spleen microarray transcriptome and cytokine data, we conducted computational analyses. We identified up-regulation of immune-related genes, including immunoglobulins, in the CNS of both models. Pro-inflammatory cytokines, interferon (IFN)-γ and interleukin (IL)-17, were associated with the disease progression in SJL/J mice, while the expression of both cytokines was detected only at the EAE onset in A.SW mice. Principal component analysis (PCA) of CNS transcriptome data demonstrated that down-regulation of prolactin may reflect disease progression. Pattern matching analysis of spleen transcriptome with CNS PCA identified 333 splenic surrogate markers, including *Stfa2l1*, which reflected the changes in the CNS. Among them, we found that two genes (*PER1*/*MIR6883* and *FKBP5*) and one gene (*SLC16A1/MCT1*) were also significantly up-regulated and down-regulated, respectively, in human MS peripheral blood, using data mining.

## Introduction

Multiple sclerosis (MS) is an inflammatory demyelinating disease of the central nervous system (CNS) (1). World-wide, MS affects about 2.5 million people (2). Although the precise etiology of MS remains unclear, MS has been proposed to be a disease caused by interactions between autoimmunity, microbial infections, and/or genetic factors (3). The clinical courses of MS are classified into four types: (1) clinically isolated syndrome (CIS), (2) relapsing-remitting (RR), (3) primary progressive (PP), (4) secondary progressive (SP) (4). CIS is a first clinical episode with CNS inflammation and demyelination (5). RR-MS is defined by “relapses” (disease attacks) with periods of “remission” (recovery) and is the most frequent occurring. SP-MS is defined by an initial RR disease course followed by continuous disease progression. Approximately 95% of RR-MS patients develop SP-MS (6). PP-MS progresses continuously from the onset without recovery. There is no biomarker that can be used to classify or predict clinical courses of the four subtypes of MS (7).

Neuroimaging studies suggest that MS lesions shifted from inflammatory demyelination to neurodegeneration during the disease progression (8, 9). In contrast, neuropathology studies suggest that the pathogenesis of MS remained the same throughout the course (10, 11). There was neither a definite mechanistic explanation of how the pathogenesis shifts from inflammatory demyelination to neurodegeneration in all MS cases, nor an explanation of whether the two conflicting views based on neuroimaging or neuropathology observations can be reconciled. The different views on disease pathogenesis in MS could be attributed to the fact that each view is based on one aspect of the disease: neuroimaging or histological changes. Alternatively, MS pathogenesis might differ among individual patients (12). The neuropathological view might be based on MS patients whose effector mechanism remains the same during the disease course, while the neuroimaging view could be based on the patient subgroup whose effector mechanism changes during the disease course. We hypothesized that inconsistencies of effectiveness of treatment, neuroimaging and neuropathology among progressive MS patients could be heterogeneities of the pathogenesis of MS.

Clinical courses of animal models for MS are also variable. Experimental autoimmune encephalomyelitis (EAE) can be induced by sensitization with CNS antigens, including myelin basic protein (MBP), myelin proteolipid protein (PLP), and myelin oligodendrocyte glycoprotein (MOG) (3, 13). The clinical course of EAE can be RR, PP, and SP, which are similar to the various forms of MS: RR-MS, PP-MS, and SP-MS, respectively. Several EAE models with different clinical courses have been established: RR-course in SJL/J mice with PLP_139−151_, PLP_178−191_, or MOG_92−106_, PP-, and SP-course in A.SW mice with MOG_92−106_ and SJL/J mice with MOG_92−106_ and additional treatment (ultraviolet irradiation, injection of *Bordetella Pertussis*, apoptotic cell, or curdlan) (14–17). Monophasic EAE can also be induced in PL/J mice with MBP_1−11_ and C57BL/6 mice with MOG_35−55_ (18, 19). In this study, we used two PP-EAE models, A.SW mice sensitized with MOG_92−106_ and SJL/J mice sensitized with MOG_92−106_ and curdlan. Previously, we reported that A.SW mice sensitized with MOG_92−106_ developed PP-EAE with large areas of demyelination, immunoglobulin deposition, neutrophil infiltration, and spleen atrophy (14, 16).

Systemic and multivariate analyses of data from animal models for MS are powerful methods to characterize each model. In MS, microarray analyses have been performed mainly using peripheral blood lymphocytes. Several reports showed that various genes related to the immune response, apoptosis, and cell cycle progression were up- or down-regulated in disease (20, 21), while microarray analyses using human CNS tissues have been limited by its nature (22). In most microarray analyses in EAE, genes related to the immune response, such as cytokines, chemokines, and complement components, are known to be up-regulated in the CNS (23–26).

We aimed to determine CNS biomarkers and peripheral surrogate markers that could characterize the two PP-EAE models induced in A.SW and SJL/J mice. We have conducted microarray and bioinformatics analyses, using the brains and spleens which reflect the changes in the CNS and peripheral lymphoid organs, respectively. There were differences in numbers of genes that were up- and down-regulated in the brains and spleens between A.SW and SJL/J mice with PP-EAE, while immune response-related genes were highly up-regulated in the brains and erythrocyte-related genes highly down-regulated in the spleens from both mouse strains. Pathway analysis showed that Fc receptor and complement-related genes were up-regulated in both mouse strains' brains, but pro-inflammatory cytokine-related genes were up-regulated only in SJL/J mouse brains. Genes irrelevant to immune responses were down-regulated in the spleens of PP-EAE mice, and the expression of T helper (Th)1/Th2-related genes differed between A.SW and SJL/J mouse brains. Principal component analysis (PCA) of transcriptome data of brains and spleens separated between control and EAE groups. Pattern matching analysis between brain PCA data and spleen transcriptome data identified the spleen surrogate marker candidates that reflect the gene expression patterns in the brain. Translational application of our bioinformatics approach would be useful to identify the brain biomarkers and peripheral surrogate markers for MS.

## Materials and Methods

### Animal Experiments

To induce PP-EAE, 5-week-old female nine SJL/J mice and 13 A.SW mice (The Jackson Laboratory, Bar Harbor, ME) were sensitized in the base of the tail with 100 nmol of MOG_92−106_ peptide (DEGGYTCFFRDHSYQ, Core Facility, University of Utah Huntsman Cancer Institute, Salt Lake City, UT) in complete Freund's adjuvant (CFA) (14–16). On day −1 prior to MOG injection, 5 mg curdlan (a Th17 inducer produced by *Alcaligenes faecalis var. myxogenes*, Wako Pure Chemical Industries, Osaka, Japan) in PBS was injected for SJL/J mice intraperitoneally (27). To induce RR-EAE, six SJL/J mice were sensitized PLP_139−151_ (HSLGKWLGHPDKF) in CFA (28). Mice were given standard laboratory rodent chow and water *ad libitum*. All experimental procedures were reviewed and approved by the Institutional Animal Care and Use Committee of Louisiana State University Health Sciences Center (LSUHSC)-Shreveport, and performed according to the criteria outlined by the National Institutes of Health (NIH) (29).

**Figure 1:**
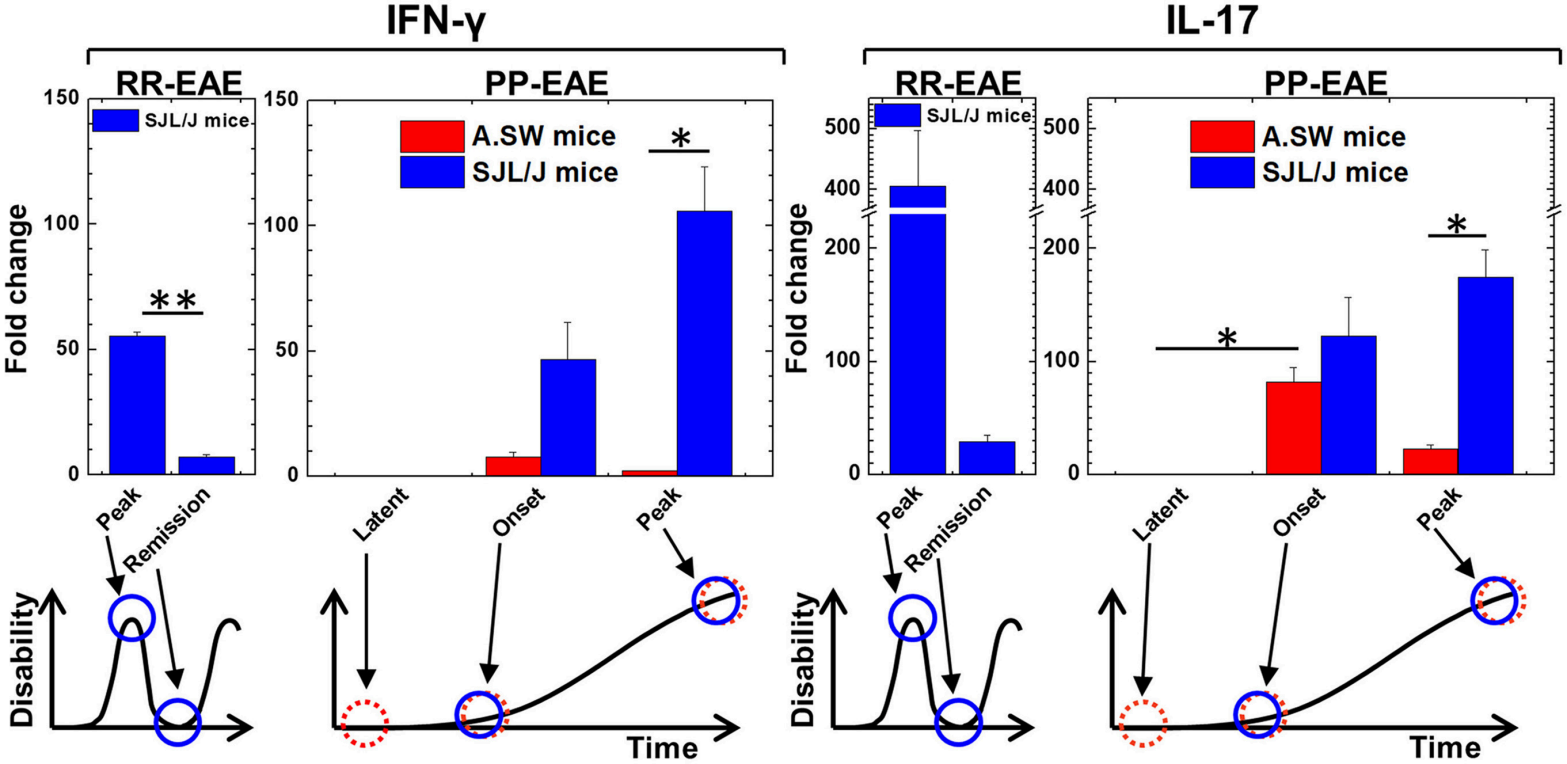
Kinetics of interferon (IFN)-γ and interleukin (IL)-17 expression of relapsing-remitting (RR)-experimental autoimmune encephalomyelitis (EAE) and primary progressive (PP)-EAE in SJL/J mice (blue column) and A.SW mice (red column). Expression of IFN-γ and IL-17 in brains were determined by real-time PCR. Expression levels were shown as fold changes compared with three control mice of each strain. In SJL/J mice with RR-EAE, both IFN-γ and IL-17 levels were high at the disease peak and low during remission (*n* = 3, at each time point). On the other hand, in PP-EAE, expression levels of both IFN-γ and IL-17 were associated with disease activity in SJL/J mice with EAE (*n* = 3, at each time point), while they increased at the onset (no increase at latent period) but decreased at the disease peak in A.SW mice with EAE (*n* = 3–6, at each time point). Data are presented as means ± standard error of the mean (SEM). *P < 0.05, **P < 0.001, ANOVA.

Clinical signs of EAE and body weights were monitored daily (14, 30). Mice were euthanized at disease peak and remission of RR-EAE and at latent period, onset and peak of PP-EAE (Figure 1). At each time point, brains and spleens were harvested from three to six mice per group and frozen immediately in liquid nitrogen.

### RNA Preparation

Brains and spleens from three to six mice per group were homogenized individually in TRI-Reagent® (Molecular Research Center, Cincinnati, OH), using the Kinematica Polytron™ homogenizer (Kinematica, Bohemia, NY). Total RNA was extracted with an RNeasy Mini Kit (Qiagen, Germantown, MD) according to the manufacturer's instructions from brain and spleen homogenate. DNase treatment was performed during RNA isolation with an RNase-Free DNase Set (Qiagen). All samples were purified to an absorbance ratio (A260/A280) between 1.9 and 2.1 (31).

### Real-Time PCR

We reverse-transcribed 1 μg of total RNA into cDNA, using ImProm-II™ Reverse Transcription System (Promega Corporation, Madison, WI) (*n* = 3–7). We mixed 50 ng of cDNA with RT^2^ Fast SYBER® Green qPCR Master Mixes (Qiagen) and primer set. The mixture was amplified and monitored using iCycler iQ System (Bio-Rad Laboratories, Hercules, CA). The following primer sets were purchased from Real Time Primers (Elkins Park, PA): interferon (IFN)-γ, interleukin (IL)-17A, chemokine (C-X-C motif) ligand 13 (CXCL13), lipocalin 2 (LCN2), CD3 antigen γ subunit (CD3G), Kell blood group (KEL), and stefin A2 like 1 (STFA2L1). The results were normalized using housekeeping genes, glyceraldehyde-3-phosphate dehydrogenase (*Gapd*) or phosphoglycerate kinase 1(*Pgk1*) (32, 33).

### Microarray Analysis

We used total RNA samples of brains and spleens from three mice with PP-EAE at the disease peak and three age-matched control mice for each mouse strain. We conducted microarray analyses, using Affymetrix GeneChip® Mouse Gene 1.0 ST Array (Affymetrix, Santa Clara, CA), according to the manufacturer's instruction. The data were visualized and quantified by Affymetrix GeneChip Command Console (AGCC), and normalized by Robust Multi-array Average (RMA) using Expression Console. Data were analyzed using the Ingenuity Pathway Analysis® (Qiagen), NetAffx database (Affymetrix; http://www.affymetrix.com/index.affx), and Mouse Genome Informatics (The Jackson Laboratory, Bar Harbor, ME; http://www.informatics.jax.org/). The datasets generated and/or analyzed during the current study are available in the Gene Expression Omnibus (GEO; http://www.ncbi.nlm.nih.gov/geo/) repository in National Center for Biotechnology Information (NCBI) (Accession number: GSE99300).

### Bioinformatics and Statistics Analyses

#### Volcano Plot

We drew a volcano plot, using the OriginPro 8.1 (OriginLab Corporation, Northampton, MA), to visualize significance and fold changes of transcriptome data (34–36). In the volcano plot, log ratios (logarithms of fold changes to base 2) of gene expression in the brains and spleens from EAE mice compared with age-matched control mice were used as an x-axis and the logarithms of *P* values to base 10 were used as a y-axis.

#### Heat Map

We drew heat maps to determine the expression patterns of top 20 up- or down-regulated genes of brain and spleen samples from EAE mice, and compared the expression levels between EAE vs. control groups, using R version 3.2.2 and the programs “gplots” and “genefilter” (37). A list of abbreviations of genes is shown in Supplemental Table 1.

#### *K*-means Clustering

To find the differences of gene expression patterns between organs or mouse strains, we conducted *k*-means clustering using an R package “cclust” (37). We used Davies-Bouldin index (38) to determine the optimum number of clusters and obtained the lowest score (0.78), when microarray data were separated into 35 clusters (Supplemental Figure 2). Graphs were drawn using 240 genes (top 80, middle 80, and bottom 80 genes) in each cluster. A radar chart was drawn using the expression patterns of cluster center genes.

#### Ingenuity Pathway Analysis (IPA)

To classify the genes functionally, we used IPA (Qiagen) where we entered the genes whose genes were over- or under-transcribed more than 2-fold compared with control samples (*P* values <0.05). IPA shows possible networks involved in microarray profiles by the IPA Network Generation Algorithm (39). The algorithm clustered and classified the entered genes, which generated the networks, each of which was composed of three canonical pathways. The networks were ranked by the network score. The network score was calculated based on the right-tailed Fisher's Exact Test that uses several parameters, including the numbers of network eligible molecules in the network, the given dataset, and the IPA database. We focused the networks whose network score was higher than 35, since the only networks with high network scores have interpretable connections.

#### Principal Component Analysis (PCA)

Using PCA, we reduced the dimensionality of a microarray data set consisting of 28,853 mRNA expression signals into two components, principal component (PC) 1 and PC2 (37, 40, 41). PCA was conducted as an “unsupervised” analysis to clarify the variance among microarray data from brain and spleen samples using an R program “prcomp,” as we described previously (37, 42). The proportion of variance was also calculated to determine the percentage of variance explained by each PC, while factor loading for PC1 or PC2 was used to rank a set of genes contributing to PC1 or PC2 values.

#### Pattern Matching Analysis

To find the splenic genes whose expression patterns correlated with PC1 values in PCA of the brains, we conducted a pattern matching analysis based on correlation (43), using the R. We focused the genes whose expression levels, compared with control samples, were up- or down-regulated more than 2-fold, and whose correlation coefficients (*r*) were more than 0.8 or <−0.8.

#### Data Mining on Human MS Transcriptome Database

We obtained the gene expression profile datasets relevant to MS patients from GEO profile database (https://www.ncbi.nlm.nih.gov/geoprofiles/), using search keywords with the gene symbols identified in the current study as follows:

“multiple sclerosis”[All Fields] AND “Homo sapiens”[Organism] AND “peripheral blood”[All Fields] AND “disease state”[Flag Information] AND (gene symbols connected by OR). The data were processed according to the instructions of GEO profile database (44), and the differentially expressed genes (*P* < 0.05) between MS patients and controls were extracted.

#### Statistics

The data were shown as mean ± standard error of the mean (SEM). Statistical comparisons were conducted using the Student *t* test or analysis of variance (ANOVA), using the OriginPro 8.1. *P* < 0.05 was considered as significant difference.

## Results

### Levels of IFN-γ and IL-17 Were Associated With Disease Activity of PP-EAE in SJL/J Mice, but Not in A.SW Mice

The precise effector mechanism of PP-MS is currently unknown. Since the pro-inflammatory cytokines, IFN-γ, and IL-17 have been shown to be key effector molecules in many, but not all EAE models (15, 16, 45–48), we first examined the kinetics of IFN-γ and IL-17 in animal models for PP-MS, using two mouse strains. We induced PP-EAE with MOG_92−106_ in A.SW mice as we described previously (14–16). We also induced PP-EAE in SJL/J mice with MOG_92−106_ sensitization, 1 day after injection of curdlan. In SJL/J mice, disease continuously progressed until mice became moribund without remission within 20 days after initial clinical signs (Supplemental Figure 1). A.SW mice developed ataxic EAE and weight loss 1 month post induction (p.i.) of EAE (14, 16), while SJL/J mice developed classical EAE with tail and limb paralysis and weight loss in both PP- and RR-EAE.

Using real-time PCR, we conducted a time course analysis of IFN-γ and IL-17 mRNA levels in the brains of A.SW and SJL/J mice with PP-EAE (Figure 1). For comparison, we also used brain samples from SJL/J mice with RR-EAE. In both RR-EAE and PP-EAE in SJL/J mice, clinical signs were associated with increased levels of both IFN-γ and IL-17 in the brain. However, in PP-EAE in A.SW mice, the levels of IFN-γ and IL-17 increased at the disease onset, but declined during the disease progression. These results suggested that the effector mechanisms in disease progression of SJL/J mice and A.SW mice differed at the disease peak. Thus, we decided to analyze potentially distinct pathomechanisms of disease progression between A.SW and SJL/J mice with PP-EAE.

### Volcano Plots of Brain and Spleen Transcriptome Data Showed Overall Greater Changes in SJL/J Mice, While A.SW Mice Had More Down-Regulated Spleen Genes

**Figure 2:**
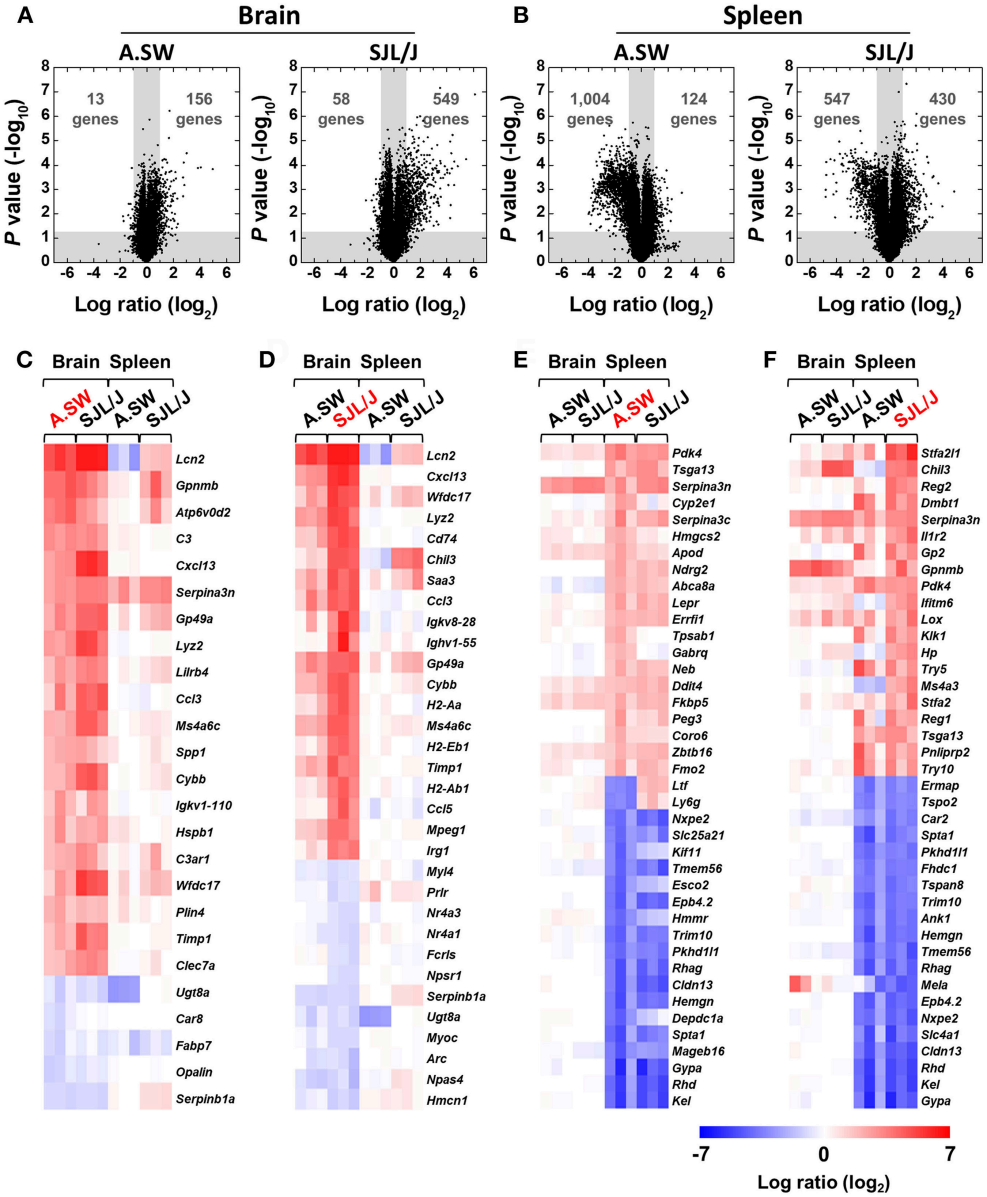
**(A,B)** Volcano plots of up- or down-regulated genes in the brains **(A)** and spleens **(B)** of PP-EAE mice. Gene expression profiles in the brains and spleens of three mice with PP-EAE at the disease peak and three age-matched control mice for each mouse strain were determined by microarray. Fold changes were calculated by division of signal values of EAE samples by those of controls. *P* values were calculated using the Student *t* test. Dots in the white areas were genes whose expressions were up- or down-regulated more than 2-fold (log_2_ ratio = 1, compared with controls), significantly (*P* < 0.05). In the brain, SJL/J mice had substantially higher numbers of both up- and down-regulated genes, compared with A.SW mice. On the other hand, in the spleen, SJL/J mice had a higher number of up-regulated genes, but a lower number of down-regulated genes, compared with A.SW mice. **(C–F)** Heat maps of identified genes among most highly up- or down-regulated 20 genes in the brains and spleens at disease peak of PP-EAE. Red, blue, and white indicate up-regulation, down-regulation, and no change, respectively. **(C)** In the brains of A.SW mice with PP-EAE, significantly up-regulated genes included: lipocalin 2 (*Lcn2*), complement-related genes, and chemokines. **(D)** In the brains of SJL/J mice with PP-EAE, *Lcn2*, chemokine-, MHC molecule-, and immunoglobulin-related genes were highly up-regulated. **(E,F)** In the spleens of both A.SW **(E)** and SJL/J **(F)** mice with PP-EAE, erythrocyte-related genes were down-regulated significantly. Overall, heat maps between A.SW and SJL/J mice were similar in the brains or spleens. A list of abbreviations of genes is shown in Supplemental Table 1.

To compare the potentially distinct effector mechanisms during the disease progression between A.SW and SJL/J mice, we conducted conventional “supervised” two-way comparison analyses of the brain and spleen transcriptome data at the disease peak from the two mouse strains with PP-EAE. First, using volcano plots, we visualized the numbers of genes whose expression levels were significantly (*P* < 0.05) up- or down-regulated more than 2-fold compared with control samples (Figures 2A,B). In the brain, higher numbers of genes were up- or down-regulated in SJL/J mice than in A.SW mice, suggesting that molecular changes in SJL/J mice could be more complex than in A.SW mice (Figure 2A). On the other hand, in the spleen, the numbers of down-regulated genes were higher in A.SW mice, while those of up-regulated genes were higher in SJL/J mice (Figure 2B). An increased number of down-regulated genes in A.SW mouse spleen seemed to be associated with spleen weight changes at disease peak in PP-EAE, where the spleen of A.SW mice, but not SJL/J mice, showed significant atrophy (16).

### Heat Maps Revealed the Up-Regulation of Immune Response-Related Genes in the Brains and Down-Regulation of Erythrocyte-Related Genes in the Spleens From Both Mouse Strains

Next, to visualize the differences in most highly up- or down-regulated genes in the brains and spleens, we drew heat maps, using microarray data at the disease peak (Figures 2C–F). Overall, heat maps of each organ (brain or spleen) were similar among samples from A.SW and SJL/J mice. Most of the highly up- or down-regulated genes in each organ in A.SW mice were also up- or down-regulated in SJL/J mice. On the other hand, heat maps between brains and spleens were different regardless of the mouse strains. Only serine (or cysteine) peptidase inhibitor, clade A, member 3N (*Serpina3n*) was highly up-regulated in both brains and spleens in both mouse strains. In the heat maps based on brain gene expression levels from A.SW (Figure 2C) and SJL/J mice (Figure 2D), commonly up-regulated genes included: lipocalin 2 (*Lcn2*); chemokines, such as chemokine (C-X-C motif) ligand 13 (*Cxcl13*); and chemokine (C-C motif) ligand 3 (*Ccl3*); complement-related genes, *C3* and complement component 3a receptor (*C3ar1*); immunoglobulin (*Igkv1-110*); MHC class II-related genes, *H2-Aa* and *Cd74* (CLIP). Serine (or cysteine) peptidase inhibitor, clade B, member 1a (*Serpinb1a*), and UDP galactosyltransferase 8A (*Ugt8a*) were down-regulated in common.

In the spleen heat maps based on gene expression levels from A.SW (Figure 2E) and SJL/J mice (Figure 2F), several genes, including pyruvate dehydrogenase kinase, isoenzyme 4 (*Pdk4*), testis specific gene A13 (*Tsga13*), and *Serpina3n*, were up-regulated in common, while erythrocyte-related genes, such as glycophorin A (*Gypa*), Kell blood group (*Kel*), Rh blood group, D antigen (*Rhd*), and claudin 13 (*Cldn13*) (49), were down-regulated significantly. On the other hand, lactotransferrin (*Ltf*) and lymphocyte antigen 6 complex, locus G (*Ly6g*/*Gr1*, a granulocyte marker) (50) showed different expression patterns between the spleens of two mouse strains with PP-EAE, down-regulation in A.SW mice and up-regulation in SJL/J mice. In addition, tryptase α/β 1 (*Tpsab1*) and γ-aminobutyric acid (GABA) A receptor, subunit θ (*Gabrq*) were up-regulated only in the spleen of A.SW mice, while immune response-related genes, such as chitinase-like 3 (*Chil3*), interferon induced transmembrane protein 6 (*Ifitm6*), and haptoglobin (*Hp*), were up-regulated only in the spleen of SJL/J mice.

### *K*-means Clustering Revealed the Different Expression Patterns of Genes Between the Brains and Spleens of A.SW and SJL/J Mice With PP-EAE

**Figure 3:**
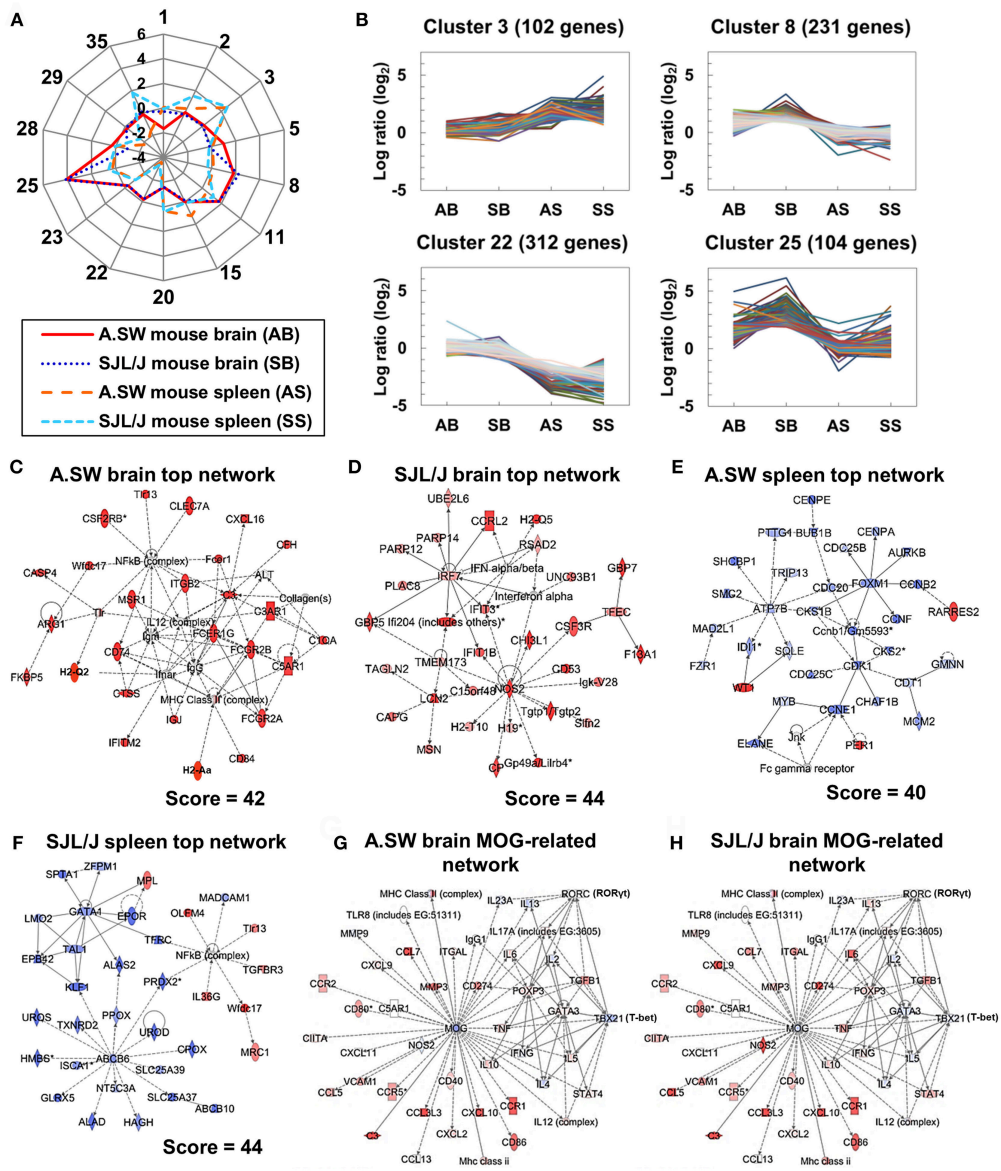
**(A,B)** *K*-means clustering for brain and spleen microarray data from two models. **(A)** A radar chart of 14 cluster center genes whose log ratios were higher than 1 or lower than −1, at least in one organ or in one mouse strain, compared with controls, among the total 35 clusters. **(B)** Dynamic expression patterns of four clusters. AB, SB, AS, and SS indicate A.SW mouse brain, SJL/J mouse brain, A.SW mouse spleen, and SJL/J mouse spleen, respectively. We used microarray data from three PP-EAE and three naïve mice of each strain (the total number of mice = 12). Genes in cluster 3 were up-regulated in the spleens of both models. Genes in cluster 8 were up-regulated in the brains of SJL/J mice. Genes in cluster 22 were down-regulated in the spleens of both models. Genes in cluster 25 were up-regulated in the brains of both models. **(C–H)** Gene networks up-regulated in the brains or down-regulated in the spleens from two models. Transcriptome data at disease peak were clustered and categorized by the Ingenuity Pathway Analysis (IPA) Network Generation. **(C)** The top network in the brains of A.SW mice was categorized as “Cell-To-Cell Signaling and Interaction,” “Hematological System Development and Function,” and “Immune Cell Trafficking,” which were composed of Fc receptor and complement-related genes. **(D)** The top network in SJL/J mouse brain was categorized as “Immunological Disease,” “Endocrine System Disorders,” and “Gastrointestinal Disease,” which were composed of IFN-α/β-induced genes and nitric oxide synthase 2 (*Nos2*). **(E)** The top network in A.SW mouse spleen was categorized as “Cell Cycle,” “Reproductive System Development and Function,” and “Cancer” which were composed of cell cycle-related genes. **(F)** The top network in SJL/J mouse spleen was categorized as “Small Molecule Biochemistry,” “Hematological Disease,” and “Metabolic Disease” which were composed of GATA1- and transporter-related genes. **(G,H)** Up- or down-regulated genes associated with MOG in the brains of A.SW mice **(G)** and SJL/J mice **(H)**. Pro-inflammatory genes were down-regulated in A.SW mice and up-regulated in SJL/J mice, while Th2-associated genes were up-regulated in A.SW mice and down-regulated in SJL/J mice. Red and blue nodes indicate up- and down-regulated genes, respectively. Solid and dashed lines indicated direct and indirect connections, respectively. Score = network score.

To further identify the genes that had distinct expression patterns among the transcriptome data from brains and spleens of A.SW and SJL/J mice with PP-EAE at the disease peak, we divided all genes into 35 clusters, using *k*-means clustering, based on Davies-Bouldin Index (Supplemental Figures 2, 3). The centroid genes of the 14 of 35 clusters showed substantial changes (>2- or <1/2-fold compared with controls), at least, in one organ or in one mouse strain (Supplemental Figures 3, 4, lists of genes in each cluster were shown in Supplemental Tables 2–15). A radar chart for centroid genes of each cluster showed that, in most clusters, gene expression levels in one organ between the two mouse strains were similar, while those between brains vs. spleens were different (a radar chart using the 14 clusters in Figure 3A; radar chart using all the 35 clusters in Supplemental Figure 4). The radar chart showed that, in most clusters, the gene expression patterns in one organ between the two mouse strains were similar, while those between brain and spleen were different.

The genes in the spleen were up-regulated in cluster 3 and down-regulated in cluster 22 in both mouse strains, while there were no substantial changes (log ratios ≈0, compared with controls) in cluster 3 or 22 in the two mouse brain samples (Figure 3B). Cluster 3 included stefins (*Stfa1* and *Stfa2l1*) (Supplemental Table 4), while cluster 22 included erythrocyte-related genes, such as *Kel, Rhd*, and *Gypa* (Supplemental Table 10). On the other hand, the genes in clusters 8 and 25 were up-regulated only in the brains, but not in the spleens, in both mouse strains. Immune response-related genes were included in clusters 8 and 25: *Cxcl9, Cxcl10*, and *Cd3g* in cluster 8; *Lcn2, Cd74*, and *H2-Aa* in cluster 25 (Supplemental Tables 6, 12). Some genes in cluster 8 were up-regulated only in the brains of SJL/J mice (e.g., *Cd3g*: 1.3-fold in A.SW mice, 4.9-fold in SJL/J mice), while several genes in cluster 25 were only down-regulated in A.SW mouse spleens (e.g., *Lcn2*: 0.3-fold). Up-regulation of *Cd3g* in SJL/J mouse brain, but not A.SW mouse brain, was consistent with our previous histological finding that CNS CD3^+^ T cell infiltration was seen only in SJL/J mice (14, 16). Thus, *k*-means clustering clearly showed that groups of immune response-related genes were induced in each organ commonly in two mouse strains, but differentially between brains vs. spleens at the peak of disease progression. However, *k*-means clustering alone was insufficient to identify individual genes that were expressed differentially between the two strains, requiring further analyses.

### Brain Pathway Analysis Revealed That Fc Receptor and Complement-Related Genes Were Up-Regulated in Both Mouse Strains Brains, but Pro-inflammatory Cytokine-Related Genes Were Up-Regulated Only in SJL/J Mice

Using the IPA, we clustered and categorized the genes up- or down-regulated in the brains and spleens of mouse models for PP-EAE (Figures 3C–F). The IPA identified one network in A.SW mice and five networks in SJL/J mice with a high network score (>35). The network identified in the brains of A.SW mice was categorized as “Cell-To-Cell Signaling and Interaction,” “Hematological System Development and Function” and “Immune Cell Trafficking” (Figure 3C). This network contained up-regulation of immune response-related genes: particularly MHC molecules [*H2-Aa* (MHC class II), *H2-Q2* (MHC class I), and *Cd74* (invariant polypeptide of MHC class II molecule)]; immunoglobulin-related genes, including immunoglobulin J chain (*Igj*) (51) and Fc receptors (*Fcgr2a, Fcgr2b, Fcer1a*, and *Fcer1g*); and complement-related genes, including complement component 3 (*C3*), C3a receptor 1 (*C3ar1*), C5a receptor 1 (*C5ar1*), complement component factor h (*Cfh*), and C1q α chain (*C1qa*) (52, 53).

In the brains of SJL/J mice, all five networks with a high network score were associated with immune responses (Figure 3D and Supplemental Figure 5). The top 1 network was categorized as “Immunological Disease,” “Endocrine System Disorders,” and “Gastrointestinal Disease” (Figure 3D). The network was composed of up-regulated genes related to IFN-α/β and nitric oxide synthase 2 (*Nos2*). The top 2 network contained similar genes to the top 1 network of A.SW mouse brain, such as Fc receptor and complement-related genes, while substantial up-regulation of the genes related to IL-6-related genes and costimulatory molecules *Cd80*/*Cd86* (B7-1/B7-2) was seen only in SJL/J mice (Supplemental Figure 5). All the top 3, 4, and 5 networks were associated with pro-inflammatory cytokines, IL-1β, tumor necrosis factor (TNF)-α, and IFN-γ, respectively.

### Spleen Pathway Analysis Revealed That Genes Irrelevant to Immune Responses Down-Regulated in the Spleens of PP-EAE Mice

In the spleen of A.SW mice, we identified three networks, which were composed of mainly down-regulated genes that are irrelevant to immune responses (Figure 3E and Supplemental Figure 6). The top 1 network in the spleens of A.SW mice was categorized as “Cell Cycle,” “Reproductive System Development and Function,” and “Cancer,” including down-regulated genes: cyclin family (*Ccne1, Ccnb1*, and *Ccnb2*) and cell division cycle family (*Cdc20, Cdc25b*, and *Cdc25c*) (Figure 3E). The top 2 and 3 networks were mainly composed of down-regulated GATA binding protein 1 (*Gata1*)-related genes and transporter genes (*Abcb6, Slc25a39*, and *Slc25a37*), respectively (Supplemental Figure 6). On the other hand, in the spleens of SJL/J mice, we identified only one network with a high network score (Figure 3F). The network was categorized as “Small Molecule Biochemistry,” “Hematological Disease,” and “Metabolic Disease,” which were composed of down-regulated genes that were listed in the top 2 and 3 networks of A.SW mouse spleen: *Gata1*-related genes and transporter genes (*Abcb6, Slc25a39*, and *Slc25a37*). *Gata1*-related genes are essential for normal hematopoiesis, particularly erythropoiesis (54), while transporter genes are cell membrane proteins that control the uptake and efflux of various compounds (55, 56). The network also included up-regulated genes, such as Toll-like receptor 13 (*Tlr13*) and transforming growth factor (TGF) β receptor III (*Tgfbr3*).

### MOG-related Pathway Analysis Revealed That Expression of Th1/Th2-Related Genes Differed Between Two Mouse Brains

In both mouse strains, we also determined the gene expression changes in a MOG-related network in the brains, using IPA (Figures 3G,H). *Mog* itself was down-regulated in both mouse strains (A.SW, 0.81-fold, *P* < 0.05; SJL/J, 0.85-fold, *P* < 0.05). The gene expression of several cytokines and chemokines was up-regulated similarly in both mouse strains, including *Cxcl10, Ccr1*, and *Il6*. However, some pro-inflammatory genes, such as *Ifng, Cxcl11, Mmp9*, and *Nos2*, were down-regulated in A.SW mice (Figure 3G), while they were up-regulated in SJL/J mice (Figure 3H). On the other hand, Th2-related genes (*Gata3* and *Il5*, but not *Il4*) were up-regulated in A.SW mice, while they were down-regulated in SJL/J mice.

### PCA of Transcriptome Data of Brains and Spleens Separated Two EAE Groups

**Figure 4:**
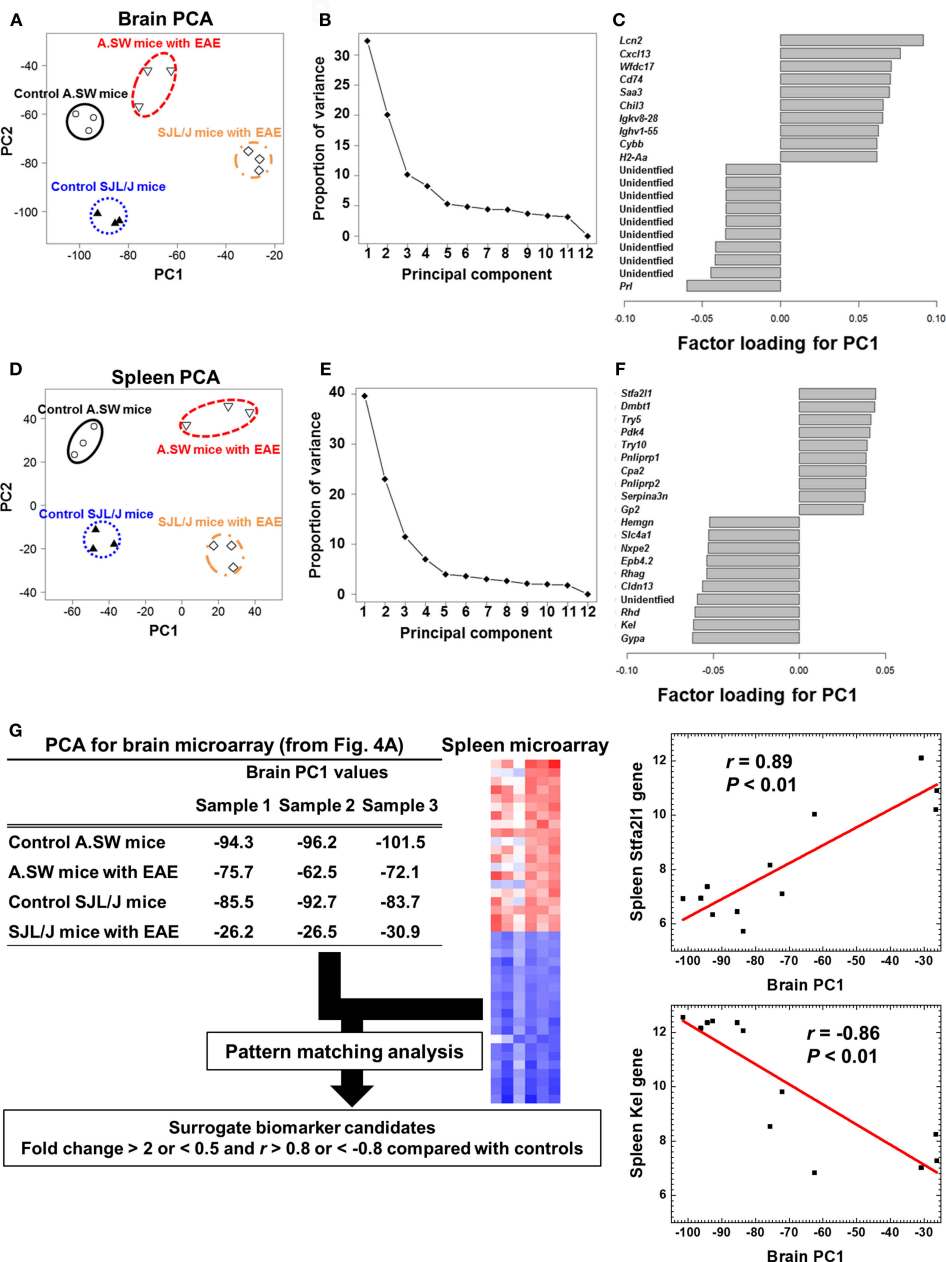
**(A–F)** Principal component analysis (PCA) of transcriptome data of brains and spleens from A.SW and SJL/J mice with PP-EAE and control mice. PCA separated the samples into four groups in both brains **(A)** and spleens **(D)**, where principal component (PC) 1 reflected the presence of EAE, while PC2 reflected strain difference. The proportion of variance showed that PC1 explained variance among samples 33% in the brains **(B)** and 40% in the spleens **(E)**. In the brain **(C)**, factor loading for PC1 showed that up-regulation of immune response-related genes, including lipocalin 2 (*Lcn2*), CXCL13, and immunoglobulins, and down-regulation of prolactin (*Prl*) contributed to PC1 values. In the factor loading for PC1 of spleen PCA **(F)**, stefin A2 like 1 (*Stfa2l1*) and erythrocyte-related genes (*Gypa, Kel*, and *Rhd*) contributed to PC1 distribution positively and negatively, respectively. Transcriptome data from three PP-EAE and three naïve mice of each strain were used. **(G)** A flow chart of pattern matching analysis using brain PC1 values and spleen microarray data from A.SW and SJL/J mice. To find peripheral surrogate biomarkers that reflect the changes in the brain, we conducted pattern matching analysis. Genes whose fold changes were >2 or <0.5 with correlation coefficients of >0.8 or <-0.8 were identified as surrogate marker candidates (Supplemental Table 15). *Stfa2l1* and *Kel* genes, which were up- and down-regulated significantly in the spleens of PP-EAE mice, respectively, were strongly correlated with brain PC1 values (*Stfa2l1*: *r* = 0.89, *P* < 0.01; *Kel*: *r* = −0.86, *P* < 0.01). We used microarray data of brains and spleens of three mice with PP-EAE and three age-matched control mice for each mouse strain (the total number of mice = 12).

To identify the molecules (biomarkers) that distinguish the samples between PP-EAE and control mice, we analyzed microarray data, using an unsupervised approach, PCA (Figure 4). PCA clearly separated brain samples into four groups, each of which was composed of samples from A.SW and SJL/J mice with PP-EAE, and their control mice (Figure 4A), showing that distinct gene expression patterns were present between the four groups. PCA showed that PC1 likely reflected the presence or absence of EAE, while PC2 reflected strain differences. Proportion of variance indicated that PC1 explained 33% of variance among samples, while PC2 explained 20% of variance (Figure 4B). By using factor loading for PC1, we ranked the genes that contributed to the PC1 values (Figure 4C and Supplemental Table 16). Up-regulation of immune response-related genes, including *Lcn2, Cxcl13, Chil3*, immunoglobulins (*Igkv8-28* and *Ighv1-55*), and MHC class II molecule (*H2-Aa*), as well as down-regulation of prolactin (*Prl*), contributed to the PC1 values. Among factor loadings for PC2, although most genes were unidentified, cytochrome P450, family two, subfamily g, polypeptide 1 (*Cyp2g1*), and BPI fold containing family B, member 9B (*Bpifb9b*) were listed (Supplemental Figure 7 and Supplemental Table 17).

PCA of spleen microarray data also separated samples clearly into four groups (Figure 4D). PC1 explained 40% of variance among samples, while PC2 explained 23% of variance (Figure 4E). PC1 reflected the presence or absence of EAE, while PC2 reflected the strain difference. Factor loading showed that stefin A2 like 1 (*Stfa2l1*), deleted in malignant brain tumors 1 (*Dmbt1*), and trypsin genes (*Try5* and *Try10*) contributed to the PC1 values positively, while erythrocyte-related genes (*Gypa, Kel, Rhd*, and *Cldn13*) contributed to the PC1 value negatively (Figure 4F and Supplemental Tables 18, 19). Among top or bottom 100 genes that were listed in factor loading for PC1 values, only three genes, *Chil3, Serpina3n*, and leucine-rich α-2-glycoprotein 1 (*Lrg1*), were in common in the brains and spleens: *Chil3*, which is also known as *Ym1*, is rodent-specific chitinase-like protein and associated with Th2 inflammation (57), *Serpina3n* is an inhibitor of granzyme b (58), and *Lrg1* is related to TGF-β signaling pathway (59). Thus, most CNS gene expression changes seemed to occur independently from those in the peripheral lymphoid organs, during the disease progression of EAE.

### Pattern Matching Analysis Showed Spleen Surrogate Marker Candidates That Reflect the Gene Expression Patterns in the Brain

In the above PCA, we attempted to find peripheral surrogate markers that reflect the changes in the brain. However, we were not able to identify the common genes using factor loading for PC1 among the brain and spleen transcriptome data. Thus, we conducted a pattern matching analysis using brain PC1 values and spleen microarray data from two PP-EAE models and controls; pattern matching analysis allowed us to find splenic genes whose expression patterns matched the PC1 values of brain samples (Figure 4G). When the results were sorted by correlation coefficients (*r* >0.8 or <-0.8) and expression ratios (>2- or <1/2-fold, compared with controls), 333 genes showed strong correlation (Supplemental Table 20). Among the 333 genes, we found 29 splenic genes positively correlated with the brain PC1 values, including adhesion G protein-coupled receptor G2 (*Adgrg2*), *Lrg1*, and phosphoinositide-3-kinase interacting protein 1 (*Pik3ip1*). On the other hand, we found 304 splenic genes negatively correlated with the brain PC1 values, including progestin and adipoQ receptor family member IX (*Paqr9*), RAB3A interacting protein (rabin3)-like 1 (*Rab3il1*), and Josephin domain containing 2 (*Josd2*). Among the positively and negatively correlated genes, *Stfa2l1* (*r* = 0.89) and erythrocyte-related genes, including *Kel* (*r* = −0.86), were listed in the top 10 of factor loading for PC1 in spleen PCA (Figure 4F). Thus, using pattern matching analysis, we were able to find the peripheral surrogate marker candidates among non-immune-related molecules that could reflect the gene expression changes in the brain.

Next, we determined whether the genes listed as peripheral surrogate marker candidates in the mouse spleens (Supplemental Table 20) were also up- or down-regulated in blood transcriptome of human MS patients obtained from the GEO profile database, using a data mining approach with following search keywords: “multiple sclerosis,” “Homo sapiens,” “peripheral blood,” “disease state,” and the 29 up-regulated gene symbols or the 304 downregulated gene symbols (Supplemental Table 21). Among the 29 positively correlated genes listed in Supplemental Table 20, we found that two genes, period circadian clock 1 [*PER1*, also known as microRNA 6883 (*MIR6883*)] and FK506 binding protein 5 (*FKBP5*) were up-regulated in MS peripheral blood, significantly (*P* < 0.01, Supplemental Table 21). Among the 304 negatively correlated genes, we found that only one gene, solute carrier family 16 member 1 [*SLC16A1*, also known as monocarboxylate transporter (MCT) 1] was down-regulated in MS peripheral blood, significantly (*P* < 0.05, Supplemental Table 21). Up-regulation of *Per1* and down-regulation *SLC16A1* were found in the data set (60) from 12 MS patients compared with 15 unaffected controls, whose other clinical data were not available. Upregulation of *FKBP5* was found in the data set of peripheral blood cells from three MS patients with high serum levels of transmembrane-type semaphorin (Sema4A) (but not from MS patients with low Sema4A levels), compared with four healthy controls with low serum levels of Sema4A (61).

### Validation of Transcriptome Data of Biomarker Candidates in the Brains and Spleens

To validate transcriptome data of brain and spleen samples, we conducted real-time PCR for the representative genes listed in the clustering, PCA factor loading and pattern matching data (Supplemental Figure 8). The expression patterns of *Cxcl13, Lcn2*, and *Cd3g* in the brain samples and those of *Kel* and *Stfa2l1* in the spleen samples between microarray and real-time PCR data were consistent. The levels of *Cxcl13, Lcn2*, and *Cd3g* in the brains with PP-EAE were higher in SJL/J mice than in A.SW mice. Similarly, in the spleen, the expression of *Stfa2l1* was also up-regulated. On the other hand, *Kel* was down-regulated in the spleens of both PP-EAE mice. Expression of *Lcn2* in the spleens was significantly down-regulated in A.SW mice and up-regulated in SJL/J mice.

## Discussion

There have been controversies on whether MS is a heterogeneous or homogenous disease (12, 47, 62). The heterogeneity of MS can be further discussed in three aspects; whether MS is heterogeneous or homogenous (1) “in time (during the time course),” (2) “in space” in individual patients with MS, and (3) in the pathology type among MS patients. These theories are based on mainly clinical neuroimaging and neuropathological studies of human MS cases, which have limitations; for example, longitudinal biopsies of CNS tissues are not possible in one individual. While such human studies have often supported one theory, and tended to deny the other theories, this can be due to the limitation of the methodology employed in each study. Our current computational studies of two EAE models for progressive MS can be a proof of concept that autoimmune demyelinating diseases can be either homogenous or heterogeneous in all three aspects, to some extent.

First, “Is MS a heterogeneous in time?” in other word, “Is MS a 1-stage or 2-stage disease (47)?” The “1-stage” disease theory is that the pathophysiology (effector mechanism) of MS is the same during the entire course of MS in individual patients. The “2-stage” disease theory is that CNS tissue damage is caused by inflammation in Stage 1, while neurodegeneration in Stage 2 is independent of inflammation, leading to disease progression. While some neuropathology studies in MS supported the 1-stage disease theory, neuroimaging and clinical studies, such as drug responses and epidemiological data, supported the 2-stage disease theory (63, 64).

In our current study, when we assessed kinetics of IFN-γ and IL-17 levels, these pro-inflammatory cytokines were associated with disease activities in RR-EAE and PP-EAE in SJL/J mice (common effector mechanism in initiation, acute attack, and disease progression), while IFN-γ and IL-17 levels in PP-EAE in A.SW mice were up-regulated only in disease initiation, but declined at disease peak. Since these results suggested that another effector mechanism independent of the pro-inflammatory responses could contribute to disease progression in A.SW mice, we further conducted transcriptome analyses of the CNS at disease peak of PP-EAE in both mouse strains. Volcano plots of transcriptome data showed the different number of up- or down-regulated genes between brains and spleens or between A.SW and SJL/J mice. While many genes were up-regulated in the brains and down-regulated in the spleens, down-regulation of genes in the spleen may be associated with splenic atrophy (16). Heat maps showed highly up-regulated genes in each brain and spleen of two mouse strains as a result of “supervised” two-way comparison. In the brains of both models, several genes were up-regulated in common. Among the genes, *Lcn2* was the most highly up-regulated gene, which has been reported as an immune mediator of EAE and MS (65, 66). Glycoprotein nonmetastatic melanoma B (*Gpnmb*) is a type I transmembrane protein which works in various biological processes, such as inflammation (67, 68). Activation of complement components, including *C3*, plays a pivotal role by recruiting inflammatory cells, increasing myelin phagocytosis by macrophages, and exerting direct cytotoxic effects on oligodendrocytes (69). *Cxcl13* attracts B lymphocytes and Th cells via chemokine receptor CXCR5 (70) and can be used as a biomarker of inflammation in MS (71). Since *Cybb*/*Nox2* was also up-regulated, oxidative stress may be related to damage in the brain (72).

In the spleens of both models, we found significant down-regulation of *Kel, Rhd, Gypa*, and *Cldn13*, which have been reported as erythrocyte-related genes (Figures 2E,F) (49). This is consistent with splenic pathway analysis data (Figure 3F and Supplemental Figure 6A), in which we found down-regulation of *Gata1*-related genes that are essential for erythropoiesis (54). *Stfa2l1*, which acts as a cathepsin inhibitor, was up-regulated in common and could regulate antigen presentation processes involved in immune response and autoimmune diseases (73). Interestingly, two neutrophil-associated proteins, *Ltf*, and *Ly6g* were down-regulated in A.SW spleens, but up-regulated in SJL/J spleens. *Ltf* is a protein contained in secondary granules of neutrophils and can ameliorate the signs of EAE (74), while *Ly6g* is expressed in neutrophils and can regulate leukocyte activation and adhesion (75). Distinct expression of these genes suggested the different role of neutrophils in two PP-EAE models.

**Figure 5:**
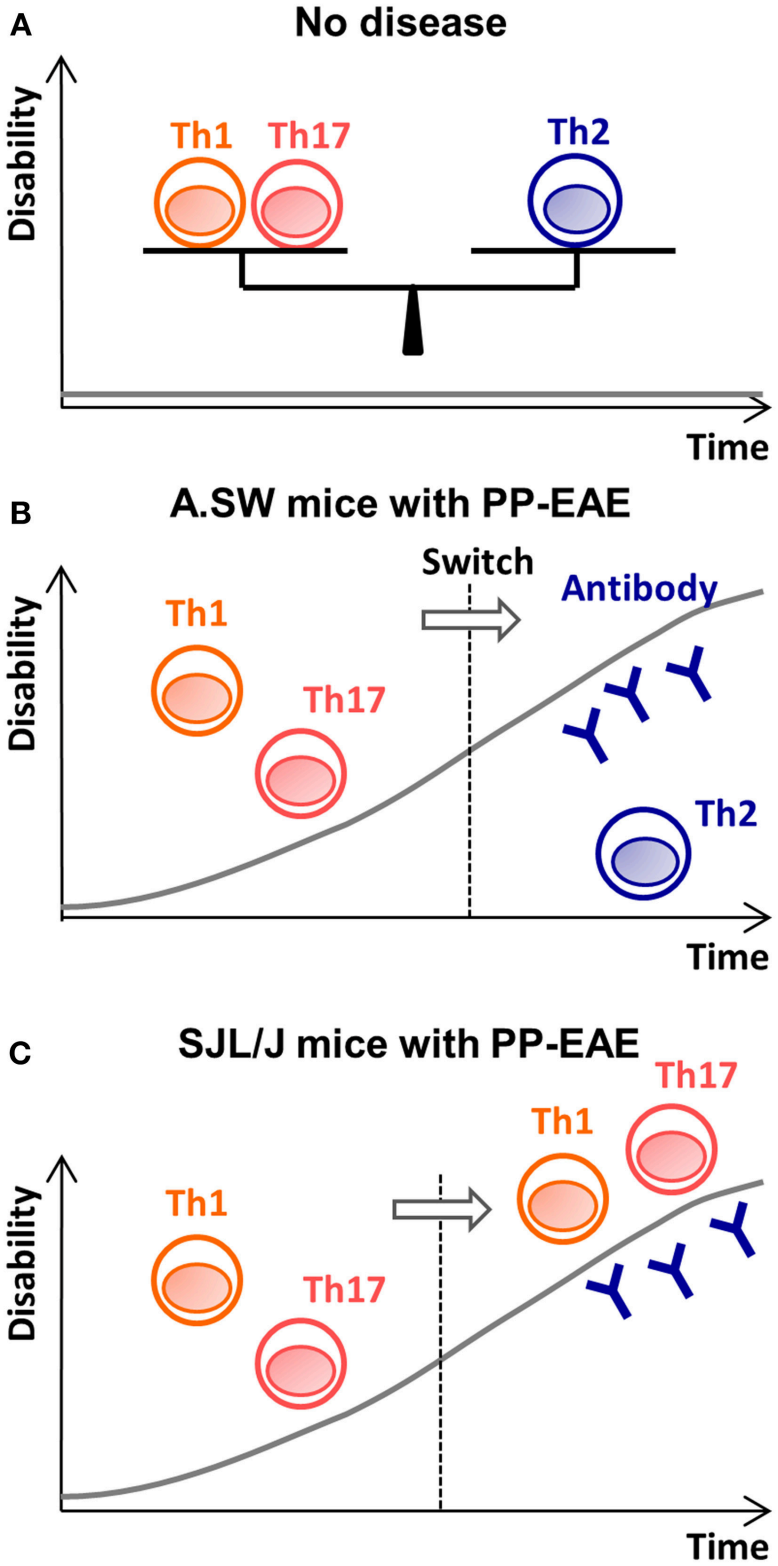
The disease theories in A.SW and SJL/J mice with PP-EAE. **(A)** When Th1/Th17 cells vs. Th2 cells is well-balanced, no disease is induced. **(B)** In A.SW mice, increased Th1/Th17 immune responses initiate disease. Later, the Th1/Th17 responses decline, and increased Th2 immune responses help auto-antibody production. Here, effector mechanism switches from proinflammatory Th1/Th17 responses (first stage) to antibody-mediated pathology (second stage). **(C)** In SJL/J mice, while Th1/Th17 cells play an effector role during the entire disease course, anti-myelin antibody also contributes to disease progression at the “second” stage.

Interestingly, our bioinformatics analyses, including pathway analyses and PCA, demonstrated that antibody-mediated pathophysiology (composed of immunoglobulin-, complement-, and FcR-related molecules) seemed to be active in both mouse strains. We confirmed the presence of immunoglobulin deposition and complement receptor positive cells by immunohistochemistry (data not shown). In PP-EAE in SJL/J mice, bioinformatics analyses also showed that the top network present in the CNS was associated with pro-inflammatory responses composed of most major inflammatory pathways, including those of IFN-α/β, IFN-γ, IL-17, IL-6, TNF-α, and IL-1β. Thus, in SJL/J mice, the pro-inflammatory effector mechanism could play a pathogenic role during the entire course (here, the disease might look a homogeneous disease, if one focuses only on these pro-inflammatory responses), while the antibody-mediated effector mechanism also seemed to be active at disease peak in both mouse strains (Figure 5). On the other hand, downregulation of prolactin also contributed to the separation between EAE and control groups in PCA. Prolactin is secreted not only by the anterior pituitary but also extra-pituitary tissues including immune cells, while prolactin receptor is found on lymphocytes and other immune cells (76). Prolactin has several roles including immunomodulation and remyelination. Although our current PCA demonstrated downregulation of prolactin could be associated with EAE progression, prolactin has been suggested to exacerbate other EAE models (77). Similarly, in human MS, an association between prolactin levels and disease activities remains controversial (76). Ysrraelit and Correale proposed that prolactin may exert dual and opposing effects in MS and that caution must be taken when prolactin levels are manipulated in MS.

However, these results do not deny the possibility of disease progression based on the 1-stage disease theory, since uncontrolled pro-inflammatory cellular responses alone can lead to disease progression regardless of the presence of involvement of antibody and complement, in theory. Indeed, many experimentally proven encephalitogenic antigens, including MBP, PLP, and neurofilament light chain (NF-L), can induce only pathogenic T cell responses that cause neurological deficits (antibodies to MBP, PLP, and NF-L do not cause tissue or cell injury because their epitopes are not expressed on cell surface) (78).

Our bioinformatics transcriptome analyses also addressed the second question, “Is MS pathology homogeneous or heterogeneous in space, in one patient, and at one time point?” or “Is there only one pathology (one effector mechanism) present in the CNS or are multiple different pathologies simultaneously present in the CNS in one patient?” At the disease peak of A.SW mice with PP-EAE, we identified only one major effector mechanism (= antibody and complement-mediated tissue damage), while two effector mechanisms may be involved in SJL/J mice: (1) antibody and complement-mediated tissue damage and (2) pro-inflammatory CD3^+^ T cell-mediated tissue damage. Histologically, in SJL/J mice, we found that some areas showed antibody deposition without T cell infiltration and other areas contained T cell infiltration with or without antibody deposition (data not shown). These results suggested that CNS demyelinating pathology can be homogenous (contain one pathology type) or can be heterogeneous (contain more than two pathologies) in a single individual. In most CNS peptide-induced EAE models, pathology has been shown to be homogeneous since most peptides are either major T cell epitopes or B cell epitopes, but not both. In contrast, multiple effector mechanisms as well as heterogeneous neuropathology can be present in one single EAE model, when EAE is induced with encephalitogens that have both T-cell and B-cell epitopes [for example, MOG_92−106_ (current experiment) and brain homogenates].

Our results also addressed the third question, “Is MS pathophysiology homogeneous (common) in all MS patients, or are there heterogeneities in pathophysiology among MS patients?” Our current experiments showed that a single encephalitogen (MOG_92−106_) can cause two different pathophysiologies (pro-inflammatory and antibody-mediated). This supports a concept and clinical pathology findings that MS neuropathology is heterogeneous. However, this does not deny the presence of possible common (homogeneous) pathologic component of demyelinating diseases. For example, in our current studies, the antibody-mediated tissue damage seemed to be a common effector mechanism in two PP-EAE models; we also found that some genes, such as *Lcn2* and *Chil3*, were commonly up-regulated in two models. In addition, common neuropathology and effector components have been demonstrated among several different EAE models that were induced with different encephalitogenic antigens. For example, EAE can be induced in SJL/J mice or C57BL/6 mice, using different encephalitogens, such as PLP_139−151_, PLP_178−192_, MOG_92−106_, and MOG_35−55_ (13, 79). Here, neuropathology and pro-inflammatory immune responses in EAE induced with these different peptides were overall indistinguishable (14, 28, 80). In this context, it should also be noted that virus-induced demyelinating models share a common pathology and effector mechanism (3). Therefore, in theory, the cause of MS (several different autoantigens or even viruses) can be homogeneous or heterogeneous. Here, one autoantigen can cause different (heterogeneous) pathology depending on the genetic background or the presence of adjuvant (which mimics polymicrobial infection). On the other hand, several different autoantigens (different causes) can induce the same (homogeneous) pathology in the CNS of MS patients.

In this study, we have also conducted splenic transcriptome analyses to find peripheral surrogate markers that reflect the change in the CNS. In clinical studies in MS, while some reports showed that immune profiles in the blood reflected disease activity, others showed that peripheral profiles did not reflect the change in the CNS (81, 82). Using heat map and network/pathway analyses, we found that highly up-regulated and down-regulated genes and pathways were different between the spleens and brains in both mouse strains. Interestingly, in splenic pathway analysis, both mouse strains had down-regulation of GATA1-related genes and transporter genes (Figure 3F and Supplemental Figures 6A,B), while only A.SW mice had down-regulation of a network related to the cell cycle (Figure 3E). Cell cycle arrest could occur in the atrophic spleen with apoptosis in A.SW mice with progressive EAE, as we reported previously (16). Thus, A.SW mice had an additional major change in the network, comparing with SJL/J mice; this is in contrast to the CNS network profiles where SJL/J mice had an additional effector mechanism, comparing with A.SW mice.

We also conducted PCA using splenic transcriptome data from A.SW and SJL/J mice. Although PC1 values reflected the presence or absence of EAE in both the CNS and spleens, we did not find commonly up- or down-regulated genes contributing to PC1 between the brain and spleen factor loading for PC1. Thus, both supervised two-way comparison and unsupervised PCA showed that there were only three genes in common between the brains and spleens: *Chil3, Serpina3n*, and *Lrg1*. This could be consistent with a hypothesis that immune responses in progressive MS are sequestered from the systemic immune responses; the pathophysiology in the CNS at this stage may occur within the intact blood-brain barrier, and be independent of systemic immune responses (83).

On the other hand, our pattern matching analyses between the brain PC1 (that may reflect brain disease) and spleen transcriptome data showed that the pattern changes in a set of peripheral genes were significantly correlated with the brain PC1 values. Interestingly, the splenic genes showed the significant correlation with brain PC1 values were not immune-mediated genes. Although the causal relationship between the brain pathophysiology and splenic transcriptome changes is unclear, these set of splenic genes could be used as surrogate markers, or may be the contributing factor and/or outcomes of the pathology in the CNS. Among the genes listed as peripheral surrogate marker candidates (Supplemental Table 20), three genes, *PER1, FKBP5*, and *SLC16A1* were up- or down-regulated significantly in the peripheral blood data sets from MS patients, although these data sets were from small numbers of patients with unknown clinical histories (Supplemental Table 21). *PER1* encodes microRNA6883, which is associated with circadian rhythm (84). *FKBP5* is a member of immunophilin protein family which works in immunoregulation and interacts with the progesterone receptor and GATA-2 (85). *SLC16A1* encodes the MCT1, whose inhibition has been found to modulate T cell responses (86, 87). This is the first report showing the association between these three genes and MS. Peripheral surrogate marker candidates identified in this study might be worth monitoring in MS blood samples.

## Data Availability

The datasets generated for this study can be found in Gene Expression Omnibus, GSE99300.

## Author Contributions

AM, JA, and IT conceived and supervised the project. SO and NK designed the experiments. SO, FS, and NM conducted the experiments. SO, A-MP, MF, and UC conducted bioinformatics analyses. SO and IT wrote the manuscript. All authors read and approved the final manuscript.

### Conflict of Interest Statement

The authors declare that the research was conducted in the absence of any commercial or financial relationships that could be construed as a potential conflict of interest.
