# Supplementary material for: Bioinformatics Analyses Determined the Distinct CNS and Peripheral Surrogate Biomarker Candidates Between Two Mouse Models for Progressive Multiple Sclerosis
Source: Front Immunol. 2019 Mar 19;10:516. doi: 10.3389/fimmu.2019.00516 (PMC6434997; doi:10.3389/fimmu.2019.00516)
Supplement: Supplementary file 1 [file Data_Sheet_1.docx]

**Supplementary data**

**Table of Contents** …… 1

**I. Supplemental Figures**

Supplemental Figure 1. Clinical course and weight change of SJL/J mice with primary progressive (PP)-experimental autoimmune encephalomyelitis (EAE) …… 3

Supplemental Figure 2. Davies-Bouldin Index of *k*-means clustering for microarray data from the brains and spleens of A.SW and SJL/J mice with PP-EAE …… 4

Supplemental Figure 3. Expression pattern of 35 clusters divided by *k*-means clustering for microarray …… 5

Supplemental Figure 4. Radar chart of 35 clusters divided by *k*-means clustering …… 7

Supplemental Figure 5. Four of top 5 networks regulated in the brains of SJL/J mice with PP-EAE …… 8

Supplemental Figure 6. Two of top 5 networks regulated in the spleens of A.SW mice with PP-EAE …… 9

Supplemental Figure 7. Factor loading for principal component (PC) 2 in the PCAs of the brains and spleens of A.SW and SJL/J mice with PP-EAE …… 10

Supplemental Figure 8. Gene expression of representative molecules in the brain and spleen of PP-EAE models …… 11

**II. Supplemental Tables**

Supplemental Table 1. Gene abbreviations …… 12

Supplemental Table 2. List of genes in cluster 1 …… 16

Supplemental Table 3. List of genes in cluster 2 …… 22

Supplemental Table 4. List of genes in cluster 3 …… 27

Supplemental Table 5. List of genes in cluster 5 …… 29

Supplemental Table 6. List of genes in cluster 8 …… 33

Supplemental Table 7. List of genes in cluster 11 …… 37

Supplemental Table 8. List of genes in cluster 15 …… 40

Supplemental Table 9. List of genes in cluster 20 …… 45

Supplemental Table 10. List of genes in cluster 22 …… 48

Supplemental Table 11. List of genes in cluster 23 …… 52

Supplemental Table 12. List of genes in cluster 25 …… 56

Supplemental Table 13. List of genes in cluster 28 …… 58

Supplemental Table 14. List of genes in cluster 29 …… 64

Supplemental Table 15. List of genes in cluster 35 …… 72

Supplemental Table 16. Top and bottom 100 genes listed in factor loading for PC1 of brain PCA …… 74

Supplemental Table 17. Top and bottom 100 genes listed in factor loading for PC2 of brain PCA …… 78

Supplemental Table 18. Top and bottom 100 genes listed in factor loading for PC1 of spleen PCA …… 82

Supplemental Table 19. Top and bottom 100 genes listed in factor loading for PC2 of spleen PCA …… 86

Supplemental Table 20. The list of spleen surrogate marker candidates suggested by pattern matching analysis between brain PC1 values and spleen microarray data …… 90

Supplemental Table 21. A result of data mining of peripheral surrogate marker candidates, which are up- or down-regulated in mouse spleen, on the human blood transcriptome database of MS patients …… 96

**
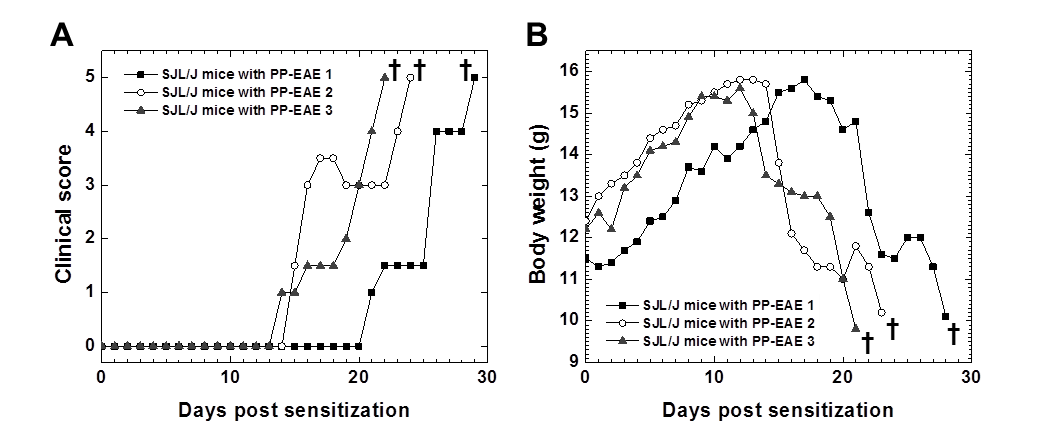
**

**Supplemental Figure 1.** Clinical course and weight change of SJL/J mice with primary progressive (PP)-experimental autoimmune encephalomyelitis (EAE). Mice were sensitized with myelin oligodendrocyte glycoprotein (MOG)_92-106_ and curdlan. Clinical score was determined by EAE score: 0; no clinical disease, 1; loss of tail tonicity, 2; mild hind leg paresis, 3; moderate hind leg paralysis, 4; complete paraplegia, 5; moribund state. Mice developed PP-EAE around 2 weeks after MOG sensitization (A). Disease progressed until mice became moribund without remission. Clinical scores continuously increased, which mirrored weight loss (B). Shown are data of three representative mice (mouse no. 1, no. 2, and no. 3) in two independent experiments.

**Supplemental Figure 2.** Davies-Bouldin Index of *k*-means clustering for microarray data from the brains and spleens of A.SW and SJL/J mice with PP-EAE.

**
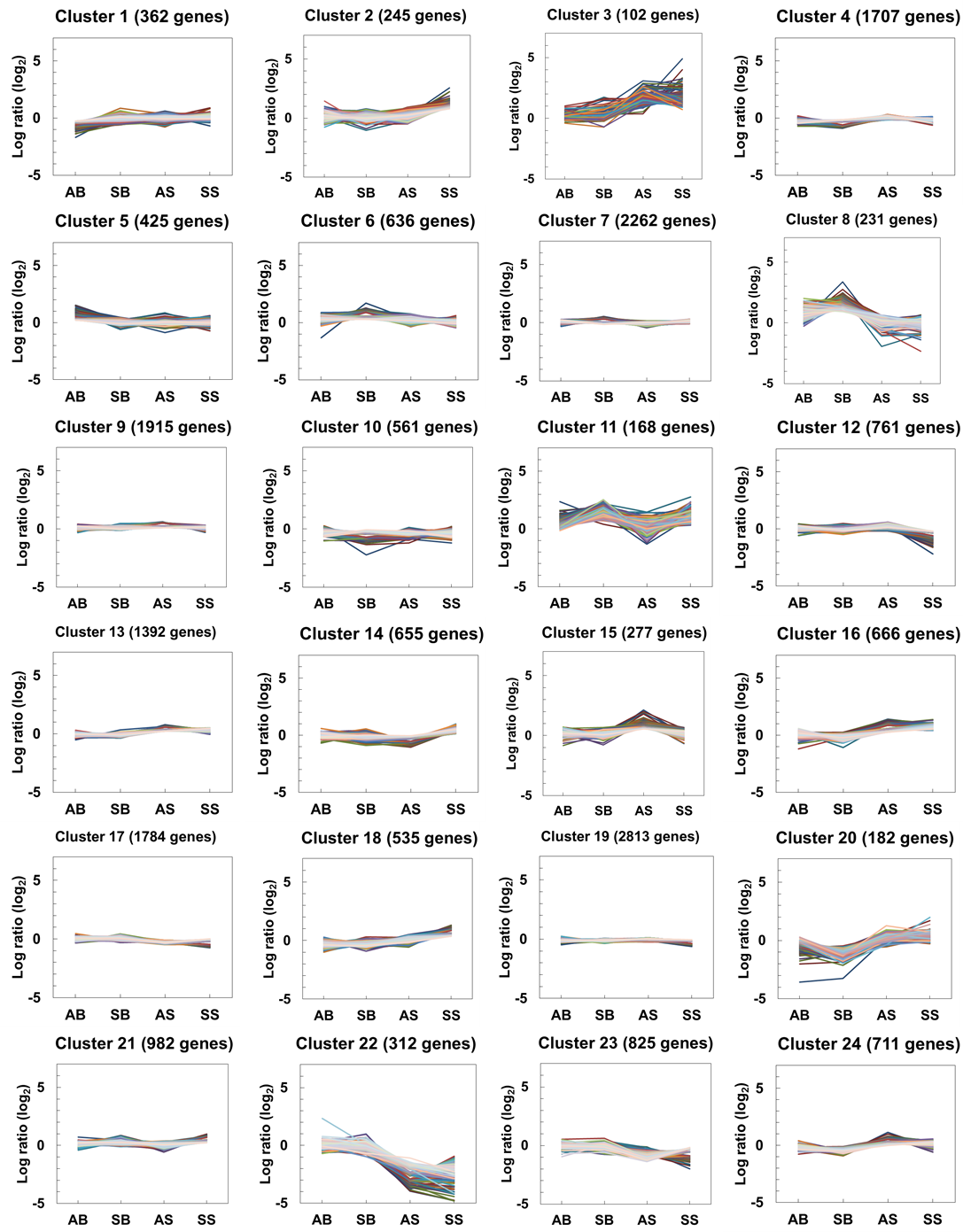
**

**
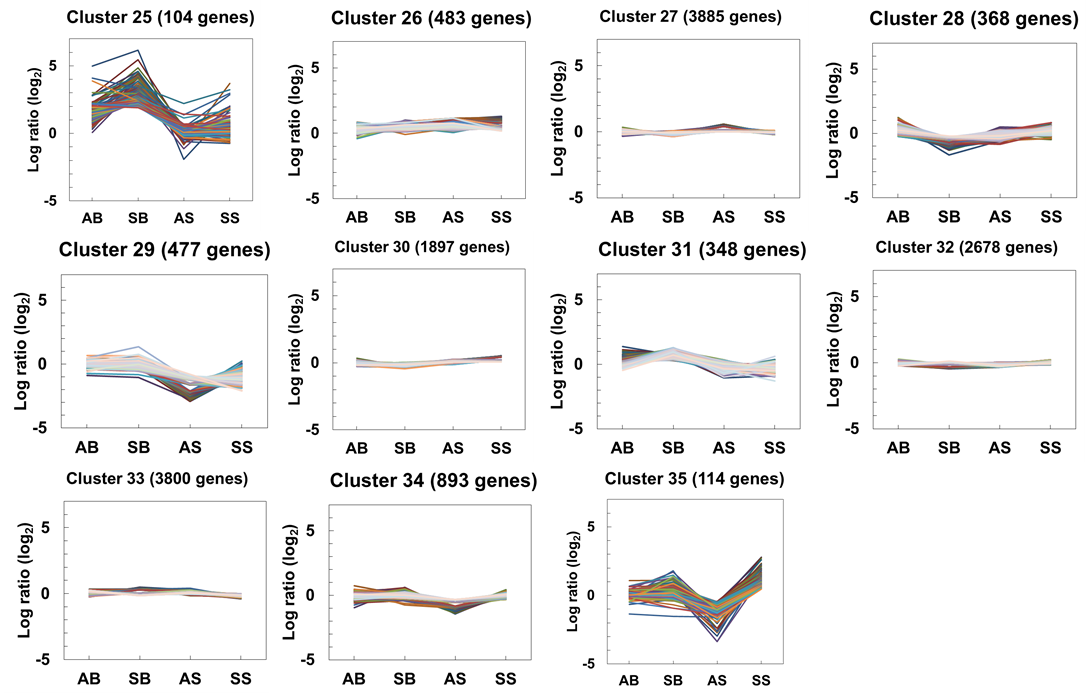
**

**Supplemental Figure 3.** *K*-means clustering of microarray data of brains and spleens from A.SW and SJL/J mice with PP-EAE. Immune response-related genes were included into clusters 8 and 25, such as *Cxcl9*, *Cxcl10*, and *Cd3g* in cluster 8 and *Lcn2*, *Cd74*, and *H2-Aa* in cluster 25. In most clusters, the gene expression patterns in one organ between the two mouse strains were similar [e.g. between A.SW mouse brain (AB) and SJL/J mouse brain (SB)], while those between brains versus spleens were different [i.e. AB and SB versus A.SW mouse spleen (AS) and SJL/J mouse spleen (SS)]. Samples from three PP-EAE and three naïve mice of each strain were used.

**Supplemental Figure 4.** A radar chart of 35 clusters separated by *k*-means clustering. The number at each vertex is the cluster number (1 to 35), while the numbers along the axis (-4 to 5) are log ratios, compared with controls. Similar patterns were showed in one organ between A.SW and SJL/J mice, while the different patterns were showed between the brains and spleens.


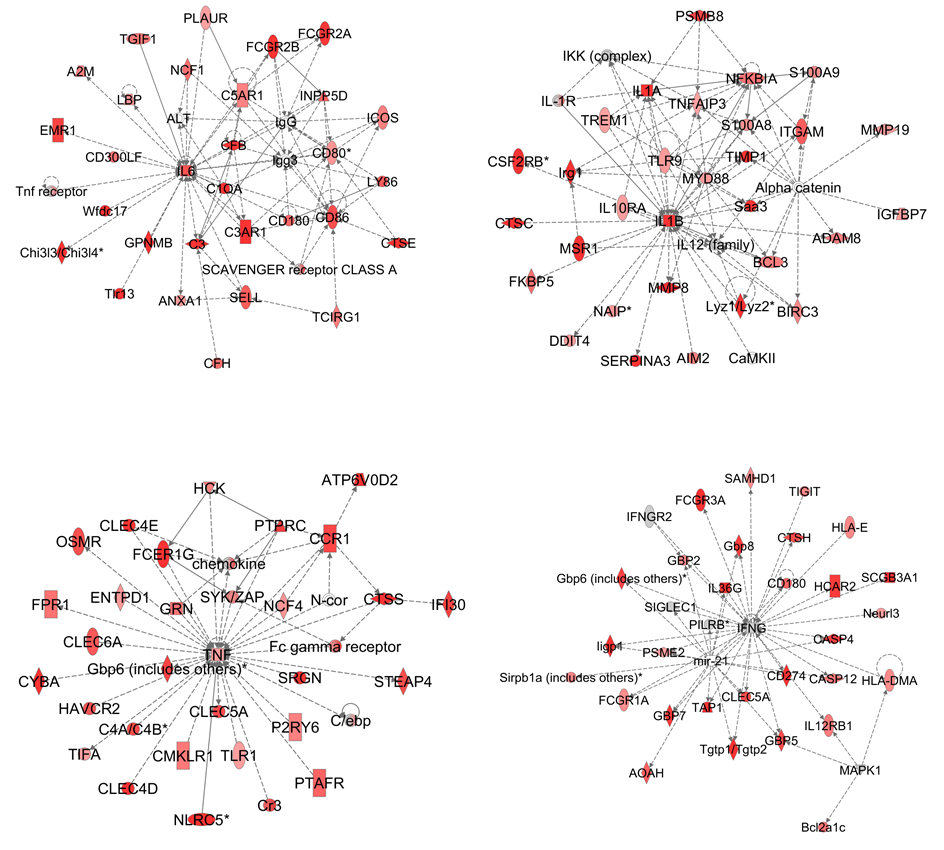


**H2-DMa**

**H2-T23**

**Score = 35**

**Score = 37**

**Score = 37**

**Score = 35**

**D**

**C**

**A**

**B**

**Supplemental Figure 5.** Four of top 5 networks regulated in the brains of SJL/J mice with PP-EAE. A) Top 2 network was associated with ‘Cellular Movement’, ‘Immune Cell Trafficking’, and ‘Hematological System Development and Function’, which were composed of IL-6, Fc receptor, and complement-related genes. B) Top 3 network was associated with ‘Infectious Disease’, ‘Cellular Movement’, and ‘Immune Cell Trafficking’, which were composed of IL-1-associated genes. C) Top 4 network was associated with ‘Immunological Disease’, ‘Cellular Function and Maintenance’, and ‘Inflammatory Response’, which were composed of TNF-α-associated genes. D) Top 5 network was associated with ‘Endocrine System Disorders’, ‘Gastrointestinal Disease’, and ‘Immunological Disease’, which were composed of IFN-γ-associated genes.

**A**

**B**


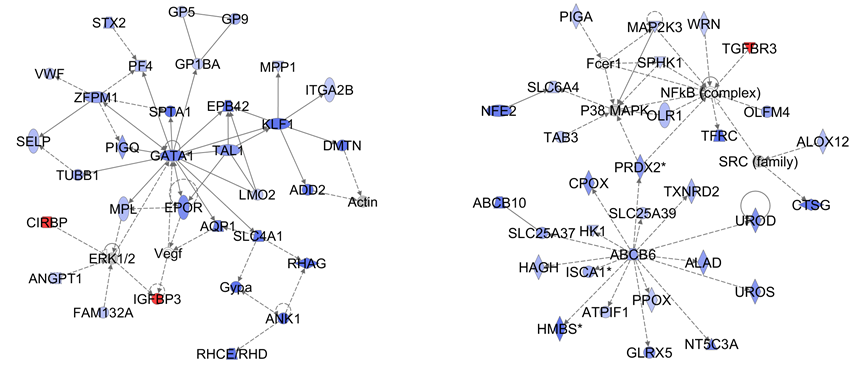


**Score = 35**

**Score = 37**

**Supplemental Figure 6.** Two of top 5 networks regulated in the spleens of A.SW mice with PP-EAE. A) Top 2 network was associated with ‘Hematological Disease’, ‘Organismal Injury’, and Abnormalities’, which were composed of GATA-1-related genes. B) Top 3 network was associated with ‘Hematological Disease’, ‘Metabolic Disease’, and ‘Small Molecule Biochemistry’, which were composed of transporter and MP kinase-related genes.


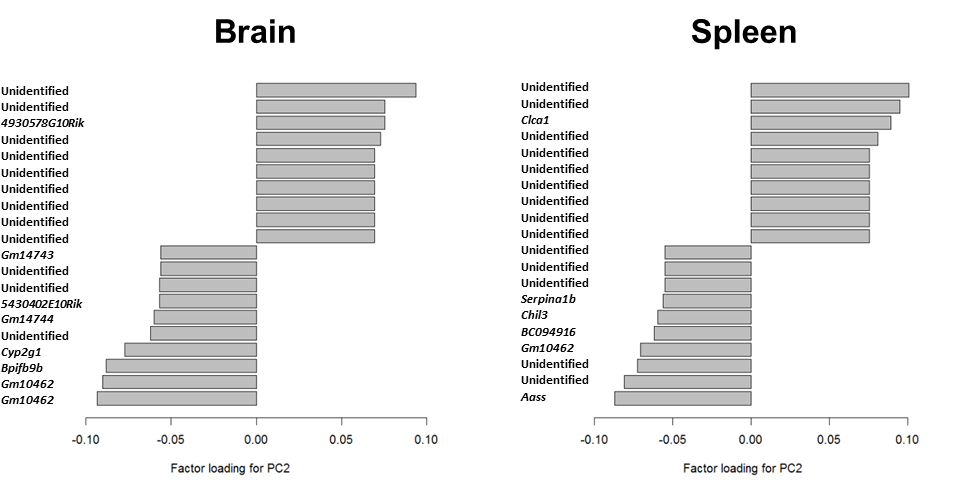


**Factor loading for PC2**

**Factor loading for PC2**

**Supplemental Figure 7.** Factor loading for principal component (PC) 2 in the PCAs of the brains and spleens of A.SW and SJL/J mice with PP-EAE.


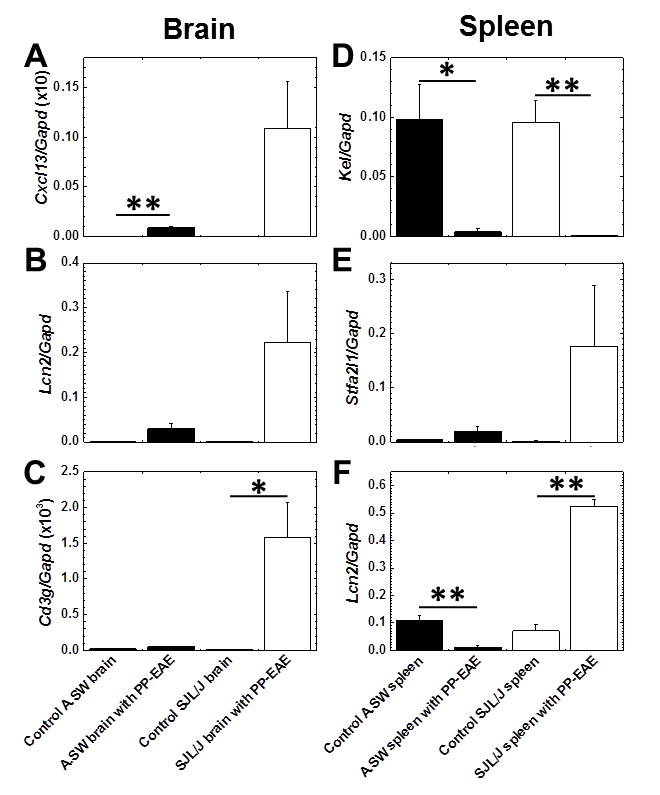


**Supplemental Figure 8.** Gene expression of representative molecules in the brain (A-C) and spleen (D-F) of PP-EAE models. The gene expression was semi-quantified by real-time PCR. *Cxcl13*, *Lcn2* and *Cd3g* in the brains were up-regulated slightly in A.SW mice and highly in SJL/J mice with PP-EAE. In the spleens, although the expression pattern of *Stfa2l1* was similar to those of *Cxcl13*, *Lcn2*, and *Cd3g* in the brains, *Kel* was down-regulated in the spleens of both PP-EAE models. *Lcn2* was down-regulated in the spleens of A.SW mice with PP-EAE and up-regulated in those of SJL/J mice with PP-EAE. The expression patterns of above genes were consistent with transcriptome data by microarrays. Samples from three PP-EAE and three naïve mice of each strain were used. **P* < 0.05, ***P* < 0.01, ANOVA.

**Supplemental Table 1.** Gene abbreviations

| **Gene symbol** | **Gene name / Synonyms** |
| --- | --- |
| *A2m* | α-2-macroglobulin |
| *Abca8a* | ATP-binding cassette, sub-family A (ABC1), member 8a |
| *Abcb10* | ATP-binding cassette, sub-family B (MDR/TAP), member 10 |
| *Abcb6* | ATP-binding cassette, sub-family B (MDR/TAP), member 6 |
| *Add2* | adducin 2 (beta) |
| *Alad* | aminolevulinate, δ-, dehydratase |
| *Alox12* | arachidonate 12-lipoxygenase |
| *Alt* | glutamic pyruvic transaminase, soluble (*Gpt*) |
| *Angpt1* | angiopoietin 1 |
| *Ank1* | ankyrin 1, erythroid |
| *Anxa1* | annexin A1 |
| *Apod* | apolipoprotein D |
| *Aqp1* | aquaporin 1 |
| *Arc* | activity regulated cytoskeletal-associated protein |
| *Arg1* | arginase, liver |
| *Atp6v0d2* | ATPase, H+ transporting, lysosomal V0 subunit D2 |
| *Atp7b* | ATPase, Cu++ transporting, β polypeptide |
| *Atpif1* | ATPase inhibitory factor 1 |
| *Aurkb* | aurora kinase B |
| *Bub1b* | budding uninhibited by benzimidazoles 1 homolog, β (S. cerevisiae) |
| *C1qa* | complement component 1, q subcomponent, α polypeptide, C1q A chain |
| *C3* | complement component 3 |
| *C3ar1* | complement component 3a receptor 1 |
| *C5ar1* | complement component 5a receptor 1 |
| *Capg* | capping protein (actin filament), gelsolin-like |
| *Car2* | carbonic anhydrase 2 |
| *Car8* | carbonic anhydrase 8 |
| *Casp4* | caspase 4, apoptosis-related cysteine peptidase |
| *Ccl3* | chemokine (C-C motif) ligand 3 (*Mip1a*) |
| *Ccl5* | chemokine (C-C motif) ligand 5 (RANTES) |
| *Ccnb1* | cyclin B1 |
| *Ccnb2* | cyclin B2 |
| *Ccne1* | cyclin E1 |
| *Ccnf* | cyclin F |
| *Ccrl2* | chemokine (C-C motif) receptor-like 2 |
| *Cd180* | CD180 antigen (*Ly78*) |
| *Cd300lf* | CD300 antigen like family member F (CLM-1/IREM1) |
| *Cd53* | CD53 antigen (*Ox-44*) |
| *Cd74* | CD74 antigen (invariant polypeptide of major histocompatibility complex, class II antigen-associated) (CLIP) |
| *Cd80* | CD80 antigen (B7-1/ Ly53) |
| *Cd84* | CD84 antigen (SLAMF5) |
| *Cd86* | CD86 antigen (B7-2/ Ly58) |
| *Cdc20* | cell division cycle 20 |
| *Cdc25b* | cell division cycle 25B |
| *Cdc25c* | cell division cycle 25C |
| *Cdk1* | cyclin-dependent kinase 1 |
| *Cdt1* | chromatin licensing and DNA replication factor 1 |
| *Cenpa* | centromere protein A |
| *Cenpe* | centromere protein E |
| *Cfb* | complement factor B |
| *Cfh* | complement component factor h |
| *Chaf1b* | chromatin assembly factor 1, subunit B (p60) |
| *Chi1* | chitinase-like 1 (*Chi3l1*/BRP-39/YKL-40) |
| *Chil3* | chitinase-like 3 (*Chi3l3*/Ym1) |
| *Cirbp* | cold inducible RNA binding protein |
| *Cks1b* | CDC28 protein kinase 1b |
| *Cks2* | CDC28 protein kinase regulatory subunit 2 |
| *Cldn13* | claudin 13 |
| *Clec7a* | C-type lectin domain family 7, member a (dectin-1) |
| *Coro6* | coronin 6 |
| *Cp* | ceruloplasmin |
| *Cpox* | coproporphyrinogen oxidase |
| *Csf2rb* | colony stimulating factor 2 receptor, β, low-affinity (granulocyte-macrophage) |
| *Csf3r* | colony stimulating factor 3 receptor (granulocyte) |
| *Ctse* | cathepsin E |
| *Ctsg* | cathepsin G |
| *Ctss* | cathepsin S |
| *Cxcl13* | chemokine (C-X-C motif) ligand 13 |
| *Cxcl16* | chemokine (C-X-C motif) ligand 16 |
| *Cybb* | cytochrome b-245, beta polypeptide (*Nox2*) |
| *Cyp2e1* | cytochrome P450, family 2, subfamily e, polypeptide 1 |
| *Ddit4* | DNA-damage-inducible transcript 4 |
| *Depdc1a* | DEP domain containing 1a |
| *Dmbt1* | deleted in malignant brain tumors 1 |
| *Dmtn* | dematin actin binding protein |
| *Elane* | elastase, neutrophil expressed |
| *Emr1* | EGF-like module containing, mucin-like, hormone receptor-like sequence 1 |
| *Epb4.2* | erythrocyte protein band 4.2 (*Epb42*) |
| *Epor* | erythropoietin receptor |
| *Erk1* | mitogen-activated protein kinase 3 (*Mapk3*) |
| *Ermap* | erythroblast membrane-associated protein |
| *Errfi1* | ERBB receptor feedback inhibitor 1 |
| *Esco2* | establishment of cohesion 1 homolog 2 (S. cerevisiae) |
| *F13a1* | coagulation factor XIII, A1 subunit |
| *Fabp7* | fatty acid binding protein 7, brain |
| *Fam132a* | family with sequence similarity 132, member A |
| *Fcer1g* | Fc receptor, IgE, high affinity I, γ polypeptide |
| *Fcgr2b* | Fc receptor, IgG, low affinity Iib (*Fcgr2a*) |
| *Fcrls* | Fc receptor-like S, scavenger receptor |
| *Fhdc1* | FH2 domain containing 1 |
| *Fkbp5* | FK506 binding protein 5 |
| *Fmo2* | flavin containing monooxygenase 2 |
| *Foxm1* | forkhead box M1 |
| *Fzr1* | fizzy/cell division cycle 20 related 1 (Drosophila) |
| *Gabrq* | γ-aminobutyric acid (GABA) A receptor, subunit θ |
| *Gata1* | GATA binding protein 1 |
| *Gbp5* | guanylate binding protein 5 |
| *Gbp7* | guanylate binding protein 7 |
| *Glrx5* | glutaredoxin 5 homolog (S. cerevisiae) |
| *Gmnn* | geminin |
| *Gp1ba* | glycoprotein 1b, α polypeptide |
| *Gp2* | glycoprotein 2 (zymogen granule membrane) |
| *Gp49a* | glycoprotein 49 A |
| *Gp5* | glycoprotein 5 (platelet) |
| *Gp9* | glycoprotein 9 (platelet) |
| *Gpnmb* | glycoprotein (transmembrane) nmb (osteoactivin) |
| *Gypa* | glycophorin A |
| *H19* | NCK-associated protein 1 (*Nckap1*) |
| *H2-Aa* | histocompatibility 2, class II antigen A, α (HLA-DQA1 in human) |
| *H2-Ab1* | histocompatibility 2, class II antigen A, β1 |
| *H2-Eb1* | histocompatibility 2, class II antigen E β |
| *H2-Q5* | histocompatibility 2, Q region locus 5 |
| *H2-T10* | histocompatibility 2, T region locus 10 |
| *Hagh* | hydroxyacyl glutathione hydrolase |
| *Hemgn* | hemogen |
| *Hk1* | hexokinase 1 |
| *Hmbs* | hydroxymethylbilane synthase |
| *Hmcn1* | hemicentin 1 |
| *Hmgcs2* | 3-hydroxy-3-methylglutaryl-Coenzyme A synthase 2 |
| *Hmmr* | hyaluronan mediated motility receptor (RHAMM) |
| *Hp* | haptoglobin |
| *Hspb1* | heat shock protein 1 |
| *Icos* | inducible T cell co-stimulator |
| *Idi1* | isopentenyl-diphosphate delta isomerase |
| *Ifi204* | interferon activated gene 204 |
| *Ifit1* | interferon-induced protein with tetratricopeptide repeats 1 |
| *Ifit3* | interferon-induced protein with tetratricopeptide repeats 3 |
| *Ifitm2* | interferon induced transmembrane protein 2 |
| *Ifitm6* | interferon induced transmembrane protein 6 |
| *Ifnar* | interferon (α and β) receptor 1 |
| *Igfbp3* | insulin-like growth factor binding protein 3 |
| *Ighv1-55* | immunoglobulin heavy variable 1-55 |
| *Igj* | immunoglobulin J chain |
| *Igkv1-110* | immunoglobulin kappa variable 1-110 |
| *Igk-v28* | immunoglobulin kappa chain variable 28 (V28) |
| *Igkv8-28* | immunoglobulin kappa variable 8-28 |
| *Igm* | immunoglobulin heavy constant mu (*Ighm*) |
| *Il1r2* | interleukin 1 receptor, type II (IL-1 receptor β chain) |
| *Il36g* | interleukin 1 family, member 9 (*Il1f9*) |
| *Il6* | interleukin 6 |
| *Inpp5d* | inositol polyphosphate-5-phosphatase D |
| *Irf7* | interferon regulatory factor 7 |
| *Irg1* | immunoresponsive gene 1 |
| *Isca1* | iron-sulfur cluster assembly 1 homolog (S. cerevisiae) |
| *Itga2b* | integrin α 2b |
| *Itgb2* | integrin β 2 |
| *Jnk* | mitogen-activated protein kinase 8 (*Mapk8*) |
| *Kel* | Kell blood group |
| *Kif11* | kinesin family member 11 |
| *Klf1* | Kruppel-like factor 1 (erythroid) |
| *Klk1* | kallikrein 1 |
| *Lbp* | lipopolysaccharide binding protein |
| *Lcn2* | lipocalin 2 |
| *Lepr* | leptin receptor |
| *Lilrb4* | leukocyte immunoglobulin-like receptor, subfamily B, member 4 |
| *Lmo2* | LIM domain only 2 |
| *Lox* | lysyl oxidase |
| *Ltf* | lactotransferrin |
| *Ly6g* | lymphocyte antigen 6 complex, locus G (*Gr1*) |
| *Ly86* | lymphocyte antigen 86 (MD1) |
| *Lyx2* | lymphocyte antigen X 2 |
| *Mad2l1* | MAD2 mitotic arrest deficient-like 1 |
| *Madcam1* | mucosal vascular addressin cell adhesion molecule 1 |
| *Mageb16* | melanoma antigen family B, 16 |
| *Map2k3* | mitogen-activated protein kinase kinase 3 |
| *Mcm2* | minichromosome maintenance deficient 2 mitotin (S. cerevisiae) |
| *Mela* | melanoma antigen |
| *Mog* | myelin oligodendrocyte glycoprotein |
| *Mpeg1* | macrophage expressed gene 1 |
| *Mpl* | myeloproliferative leukemia virus oncogene |
| *Mpp1* | membrane protein, palmitoylated |
| *Mrc1* | mannose receptor, C type 1 |
| *Ms4a3* | membrane-spanning 4-domains, subfamily A, member 3 |
| *Ms4a6c* | membrane-spanning 4-domains, subfamily A, member 6C |
| *Msn* | moesin |
| *Msr1* | macrophage scavenger receptor 1 |
| *Myb* | myeloblastosis oncogene |
| *Myl4* | myosin, light polypeptide 4 |
| *Myoc* | myocilin |
| *Ncf1* | neutrophil cytosolic factor 1 |
| *Ndrg2* | N-myc downstream regulated gene 2 |
| *Neb* | nebulin |
| *Nfe2* | nuclear factor, erythroid derived 2 |
| *Nfkb* | v-rel reticuloendotheliosis viral oncogene homolog A (avian) (*Rela*) / NF kappaB |
| *Nos2* | nitric oxide synthase 2, inducible |
| *Npas4* | neuronal PAS domain protein 4 |
| *Nr4a1* | nuclear receptor subfamily 4, group A, member 1 |
| *Nr4a3* | nuclear receptor subfamily 4, group A, member 3 |
| *Nt5c3* | 5'-nucleotidase, cytosolic III |
| *Nxpe2* | neurexophilin and PC-esterase domain family, member 2 |
| *Olfm4* | olfactomedin 4 |
| *Olr1* | oxidized low density lipoprotein (lectin-like) receptor 1 |
| *Opalin* | oligodendrocytic myelin paranodal and inner loop protein |
| *P38Mapk* | mitogen-activated protein kinase 14 (*Mapk14*) |
| *Parp12* | poly (ADP-ribose) polymerase family, member 12 |
| *Parp14* | poly (ADP-ribose) polymerase family, member 14 |
| *Pdk4* | pyruvate dehydrogenase kinase, isoenzyme 4 |
| *Peg3* | paternally expressed 3 |
| *Per1* | period circadian clock 1 |
| *Pf4* | platelet factor 4 |
| *Piga* | phosphatidylinositol glycan anchor biosynthesis, class A |
| *Pigq* | phosphatidylinositol glycan anchor biosynthesis, class Q |
| *Pkhd1l1* | polycystic kidney and hepatic disease 1-like 1 |
| *Plac8* | placenta-specific 8 |
| *Plaur* | plasminogen activator, urokinase receptor |
| *Plin4* | perilipin 4 |
| *Pnliprp2* | pancreatic lipase-related protein 2 |
| *Ppox* | protoporphyrinogen oxidase |
| *Prdx2* | peroxiredoxin 2 |
| *Prlr* | prolactin receptor |
| *Pttg1* | pituitary tumor-transforming gene 1 |
| *Rarres2* | retinoic acid receptor responder (tazarotene induced) 2 |
| *Reg1* | regenerating islet-derived 1 |
| *Reg2* | regenerating islet-derived 2 |
| *Rhag* | Rhesus blood group-associated A glycoprotein |
| *Rhd* | Rh blood group, D antigen |
| *Rsad2* | radical S-adenosyl methionine domain containing 2 |
| *Saa3* | serum amyloid A 3 |
| *Sell* | selectin, lymphocyte |
| *Selp* | selectin, platelet |
| *Serpina3c* | serine (or cysteine) peptidase inhibitor, clade A, member 3C |
| *Serpina3n* | serine (or cysteine) peptidase inhibitor, clade A, member 3N |
| *Serpinb1a* | serine (or cysteine) peptidase inhibitor, clade B, member 1a |
| *Shcbp1* | Shc SH2-domain binding protein 1 |
| *Slc25a21* | solute carrier family 25 (mitochondrial oxodicarboxylate carrier), member 21 |
| *Slc25a37* | solute carrier family 25, member 37 |
| *Slc25a39* | solute carrier family 25, member 39 |
| *Slc4a1* | solute carrier family 4 (anion exchanger), member 1 |
| *Slc6a4* | solute carrier family 6 (neurotransmitter transporter, serotonin), member 4 |
| *Slfn2* | schlafen 2 |
| *Smc2* | structural maintenance of chromosomes 2 |
| *Sphk1* | sphingosine kinase 1 |
| *Spp1* | secreted phosphoprotein 1 (osteopontin) |
| *Spta1* | spectrin α, erythrocytic 1 |
| *Sqle* | squalene epoxidase |
| *Src* | Rous sarcoma oncogene |
| *Stfa2* | stefin A2 |
| *Stfa2l1* | stefin A2 like 1 |
| *Stx2* | syntaxin 2 |
| *Tab3* | TGF-beta activated kinase 1/MAP3K7 binding protein 3 |
| *Tagln2* | transgelin 2 |
| *Tal1* | T cell acute lymphocytic leukemia 1 |
| *Tcirg1* | T cell, immune regulator 1, ATPase, H+ transporting, lysosomal V0 protein A3 |
| *Tfec* | transcription factor EC |
| *Tfrc* | transferrin receptor |
| *Tgfbr3* | transforming growth factor, beta receptor III |
| *Tgif1* | TGFB-induced factor homeobox 1 |
| *Tgtp1* | T cell specific GTPase 1 |
| *Timp1* | tissue inhibitor of metalloproteinase 1 |
| *Tlr13* | toll-like receptor 13 |
| *Tlr3* | toll-like receptor 3 |
| *Tmem173* | transmembrane protein 173 |
| *Tmem56* | transmembrane protein 56 |
| *Tpsab1* | tryptase α/β 1 |
| *Trim10* | tripartite motif-containing 10 |
| *Trip13* | thyroid hormone receptor interactor 13 |
| *Try10* | trypsin 10 |
| *Try5* | trypsin 5 |
| *Tsga13* | testis specific gene A13 |
| *Tspan8* | tetraspanin 8 |
| *Tspo2* | translocator protein 2 |
| *Tubb* | tubulin, β 4A class IVA (*Tubb4a*) |
| *Txnrd2* | thioredoxin reductase 2 |
| *Ube2l6* | ubiquitin-conjugating enzyme E2L 6 |
| *Ugt8a* | UDP galactosyltransferase 8A |
| *Unc93b1* | unc-93 homolog B1 (C. elegans) |
| *Urod* | uroporphyrinogen decarboxylase |
| *Uros* | uroporphyrinogen III synthase |
| *Vegf* | vascular endothelial growth factor A (*Vegf*) |
| *Vwf* | Von Willebrand factor homolog |
| *Wfdc17* | WAP four-disulfide core domain 17 |
| *Wrn* | Werner syndrome homolog (human) |
| *Wt1* | Wilms tumor 1 homolog |
| *Zbtb16* | zinc finger and BTB domain containing 16 |
| *Zfpm1* | zinc finger protein, multitype 1 |

**Supplemental Table 2.** List of genes in cluster 1

|  | **Log ratio** | | | |  |
| --- | --- | --- | --- | --- | --- |
| **Probe Set ID** | **A.SW Brain** | **SJL Brain** | **A.SW Spleen** | **SJL Spleen** | **Symbol** |
| 10340032 | -1.70 | 0.36 | 0.27 | -0.67 | Unidentified |
| 10342449 | -1.37 | -0.19 | -0.40 | 0.84 | Unidentified |
| 10341932 | -1.35 | -0.50 | -0.01 | -0.10 | Unidentified |
| 10344131 | -1.20 | -0.22 | 0.43 | -0.05 | Unidentified |
| 10342759 | -1.17 | 0.29 | -0.37 | 0.34 | Unidentified |
| 10344162 | -1.16 | 0.16 | -0.78 | 0.84 | Unidentified |
| 10342561 | -1.09 | -0.40 | -0.48 | 0.04 | Unidentified |
| 10338288 | -1.03 | 0.54 | 0.01 | 0.89 | Unidentified |
| 10342344 | -1.01 | -0.29 | -0.22 | 0.16 | Unidentified |
| 10342421 | -0.99 | 0.35 | -0.16 | -0.21 | Unidentified |
| 10338444 | -0.98 | 0.01 | -0.48 | 0.25 | Unidentified |
| 10338636 | -0.98 | -0.35 | -0.37 | 0.40 | Unidentified |
| 10344167 | -0.94 | -0.03 | 0.29 | -0.08 | Unidentified |
| 10342378 | -0.94 | -0.27 | -0.30 | 0.51 | Unidentified |
| 10340011 | -0.90 | 0.16 | 0.05 | 0.31 | Unidentified |
| 10342187 | -0.89 | -0.55 | -0.06 | 0.26 | Unidentified |
| 10340762 | -0.88 | 0.14 | -0.18 | -0.42 | Unidentified |
| 10343310 | -0.87 | -0.23 | -0.46 | 0.35 | Unidentified |
| 10343126 | -0.87 | -0.59 | -0.04 | -0.07 | Unidentified |
| 10511429 | -0.87 | -0.17 | 0.04 | -0.03 | Car8 |
| 10344416 | -0.86 | -0.51 | -0.09 | 0.10 | Unidentified |
| 10339122 | -0.86 | -0.27 | -0.32 | 0.01 | Unidentified |
| 10344469 | -0.86 | 0.10 | 0.36 | 0.16 | Unidentified |
| 10341798 | -0.85 | -0.22 | 0.20 | 0.05 | Unidentified |
| 10343286 | -0.85 | -0.16 | 0.62 | -0.11 | Unidentified |
| 10342319 | -0.84 | -0.47 | -0.03 | 0.09 | Unidentified |
| 10447108 | -0.84 | -0.20 | 0.07 | -0.27 | Arhgef33 |
| 10343372 | -0.83 | 0.06 | 0.09 | -0.06 | Unidentified |
| 10338241 | -0.83 | 0.10 | 0.06 | -0.05 | Unidentified |
| 10344301 | -0.82 | -0.43 | -0.33 | 0.08 | Unidentified |
| 10538519 | -0.81 | -0.19 | 0.08 | -0.06 | Ppp1r17 |
| 10426773 | -0.81 | -0.60 | -0.06 | -0.14 | Aqp6 |
| 10339788 | -0.81 | -0.24 | 0.00 | -0.22 | Unidentified |
| 10340247 | -0.80 | 0.17 | 0.18 | -0.09 | Unidentified |
| 10341500 | -0.79 | -0.14 | -0.45 | -0.04 | Unidentified |
| 10339705 | -0.79 | -0.31 | 0.45 | 0.01 | Unidentified |
| 10344007 | -0.79 | 0.32 | -0.55 | -0.15 | Unidentified |
| 10342408 | -0.79 | 0.00 | 0.52 | 0.08 | Unidentified |
| 10343300 | -0.79 | -0.44 | -0.02 | 0.21 | Unidentified |
| 10576749 | -0.75 | -0.19 | 0.01 | -0.30 | Pcp2 |
| 10344406 | -0.75 | -0.16 | 0.17 | 0.32 | Unidentified |
| 10339095 | -0.75 | 0.87 | 0.33 | 0.14 | Unidentified |
| 10362434 | -0.74 | -0.23 | -0.30 | -0.13 | Unidentified |
| 10344088 | -0.73 | 0.11 | -0.13 | -0.18 | Unidentified |
| 10370766 | -0.73 | -0.47 | 0.06 | -0.01 | Gamt |
| 10340942 | -0.73 | -0.19 | 0.34 | -0.03 | Unidentified |
| 10342874 | -0.73 | -0.18 | 0.12 | 0.33 | Unidentified |
| 10338304 | -0.70 | -0.24 | 0.05 | 0.27 | Unidentified |
| 10501876 | -0.69 | 0.57 | -0.07 | -0.09 | Unidentified |
| 10342257 | -0.69 | 0.27 | 0.51 | 0.26 | Unidentified |
| 10342286 | -0.69 | -0.01 | 0.37 | 0.47 | Unidentified |
| 10340118 | -0.69 | -0.35 | 0.15 | 0.26 | Unidentified |
| 10341945 | -0.69 | -0.31 | -0.66 | 0.23 | Unidentified |
| 10478283 | -0.68 | -0.07 | -0.42 | -0.17 | Mphosph6 |
| 10342998 | -0.68 | 0.51 | -0.25 | 0.46 | Unidentified |
| 10341957 | -0.68 | 0.47 | -0.20 | 0.57 | Unidentified |
| 10341449 | -0.67 | 0.01 | -0.29 | -0.05 | Unidentified |
| 10342615 | -0.67 | -0.34 | -0.27 | 0.35 | Unidentified |
| 10343596 | -0.66 | 0.17 | 0.41 | 0.35 | Unidentified |
| 10340540 | -0.66 | 0.21 | -0.35 | 0.42 | Unidentified |
| 10340559 | -0.65 | -0.17 | -0.47 | -0.07 | Unidentified |
| 10339742 | -0.65 | -0.40 | -0.52 | 0.18 | Unidentified |
| 10350725 | -0.65 | -0.26 | 0.09 | -0.30 | Rgs8 |
| 10522285 | -0.65 | -0.26 | -0.01 | -0.34 | Unidentified |
| 10339477 | -0.64 | -0.15 | -0.21 | 0.19 | Unidentified |
| 10339558 | -0.64 | -0.24 | 0.43 | 0.18 | Unidentified |
| 10343410 | -0.64 | -0.12 | 0.08 | 0.18 | Unidentified |
| 10342560 | -0.64 | -0.21 | 0.30 | 0.17 | Unidentified |
| 10341591 | -0.64 | -0.55 | -0.38 | 0.31 | Unidentified |
| 10341503 | -0.63 | -0.21 | -0.40 | -0.05 | Unidentified |
| 10343969 | -0.63 | 0.36 | 0.14 | 0.48 | Unidentified |
| 10340170 | -0.63 | 0.26 | 0.44 | 0.11 | Unidentified |
| 10340766 | -0.63 | 0.52 | -0.27 | 0.26 | Unidentified |
| 10343072 | -0.62 | -0.03 | 0.07 | 0.17 | Unidentified |
| 10342435 | -0.62 | -0.25 | -0.31 | 0.04 | Unidentified |
| 10514491 | -0.62 | -0.52 | -0.24 | 0.08 | Cyp2j12 |
| 10341840 | -0.61 | -0.18 | 0.49 | 0.00 | Unidentified |
| 10378216 | -0.61 | -0.13 | 0.09 | 0.40 | Atp2a3 |
| 10529895 | -0.61 | -0.44 | -0.06 | -0.05 | Qdpr |
| 10343546 | -0.61 | 0.24 | 0.33 | 0.53 | Unidentified |
| 10341719 | -0.61 | 0.20 | -0.05 | -0.24 | Unidentified |
| 10341270 | -0.61 | 0.11 | -0.03 | -0.30 | Unidentified |
| 10479221 | -0.60 | -0.35 | -0.22 | -0.13 | Gm14403 |
| 10344470 | -0.60 | -0.11 | -0.05 | 0.37 | Unidentified |
| 10338943 | -0.60 | -0.31 | 0.05 | -0.16 | Unidentified |
| 10344360 | -0.60 | -0.08 | 0.25 | 0.27 | Unidentified |
| 10339395 | -0.60 | -0.19 | 0.08 | 0.36 | Unidentified |
| 10341040 | -0.59 | -0.24 | 0.11 | -0.08 | Unidentified |
| 10338939 | -0.59 | -0.33 | 0.05 | 0.32 | Unidentified |
| 10343519 | -0.59 | -0.19 | 0.00 | 0.33 | Unidentified |
| 10344186 | -0.59 | -0.59 | -0.33 | 0.21 | Unidentified |
| 10339398 | -0.59 | -0.06 | 0.29 | -0.16 | Unidentified |
| 10343074 | -0.58 | -0.25 | 0.21 | 0.02 | Unidentified |
| 10340222 | -0.58 | -0.02 | 0.58 | -0.24 | Unidentified |
| 10341680 | -0.58 | -0.08 | -0.20 | 0.43 | Unidentified |
| 10338072 | -0.57 | -0.17 | 0.23 | 0.04 | Unidentified |
| 10343533 | -0.57 | -0.08 | -0.09 | 0.23 | Unidentified |
| 10338555 | -0.56 | -0.21 | -0.18 | -0.25 | Unidentified |
| 10475643 | -0.56 | -0.18 | 0.21 | -0.12 | Fgf7 |
| 10582146 | -0.56 | 0.04 | 0.06 | -0.26 | Kcng4 |
| 10343613 | -0.56 | -0.46 | 0.01 | 0.37 | Unidentified |
| 10341295 | -0.56 | 0.07 | -0.10 | 0.16 | Unidentified |
| 10341103 | -0.56 | -0.46 | -0.20 | 0.27 | Unidentified |
| 10340434 | -0.56 | 0.04 | -0.23 | 0.19 | Unidentified |
| 10365225 | -0.55 | -0.04 | -0.01 | 0.20 | Gm4924 |
| 10340855 | -0.55 | 0.11 | -0.35 | 0.30 | Unidentified |
| 10339493 | -0.55 | -0.16 | -0.11 | 0.30 | Unidentified |
| 10343838 | -0.55 | -0.06 | 0.33 | 0.38 | Unidentified |
| 10341064 | -0.55 | 0.27 | 0.09 | -0.16 | Unidentified |
| 10338103 | -0.55 | -0.14 | 0.34 | 0.19 | Unidentified |
| 10338508 | -0.53 | 0.20 | 0.02 | 0.32 | Unidentified |
| 10458033 | -0.53 | -0.42 | -0.38 | 0.16 | Stard4 |
| 10338181 | -0.53 | -0.06 | 0.14 | 0.33 | Unidentified |
| 10342962 | -0.53 | -0.09 | -0.08 | 0.36 | Unidentified |
| 10343482 | -0.53 | 0.14 | -0.13 | 0.33 | Unidentified |
| 10344009 | -0.53 | -0.04 | 0.22 | 0.43 | Unidentified |
| 10342082 | -0.53 | -0.37 | 0.10 | 0.37 | Unidentified |
| 10338592 | -0.53 | -0.18 | -0.49 | 0.01 | Unidentified |
| 10340670 | -0.53 | 0.06 | 0.19 | 0.39 | Unidentified |
| 10344560 | -0.52 | -0.32 | -0.14 | -0.13 | Unidentified |
| 10448186 | -0.52 | -0.01 | 0.04 | 0.03 | Zfp820 |
| 10342544 | -0.52 | -0.06 | -0.02 | 0.05 | Unidentified |
| 10466359 | -0.52 | -0.10 | -0.10 | -0.02 | Olfr1475 |
| 10339252 | -0.52 | 0.01 | 0.30 | 0.06 | Unidentified |
| 10572046 | -0.52 | 0.18 | -0.11 | 0.38 | Unidentified |
| 10341747 | -0.52 | 0.11 | 0.23 | 0.00 | Unidentified |
| 10479182 | -0.51 | -0.23 | -0.23 | 0.13 | Gm14434 |
| 10479189 | -0.51 | -0.23 | -0.23 | 0.13 | Gm14434 |
| 10490259 | -0.51 | -0.23 | -0.23 | 0.13 | Gm14305 |
| 10490262 | -0.51 | -0.23 | -0.23 | 0.13 | Gm14434 |
| 10490265 | -0.51 | -0.23 | -0.23 | 0.13 | Gm14434 |
| 10490268 | -0.51 | -0.23 | -0.23 | 0.13 | Gm14434 |
| 10490291 | -0.51 | -0.23 | -0.23 | 0.13 | Gm14434 |
| 10340513 | -0.51 | 0.12 | 0.10 | -0.01 | Unidentified |
| 10340139 | -0.51 | -0.38 | -0.10 | 0.12 | Unidentified |
| 10339792 | -0.51 | -0.10 | -0.28 | -0.06 | Unidentified |
| 10342103 | -0.51 | -0.08 | -0.05 | 0.01 | Unidentified |
| 10339555 | -0.51 | -0.25 | 0.25 | 0.06 | Unidentified |
| 10339774 | -0.51 | 0.07 | -0.16 | -0.32 | Unidentified |
| 10341772 | -0.50 | 0.46 | -0.12 | -0.06 | Unidentified |
| 10343966 | -0.50 | 0.21 | 0.47 | -0.27 | Unidentified |
| 10406226 | -0.50 | 0.05 | -0.41 | 0.10 | Unidentified |
| 10342071 | -0.50 | 0.15 | -0.45 | 0.16 | Unidentified |
| 10341059 | -0.50 | 0.14 | -0.19 | -0.21 | Unidentified |
| 10341548 | -0.49 | -0.14 | -0.13 | 0.43 | Unidentified |
| 10338874 | -0.49 | -0.22 | 0.08 | -0.16 | Unidentified |
| 10600249 | -0.49 | -0.28 | 0.05 | -0.04 | Plxnb3 |
| 10340985 | -0.49 | -0.11 | 0.13 | 0.18 | Unidentified |
| 10340296 | -0.49 | 0.20 | 0.14 | -0.10 | Unidentified |
| 10408220 | -0.49 | -0.24 | -0.14 | 0.14 | Hist1h2ac |
| 10341359 | -0.49 | -0.17 | 0.12 | 0.20 | Unidentified |
| 10342937 | -0.49 | -0.41 | -0.15 | 0.04 | Unidentified |
| 10344609 | -0.49 | -0.11 | -0.16 | -0.21 | Unidentified |
| 10343216 | -0.48 | -0.04 | 0.36 | -0.24 | Unidentified |
| 10342906 | -0.48 | -0.35 | 0.19 | 0.07 | Unidentified |
| 10340102 | -0.48 | -0.28 | -0.12 | 0.37 | Unidentified |
| 10338364 | -0.48 | 0.15 | -0.24 | 0.04 | Unidentified |
| 10374551 | -0.47 | -0.13 | -0.13 | -0.29 | Unidentified |
| 10484630 | -0.47 | -0.03 | 0.12 | 0.01 | Olfr1090 |
| 10339903 | -0.47 | 0.13 | 0.46 | -0.11 | Unidentified |
| 10342019 | -0.47 | 0.05 | 0.13 | -0.13 | Unidentified |
| 10339222 | -0.47 | -0.07 | -0.36 | -0.05 | Unidentified |
| 10343195 | -0.47 | -0.15 | -0.17 | -0.09 | Unidentified |
| 10599871 | -0.47 | 0.12 | -0.37 | -0.21 | Unidentified |
| 10342740 | -0.47 | 0.16 | 0.11 | -0.03 | Unidentified |
| 10344499 | -0.47 | 0.12 | -0.06 | -0.10 | Unidentified |
| 10340124 | -0.46 | 0.37 | -0.32 | 0.04 | Unidentified |
| 10498307 | -0.46 | 0.52 | 0.15 | 0.26 | Unidentified |
| 10338980 | -0.46 | -0.26 | 0.03 | 0.29 | Unidentified |
| 10340056 | -0.46 | 0.33 | 0.03 | -0.04 | Unidentified |
| 10339494 | -0.46 | 0.64 | -0.12 | -0.17 | Unidentified |
| 10343367 | -0.46 | -0.01 | 0.02 | -0.11 | Unidentified |
| 10490061 | -0.46 | -0.14 | -0.19 | -0.28 | Bcas1 |
| 10338100 | -0.46 | 0.07 | 0.03 | 0.03 | Unidentified |
| 10527186 | -0.46 | -0.17 | 0.05 | -0.11 | Grid2ip |
| 10343091 | -0.46 | 0.23 | 0.17 | -0.06 | Unidentified |
| 10344060 | -0.46 | -0.15 | 0.03 | 0.23 | Unidentified |
| 10344108 | -0.45 | -0.24 | -0.02 | 0.17 | Unidentified |
| 10342206 | -0.45 | -0.30 | 0.08 | 0.06 | Unidentified |
| 10338198 | -0.45 | 0.05 | 0.02 | 0.38 | Unidentified |
| 10342830 | -0.45 | -0.41 | -0.07 | 0.13 | Unidentified |
| 10359113 | -0.45 | -0.27 | -0.12 | 0.26 | Fam163a |
| 10338719 | -0.45 | -0.11 | 0.30 | -0.02 | Unidentified |
| 10342492 | -0.45 | 0.03 | 0.31 | 0.22 | Unidentified |
| 10340068 | -0.45 | -0.02 | 0.19 | -0.27 | Unidentified |
| 10344182 | -0.45 | -0.07 | 0.16 | 0.29 | Unidentified |
| 10562152 | -0.45 | -0.16 | -0.01 | -0.17 | Mag |
| 10341236 | -0.45 | -0.42 | 0.02 | 0.24 | Unidentified |
| 10342158 | -0.44 | 0.11 | -0.13 | 0.26 | Unidentified |
| 10338412 | -0.44 | 0.21 | -0.03 | 0.30 | Unidentified |
| 10338091 | -0.44 | -0.26 | 0.17 | -0.01 | Unidentified |
| 10341311 | -0.44 | -0.07 | 0.10 | 0.30 | Unidentified |
| 10545026 | -0.44 | 0.04 | 0.46 | -0.12 | Vmn1r23 |
| 10469433 | -0.43 | 0.10 | -0.01 | 0.01 | Unidentified |
| 10341138 | -0.43 | -0.10 | -0.25 | -0.05 | Unidentified |
| 10338892 | -0.43 | 0.05 | -0.30 | -0.21 | Unidentified |
| 10396148 | -0.43 | -0.18 | 0.07 | -0.09 | Abhd12b |
| 10342883 | -0.43 | -0.05 | -0.05 | 0.11 | Unidentified |
| 10465553 | -0.43 | -0.16 | -0.09 | -0.17 | Fkbp2 |
| 10341251 | -0.43 | -0.18 | 0.22 | 0.07 | Unidentified |
| 10343200 | -0.42 | 0.38 | 0.05 | 0.03 | Unidentified |
| 10344285 | -0.42 | 0.02 | 0.26 | 0.26 | Unidentified |
| 10342291 | -0.42 | 0.17 | 0.01 | 0.09 | Unidentified |
| 10538354 | -0.42 | -0.04 | 0.07 | -0.02 | Gm16499 |
| 10339863 | -0.42 | 0.03 | -0.30 | -0.10 | Unidentified |
| 10339972 | -0.42 | -0.09 | 0.23 | -0.08 | Unidentified |
| 10338079 | -0.42 | -0.03 | -0.10 | 0.25 | Unidentified |
| 10343000 | -0.42 | 0.09 | 0.18 | 0.08 | Unidentified |
| 10342579 | -0.42 | -0.10 | -0.10 | 0.44 | Unidentified |
| 10400948 | -0.42 | -0.15 | -0.07 | 0.02 | 4930447C04Rik |
| 10343331 | -0.41 | -0.12 | 0.07 | 0.40 | Unidentified |
| 10503416 | -0.41 | -0.24 | -0.06 | -0.06 | Calb1 |
| 10351525 | -0.41 | -0.18 | 0.00 | -0.15 | Mpz |
| 10342340 | -0.41 | -0.23 | -0.22 | 0.08 | Unidentified |
| 10338119 | -0.41 | -0.04 | 0.31 | -0.10 | Unidentified |
| 10401834 | -0.41 | 0.44 | -0.05 | -0.18 | Gm5039 |
| 10342614 | -0.41 | -0.12 | -0.31 | 0.14 | Unidentified |
| 10339485 | -0.40 | -0.28 | -0.15 | 0.09 | Unidentified |
| 10338747 | -0.40 | -0.10 | 0.03 | -0.13 | Unidentified |
| 10340966 | -0.40 | 0.03 | -0.11 | -0.18 | Unidentified |
| 10344396 | -0.40 | -0.39 | -0.19 | 0.29 | Unidentified |
| 10340544 | -0.40 | 0.09 | 0.06 | 0.20 | Unidentified |
| 10339149 | -0.40 | -0.02 | 0.04 | 0.21 | Unidentified |
| 10340876 | -0.40 | 0.24 | 0.34 | 0.00 | Unidentified |
| 10570752 | -0.40 | -0.09 | 0.03 | -0.04 | Defb10 |
| 10419122 | -0.39 | 0.02 | -0.01 | -0.01 | Unidentified |
| 10339801 | -0.39 | -0.12 | -0.25 | 0.11 | Unidentified |
| 10339317 | -0.39 | -0.07 | 0.04 | -0.12 | Unidentified |
| 10340436 | -0.39 | 0.19 | 0.11 | -0.08 | Unidentified |
| 10338075 | -0.39 | -0.12 | -0.30 | 0.11 | Unidentified |
| 10338738 | -0.39 | -0.12 | 0.25 | -0.05 | Unidentified |
| 10342023 | -0.39 | 0.07 | -0.09 | 0.20 | Unidentified |
| 10478936 | -0.39 | -0.17 | 0.12 | 0.25 | Unidentified |
| 10367828 | -0.39 | -0.20 | -0.01 | 0.00 | Unidentified |
| 10341903 | -0.39 | 0.01 | -0.31 | 0.24 | Unidentified |
| 10338333 | -0.39 | 0.16 | 0.18 | -0.03 | Unidentified |
| 10341099 | -0.38 | -0.03 | 0.08 | 0.13 | Unidentified |
| 10342755 | -0.38 | -0.23 | -0.06 | 0.26 | Unidentified |
| 10341180 | -0.38 | -0.01 | -0.14 | 0.02 | Unidentified |
| 10339279 | -0.38 | -0.31 | -0.09 | 0.14 | Unidentified |
| 10338921 | -0.38 | -0.03 | 0.19 | 0.32 | Unidentified |
| 10598236 | -0.38 | -0.10 | -0.10 | 0.05 | Nudt11 |
| 10341060 | -0.38 | -0.02 | 0.36 | 0.05 | Unidentified |
| 10340374 | -0.38 | 0.25 | -0.01 | 0.01 | Unidentified |
| 10424670 | -0.38 | 0.02 | 0.13 | -0.24 | Gml2 |
| 10500754 | -0.38 | -0.06 | 0.09 | 0.06 | Sycp1 |
| 10344434 | -0.38 | 0.16 | -0.25 | 0.06 | Unidentified |
| 10340291 | -0.38 | 0.04 | -0.15 | 0.19 | Unidentified |
| 10338902 | -0.37 | 0.09 | -0.22 | 0.24 | Unidentified |
| 10514699 | -0.37 | -0.18 | -0.02 | 0.00 | B020004J07Rik |
| 10343791 | -0.37 | -0.14 | -0.15 | 0.02 | Unidentified |
| 10344520 | -0.37 | 0.13 | -0.14 | 0.10 | Unidentified |
| 10559333 | -0.37 | -0.18 | 0.21 | 0.01 | Shank2 |
| 10605871 | -0.37 | -0.27 | 0.10 | 0.20 | Unidentified |
| 10419119 | -0.37 | -0.08 | -0.01 | -0.01 | Unidentified |
| 10341231 | -0.37 | -0.07 | -0.09 | -0.19 | Unidentified |
| 10342350 | -0.37 | 0.29 | 0.17 | -0.03 | Unidentified |
| 10343708 | -0.37 | 0.02 | 0.28 | -0.13 | Unidentified |
| 10503008 | -0.37 | -0.19 | 0.22 | 0.17 | Unidentified |
| 10340701 | -0.37 | 0.23 | 0.32 | -0.07 | Unidentified |
| 10485378 | -0.36 | -0.27 | -0.18 | 0.04 | Prr5l |
| 10341069 | -0.36 | -0.10 | 0.12 | -0.09 | Unidentified |
| 10484708 | -0.36 | -0.23 | -0.13 | 0.00 | Olfr1160 |
| 10342976 | -0.36 | 0.19 | 0.06 | 0.44 | Unidentified |
| 10342565 | -0.36 | -0.22 | 0.03 | 0.10 | Unidentified |
| 10339648 | -0.36 | 0.00 | 0.16 | 0.11 | Unidentified |
| 10341537 | -0.36 | 0.07 | -0.26 | 0.22 | Unidentified |
| 10487937 | -0.36 | -0.12 | -0.02 | -0.14 | Prokr2 |
| 10342869 | -0.36 | -0.18 | 0.10 | 0.27 | Unidentified |
| 10342590 | -0.35 | -0.13 | 0.01 | 0.14 | Unidentified |
| 10536324 | -0.35 | 0.06 | 0.14 | -0.09 | Asb4 |
| 10343022 | -0.35 | 0.22 | 0.20 | 0.05 | Unidentified |
| 10343326 | -0.35 | -0.03 | 0.06 | 0.17 | Unidentified |
| 10496359 | -0.35 | 0.08 | 0.16 | -0.05 | Emcn |
| 10338279 | -0.35 | 0.07 | 0.19 | 0.25 | Unidentified |
| 10484714 | -0.35 | 0.11 | 0.32 | -0.03 | Olfr1163 |
| 10341340 | -0.35 | 0.40 | 0.05 | 0.10 | Unidentified |
| 10342290 | -0.35 | -0.03 | 0.01 | -0.08 | Unidentified |
| 10339564 | -0.35 | -0.05 | -0.17 | 0.32 | Unidentified |
| 10469811 | -0.35 | -0.04 | -0.04 | -0.17 | Il1f10 |
| 10340326 | -0.35 | -0.02 | 0.03 | 0.07 | Unidentified |
| 10479456 | -0.35 | -0.15 | -0.06 | 0.14 | Unidentified |
| 10479203 | -0.34 | -0.20 | 0.05 | 0.04 | Gm14432 |
| 10490276 | -0.34 | -0.20 | 0.05 | 0.04 | Gm14432 |
| 10342008 | -0.34 | 0.12 | -0.18 | 0.02 | Unidentified |
| 10476612 | -0.34 | -0.10 | -0.01 | -0.12 | Unidentified |
| 10344477 | -0.34 | 0.07 | 0.16 | 0.05 | Unidentified |
| 10340750 | -0.34 | -0.10 | 0.14 | 0.02 | Unidentified |
| 10340893 | -0.34 | 0.09 | 0.00 | 0.10 | Unidentified |
| 10534324 | -0.33 | -0.13 | 0.04 | 0.13 | Limk1 |
| 10473551 | -0.33 | 0.09 | -0.11 | -0.02 | Olfr153 |
| 10354492 | -0.33 | -0.01 | 0.14 | 0.13 | Unidentified |
| 10341152 | -0.33 | 0.04 | -0.04 | 0.42 | Unidentified |
| 10343783 | -0.33 | 0.42 | 0.13 | 0.05 | Unidentified |
| 10338620 | -0.33 | -0.07 | 0.15 | 0.24 | Unidentified |
| 10344326 | -0.33 | -0.03 | 0.03 | 0.16 | Unidentified |
| 10344116 | -0.33 | 0.04 | 0.12 | 0.02 | Unidentified |
| 10343962 | -0.32 | -0.02 | 0.00 | -0.11 | Unidentified |
| 10590688 | -0.32 | 0.00 | -0.02 | 0.04 | Unidentified |
| 10338284 | -0.32 | 0.02 | -0.15 | 0.05 | Unidentified |
| 10445167 | -0.32 | -0.16 | -0.12 | -0.07 | Olfr127 |
| 10341783 | -0.32 | 0.09 | 0.12 | 0.28 | Unidentified |
| 10417498 | -0.32 | -0.17 | 0.09 | 0.06 | Gm11109 |
| 10463061 | -0.32 | -0.01 | 0.05 | 0.09 | Cyp2c50 |
| 10498395 | -0.32 | 0.03 | -0.24 | 0.12 | Unidentified |
| 10343767 | -0.32 | -0.11 | -0.26 | 0.17 | Unidentified |
| 10546337 | -0.32 | 0.09 | 0.11 | 0.21 | Unidentified |
| 10453743 | -0.32 | -0.19 | 0.05 | 0.02 | Unidentified |
| 10346069 | -0.32 | -0.08 | 0.18 | 0.03 | Unidentified |
| 10339683 | -0.32 | 0.06 | 0.19 | 0.17 | Unidentified |
| 10495197 | -0.32 | -0.19 | 0.10 | 0.13 | Unidentified |
| 10408541 | -0.31 | 0.12 | -0.06 | -0.05 | Unidentified |
| 10510144 | -0.31 | -0.03 | -0.11 | 0.20 | Zfp985 |
| 10339238 | -0.31 | -0.03 | 0.00 | 0.09 | Unidentified |
| 10479176 | -0.31 | -0.24 | -0.05 | 0.12 | Gm6710 |
| 10490250 | -0.31 | -0.24 | -0.05 | 0.12 | Gm6710 |
| 10437580 | -0.31 | -0.09 | 0.14 | -0.02 | Gm9851 |
| 10341463 | -0.31 | 0.12 | -0.21 | 0.17 | Unidentified |
| 10341519 | -0.31 | 0.09 | 0.25 | 0.06 | Unidentified |
| 10490294 | -0.31 | -0.26 | -0.08 | 0.10 | Gm14434 |
| 10338792 | -0.31 | 0.20 | 0.24 | 0.14 | Unidentified |
| 10566221 | -0.31 | -0.03 | -0.16 | -0.11 | Olfr611 |
| 10434878 | -0.30 | -0.07 | 0.06 | -0.03 | Unidentified |
| 10343454 | -0.30 | 0.20 | 0.09 | 0.22 | Unidentified |
| 10499935 | -0.30 | -0.06 | 0.11 | 0.01 | Lce1i |
| 10340947 | -0.30 | -0.06 | 0.20 | 0.06 | Unidentified |
| 10338396 | -0.30 | 0.01 | 0.26 | 0.09 | Unidentified |
| 10339837 | -0.30 | 0.22 | -0.17 | 0.19 | Unidentified |
| 10343179 | -0.30 | 0.03 | 0.03 | 0.01 | Unidentified |
| 10342691 | -0.30 | -0.29 | -0.06 | 0.17 | Unidentified |
| 10396055 | -0.29 | -0.12 | -0.05 | 0.06 | Wdr20rt |
| 10473180 | -0.29 | 0.01 | -0.03 | -0.09 | Ppp1r1c |
| 10419147 | -0.29 | -0.10 | 0.04 | -0.03 | Gm10375 |
| 10504640 | -0.29 | -0.04 | -0.07 | -0.05 | Unidentified |
| 10408097 | -0.29 | 0.02 | -0.14 | 0.19 | Prss16 |
| 10510241 | -0.29 | 0.03 | -0.08 | 0.02 | Zfp985 |
| 10545479 | -0.29 | 0.04 | 0.13 | -0.02 | Tmsb10 |
| 10604879 | -0.29 | -0.05 | 0.02 | -0.07 | Unidentified |
| 10338946 | -0.29 | -0.03 | -0.08 | 0.20 | Unidentified |
| 10339528 | -0.29 | -0.06 | -0.03 | 0.13 | Unidentified |
| 10339625 | -0.29 | 0.00 | 0.10 | -0.04 | Unidentified |
| 10340788 | -0.29 | 0.20 | 0.05 | 0.18 | Unidentified |
| 10551009 | -0.28 | 0.02 | 0.11 | 0.03 | Tmsb10 |
| 10340441 | -0.28 | 0.08 | -0.02 | 0.23 | Unidentified |
| 10343532 | -0.28 | -0.01 | 0.04 | 0.21 | Unidentified |
| 10404447 | -0.28 | -0.07 | 0.03 | -0.04 | Serpinb1b |
| 10340889 | -0.28 | 0.13 | -0.01 | 0.30 | Unidentified |
| 10419284 | -0.28 | 0.04 | -0.03 | -0.03 | Gm10101 |
| 10490246 | -0.28 | -0.12 | -0.07 | 0.04 | Gm14326 |
| 10338891 | -0.27 | 0.02 | 0.17 | 0.12 | Unidentified |
| 10505982 | -0.27 | -0.04 | 0.09 | 0.14 | Fggy |
| 10343064 | -0.27 | 0.02 | -0.13 | 0.04 | Unidentified |
| 10421920 | -0.27 | -0.08 | 0.02 | 0.05 | Unidentified |
| 10584378 | -0.27 | 0.00 | 0.02 | -0.01 | Olfr890 |
| 10605499 | -0.27 | -0.08 | 0.07 | 0.13 | 3426406K10Rik |
| 10343295 | -0.26 | 0.03 | -0.05 | -0.02 | Unidentified |
| 10399629 | -0.26 | 0.00 | -0.01 | -0.03 | Ywhaq |
| 10355199 | -0.26 | 0.08 | 0.03 | 0.03 | Crygb |
| 10343604 | -0.26 | 0.06 | 0.09 | 0.10 | Unidentified |
| 10601863 | -0.26 | 0.03 | -0.07 | 0.13 | Unidentified |
| 10338872 | -0.26 | 0.16 | 0.10 | 0.07 | Unidentified |
| 10426606 | -0.25 | 0.08 | -0.05 | -0.04 | 4930415O20Rik |

**Supplemental Table 3.** List of genes in cluster 2

|  | **Log ratio** | | | |  |
| --- | --- | --- | --- | --- | --- |
| **Probe Set ID** | **A.SW Brain** | **SJL Brain** | **A.SW Spleen** | **SJL Spleen** | **Symbol** |
| 10571865 | 0.68 | 0.64 | 0.14 | 2.57 | Scrg1 |
| 10435501 | 0.02 | 0.52 | -0.18 | 2.23 | Stfa1 |
| 10410931 | 0.42 | 0.45 | 0.15 | 2.23 | Vcan |
| 10608637 | 0.34 | 0.48 | 0.60 | 1.89 | Unidentified |
| 10340050 | 0.11 | -0.19 | 0.63 | 1.70 | Unidentified |
| 10401933 | -0.07 | 0.25 | 0.70 | 1.68 | Unidentified |
| 10504759 | -0.07 | 0.25 | 0.70 | 1.68 | Unidentified |
| 10449245 | -0.09 | -0.19 | 0.80 | 1.68 | Pdia2 |
| 10537561 | -0.11 | -0.15 | 0.82 | 1.67 | 1810009J06Rik |
| 10343554 | 0.36 | -0.90 | 0.38 | 1.65 | Unidentified |
| 10340445 | 0.10 | -1.04 | -0.22 | 1.64 | Unidentified |
| 10544320 | -0.01 | -0.08 | 0.86 | 1.64 | 1810009J06Rik |
| 10472538 | 0.08 | 0.24 | -0.16 | 1.63 | Dhrs9 |
| 10581990 | -0.19 | -0.22 | 0.78 | 1.62 | Unidentified |
| 10340505 | 0.08 | 0.38 | 0.25 | 1.61 | Unidentified |
| 10343945 | 0.97 | 0.22 | -0.11 | 1.57 | Unidentified |
| 10338303 | 0.75 | -0.09 | 0.18 | 1.54 | Unidentified |
| 10342010 | 0.20 | 0.13 | 0.16 | 1.51 | Unidentified |
| 10407327 | -0.07 | 0.21 | 0.05 | 1.50 | Emb |
| 10340883 | -0.29 | -0.04 | 0.47 | 1.50 | Unidentified |
| 10593671 | -0.13 | -0.21 | 0.57 | 1.49 | Dmxl2 |
| 10355984 | 0.22 | 0.07 | 0.18 | 1.48 | Serpine2 |
| 10449284 | -0.03 | -0.01 | 0.85 | 1.47 | Dusp1 |
| 10475946 | -0.02 | 0.02 | 0.88 | 1.47 | Zc3h6 |
| 10345824 | 0.12 | 0.79 | 0.19 | 1.47 | Il18rap |
| 10564805 | -0.03 | -0.19 | 0.92 | 1.46 | Pex11a |
| 10457733 | -0.07 | -0.06 | -0.18 | 1.45 | B4galt6 |
| 10431697 | -0.14 | -0.22 | -0.20 | 1.44 | Abcd2 |
| 10361381 | -0.10 | -0.24 | -0.40 | 1.43 | Syne1 |
| 10607752 | -0.03 | -0.07 | -0.19 | 1.43 | Bmx |
| 10598071 | -0.46 | 0.29 | 0.75 | 1.42 | Unidentified |
| 10341974 | -0.08 | -0.50 | -0.48 | 1.42 | Unidentified |
| 10338912 | -0.11 | 0.15 | 0.26 | 1.42 | Unidentified |
| 10569877 | 0.26 | 0.64 | -0.40 | 1.42 | Mcemp1 |
| 10520862 | 0.08 | 0.09 | 0.58 | 1.41 | Fosl2 |
| 10502224 | 0.13 | 0.12 | -0.26 | 1.41 | Sgms2 |
| 10340930 | 0.54 | -0.39 | 0.63 | 1.41 | Unidentified |
| 10343297 | 0.36 | -0.28 | 0.22 | 1.40 | Unidentified |
| 10343944 | 0.33 | 0.37 | 0.54 | 1.39 | Unidentified |
| 10368175 | -0.06 | -0.20 | 0.60 | 1.39 | Pde7b |
| 10338693 | 0.16 | -0.02 | 0.46 | 1.39 | Unidentified |
| 10339026 | 0.15 | 0.39 | -0.26 | 1.39 | Unidentified |
| 10338777 | -0.27 | -0.17 | 0.09 | 1.38 | Unidentified |
| 10343187 | -0.51 | 0.04 | 0.49 | 1.38 | Unidentified |
| 10340010 | 0.34 | 0.08 | 0.05 | 1.38 | Unidentified |
| 10342224 | 0.07 | -0.42 | 0.34 | 1.37 | Unidentified |
| 10341810 | -0.07 | 0.33 | 0.61 | 1.37 | Unidentified |
| 10342798 | 0.04 | 0.07 | 0.40 | 1.37 | Unidentified |
| 10439299 | 0.09 | -0.27 | 0.17 | 1.36 | Stfa3 |
| 10399360 | 0.38 | 0.42 | 0.67 | 1.36 | Rhob |
| 10447036 | 0.43 | 0.44 | 0.85 | 1.36 | Unidentified |
| 10340560 | 0.44 | -0.35 | -0.12 | 1.33 | Unidentified |
| 10377429 | 0.21 | -0.03 | 0.86 | 1.33 | Snord118 |
| 10399428 | 0.21 | -0.03 | 0.86 | 1.33 | Snord118 |
| 10338252 | -0.15 | 0.24 | 0.34 | 1.32 | Unidentified |
| 10339339 | 0.14 | -0.07 | 0.54 | 1.31 | Unidentified |
| 10438769 | 0.66 | 0.60 | -0.17 | 1.31 | Cldn1 |
| 10344558 | 0.19 | -0.09 | 0.38 | 1.30 | Unidentified |
| 10498935 | -0.15 | -0.23 | 0.09 | 1.30 | Gucy1b3 |
| 10340660 | -0.11 | 0.55 | 0.09 | 1.29 | Unidentified |
| 10344312 | 0.98 | -0.17 | -0.40 | 1.29 | Unidentified |
| 10344087 | 0.04 | -0.27 | 0.12 | 1.28 | Unidentified |
| 10443408 | 0.02 | 0.07 | -0.11 | 1.28 | Mapk13 |
| 10427653 | -0.16 | -0.21 | 0.40 | 1.27 | Unidentified |
| 10338554 | 0.07 | -0.12 | 0.74 | 1.27 | Unidentified |
| 10414269 | -0.07 | 0.04 | 0.84 | 1.27 | BC061237 |
| 10339289 | 0.18 | -0.24 | 0.54 | 1.27 | Unidentified |
| 10343446 | 0.19 | 0.59 | 0.16 | 1.27 | Unidentified |
| 10338840 | -0.43 | -0.41 | 0.44 | 1.26 | Unidentified |
| 10343244 | -0.57 | 0.50 | -0.26 | 1.26 | Unidentified |
| 10603051 | -0.14 | 0.00 | 0.22 | 1.26 | Ap1s2 |
| 10475910 | 0.33 | 0.33 | 0.55 | 1.25 | Unidentified |
| 10427655 | -0.13 | -0.31 | 0.25 | 1.25 | Unidentified |
| 10341018 | 0.10 | -0.28 | 0.34 | 1.24 | Unidentified |
| 10449741 | 0.15 | 0.27 | 0.85 | 1.24 | Sik1 |
| 10401968 | 0.14 | 0.18 | 0.38 | 1.23 | Galc |
| 10339091 | 0.08 | 0.69 | -0.03 | 1.22 | Unidentified |
| 10343075 | 0.15 | 0.07 | 0.79 | 1.22 | Unidentified |
| 10578324 | -0.20 | -0.24 | 0.46 | 1.22 | Mtus1 |
| 10544315 | 0.03 | 0.01 | 0.69 | 1.21 | Unidentified |
| 10339401 | -0.09 | -0.49 | 0.54 | 1.21 | Unidentified |
| 10341864 | 0.35 | 0.48 | 0.37 | 1.21 | Unidentified |
| 10343835 | 0.57 | 0.14 | 0.42 | 1.21 | Unidentified |
| 10543120 | -0.08 | -0.28 | 0.11 | 1.20 | Ica1 |
| 10338271 | -0.03 | -0.26 | 0.46 | 1.20 | Unidentified |
| 10547641 | 0.04 | -0.04 | -0.27 | 1.19 | Slc2a3 |
| 10338989 | 0.13 | -0.18 | 0.13 | 1.19 | Unidentified |
| 10339422 | 0.51 | -0.40 | -0.34 | 1.19 | Unidentified |
| 10342195 | -0.19 | -0.07 | -0.18 | 1.19 | Unidentified |
| 10340006 | 0.19 | 0.01 | 0.58 | 1.19 | Unidentified |
| 10339745 | 0.31 | -0.02 | 0.15 | 1.18 | Unidentified |
| 10487238 | 0.12 | 0.51 | 0.16 | 1.18 | Hdc |
| 10495285 | -0.10 | -0.15 | -0.33 | 1.18 | Sort1 |
| 10501229 | 0.12 | -0.01 | 0.74 | 1.18 | Gstm1 |
| 10339200 | 0.02 | -0.40 | 0.30 | 1.18 | Unidentified |
| 10338575 | 0.30 | 0.19 | 0.15 | 1.18 | Unidentified |
| 10342078 | -0.10 | 0.54 | 0.19 | 1.17 | Unidentified |
| 10544644 | 0.04 | 0.26 | -0.20 | 1.17 | Dfna5 |
| 10343409 | 0.00 | 0.30 | 0.25 | 1.17 | Unidentified |
| 10445293 | 0.05 | 0.21 | 0.85 | 1.17 | Pla2g7 |
| 10343782 | 0.02 | 0.28 | 0.15 | 1.17 | Unidentified |
| 10342481 | 0.40 | -0.05 | -0.25 | 1.17 | Unidentified |
| 10427663 | -0.06 | -0.35 | 0.10 | 1.17 | Unidentified |
| 10595840 | -0.13 | -0.23 | -0.12 | 1.17 | Pxylp1 |
| 10339044 | 0.13 | 0.33 | 0.34 | 1.16 | Unidentified |
| 10378855 | 0.06 | 0.28 | 0.65 | 1.16 | Ssh2 |
| 10340626 | 0.00 | 0.74 | 0.10 | 1.15 | Unidentified |
| 10406334 | 0.20 | 0.19 | -0.25 | 1.15 | Mctp1 |
| 10505064 | -0.05 | -0.04 | 0.43 | 1.15 | Tmem38b |
| 10476648 | -0.07 | -0.04 | -0.15 | 1.15 | Dstn |
| 10342190 | 0.00 | -0.09 | -0.05 | 1.14 | Unidentified |
| 10344273 | 0.45 | 0.54 | 0.08 | 1.14 | Unidentified |
| 10605884 | -0.04 | -0.02 | -0.20 | 1.14 | Ophn1 |
| 10341835 | 0.19 | -0.19 | -0.08 | 1.13 | Unidentified |
| 10593668 | -0.14 | -0.19 | 0.47 | 1.13 | Dmxl2 |
| 10409276 | -0.23 | 0.20 | 0.12 | 1.13 | Unidentified |
| 10388488 | 0.01 | -0.02 | 0.51 | 1.13 | Rflnb |
| 10541644 | 0.41 | 0.52 | 0.23 | 1.12 | Cd163 |
| 10343120 | -0.41 | 0.41 | 0.12 | 1.12 | Unidentified |
| 10406536 | 0.09 | 0.02 | 0.20 | 1.12 | Unidentified |
| 10342040 | -0.76 | 0.56 | 0.34 | 1.12 | Unidentified |
| 10339054 | 0.32 | -0.43 | 0.22 | 1.12 | Unidentified |
| 10341293 | -0.07 | 0.09 | -0.11 | 1.11 | Unidentified |
| 10344242 | 0.13 | 0.14 | 0.14 | 1.10 | Unidentified |
| 10338158 | -0.01 | 0.12 | 0.31 | 1.10 | Unidentified |
| 10419568 | 0.86 | -0.18 | -0.46 | 1.10 | Rnase2a |
| 10340469 | 0.12 | 0.31 | 0.31 | 1.10 | Unidentified |
| 10339882 | 0.59 | -0.07 | 0.30 | 1.10 | Unidentified |
| 10365749 | 0.03 | 0.01 | -0.21 | 1.10 | Lta4h |
| 10587023 | 0.10 | 0.31 | -0.12 | 1.10 | Rab27a |
| 10575976 | 0.17 | 0.53 | 0.46 | 1.10 | Crispld2 |
| 10556528 | 0.09 | 0.12 | 0.67 | 1.09 | Pde3b |
| 10543802 | -0.11 | -0.11 | -0.30 | 1.09 | Plxna4 |
| 10339275 | 0.03 | 0.24 | 0.41 | 1.09 | Unidentified |
| 10542140 | 0.27 | 0.12 | -0.14 | 1.09 | Klrb1f |
| 10338714 | 1.45 | -0.61 | 0.12 | 1.09 | Unidentified |
| 10344524 | -0.53 | 0.41 | 0.34 | 1.09 | Unidentified |
| 10339006 | 0.08 | 0.39 | 0.43 | 1.08 | Unidentified |
| 10379736 | 0.00 | 0.11 | -0.18 | 1.08 | Wfdc21 |
| 10339791 | 0.21 | 0.34 | 0.57 | 1.08 | Unidentified |
| 10342908 | 0.00 | -0.27 | 0.13 | 1.08 | Unidentified |
| 10342638 | -0.04 | -0.24 | 0.44 | 1.07 | Unidentified |
| 10340726 | 0.45 | 0.13 | 0.56 | 1.07 | Unidentified |
| 10343688 | -0.20 | -0.19 | 0.44 | 1.07 | Unidentified |
| 10343666 | 0.13 | 0.54 | -0.22 | 1.07 | Unidentified |
| 10361828 | 0.21 | 0.15 | 0.71 | 1.07 | Cited2 |
| 10441178 | 0.02 | 0.03 | -0.44 | 1.07 | Itgb2l |
| 10342259 | 0.13 | -0.35 | 0.40 | 1.06 | Unidentified |
| 10552604 | -0.07 | 0.02 | 0.55 | 1.06 | Klk1b24 |
| 10338667 | 0.04 | 0.02 | 0.44 | 1.06 | Unidentified |
| 10340061 | 0.09 | -0.17 | -0.07 | 1.06 | Unidentified |
| 10342528 | -0.12 | 0.08 | -0.14 | 1.06 | Unidentified |
| 10339197 | 0.25 | 0.18 | -0.09 | 1.05 | Unidentified |
| 10340775 | 0.45 | -0.22 | 0.04 | 1.05 | Unidentified |
| 10593293 | -0.08 | -0.13 | 0.43 | 1.05 | Ncam1 |
| 10338131 | -0.08 | 0.10 | -0.03 | 1.04 | Unidentified |
| 10340805 | 0.59 | -0.66 | 0.33 | 1.04 | Unidentified |
| 10542575 | -0.13 | -0.03 | -0.02 | 1.04 | Pde3a |
| 10411156 | -0.06 | -0.15 | -0.03 | 1.04 | Scamp1 |
| 10339145 | 0.11 | 0.10 | 0.45 | 1.04 | Unidentified |
| 10344252 | -0.14 | 0.25 | 0.11 | 1.03 | Unidentified |
| 10599997 | 0.16 | 0.05 | 0.28 | 1.03 | Mtmr1 |
| 10591643 | 0.21 | 0.09 | 0.30 | 1.03 | Rab3d |
| 10338083 | 0.63 | -0.06 | 0.45 | 1.03 | Unidentified |
| 10384572 | 0.07 | 0.09 | -0.13 | 1.02 | Lgalsl |
| 10342121 | -0.37 | 0.27 | -0.14 | 1.02 | Unidentified |
| 10343327 | 0.10 | 0.12 | 0.27 | 1.01 | Unidentified |
| 10340875 | -0.32 | 0.38 | 0.23 | 1.01 | Unidentified |
| 10344453 | 0.69 | 0.42 | -0.41 | 1.01 | Unidentified |
| 10415392 | 0.12 | 0.08 | -0.34 | 1.01 | Ltb4r1 |
| 10351623 | -0.02 | 0.13 | 0.33 | 1.01 | F11r |
| 10563709 | 0.14 | -0.04 | -0.27 | 1.01 | Mrgpra1 |
| 10403464 | 0.03 | -0.08 | 0.39 | 1.00 | Dip2c |
| 10538873 | 0.12 | 0.31 | -0.11 | 1.00 | Unidentified |
| 10565775 | 0.14 | 0.17 | 0.26 | 1.00 | Dgat2 |
| 10425207 | -0.12 | 0.07 | 0.21 | 0.99 | H1f0 |
| 10343852 | -0.38 | 0.60 | 0.23 | 0.99 | Unidentified |
| 10406905 | 0.03 | 0.27 | 0.34 | 0.99 | Ccdc125 |
| 10371387 | 0.06 | 0.08 | 0.01 | 0.99 | Ckap4 |
| 10344078 | 0.17 | -0.31 | -0.03 | 0.99 | Unidentified |
| 10475912 | 0.08 | 0.22 | 0.54 | 0.99 | Tmem87b |
| 10340205 | 0.27 | -0.09 | 0.14 | 0.98 | Unidentified |
| 10392259 | 0.02 | -0.16 | 0.09 | 0.98 | Unidentified |
| 10339844 | 0.57 | 0.01 | -0.08 | 0.98 | Unidentified |
| 10340817 | 0.05 | 0.28 | -0.01 | 0.98 | Unidentified |
| 10400941 | 0.10 | 0.03 | 0.48 | 0.97 | Dhrs7 |
| 10340400 | 0.23 | 0.32 | 0.21 | 0.97 | Unidentified |
| 10453759 | 0.46 | -0.10 | 0.40 | 0.97 | Unidentified |
| 10338982 | 0.63 | 0.21 | 0.09 | 0.96 | Unidentified |
| 10340739 | 0.16 | 0.18 | 0.24 | 0.96 | Unidentified |
| 10555174 | 0.13 | -0.02 | -0.09 | 0.96 | Lrrc32 |
| 10352448 | 0.04 | -0.07 | 0.37 | 0.96 | Dusp10 |
| 10338995 | 0.54 | -0.23 | 0.37 | 0.95 | Unidentified |
| 10409278 | -0.02 | 0.07 | 0.05 | 0.95 | Nfil3 |
| 10366707 | 0.09 | 0.13 | 0.39 | 0.94 | Avpr1a |
| 10384670 | 0.08 | -0.22 | 0.33 | 0.94 | Unidentified |
| 10596454 | 0.09 | -0.03 | -0.04 | 0.94 | Alas1 |
| 10443470 | 0.20 | 0.11 | -0.31 | 0.94 | Rab44 |
| 10339047 | 0.10 | -0.08 | 0.08 | 0.93 | Unidentified |
| 10472820 | 0.07 | 0.17 | -0.29 | 0.93 | Itga6 |
| 10457168 | 0.20 | 0.37 | -0.14 | 0.93 | Cd226 |
| 10343664 | 0.04 | 0.43 | 0.11 | 0.93 | Unidentified |
| 10492689 | 0.16 | 0.00 | 0.05 | 0.93 | Pdgfc |
| 10341473 | -0.08 | 0.24 | -0.05 | 0.93 | Unidentified |
| 10491599 | -0.01 | -0.14 | 0.37 | 0.92 | Unidentified |
| 10339223 | 0.11 | -0.21 | 0.36 | 0.92 | Unidentified |
| 10575993 | -0.09 | -0.08 | 0.28 | 0.92 | 6430548M08Rik |
| 10341654 | -0.10 | 0.30 | 0.14 | 0.92 | Unidentified |
| 10398859 | 0.26 | 0.30 | 0.26 | 0.92 | Adssl1 |
| 10343125 | -0.03 | 0.00 | 0.40 | 0.91 | Unidentified |
| 10453636 | 0.22 | 0.25 | 0.13 | 0.91 | Svil |
| 10341664 | 0.13 | -0.04 | 0.30 | 0.90 | Unidentified |
| 10341936 | -0.01 | 0.18 | 0.16 | 0.90 | Unidentified |
| 10341296 | 0.04 | 0.07 | 0.16 | 0.90 | Unidentified |
| 10561008 | -0.03 | 0.36 | -0.03 | 0.90 | Ceacam1 |
| 10583326 | 0.11 | 0.12 | 0.33 | 0.90 | Slc36a4 |
| 10342456 | -0.03 | 0.43 | 0.21 | 0.90 | Unidentified |
| 10338099 | -0.28 | 0.35 | 0.27 | 0.89 | Unidentified |
| 10339033 | -0.12 | 0.42 | 0.12 | 0.89 | Unidentified |
| 10342401 | 0.04 | -0.05 | 0.36 | 0.88 | Unidentified |
| 10342371 | 0.17 | -0.12 | -0.08 | 0.88 | Unidentified |
| 10341629 | -0.13 | 0.03 | 0.33 | 0.88 | Unidentified |
| 10343458 | 0.15 | 0.05 | 0.12 | 0.88 | Unidentified |
| 10582896 | -0.20 | 0.31 | 0.09 | 0.88 | Unidentified |
| 10554094 | 0.12 | 0.11 | 0.15 | 0.87 | Igf1r |
| 10340598 | 0.15 | 0.02 | 0.31 | 0.87 | Unidentified |
| 10552570 | 0.21 | -0.12 | 0.09 | 0.87 | Klk1b11 |
| 10599435 | 0.15 | 0.02 | 0.13 | 0.87 | Ocrl |
| 10343309 | 0.12 | 0.19 | 0.10 | 0.86 | Unidentified |
| 10339270 | 0.44 | 0.00 | 0.21 | 0.85 | Unidentified |
| 10341855 | 0.12 | 0.09 | 0.34 | 0.85 | Unidentified |
| 10342315 | 0.09 | 0.03 | 0.20 | 0.85 | Unidentified |
| 10343157 | 0.57 | -0.17 | 0.30 | 0.85 | Unidentified |
| 10436500 | 0.03 | -0.01 | 0.17 | 0.84 | Gbe1 |
| 10344026 | -0.05 | 0.06 | 0.21 | 0.84 | Unidentified |
| 10431637 | 0.04 | 0.06 | 0.17 | 0.84 | Cpne8 |
| 10341282 | 0.16 | 0.14 | 0.27 | 0.83 | Unidentified |
| 10342292 | 0.05 | -0.06 | 0.28 | 0.83 | Unidentified |
| 10339516 | -0.03 | 0.05 | 0.20 | 0.83 | Unidentified |
| 10365983 | 0.31 | -0.05 | 0.05 | 0.83 | Lum |
| 10341955 | 0.31 | 0.06 | 0.28 | 0.82 | Unidentified |
| 10341887 | 0.03 | 0.06 | 0.24 | 0.82 | Unidentified |
| 10342218 | 0.71 | -0.33 | 0.10 | 0.81 | Unidentified |
| 10343279 | 0.27 | -0.15 | 0.19 | 0.79 | Unidentified |
| 10338512 | 0.19 | -0.06 | 0.24 | 0.78 | Unidentified |

**Supplemental Table 4.** List of genes in cluster 3

|  | **Log ratio** | | | |  |
| --- | --- | --- | --- | --- | --- |
| **Probe Set ID** | **A.SW Brain** | **SJL Brain** | **A.SW Spleen** | **SJL Spleen** | **Symbol** |
| 10435497 | 0.38 | 1.46 | 1.36 | 4.90 | Stfa2l1 |
| 10439292 | -0.04 | 0.34 | 0.36 | 4.01 | BC100530 |
| 10539194 | 0.21 | 0.24 | 0.56 | 3.34 | Reg2 |
| 10558159 | -0.07 | -0.15 | 2.62 | 3.27 | Dmbt1 |
| 10345752 | 0.49 | 1.73 | 0.90 | 3.17 | Il1r2 |
| 10567366 | -0.14 | -0.03 | 1.95 | 3.02 | Gp2 |
| 10543017 | 0.72 | 0.93 | 3.09 | 2.83 | Pdk4 |
| 10552546 | 0.22 | -0.01 | 1.36 | 2.78 | Klk1 |
| 10544333 | -0.32 | -0.26 | 2.89 | 2.74 | Try5 |
| 10439296 | 0.28 | 0.70 | 0.85 | 2.69 | Stfa2 |
| 10539200 | -0.09 | -0.15 | 1.25 | 2.68 | Reg1 |
| 10543762 | -0.13 | 0.02 | 2.33 | 2.68 | Tsga13 |
| 10464328 | 0.00 | -0.03 | 2.47 | 2.65 | Pnliprp2 |
| 10537638 | 0.31 | 0.24 | 2.64 | 2.61 | Try10 |
| 10537014 | -0.19 | -0.06 | 2.59 | 2.51 | Cpa2 |
| 10402435 | 0.45 | 0.57 | 2.07 | 2.43 | Serpina3c |
| 10517573 | -0.04 | -0.11 | 2.40 | 2.38 | Cela3b |
| 10545561 | -0.13 | 0.00 | 0.94 | 2.28 | Reg3d |
| 10464313 | -0.03 | -0.11 | 2.83 | 2.27 | Pnliprp1 |
| 10598041 | 0.46 | 1.63 | 1.59 | 2.27 | Unidentified |
| 10432652 | -0.03 | 0.07 | 1.53 | 2.24 | Cela1 |
| 10518059 | 0.05 | -0.03 | 1.54 | 2.23 | Ctrc |
| 10538890 | 0.13 | 0.50 | 1.42 | 2.21 | LOC641050 |
| 10568165 | 0.01 | -0.09 | 1.77 | 2.18 | Zg16 |
| 10458704 | -0.17 | -0.01 | 1.72 | 2.12 | Spink1 |
| 10506301 | 0.34 | 0.00 | 1.96 | 2.11 | Lepr |
| 10512279 | 0.05 | -0.04 | 1.58 | 2.09 | Cntfr |
| 10510574 | 0.34 | 0.37 | 1.94 | 2.08 | Errfi1 |
| 10524698 | -0.10 | 0.08 | 1.42 | 2.08 | Pla2g1b |
| 10369290 | 0.59 | 1.08 | 1.75 | 2.07 | Ddit4 |
| 10376201 | 0.53 | 0.64 | 1.52 | 2.07 | Gpx3 |
| 10449452 | 1.03 | 1.54 | 1.69 | 2.02 | Fkbp5 |
| 10414192 | -0.06 | 0.02 | 1.00 | 2.01 | Mat1a |
| 10593225 | 0.67 | 0.94 | 1.62 | 2.00 | Zbtb16 |
| 10552618 | -0.13 | -0.19 | 1.10 | 1.97 | Klk1b5 |
| 10359582 | 0.63 | -0.10 | 1.61 | 1.92 | Fmo2 |
| 10518050 | -0.06 | -0.01 | 1.92 | 1.92 | Cela2a |
| 10602896 | -0.05 | -0.02 | 1.22 | 1.91 | Adgrg2 |
| 10401931 | 0.09 | 0.36 | 1.37 | 1.91 | Unidentified |
| 10401937 | 0.09 | 0.36 | 1.37 | 1.91 | BC005685 |
| 10481278 | -0.05 | 0.06 | 2.11 | 1.90 | Cel |
| 10419578 | 0.06 | 0.04 | 2.01 | 1.90 | Ndrg2 |
| 10504753 | 0.19 | 0.23 | 1.25 | 1.88 | LOC641050 |
| 10464298 | -0.06 | -0.10 | 2.43 | 1.88 | Pnlip |
| 10568502 | 0.05 | 0.17 | 1.63 | 1.88 | Cuzd1 |
| 10497463 | 0.11 | 0.19 | 2.49 | 1.86 | Cpb1 |
| 10581355 | 0.07 | -0.22 | 1.83 | 1.86 | Ctrl |
| 10606989 | 0.22 | 0.76 | 1.40 | 1.85 | Tsc22d3 |
| 10373740 | 0.24 | 0.48 | 1.54 | 1.84 | Pik3ip1 |
| 10538892 | 0.06 | 0.29 | 1.22 | 1.84 | LOC641050 |
| 10427436 | 0.23 | 0.08 | 1.57 | 1.83 | C7 |
| 10537645 | -0.08 | 0.09 | 1.41 | 1.83 | Gm5409 |
| 10538901 | -0.01 | 0.14 | 0.98 | 1.82 | BC005685 |
| 10504761 | 0.14 | 0.20 | 1.24 | 1.81 | LOC641050 |
| 10401935 | -0.03 | 0.15 | 0.89 | 1.80 | BC005685 |
| 10482528 | -0.14 | -0.26 | 1.76 | 1.76 | Neb |
| 10552587 | 0.23 | 0.04 | 1.11 | 1.76 | Klk1b21 |
| 10504757 | -0.01 | 0.21 | 0.97 | 1.75 | BC005685 |
| 10522503 | 0.26 | 0.30 | 1.56 | 1.75 | Pdgfra |
| 10392522 | -0.40 | -0.71 | 1.96 | 1.73 | Abca8a |
| 10447461 | 0.21 | 0.46 | 0.91 | 1.72 | Ston1 |
| 10578136 | 0.15 | -0.24 | 1.42 | 1.72 | Unidentified |
| 10462091 | 0.29 | 0.44 | 1.32 | 1.68 | Klf9 |
| 10537627 | 0.00 | -0.10 | 2.11 | 1.63 | Prss2 |
| 10544326 | -0.01 | 0.02 | 2.50 | 1.62 | 2210010C04Rik |
| 10501555 | 0.24 | 0.16 | 1.25 | 1.60 | Amy1 |
| 10377439 | 0.44 | 0.37 | 1.20 | 1.56 | Per1 |
| 10537634 | -0.03 | -0.21 | 1.71 | 1.55 | Try4 |
| 10345065 | 0.24 | -0.14 | 1.43 | 1.55 | Gsta3 |
| 10341419 | 0.35 | -0.69 | 1.13 | 1.53 | Unidentified |
| 10430851 | 0.63 | 0.43 | 1.34 | 1.52 | Cyp2d22 |
| 10419563 | 0.11 | -0.22 | 1.83 | 1.49 | Rnase1 |
| 10498741 | 0.02 | -0.10 | 1.21 | 1.49 | Serpini2 |
| 10494978 | 0.13 | 0.39 | 1.12 | 1.47 | Ptpn22 |
| 10449467 | 0.05 | -0.16 | 1.99 | 1.46 | Clps |
| 10537051 | 0.01 | 0.07 | 2.26 | 1.44 | Cpa1 |
| 10559796 | -0.01 | -0.09 | 1.69 | 1.43 | Peg3 |
| 10504755 | 0.29 | 0.46 | 1.21 | 1.42 | BC057193 |
| 10551531 | -0.02 | -0.02 | 2.18 | 1.39 | Sycn |
| 10449661 | 0.00 | -0.02 | 1.35 | 1.39 | Tff2 |
| 10509063 | -0.04 | -0.24 | 1.29 | 1.38 | Il22ra1 |
| 10501494 | 0.33 | -0.04 | 2.63 | 1.38 | Amy2a5 |
| 10517568 | 0.10 | -0.04 | 1.47 | 1.38 | Cela3a |
| 10483353 | 0.12 | 0.20 | 1.23 | 1.36 | Scn7a |
| 10581882 | -0.03 | 0.08 | 2.55 | 1.35 | Ctrb1 |
| 10567995 | 0.48 | 0.86 | 1.61 | 1.34 | Nupr1 |
| 10505779 | 0.42 | 0.91 | 1.25 | 1.31 | Acer2 |
| 10604175 | 0.00 | -0.18 | 1.33 | 1.30 | Tmem255a |
| 10362201 | 0.83 | 0.88 | 1.49 | 1.24 | Ctgf |
| 10537650 | -0.15 | 0.08 | 1.84 | 1.23 | Prss1 |
| 10501544 | 0.19 | -0.02 | 2.64 | 1.21 | Unidentified |
| 10339265 | 0.70 | -0.19 | 1.73 | 1.18 | Unidentified |
| 10501500 | 0.19 | -0.01 | 2.61 | 1.17 | Amy2a5 |
| 10501511 | 0.19 | -0.01 | 2.61 | 1.17 | Amy2a5 |
| 10501522 | 0.19 | -0.01 | 2.61 | 1.17 | Amy2a5 |
| 10501533 | 0.19 | -0.01 | 2.61 | 1.17 | Amy2a5 |
| 10470392 | 0.10 | 0.34 | 1.25 | 1.12 | Adamtsl2 |
| 10439009 | 0.92 | 1.16 | 2.01 | 1.01 | Apod |
| 10359571 | 0.14 | -0.04 | 1.52 | 0.99 | Fmo1 |
| 10378857 | -0.02 | -0.02 | 1.66 | 0.96 | Coro6 |
| 10459225 | 0.23 | 0.20 | 1.51 | 0.90 | mir-145 |
| 10504203 | 0.19 | 0.68 | 2.26 | 0.71 | 4930578G10Rik |

**Supplemental Table 5.** List of genes in cluster 5

|  | **Log ratio** | | | |  |
| --- | --- | --- | --- | --- | --- |
| **Probe Set ID** | **A.SW_Brain** | **SJL_Brain** | **A.SW_Spleen** | **SJL_Spleen** | **Symbol** |
| 10342609 | 1.52 | 0.20 | -0.55 | 0.18 | Unidentified |
| 10545237 | 1.50 | 0.16 | 0.06 | -0.42 | Unidentified |
| 10545239 | 1.48 | 0.18 | 0.19 | -0.30 | Unidentified |
| 10545220 | 1.45 | 0.24 | 0.77 | 0.12 | Igk |
| 10338497 | 1.32 | 0.18 | 0.84 | -0.26 | Unidentified |
| 10415438 | 1.23 | 0.23 | -0.49 | -0.40 | Mcpt2 |
| 10340796 | 1.22 | 0.45 | -0.10 | 0.46 | Unidentified |
| 10341453 | 1.20 | -0.20 | 0.24 | -0.73 | Unidentified |
| 10341175 | 1.16 | 0.30 | 0.25 | 0.27 | Unidentified |
| 10340196 | 1.12 | -0.53 | 0.10 | 0.20 | Unidentified |
| 10341181 | 1.11 | 0.26 | -0.33 | 0.32 | Unidentified |
| 10344101 | 1.09 | -0.02 | -0.19 | -0.41 | Unidentified |
| 10338735 | 1.08 | 0.12 | -0.88 | 0.01 | Unidentified |
| 10338950 | 1.04 | 0.32 | 0.00 | -0.24 | Unidentified |
| 10339143 | 1.02 | -0.37 | -0.11 | 0.34 | Unidentified |
| 10343479 | 0.99 | -0.09 | -0.01 | 0.61 | Unidentified |
| 10341594 | 0.99 | -0.58 | 0.17 | -0.12 | Unidentified |
| 10341291 | 0.93 | 0.24 | -0.34 | -0.28 | Unidentified |
| 10343337 | 0.92 | -0.32 | 0.02 | 0.20 | Unidentified |
| 10344292 | 0.90 | -0.20 | 0.12 | 0.56 | Unidentified |
| 10341541 | 0.87 | 0.02 | -0.54 | 0.15 | Unidentified |
| 10338685 | 0.86 | 0.12 | -0.27 | 0.47 | Unidentified |
| 10344501 | 0.81 | 0.19 | -0.14 | -0.35 | Unidentified |
| 10545242 | 0.81 | 0.25 | 0.53 | -0.01 | Igkv6-20 |
| 10342879 | 0.80 | -0.07 | 0.06 | 0.04 | Unidentified |
| 10379530 | 0.80 | 0.38 | -0.13 | -0.13 | Ccl12 |
| 10343833 | 0.78 | 0.22 | -0.43 | 0.30 | Unidentified |
| 10340835 | 0.77 | 0.03 | 0.59 | 0.01 | Unidentified |
| 10340595 | 0.77 | 0.06 | -0.06 | -0.54 | Unidentified |
| 10342511 | 0.74 | -0.39 | 0.06 | 0.29 | Unidentified |
| 10435043 | 0.73 | 0.43 | -0.45 | 0.47 | Tm4sf19 |
| 10341522 | 0.72 | 0.44 | -0.15 | 0.14 | Unidentified |
| 10349661 | 0.71 | 0.04 | -0.18 | 0.27 | Rab7b |
| 10339478 | 0.70 | 0.06 | 0.17 | 0.49 | Unidentified |
| 10339554 | 0.70 | -0.22 | 0.42 | 0.37 | Unidentified |
| 10340841 | 0.70 | -0.34 | -0.23 | 0.03 | Unidentified |
| 10539143 | 0.69 | 0.43 | -0.09 | -0.10 | Retsat |
| 10343493 | 0.69 | 0.00 | 0.07 | 0.19 | Unidentified |
| 10560608 | 0.69 | 0.22 | 0.53 | -0.07 | Apoc2 |
| 10341542 | 0.69 | -0.01 | 0.05 | 0.44 | Unidentified |
| 10560799 | 0.69 | -0.11 | 0.18 | -0.16 | Vmn1r180 |
| 10344263 | 0.69 | -0.35 | 0.55 | 0.27 | Unidentified |
| 10515181 | 0.69 | 0.00 | -0.45 | 0.21 | Unidentified |
| 10492396 | 0.68 | -0.04 | 0.56 | 0.16 | Vmn2r1 |
| 10593191 | 0.67 | 0.08 | -0.15 | -0.17 | Unidentified |
| 10341539 | 0.66 | -0.09 | -0.26 | -0.08 | Unidentified |
| 10343990 | 0.66 | -0.14 | -0.04 | 0.31 | Unidentified |
| 10343177 | 0.66 | -0.15 | -0.06 | 0.11 | Unidentified |
| 10483546 | 0.66 | 0.21 | -0.20 | 0.27 | Unidentified |
| 10434773 | 0.65 | -0.14 | -0.03 | -0.28 | Rtp1 |
| 10497451 | 0.65 | -0.04 | 0.32 | 0.44 | Cpa3 |
| 10341323 | 0.63 | 0.06 | -0.26 | 0.40 | Unidentified |
| 10405058 | 0.63 | 0.34 | 0.01 | 0.14 | Omd |
| 10492021 | 0.63 | 0.06 | -0.38 | -0.12 | Postn |
| 10340950 | 0.63 | 0.34 | 0.06 | 0.30 | Unidentified |
| 10341245 | 0.63 | 0.24 | -0.12 | -0.11 | Unidentified |
| 10409567 | 0.63 | 0.29 | -0.04 | -0.04 | Tifab |
| 10527487 | 0.61 | 0.08 | 0.16 | 0.17 | Cyp3a57 |
| 10340127 | 0.61 | -0.33 | 0.03 | -0.33 | Unidentified |
| 10342570 | 0.61 | 0.03 | 0.27 | -0.45 | Unidentified |
| 10341863 | 0.61 | 0.00 | 0.33 | -0.34 | Unidentified |
| 10351477 | 0.60 | 0.10 | -0.13 | -0.46 | Sh2d1b1 |
| 10343040 | 0.59 | -0.26 | -0.23 | -0.23 | Unidentified |
| 10344515 | 0.59 | 0.11 | -0.10 | -0.29 | Unidentified |
| 10341403 | 0.59 | -0.04 | 0.18 | 0.17 | Unidentified |
| 10342113 | 0.59 | 0.02 | 0.29 | 0.38 | Unidentified |
| 10349295 | 0.59 | 0.37 | 0.16 | -0.11 | Tfcp2l1 |
| 10502801 | 0.59 | 0.05 | -0.01 | -0.17 | Ifi44l |
| 10338070 | 0.58 | -0.06 | 0.23 | 0.02 | Unidentified |
| 10583421 | 0.57 | 0.16 | -0.05 | -0.05 | Unidentified |
| 10340134 | 0.57 | -0.32 | 0.30 | 0.51 | Unidentified |
| 10488553 | 0.56 | -0.19 | 0.13 | -0.18 | 4921509C19Rik |
| 10349947 | 0.56 | -0.19 | 0.34 | -0.02 | Fmod |
| 10339535 | 0.56 | -0.28 | 0.20 | -0.04 | Unidentified |
| 10460010 | 0.56 | -0.01 | 0.17 | -0.13 | Galr1 |
| 10340517 | 0.56 | -0.14 | -0.21 | 0.08 | Unidentified |
| 10341438 | 0.55 | -0.24 | -0.06 | 0.33 | Unidentified |
| 10392834 | 0.54 | 0.23 | -0.15 | -0.22 | Cd300ld5 |
| 10603059 | 0.54 | 0.41 | -0.06 | -0.03 | Tmem27 |
| 10339561 | 0.54 | -0.03 | 0.20 | 0.09 | Unidentified |
| 10397835 | 0.40 | 0.36 | -0.02 | -0.06 | Slc24a4 |
| 10545079 | 0.40 | -0.02 | 0.05 | -0.13 | A530053G22Rik |
| 10341041 | 0.40 | 0.29 | -0.29 | 0.19 | Unidentified |
| 10351465 | 0.40 | 0.22 | -0.02 | -0.03 | Ccdc190 |
| 10342692 | 0.40 | 0.22 | 0.24 | -0.18 | Unidentified |
| 10441774 | 0.40 | -0.23 | 0.13 | -0.14 | Slc22a2 |
| 10384685 | 0.40 | -0.02 | 0.12 | 0.09 | 1700093K21Rik |
| 10341393 | 0.39 | -0.17 | -0.31 | -0.03 | Unidentified |
| 10484520 | 0.39 | 0.09 | -0.14 | -0.37 | 4833423E24Rik |
| 10504417 | 0.39 | 0.28 | 0.23 | -0.03 | Hrct1 |
| 10504582 | 0.39 | 0.20 | 0.25 | -0.01 | Stra6l |
| 10343208 | 0.39 | 0.42 | -0.25 | 0.22 | Unidentified |
| 10498383 | 0.39 | 0.27 | 0.09 | -0.23 | Unidentified |
| 10344487 | 0.39 | -0.18 | 0.13 | -0.10 | Unidentified |
| 10341071 | 0.39 | 0.10 | 0.07 | 0.21 | Unidentified |
| 10342168 | 0.39 | -0.13 | 0.13 | 0.25 | Unidentified |
| 10485773 | 0.38 | -0.18 | 0.08 | 0.22 | Olfr1290 |
| 10440677 | 0.38 | -0.23 | -0.14 | -0.08 | Krtap19-1 |
| 10342326 | 0.38 | 0.00 | 0.21 | 0.05 | Unidentified |
| 10342194 | 0.38 | -0.18 | 0.36 | -0.05 | Unidentified |
| 10538377 | 0.38 | 0.26 | 0.07 | 0.04 | Wipf3 |
| 10361065 | 0.38 | 0.04 | -0.11 | 0.30 | Mfsd7b |
| 10495621 | 0.38 | -0.14 | -0.03 | 0.13 | mir-137 |
| 10341728 | 0.38 | 0.10 | -0.25 | -0.06 | Unidentified |
| 10435383 | 0.38 | 0.40 | -0.24 | 0.15 | Hacd2 |
| 10343219 | 0.38 | 0.10 | 0.00 | 0.03 | Unidentified |
| 10342955 | 0.37 | -0.09 | 0.13 | 0.12 | Unidentified |
| 10576051 | 0.37 | -0.13 | -0.03 | -0.15 | Foxc2 |
| 10537684 | 0.37 | -0.03 | -0.04 | 0.14 | Sval2 |
| 10344119 | 0.37 | 0.31 | -0.11 | 0.12 | Unidentified |
| 10548677 | 0.37 | 0.06 | -0.03 | -0.36 | Tas2r140 |
| 10429387 | 0.37 | 0.27 | -0.04 | 0.20 | Hacd2 |
| 10340564 | 0.37 | -0.18 | 0.12 | -0.21 | Unidentified |
| 10571726 | 0.37 | -0.13 | 0.11 | -0.13 | Unidentified |
| 10342567 | 0.37 | -0.17 | -0.03 | 0.15 | Unidentified |
| 10601867 | 0.37 | 0.11 | -0.15 | -0.18 | Kir3dl1 |
| 10442112 | 0.37 | -0.06 | -0.08 | -0.09 | Vmn2r93 |
| 10340053 | 0.37 | -0.03 | 0.20 | -0.28 | Unidentified |
| 10401948 | 0.36 | -0.24 | -0.26 | 0.01 | Unidentified |
| 10472514 | 0.36 | 0.14 | 0.15 | 0.00 | Nostrin |
| 10438738 | 0.36 | 0.28 | 0.02 | -0.32 | Bcl6 |
| 10339357 | 0.36 | 0.29 | -0.26 | -0.07 | Unidentified |
| 10428940 | 0.36 | 0.06 | -0.37 | -0.03 | 4930444G20Rik |
| 10341585 | 0.36 | 0.13 | 0.11 | 0.09 | Unidentified |
| 10466735 | 0.36 | 0.16 | 0.26 | -0.08 | Fam189a2 |
| 10340519 | 0.36 | 0.04 | 0.01 | -0.10 | Unidentified |
| 10598203 | 0.36 | -0.03 | 0.18 | -0.02 | Ccl28 |
| 10342271 | 0.36 | 0.13 | 0.13 | -0.02 | Unidentified |
| 10582080 | 0.36 | -0.09 | 0.04 | -0.18 | Unidentified |
| 10607465 | 0.36 | 0.18 | 0.02 | 0.08 | Unidentified |
| 10565837 | 0.35 | 0.01 | 0.09 | 0.09 | F730035P03Rik |
| 10342341 | 0.35 | -0.12 | 0.17 | 0.20 | Unidentified |
| 10340919 | 0.35 | -0.25 | -0.16 | -0.03 | Unidentified |
| 10339309 | 0.35 | 0.09 | 0.02 | -0.07 | Unidentified |
| 10344384 | 0.35 | 0.18 | 0.13 | 0.03 | Unidentified |
| 10341717 | 0.35 | 0.15 | 0.08 | 0.01 | Unidentified |
| 10445071 | 0.35 | -0.03 | 0.07 | 0.05 | Zfp57 |
| 10465840 | 0.35 | -0.18 | 0.23 | -0.04 | Scgb1a1 |
| 10339662 | 0.35 | -0.26 | 0.05 | -0.16 | Unidentified |
| 10439980 | 0.35 | -0.01 | 0.16 | -0.04 | Pcnp |
| 10339688 | 0.35 | 0.02 | 0.16 | -0.12 | Unidentified |
| 10388086 | 0.35 | -0.02 | 0.01 | 0.12 | Nlrp1c |
| 10343683 | 0.35 | 0.08 | 0.03 | -0.04 | Unidentified |
| 10584280 | 0.35 | 0.26 | -0.06 | -0.07 | Hepacam |
| 10572525 | 0.34 | -0.18 | 0.04 | -0.10 | Gm10654 |
| 10344076 | 0.34 | -0.12 | 0.25 | -0.10 | Unidentified |
| 10338517 | 0.34 | 0.14 | -0.12 | 0.19 | Unidentified |
| 10339911 | 0.34 | -0.21 | 0.11 | 0.05 | Unidentified |
| 10419154 | 0.34 | 0.02 | 0.05 | -0.04 | Ear1 |
| 10514317 | 0.34 | 0.06 | 0.03 | -0.16 | Ifnab |
| 10340003 | 0.34 | 0.26 | -0.15 | -0.27 | Unidentified |
| 10416950 | 0.34 | -0.05 | 0.14 | 0.24 | mir-17 |
| 10414262 | 0.34 | -0.25 | 0.24 | -0.05 | Ear2 |
| 10436945 | 0.34 | -0.18 | 0.08 | -0.21 | Slc5a3 |
| 10549873 | 0.34 | 0.26 | 0.10 | -0.09 | Unidentified |
| 10541333 | 0.34 | 0.36 | 0.00 | -0.10 | Slc6a12 |
| 10341711 | 0.34 | -0.04 | -0.09 | -0.15 | Unidentified |
| 10338672 | 0.34 | 0.36 | -0.01 | -0.15 | Unidentified |
| 10530615 | 0.34 | 0.26 | -0.11 | 0.05 | Ociad2 |
| 10405033 | 0.34 | 0.12 | 0.12 | 0.27 | Ecm2 |
| 10343531 | 0.29 | 0.18 | 0.15 | 0.04 | Unidentified |
| 10366528 | 0.29 | 0.04 | 0.05 | -0.16 | Best3 |
| 10342670 | 0.29 | -0.09 | -0.01 | 0.20 | Unidentified |
| 10578743 | 0.29 | -0.11 | 0.00 | -0.06 | Adam29 |
| 10340774 | 0.29 | -0.21 | 0.20 | 0.04 | Unidentified |
| 10431962 | 0.29 | 0.19 | -0.01 | -0.08 | Endou |
| 10459530 | 0.29 | -0.01 | 0.02 | -0.02 | B430212C06Rik |
| 10340831 | 0.29 | 0.00 | -0.12 | 0.15 | Unidentified |
| 10342309 | 0.29 | 0.09 | 0.00 | 0.18 | Unidentified |
| 10546417 | 0.29 | 0.12 | -0.04 | -0.10 | Trh |
| 10340827 | 0.29 | 0.09 | -0.15 | -0.04 | Unidentified |
| 10343240 | 0.28 | -0.14 | 0.12 | 0.10 | Unidentified |
| 10341445 | 0.28 | -0.13 | 0.12 | 0.09 | Unidentified |
| 10347772 | 0.28 | 0.01 | -0.13 | -0.16 | Gm10555 |
| 10563602 | 0.28 | 0.11 | 0.03 | -0.07 | Saa4 |
| 10490378 | 0.28 | 0.08 | 0.00 | -0.14 | Hrh3 |
| 10342904 | 0.28 | 0.10 | -0.10 | -0.08 | Unidentified |
| 10339259 | 0.28 | -0.05 | -0.18 | 0.01 | Unidentified |
| 10340351 | 0.28 | -0.04 | -0.11 | -0.19 | Unidentified |
| 10339650 | 0.28 | -0.10 | -0.31 | 0.09 | Unidentified |
| 10550738 | 0.27 | 0.16 | -0.15 | -0.10 | Rpl7a-ps8 |
| 10403041 | 0.27 | -0.08 | 0.14 | -0.03 | Unidentified |
| 10387194 | 0.27 | -0.03 | 0.05 | -0.01 | Odf4 |
| 10343123 | 0.27 | -0.16 | 0.17 | 0.12 | Unidentified |
| 10367582 | 0.27 | 0.23 | 0.04 | -0.11 | Vip |
| 10491197 | 0.27 | -0.10 | 0.13 | -0.04 | Gm1527 |
| 10498508 | 0.27 | -0.04 | 0.02 | -0.08 | Vmn2r-ps11 |
| 10360139 | 0.27 | 0.17 | 0.05 | -0.10 | Klhdc9 |
| 10563933 | 0.27 | 0.11 | 0.04 | -0.05 | Unidentified |
| 10571111 | 0.27 | 0.06 | 0.16 | 0.06 | Zfp703 |
| 10608100 | 0.27 | -0.14 | -0.01 | -0.08 | Zfy1 |
| 10497345 | 0.27 | 0.11 | 0.00 | 0.12 | Gm9733 |
| 10484573 | 0.27 | -0.15 | -0.03 | -0.14 | Olfr1047 |
| 10507574 | 0.27 | 0.06 | 0.00 | -0.12 | Olfr1339 |
| 10600482 | 0.27 | -0.22 | 0.07 | 0.10 | Unidentified |
| 10542267 | 0.27 | 0.06 | 0.12 | 0.01 | 5530400C23Rik |
| 10550409 | 0.26 | -0.01 | -0.01 | -0.04 | Psg16 |
| 10516037 | 0.26 | 0.09 | -0.14 | -0.17 | Gm10573 |
| 10554487 | 0.26 | 0.13 | 0.14 | 0.05 | Alpk3 |
| 10387855 | 0.26 | 0.07 | 0.10 | 0.06 | Alox15 |
| 10568496 | 0.26 | -0.07 | 0.12 | -0.08 | 4933402N03Rik |
| 10340516 | 0.26 | -0.03 | -0.03 | 0.15 | Unidentified |
| 10608689 | 0.26 | 0.05 | -0.07 | -0.08 | Unidentified |
| 10566676 | 0.26 | -0.12 | -0.08 | -0.20 | Olfr484 |
| 10546047 | 0.26 | 0.02 | 0.16 | -0.02 | 1810020O05Rik |
| 10537123 | 0.26 | -0.07 | -0.03 | -0.16 | Lrguk |
| 10440685 | 0.26 | -0.10 | 0.14 | -0.10 | Krtap19-3 |
| 10338086 | 0.26 | -0.09 | 0.04 | 0.21 | Unidentified |
| 10455591 | 0.26 | 0.11 | 0.04 | -0.05 | Unidentified |
| 10414477 | 0.26 | 0.24 | -0.05 | -0.04 | Olfr739 |
| 10380059 | 0.26 | -0.05 | 0.15 | 0.11 | Rnu3b1 |
| 10380061 | 0.26 | -0.05 | 0.15 | 0.11 | Rnu3b1 |
| 10380063 | 0.26 | -0.05 | 0.15 | 0.11 | Rnu3b1 |
| 10380065 | 0.26 | -0.05 | 0.15 | 0.11 | Rnu3b1 |
| 10439837 | 0.26 | -0.23 | -0.02 | 0.05 | Unidentified |
| 10339306 | 0.26 | -0.16 | 0.05 | -0.04 | Unidentified |
| 10346141 | 0.26 | -0.04 | 0.06 | -0.06 | C230029F24Rik |
| 10530854 | 0.26 | 0.09 | 0.12 | 0.06 | Tecrl |
| 10484675 | 0.26 | -0.19 | 0.01 | -0.06 | Olfr1128 |
| 10412207 | 0.26 | 0.12 | -0.19 | -0.11 | Gpx8 |
| 10549592 | 0.26 | -0.10 | 0.04 | 0.02 | Unidentified |
| 10343150 | 0.25 | -0.07 | -0.10 | 0.14 | Unidentified |
| 10584244 | 0.25 | -0.19 | 0.07 | -0.02 | Gm3867 |
| 10341254 | 0.25 | -0.01 | -0.10 | -0.11 | Unidentified |
| 10338688 | 0.25 | -0.10 | -0.03 | -0.04 | Unidentified |
| 10339872 | 0.25 | 0.13 | 0.11 | 0.08 | Unidentified |
| 10487366 | 0.25 | 0.03 | 0.10 | 0.04 | 1810024B03Rik |
| 10341560 | 0.24 | -0.12 | 0.09 | 0.04 | Unidentified |
| 10484723 | 0.24 | -0.10 | 0.01 | 0.03 | Olfr1167 |
| 10382852 | 0.24 | 0.02 | 0.05 | 0.08 | Mfsd11 |
| 10566672 | 0.24 | -0.09 | 0.04 | -0.06 | Olfr480 |
| 10528922 | 0.24 | -0.08 | -0.13 | -0.05 | Unidentified |
| 10484685 | 0.24 | -0.08 | -0.12 | -0.08 | Olfr1135 |
| 10571747 | 0.24 | -0.04 | 0.02 | -0.06 | Unidentified |
| 10493812 | 0.23 | 0.06 | -0.12 | -0.09 | S100a4 |
| 10412828 | 0.23 | -0.09 | 0.04 | 0.05 | Unidentified |
| 10449000 | 0.23 | 0.01 | -0.16 | -0.09 | Msln |
| 10553786 | 0.23 | 0.05 | -0.06 | -0.06 | Unidentified |
| 10608679 | 0.23 | -0.11 | 0.07 | -0.02 | Unidentified |
| 10461934 | 0.23 | -0.11 | 0.05 | -0.02 | Trpm6 |

**Supplemental Table 6.** List of genes in cluster 8

|  | **Log ratio** | | | |  |
| --- | --- | --- | --- | --- | --- |
| **Probe Set ID** | **A.SW_Brain** | **SJL_Brain** | **A.SW_Spleen** | **SJL_Spleen** | **Symbol** |
| 10531407 | 0.18 | 3.36 | -0.81 | -1.01 | Cxcl9 |
| 10531994 | 0.27 | 2.75 | 0.12 | -0.31 | Gbp6 |
| 10385518 | 0.28 | 2.48 | -0.09 | -0.77 | Tgtp1 |
| 10385533 | 0.23 | 2.47 | -0.21 | -0.73 | Tgtp1 |
| 10403015 | 0.57 | 2.45 | 0.05 | -0.07 | Ighv1-22 |
| 10574098 | 0.91 | 2.42 | 0.37 | -0.09 | Nlrc5 |
| 10360406 | 0.72 | 2.38 | 0.04 | -0.59 | Ifi205 |
| 10524621 | 0.57 | 2.37 | -0.16 | -0.62 | Oasl2 |
| 10487597 | 0.60 | 2.36 | -0.19 | 0.66 | Il1b |
| 10444244 | 0.58 | 2.34 | 0.30 | 0.02 | Tap1 |
| 10349648 | 0.51 | 2.33 | -1.95 | -0.96 | Ctse |
| 10376326 | 0.54 | 2.33 | -1.04 | -1.02 | Igtp |
| 10531415 | 1.08 | 2.31 | -0.37 | -1.39 | Cxcl10 |
| 10393573 | 1.03 | 2.29 | 0.10 | 0.02 | Lgals3bp |
| 10461594 | 0.42 | 2.29 | 0.37 | -0.65 | Ms4a4c |
| 10341361 | -0.07 | 2.29 | 0.30 | 0.61 | Unidentified |
| 10593015 | 0.33 | 2.28 | 0.54 | -0.20 | Cd3g |
| 10574102 | 0.61 | 2.28 | 0.27 | -0.16 | Nlrc5 |
| 10379228 | -0.01 | 2.27 | 0.00 | -0.13 | Nos2 |
| 10398907 | 0.92 | 2.27 | 0.07 | -0.18 | Pld4 |
| 10534667 | 1.15 | 2.26 | 0.45 | 0.16 | Serpine1 |
| 10450699 | 0.91 | 2.23 | 0.27 | 0.00 | Gm11127 |
| 10531737 | 1.30 | 2.23 | -0.14 | 0.17 | Hpse |
| 10347335 | 1.07 | 2.22 | 0.35 | 0.33 | Slc11a1 |
| 10379511 | 1.33 | 2.21 | 0.11 | -0.25 | Ccl2 |
| 10444821 | 0.45 | 2.20 | 0.63 | 0.03 | H2-Q8 |
| 10499899 | 1.56 | 2.20 | 0.02 | -0.45 | Sprr1a |
| 10450325 | 0.35 | 2.18 | -0.24 | -0.61 | Cfb |
| 10455957 | 0.49 | 2.16 | 0.21 | 0.04 | Unidentified |
| 10387890 | 1.32 | 2.16 | 0.25 | 0.01 | Cxcl16 |
| 10496569 | 0.34 | 2.15 | -0.06 | -0.59 | Gbp7 |
| 10533720 | 0.97 | 2.13 | -0.26 | -0.70 | Hcar2 |
| 10545173 | 1.65 | 2.12 | -0.22 | -0.12 | Igkv10-96 |
| 10569017 | 1.29 | 2.10 | -0.10 | 0.13 | Ifitm3 |
| 10582997 | 1.20 | 2.10 | 0.15 | 0.43 | Casp4 |
| 10358224 | 0.98 | 2.10 | 0.13 | -0.06 | Ptprc |
| 10531972 | -0.06 | 2.09 | 0.13 | -0.59 | Gbp8 |
| 10574145 | 0.53 | 2.07 | 0.18 | -0.13 | Nlrc5 |
| 10566358 | 0.76 | 2.06 | 0.25 | -0.13 | Trim30a |
| 10531987 | -0.12 | 2.06 | 0.30 | -0.20 | Gbp4 |
| 10579347 | 1.41 | 2.05 | 0.29 | -0.49 | Ifi30 |
| 10446282 | 0.61 | 2.04 | -0.47 | -0.54 | Adgre1 |
| 10411595 | 0.54 | 2.04 | -0.02 | 0.38 | Naip2 |
| 10597279 | 0.80 | 2.03 | -0.79 | -0.46 | Ccrl2 |
| 10444258 | 0.35 | 2.02 | 0.24 | -0.10 | Psmb8 |
| 10444236 | 0.43 | 2.01 | 0.29 | -0.26 | H2-DMb2 |
| 10379535 | 1.17 | 2.00 | -0.12 | -0.13 | Ccl8 |
| 10551883 | 1.27 | 2.00 | 0.04 | 0.46 | Tyrobp |
| 10346191 | 0.15 | 1.99 | -0.32 | -0.66 | Stat1 |
| 10587683 | 1.00 | 1.99 | -0.08 | -0.35 | Bcl2a1a |
| 10541683 | 0.83 | 1.99 | 0.18 | 0.01 | C1rb |
| 10446253 | 0.77 | 1.98 | 0.38 | 0.33 | Vav1 |
| 10376060 | 0.33 | 1.97 | 0.07 | -0.29 | Irf1 |
| 10416437 | 1.01 | 1.97 | -0.10 | -0.03 | Lcp1 |
| 10450242 | 0.67 | 1.95 | 0.28 | 0.40 | C4b |
| 10375145 | 0.68 | 1.95 | 0.23 | 0.30 | Lcp2 |
| 10496539 | 0.10 | 1.94 | 0.21 | -0.43 | Gbp5 |
| 10376324 | 0.12 | 1.94 | -0.24 | -0.68 | Gm12250 |
| 10587733 | 0.95 | 1.93 | 0.21 | 0.23 | Ctsh |
| 10439312 | 0.85 | 1.92 | 0.13 | 0.27 | Cd86 |
| 10587690 | 0.93 | 1.92 | -0.02 | -0.37 | Bcl2a1b |
| 10519497 | 1.24 | 1.91 | 0.18 | 0.37 | Steap4 |
| 10365769 | 1.81 | 1.90 | 0.05 | 0.34 | Hal |
| 10457640 | 1.07 | 1.90 | 0.05 | 0.24 | S100a11 |
| 10489204 | 0.73 | 1.89 | -0.30 | -0.49 | Tgm2 |
| 10473809 | 0.95 | 1.89 | 0.07 | 0.51 | Spi1 |
| 10608646 | 0.41 | 1.88 | -0.07 | -0.27 | Unidentified |
| 10391798 | 1.74 | 1.88 | -0.99 | -0.88 | Gfap |
| 10525365 | 0.80 | 1.88 | 0.22 | 0.06 | Hvcn1 |
| 10595633 | 0.96 | 1.88 | -0.05 | -0.35 | Bcl2a1d |
| 10520452 | 0.14 | 1.87 | -0.20 | 0.09 | Il6 |
| 10368343 | 1.30 | 1.86 | 0.08 | 0.02 | Arg1 |
| 10385118 | 0.62 | 1.84 | 0.23 | 0.03 | Dock2 |
| 10548892 | 0.50 | 1.84 | 0.17 | 0.24 | Arhgdib |
| 10534927 | 0.30 | 1.84 | -0.39 | 0.25 | Pilra |
| 10375443 | 1.13 | 1.84 | 0.18 | 0.22 | Havcr2 |
| 10462623 | 0.61 | 1.83 | -1.09 | -0.90 | Ifit1 |
| 10341452 | 1.11 | 1.83 | 0.18 | -0.23 | Unidentified |
| 10450880 | -0.01 | 1.83 | -0.22 | -0.48 | H2-M2 |
| 10433172 | 0.60 | 1.82 | -0.63 | -2.35 | Glycam1 |
| 10372410 | 0.82 | 1.82 | -0.02 | 0.09 | Glipr1 |
| 10574149 | 0.44 | 1.81 | 0.27 | -0.12 | Nlrc5 |
| 10469816 | 0.58 | 1.81 | -0.06 | -0.21 | Il1rn |
| 10541605 | 0.76 | 1.81 | -0.09 | 0.06 | Clec4n |
| 10430372 | 0.67 | 1.80 | 0.21 | -0.14 | Rac2 |
| 10545175 | 0.96 | 1.79 | -0.12 | -0.15 | Igk-J1 |
| 10475414 | 0.72 | 1.79 | 0.10 | 0.04 | B2m |
| 10343030 | -0.27 | 1.79 | -0.45 | 0.40 | Unidentified |
| 10441003 | 0.96 | 1.79 | -0.02 | 0.10 | Runx1 |
| 10508663 | 0.68 | 1.78 | 0.30 | 0.16 | Laptm5 |
| 10538903 | 1.50 | 1.78 | -0.17 | -0.03 | Igkv8-30 |
| 10527441 | 1.10 | 1.78 | -0.01 | 0.11 | Arpc1b |
| 10485405 | 1.41 | 1.78 | -0.10 | 0.24 | Cd44 |
| 10404606 | 0.82 | 1.78 | 0.19 | -0.12 | Ly86 |
| 10403018 | 0.46 | 1.77 | 0.07 | -0.11 | Ighv1-26 |
| 10351197 | 0.40 | 1.77 | 0.40 | 0.11 | Sell |
| 10441601 | 0.83 | 1.77 | 0.36 | -0.07 | Tagap |
| 10591739 | 0.79 | 1.77 | 0.55 | 0.25 | Acp5 |
| 10574104 | 0.49 | 1.77 | 0.33 | -0.05 | Nlrc5 |
| 10574139 | 0.52 | 1.76 | 0.29 | -0.13 | Nlrc5 |
| 10375515 | 0.32 | 1.75 | -0.39 | -0.26 | Ifi47 |
| 10469322 | 1.50 | 1.74 | 0.00 | 0.19 | Vim |
| 10467136 | 0.80 | 1.74 | 0.40 | 0.20 | Ch25h |
| 10461558 | 0.80 | 1.74 | 0.15 | -0.12 | Slc15a3 |
| 10493990 | 0.96 | 1.73 | 0.04 | 0.20 | S100a11 |
| 10427336 | 0.99 | 1.73 | 0.29 | 0.21 | Nckap1l |
| 10604763 | 1.04 | 1.72 | -0.07 | 0.18 | Arpc1b |
| 10348244 | 0.74 | 1.71 | 0.33 | 0.43 | Inpp5d |
| 10434778 | 0.47 | 1.70 | -0.26 | -0.57 | Rtp4 |
| 10450374 | 0.62 | 1.70 | -0.11 | -0.30 | D17H6S56E-5 |
| 10403034 | 2.00 | 1.70 | 0.04 | 0.12 | LOC100046275 |
| 10490212 | 0.86 | 1.70 | 0.30 | 0.08 | Ctsz |
| 10539135 | 0.92 | 1.69 | 0.18 | -0.35 | Capg |
| 10496580 | 0.29 | 1.69 | -0.01 | -0.21 | Gbp3 |
| 10572897 | 0.91 | 1.69 | -0.43 | 0.09 | Hmox1 |
| 10508734 | 0.92 | 1.69 | 0.01 | 0.08 | Ptafr |
| 10576034 | 0.69 | 1.69 | 0.26 | -0.31 | Irf8 |
| 10403048 | 0.74 | 1.68 | 0.06 | 0.06 | Unidentified |
| 10462618 | 0.69 | 1.68 | -0.50 | -1.21 | Ifit3 |
| 10406928 | 0.97 | 1.68 | 0.30 | -0.52 | Cd180 |
| 10460237 | 0.58 | 1.66 | 0.39 | -0.12 | Unc93b1 |
| 10608650 | 0.43 | 1.66 | 0.24 | -0.04 | Unidentified |
| 10396476 | 1.25 | 1.66 | -0.09 | 0.44 | Rhoj |
| 10600836 | 0.90 | 1.64 | 0.20 | -0.04 | Msn |
| 10466200 | 1.21 | 1.62 | -0.10 | 0.40 | Ms4a7 |
| 10379633 | 0.46 | 1.61 | 0.43 | 0.19 | Slfn1 |
| 10459066 | 0.35 | 1.61 | 0.03 | -0.60 | Gm4841 |
| 10389143 | 0.44 | 1.60 | 0.25 | -0.03 | Slfn8 |
| 10564818 | 1.44 | 1.59 | 0.13 | 0.17 | Anpep |
| 10574135 | 0.26 | 1.59 | 0.34 | -0.12 | Nlrc5 |
| 10344291 | 0.21 | 1.58 | 0.03 | 0.46 | Unidentified |
| 10562192 | 0.85 | 1.57 | 0.37 | 0.19 | Fxyd5 |
| 10555389 | 1.08 | 1.57 | -0.42 | -0.37 | Ucp2 |
| 10545215 | 0.94 | 1.57 | -0.07 | 0.04 | Igk-V28 |
| 10351873 | 0.30 | 1.57 | 0.42 | 0.05 | Pyhin1 |
| 10518300 | 0.82 | 1.56 | 0.28 | 0.33 | Tnfrsf1b |
| 10534202 | 0.68 | 1.56 | -0.29 | 0.54 | Ncf1 |
| 10531126 | 1.53 | 1.55 | -0.21 | 0.01 | Jchain |
| 10502791 | 0.85 | 1.54 | -0.59 | -1.22 | Ifi44 |
| 10381588 | 0.92 | 1.54 | 0.19 | 0.27 | Grn |
| 10571840 | 0.71 | 1.54 | -0.11 | -0.10 | Hpgd |
| 10392815 | 0.45 | 1.53 | -0.09 | 0.30 | Cd300c2 |
| 10589884 | 1.16 | 1.53 | -0.16 | -0.16 | Bcl2a1c |
| 10559454 | 1.06 | 1.53 | 0.05 | 0.26 | Pira11 |
| 10531420 | -0.15 | 1.52 | 0.12 | -0.47 | Cxcl11 |
| 10583519 | 0.59 | 1.51 | 0.09 | -0.28 | Icam1 |
| 10566366 | 0.18 | 1.51 | 0.52 | -0.20 | Trim30d |
| 10548817 | 0.55 | 1.51 | -0.15 | 0.06 | Plbd1 |
| 10355403 | 0.65 | 1.50 | -0.72 | 0.42 | Fn1 |
| 10514221 | 1.14 | 1.50 | 0.32 | -0.05 | Plin2 |
| 10516966 | 0.51 | 1.50 | 0.07 | -0.48 | Themis2 |
| 10405179 | 1.19 | 1.49 | 0.13 | -0.71 | S1pr3 |
| 10541564 | 0.82 | 1.48 | -0.19 | -0.07 | Clec4a3 |
| 10403028 | 0.86 | 1.48 | 0.06 | 0.12 | Ighv1-61 |
| 10435565 | 0.48 | 1.47 | 0.25 | 0.25 | Hcls1 |
| 10343411 | 0.16 | 1.46 | -0.74 | 0.01 | Unidentified |
| 10496077 | 1.41 | 1.44 | 0.04 | -0.10 | Etnppl |
| 10521678 | 0.78 | 1.44 | 0.24 | -0.02 | Cd38 |
| 10444068 | 0.29 | 1.44 | 0.06 | -0.24 | Tapbp |
| 10403060 | 0.69 | 1.43 | 0.07 | 0.11 | Ighv1-61 |
| 10583008 | 0.97 | 1.43 | -0.06 | 0.26 | Casp12 |
| 10385513 | 0.59 | 1.43 | 0.42 | -0.40 | Gm5431 |
| 10469278 | 0.37 | 1.43 | 0.65 | 0.18 | Il2ra |
| 10412123 | 0.59 | 1.43 | 0.11 | 0.24 | Ncf2 |
| 10572497 | 0.17 | 1.43 | 0.16 | -0.12 | Il12rb1 |
| 10342539 | 0.09 | 1.42 | 0.19 | 0.31 | Unidentified |
| 10456071 | 0.43 | 1.42 | 0.15 | 0.37 | Csf1r |
| 10538924 | 0.35 | 1.42 | 0.05 | -0.28 | Igkv2-109 |
| 10443980 | 0.99 | 1.40 | 0.05 | 0.38 | Myo1f |
| 10360173 | 0.60 | 1.40 | 0.19 | -0.43 | Slamf7 |
| 10445119 | 0.11 | 1.39 | 0.52 | -0.03 | H2-M3 |
| 10560685 | 0.82 | 1.39 | 0.05 | 0.10 | Bcl3 |
| 10450145 | 0.20 | 1.39 | 0.03 | -0.30 | Psmb9 |
| 10604393 | 0.59 | 1.38 | 0.33 | 0.13 | Elf4 |
| 10435920 | 0.64 | 1.38 | -0.22 | -0.05 | Cd200r4 |
| 10541354 | 1.70 | 1.38 | 0.22 | -0.13 | A2m |
| 10565958 | 0.83 | 1.37 | 0.22 | -0.18 | P2ry6 |
| 10346799 | 0.22 | 1.37 | 0.15 | -0.16 | Icos |
| 10467578 | 0.75 | 1.37 | 0.13 | -0.32 | Pik3ap1 |
| 10439249 | 0.37 | 1.37 | -0.01 | -0.36 | Parp14 |
| 10351658 | 0.79 | 1.36 | 0.04 | -0.14 | Cd48 |
| 10477250 | 0.51 | 1.36 | -0.02 | 0.14 | Hck |
| 10497358 | 0.34 | 1.34 | 0.43 | -0.07 | Sirpb1a |
| 10344598 | 0.74 | 1.34 | 0.39 | 0.28 | Unidentified |
| 10385504 | 0.27 | 1.34 | 0.21 | -0.12 | Gm5431 |
| 10559207 | 0.46 | 1.34 | 0.09 | -0.15 | Lsp1 |
| 10419744 | 0.41 | 1.34 | 0.36 | 0.02 | Slc7a7 |
| 10347928 | 0.68 | 1.34 | 0.13 | -0.18 | Gm16026 |
| 10582874 | 0.68 | 1.34 | 0.13 | -0.18 | Gm16026 |
| 10443527 | 0.42 | 1.33 | -0.67 | 0.04 | Pim1 |
| 10403063 | 1.83 | 1.32 | 0.05 | 0.01 | LOC100046275 |
| 10608680 | 0.30 | 1.32 | 0.22 | -0.12 | Unidentified |
| 10385526 | 0.55 | 1.32 | 0.38 | -0.39 | Gm5431 |
| 10444814 | 0.63 | 1.32 | 0.44 | 0.07 | H2-Q2 |
| 10403038 | 0.93 | 1.31 | 0.06 | 0.26 | Ighv1-61 |
| 10402211 | 1.25 | 1.31 | -0.13 | -0.09 | Fbln5 |
| 10405216 | 0.56 | 1.31 | 0.42 | 0.27 | Syk |
| 10351691 | 0.33 | 1.30 | 0.47 | 0.13 | Slamf6 |
| 10388902 | 0.51 | 1.30 | -0.27 | -0.23 | Lgals9 |
| 10574157 | 0.36 | 1.30 | 0.28 | -0.15 | Nlrc5 |
| 10450682 | 0.58 | 1.28 | 0.23 | -0.14 | H2-T23 |
| 10345762 | 0.62 | 1.28 | 0.09 | 0.29 | Il1r1 |
| 10398039 | 0.20 | 1.27 | 0.17 | -0.10 | Serpina3f |
| 10498992 | 0.51 | 1.26 | 0.05 | 0.16 | Tlr2 |
| 10447591 | 0.55 | 1.26 | -0.03 | -0.05 | Ftl2-ps |
| 10569319 | 0.85 | 1.25 | -0.07 | 0.28 | Ctsd |
| 10542355 | 1.35 | 1.25 | 0.17 | -0.09 | Emp1 |
| 10403021 | 0.36 | 1.25 | 0.63 | -0.58 | Ighv1-43 |
| 10361091 | 0.88 | 1.25 | -0.11 | 0.20 | Atf3 |
| 10372652 | 1.00 | 1.25 | -0.50 | -0.45 | Lyz1 |
| 10429564 | 1.49 | 1.24 | -0.09 | -0.45 | Ly6a |
| 10500335 | 0.73 | 1.23 | -0.16 | 0.08 | Fcgr1 |
| 10403043 | 0.41 | 1.22 | 0.04 | 0.08 | Ighv1-62-3 |
| 10433114 | 0.62 | 1.21 | -0.29 | 0.04 | Itga5 |
| 10406198 | 0.53 | 1.21 | -0.02 | -0.01 | Ftl2-ps |
| 10482059 | 0.78 | 1.20 | 0.23 | 0.14 | Ggta1 |
| 10563295 | 0.52 | 1.19 | -0.01 | -0.01 | Ftl1 |
| 10587383 | 1.45 | 1.18 | 0.06 | 0.04 | Cd109 |
| 10563178 | 0.70 | 1.18 | 0.36 | -0.29 | Cd37 |
| 10458314 | 0.37 | 1.17 | 0.37 | -0.28 | Tmem173 |
| 10444658 | 0.62 | 1.17 | 0.11 | 0.32 | Clic1 |
| 10508069 | 0.52 | 1.17 | -0.01 | 0.00 | Ftl2-ps |
| 10464471 | 0.66 | 1.16 | 0.61 | -0.22 | Gal |
| 10467258 | 0.79 | 1.16 | 0.00 | 0.24 | Myof |
| 10403069 | 0.80 | 1.15 | -0.34 | 0.38 | Ighv1-74 |
| 10346564 | 0.63 | 1.15 | 0.25 | 0.02 | Casp8 |
| 10538150 | 0.95 | 1.13 | 0.55 | 0.14 | Tmem176a |
| 10425092 | 0.74 | 1.02 | 0.14 | -0.11 | Cyth4 |
| 10379518 | 1.10 | 1.02 | 0.05 | -0.10 | Ccl7 |
| 10516064 | 0.98 | 1.02 | -0.09 | -0.01 | Mfsd2a |
| 10557895 | 1.17 | 0.91 | 0.24 | 0.08 | Itgax |

**Supplemental Table 7.** List of genes in cluster 11

|  | **Log ratio** | | | |  |
| --- | --- | --- | --- | --- | --- |
| **Probe Set ID** | **A.SW_Brain** | **SJL_Brain** | **A.SW_Spleen** | **SJL_Spleen** | **Symbol** |
| 10341669 | 2.36 | 0.92 | -0.03 | 0.57 | Unidentified |
| 10342422 | 1.61 | 0.41 | -0.32 | 0.75 | Unidentified |
| 10427471 | 1.59 | 1.90 | 0.91 | 1.12 | Osmr |
| 10490989 | 1.51 | 1.99 | 0.81 | 0.80 | Cp |
| 10583056 | 1.36 | 1.36 | 0.03 | 1.06 | Mmp12 |
| 10478048 | 1.34 | 1.16 | 0.00 | 2.29 | Lbp |
| 10474700 | 1.30 | 1.28 | -1.31 | 1.04 | Thbs1 |
| 10443463 | 1.27 | 1.58 | 0.52 | 0.98 | Cdkn1a |
| 10342498 | 1.26 | 1.30 | 0.09 | 0.97 | Unidentified |
| 10452815 | 1.22 | 2.00 | 1.08 | 1.91 | Xdh |
| 10458894 | 1.18 | 2.19 | 1.44 | 2.78 | Lox |
| 10560329 | 1.18 | 1.29 | 0.72 | 0.58 | Hif3a |
| 10607868 | 1.17 | 2.01 | -0.01 | 1.13 | Tlr8 |
| 10358476 | 1.14 | 1.01 | 0.27 | 0.55 | Prg4 |
| 10494428 | 1.13 | 1.61 | 0.83 | 0.71 | Txnip |
| 10608675 | 1.13 | 0.85 | 0.97 | 1.49 | Unidentified |
| 10558769 | 1.12 | 1.49 | 1.17 | 0.87 | Ifitm1 |
| 10362073 | 1.11 | 1.40 | 0.66 | 0.81 | Sgk1 |
| 10383756 | 1.09 | 1.43 | 0.52 | 0.67 | Ifitm2 |
| 10367400 | 1.08 | 1.08 | 0.26 | 1.00 | Mmp19 |
| 10574027 | 1.08 | 1.21 | 0.28 | 0.94 | Mt1 |
| 10360040 | 1.06 | 2.16 | 0.29 | 0.97 | Fcgr3 |
| 10553299 | 1.06 | 1.43 | 0.49 | 0.67 | Ifitm2 |
| 10560618 | 1.05 | 0.90 | 0.68 | 0.57 | Apoc1 |
| 10587799 | 1.05 | 0.82 | -0.04 | 1.19 | Plscr2 |
| 10358339 | 1.05 | 1.56 | 0.89 | 1.68 | Cfh |
| 10523451 | 1.04 | 1.03 | -0.73 | 1.24 | Anxa3 |
| 10570434 | 1.04 | 1.55 | 0.98 | 0.96 | Ifitm1 |
| 10560242 | 1.03 | 1.36 | 0.02 | 1.87 | C5ar1 |
| 10598004 | 1.01 | 2.00 | 0.17 | 2.29 | Ccr1 |
| 10568001 | 1.01 | 1.16 | 1.41 | 1.49 | Sult1a1 |
| 10382106 | 1.00 | 1.18 | 0.28 | 0.71 | Milr1 |
| 10541555 | 1.00 | 1.39 | 0.12 | 0.53 | Clec4a1 |
| 10495596 | 0.99 | 1.12 | -0.54 | 0.57 | Frrs1 |
| 10453057 | 0.99 | 1.47 | 1.47 | 1.06 | Cyp1b1 |
| 10512949 | 0.96 | 1.16 | 1.18 | 1.01 | Abca1 |
| 10470959 | 0.96 | 1.54 | 0.90 | 0.86 | Phyhd1 |
| 10384458 | 0.94 | 1.76 | -0.44 | 0.77 | Plek |
| 10384223 | 0.93 | 1.26 | 1.08 | 0.92 | Igfbp3 |
| 10408693 | 0.90 | 2.41 | -1.20 | 2.16 | F13a1 |
| 10469358 | 0.90 | 0.99 | 0.20 | 1.00 | Mrc1 |
| 10581434 | 0.89 | 1.47 | 0.46 | 0.65 | Dpep2 |
| 10489246 | 0.88 | 1.20 | 0.33 | 0.48 | Mafb |
| 10389214 | 0.88 | 2.35 | 0.27 | 1.54 | Ccl9 |
| 10358408 | 0.87 | 1.98 | 0.71 | 1.95 | Rgs1 |
| 10569014 | 0.87 | 1.27 | 0.54 | 0.80 | Ifitm2 |
| 10562709 | 0.86 | 2.29 | 0.43 | 0.83 | Cd33 |
| 10452633 | 0.86 | 1.45 | 0.53 | 0.36 | Tgif1 |
| 10466190 | 0.86 | 1.07 | 0.14 | 0.31 | Ms4a14 |
| 10573583 | 0.82 | 1.53 | 0.46 | 0.54 | Man2b1 |
| 10608654 | 0.82 | 1.28 | 0.82 | 1.63 | Unidentified |
| 10568873 | 0.81 | 1.35 | -0.10 | 0.58 | Adam8 |
| 10532711 | 0.81 | 1.45 | 0.45 | 0.60 | Cmklr1 |
| 10425053 | 0.81 | 1.14 | -0.09 | 0.46 | Ncf4 |
| 10357472 | 0.78 | 0.82 | 0.81 | 0.78 | Cxcr4 |
| 10600852 | 0.78 | 0.89 | -0.23 | 1.23 | F630028O10Rik |
| 10557862 | 0.78 | 1.75 | -0.69 | 1.53 | Itgam |
| 10410124 | 0.77 | 0.77 | 0.49 | 1.17 | Ctsl |
| 10508829 | 0.77 | 1.14 | 0.61 | 1.03 | Map3k6 |
| 10393449 | 0.77 | 2.00 | 0.32 | 0.73 | Socs3 |
| 10586744 | 0.77 | 0.89 | -0.31 | 0.70 | Anxa2 |
| 10400405 | 0.76 | 1.50 | 0.61 | 0.63 | Nfkbia |
| 10512129 | 0.76 | 1.42 | -0.10 | 0.49 | B4galt1 |
| 10398052 | 0.76 | 1.87 | 0.61 | 0.65 | Serpina3g |
| 10475890 | 0.76 | 0.97 | 0.55 | 0.79 | Mertk |
| 10436456 | 0.75 | 0.90 | 0.02 | 0.82 | Pros1 |
| 10558921 | 0.75 | 1.22 | 0.72 | 0.90 | Pnpla2 |
| 10365974 | 0.75 | 0.82 | 0.65 | 1.16 | Dcn |
| 10508074 | 0.74 | 1.84 | 0.01 | 1.07 | Csf3r |
| 10360398 | 0.73 | 1.70 | -0.26 | 0.64 | Ifi202b |
| 10488382 | 0.73 | 0.99 | 0.27 | 0.50 | Cd93 |
| 10345777 | 0.73 | 0.81 | 0.23 | 0.63 | Il1rl2 |
| 10571444 | 0.73 | 1.09 | 0.89 | 0.43 | Slc7a2 |
| 10351825 | 0.72 | 1.17 | -0.06 | 0.40 | Tagln2 |
| 10397645 | 0.70 | 2.13 | 0.53 | 1.19 | Gpr65 |
| 10454198 | 0.70 | 1.16 | -0.09 | 1.01 | Rnf125 |
| 10343229 | 0.70 | 1.04 | 0.37 | 0.52 | Unidentified |
| 10543239 | 0.69 | 1.42 | -0.05 | 0.91 | Tfec |
| 10541895 | 0.69 | 1.06 | 0.26 | 0.72 | Tnfrsf1a |
| 10348354 | 0.69 | 1.07 | 0.28 | 1.49 | Ugt1a9 |
| 10343092 | 0.69 | 1.30 | 0.62 | 0.44 | Unidentified |
| 10519983 | 0.67 | 2.14 | 0.15 | 0.74 | Fgl2 |
| 10464529 | 0.67 | 1.40 | 0.39 | 0.47 | Tcirg1 |
| 10433885 | 0.67 | 1.36 | 0.89 | 1.80 | Cebpd |
| 10429128 | 0.66 | 1.00 | 0.36 | 0.76 | Sla |
| 10341691 | 0.65 | 1.22 | 0.29 | 0.47 | Unidentified |
| 10500204 | 0.65 | 1.41 | 0.22 | 0.57 | Ecm1 |
| 10527638 | 0.64 | 1.55 | 0.13 | 1.88 | Alox5ap |
| 10542470 | 0.64 | 1.05 | 0.29 | 1.34 | Mgst1 |
| 10559446 | 0.64 | 1.30 | 0.38 | 0.64 | Pirb |
| 10561702 | 0.64 | 1.26 | 0.10 | 0.68 | Kcnk6 |
| 10344113 | 0.64 | 0.73 | 0.18 | 0.85 | Unidentified |
| 10367436 | 0.64 | 0.79 | -0.03 | 0.68 | Cd63 |
| 10459071 | 0.63 | 1.31 | -0.78 | 1.41 | Smim3 |
| 10417561 | 0.63 | 0.84 | 1.02 | 1.22 | Fam107a |
| 10548879 | 0.63 | 0.85 | 0.22 | 0.69 | Mgp |
| 10436100 | 0.62 | 1.20 | -0.48 | 2.08 | Retnlg |
| 10379389 | 0.62 | 1.56 | 0.89 | 0.82 | Adap2 |
| 10461636 | 0.62 | 1.64 | 0.17 | 0.48 | Unidentified |
| 10493820 | 0.62 | 1.01 | 0.08 | 0.79 | S100a6 |
| 10351867 | 0.62 | 1.37 | 0.41 | 0.52 | Aim2 |
| 10599174 | 0.61 | 1.09 | 0.11 | 0.56 | Il13ra1 |
| 10469867 | 0.60 | 1.04 | 0.60 | 0.58 | Pnpla7 |
| 10557326 | 0.60 | 1.09 | 0.48 | 0.37 | Il4ra |
| 10550906 | 0.60 | 1.01 | 0.58 | 1.40 | Plaur |
| 10438445 | 0.60 | 1.96 | 0.98 | 1.27 | Klhl6 |
| 10341357 | 0.59 | 1.78 | 0.35 | 0.53 | Unidentified |
| 10561453 | 0.59 | 1.73 | 0.47 | 0.48 | Zfp36 |
| 10570291 | 0.58 | 1.90 | -0.77 | 1.22 | F10 |
| 10462603 | 0.58 | 1.16 | 0.32 | 0.38 | Fas |
| 10537410 | 0.57 | 1.02 | -0.61 | 0.66 | Tbxas1 |
| 10338157 | 0.57 | 1.11 | 0.35 | 0.58 | Unidentified |
| 10418506 | 0.55 | 0.99 | 0.86 | 0.74 | Stab1 |
| 10482802 | 0.54 | 1.77 | 0.46 | 0.68 | Cytip |
| 10370721 | 0.53 | 1.21 | 0.17 | 0.50 | Sbno2 |
| 10544273 | 0.52 | 1.94 | -0.99 | 1.73 | Clec5a |
| 10403871 | 0.52 | 1.71 | -0.64 | 1.20 | Aoah |
| 10411373 | 0.52 | 1.11 | 0.21 | 0.52 | Hexb |
| 10338798 | 0.52 | 0.94 | 0.25 | 1.07 | Unidentified |
| 10430931 | 0.50 | 0.87 | -0.01 | 0.66 | Nfam1 |
| 10607467 | 0.50 | 1.11 | 0.24 | 0.54 | Sat1 |
| 10361897 | 0.50 | 1.01 | 0.38 | 0.74 | Ifngr1 |
| 10453272 | 0.50 | 0.88 | 0.90 | 1.18 | Zfp36l2 |
| 10379630 | 0.49 | 1.31 | 0.38 | 0.38 | Slfn2 |
| 10475517 | 0.49 | 1.50 | -0.20 | 2.00 | C15orf48 |
| 10597648 | 0.48 | 1.09 | 0.13 | 0.43 | Myd88 |
| 10529815 | 0.48 | 0.91 | 0.40 | 1.18 | Unidentified |
| 10444890 | 0.48 | 0.91 | 0.06 | 1.07 | Ier3 |
| 10501608 | 0.47 | 1.14 | 0.34 | 0.64 | Vcam1 |
| 10413609 | 0.47 | 1.25 | 0.71 | 0.40 | Mustn1 |
| 10542981 | 0.46 | 1.53 | 0.19 | 0.56 | Gmfg |
| 10343619 | 0.46 | 1.07 | 1.05 | 0.97 | Unidentified |
| 10429568 | 0.45 | 1.11 | -0.05 | 0.64 | Ly6c1 |
| 10392845 | 0.45 | 1.53 | 0.53 | 0.77 | Cd300lf |
| 10502655 | 0.43 | 1.34 | -0.24 | 0.74 | Cyr61 |
| 10429573 | 0.43 | 1.06 | -0.01 | 0.60 | Ly6c1 |
| 10399924 | 0.43 | 1.36 | 0.19 | 0.76 | Pik3cg |
| 10448124 | 0.42 | 1.53 | 0.11 | 0.87 | Fpr1 |
| 10590801 | 0.40 | 1.28 | 0.47 | 0.43 | Birc3 |
| 10411611 | 0.40 | 1.12 | 0.29 | 0.43 | Naip5 |
| 10342932 | 0.39 | 0.95 | -0.02 | 1.35 | Unidentified |
| 10583100 | 0.38 | 2.48 | -1.05 | 2.36 | Mmp8 |
| 10358879 | 0.36 | 1.90 | -0.10 | 0.88 | Npl |
| 10409876 | 0.36 | 1.55 | 0.21 | 1.16 | Ctla2a |
| 10342682 | 0.35 | 0.99 | -0.06 | 0.72 | Unidentified |
| 10442596 | 0.35 | 0.91 | -0.08 | 1.03 | Msrb1 |
| 10341319 | 0.35 | 1.09 | 0.77 | 1.51 | Unidentified |
| 10531724 | 0.33 | 1.49 | 0.17 | 0.62 | Plac8 |
| 10537227 | 0.32 | 1.15 | 0.36 | 0.48 | Tmem140 |
| 10409866 | 0.32 | 0.79 | 0.01 | 0.91 | Ctla2b |
| 10445746 | 0.31 | 1.00 | 0.29 | 1.18 | Trem1 |
| 10372781 | 0.30 | 1.26 | 0.63 | 0.68 | Irak3 |
| 10466130 | 0.26 | 1.49 | 0.62 | 1.12 | Ms4a8a |
| 10340582 | 0.25 | 1.23 | -0.39 | 0.75 | Unidentified |
| 10342497 | 0.24 | 1.03 | 0.36 | 0.71 | Unidentified |
| 10463070 | 0.22 | 1.11 | 0.48 | 0.58 | Entpd1 |
| 10487208 | 0.22 | 1.06 | -0.16 | 1.33 | Atp8b4 |
| 10548552 | 0.22 | 0.98 | -0.29 | 0.76 | Klra2 |
| 10469786 | 0.19 | 2.55 | -0.53 | 1.24 | Il1f9 |
| 10340299 | 0.18 | 0.81 | -0.18 | 1.04 | Unidentified |
| 10413152 | 0.17 | 1.03 | 0.33 | 0.70 | C10orf11 |
| 10404783 | 0.10 | 1.06 | 0.63 | 1.09 | Edn1 |
| 10548333 | 0.08 | 1.35 | 0.79 | 0.91 | Cd69 |
| 10396831 | 0.05 | 0.96 | 0.19 | 1.59 | Arg2 |
| 10407985 | 0.01 | 1.19 | 0.43 | 1.59 | Gpr141 |
| 10601416 | -0.04 | 1.26 | 0.45 | 0.74 | P2ry10 |
| 10344363 | -0.04 | 1.74 | -0.18 | 1.03 | Unidentified |
| 10340197 | -0.15 | 1.28 | -0.01 | 0.68 | Unidentified |

**Supplemental Table 8.** List of genes in cluster 15

|  | **Log ratio** | | | |  |
| --- | --- | --- | --- | --- | --- |
| **Probe Set ID** | **A.SW_Brain** | **SJL_Brain** | **A.SW_Spleen** | **SJL_Spleen** | **Symbol** |
| 10558673 | 0.06 | -0.26 | 2.14 | 0.10 | Cyp2e1 |
| 10494643 | 0.69 | 0.25 | 2.01 | 0.53 | Hmgcs2 |
| 10448916 | 0.25 | -0.08 | 1.90 | 0.13 | Tpsab1 |
| 10504137 | -0.01 | 0.21 | 1.83 | 0.24 | 4933409K07Rik |
| 10504201 | -0.01 | 0.21 | 1.83 | 0.24 | 4933409K07Rik |
| 10512350 | -0.01 | 0.21 | 1.83 | 0.24 | 4933409K07Rik |
| 10512352 | -0.01 | 0.21 | 1.83 | 0.24 | 4933409K07Rik |
| 10600069 | -0.08 | -0.29 | 1.80 | -0.20 | Gabrq |
| 10340392 | -0.84 | 0.18 | 1.52 | 0.17 | Unidentified |
| 10477600 | 0.09 | -0.03 | 1.46 | 0.33 | Unidentified |
| 10512332 | 0.14 | 0.19 | 1.42 | 0.42 | Unidentified |
| 10512354 | 0.14 | 0.19 | 1.42 | 0.42 | Unidentified |
| 10604944 | -0.03 | -0.33 | 1.35 | 0.07 | Gabre |
| 10346374 | 0.19 | 0.08 | 1.29 | 0.69 | Aox1 |
| 10343692 | -0.15 | -0.12 | 1.29 | 0.36 | Unidentified |
| 10344486 | -0.01 | -0.61 | 1.28 | 0.27 | Unidentified |
| 10529485 | 0.29 | 0.18 | 1.21 | 0.65 | Htra3 |
| 10340474 | -0.37 | 0.42 | 1.20 | 0.61 | Unidentified |
| 10402981 | 0.02 | -0.19 | 1.19 | -0.69 | Ighe |
| 10551626 | 0.09 | 0.04 | 1.17 | 0.32 | Lgals4 |
| 10598081 | -0.51 | 0.24 | 1.17 | 0.47 | Unidentified |
| 10561690 | -0.21 | 0.16 | 1.14 | 0.39 | Catsperg1 |
| 10545224 | 0.13 | 0.20 | 1.14 | -0.14 | Unidentified |
| 10342781 | 0.25 | -0.12 | 1.14 | -0.61 | Unidentified |
| 10422164 | -0.04 | -0.29 | 1.13 | 0.44 | Ednrb |
| 10459227 | 0.10 | 0.32 | 1.12 | 0.36 | mir-143 |
| 10346410 | -0.13 | -0.24 | 1.10 | 0.37 | Aox3 |
| 10527878 | 0.02 | -0.30 | 1.10 | 0.18 | V1rg10 |
| 10497265 | 0.32 | 0.17 | 1.10 | 0.46 | Fabp4 |
| 10572647 | 0.03 | 0.15 | 1.09 | 0.49 | Slc27a1 |
| 10338753 | 0.21 | -0.13 | 1.09 | 0.11 | Unidentified |
| 10598089 | 0.12 | 0.46 | 1.09 | 0.19 | Unidentified |
| 10549990 | 0.06 | -0.28 | 1.08 | 0.15 | V1rg10 |
| 10496438 | 0.30 | -0.77 | 1.08 | 0.33 | Adh1 |
| 10360248 | 0.31 | 0.25 | 1.06 | 0.18 | Atp1a4 |
| 10577240 | 0.03 | -0.15 | 1.05 | 0.50 | Csmd1 |
| 10544573 | 0.35 | 0.59 | 1.03 | 0.22 | Rarres2 |
| 10425799 | 0.11 | -0.33 | 1.01 | 0.26 | Rnu12 |
| 10450038 | 0.59 | 0.66 | 1.00 | -0.03 | Angptl4 |
| 10344459 | -0.05 | 0.32 | 0.99 | 0.61 | Unidentified |
| 10339282 | 0.01 | -0.21 | 0.99 | 0.33 | Unidentified |
| 10508723 | 0.12 | 0.08 | 0.98 | 0.56 | Snora61 |
| 10432675 | -0.10 | 0.29 | 0.98 | 0.20 | Unidentified |
| 10472235 | 0.34 | -0.34 | 0.98 | 0.07 | Dapl1 |
| 10486041 | -0.14 | -0.20 | 0.98 | 0.40 | Meis2 |
| 10339333 | -0.64 | -0.14 | 0.97 | -0.09 | Unidentified |
| 10601312 | 0.01 | -0.19 | 0.96 | 0.42 | Unidentified |
| 10338362 | -0.50 | 0.44 | 0.96 | 0.50 | Unidentified |
| 10604954 | -0.06 | -0.04 | 0.95 | -0.15 | mir-224 |
| 10368638 | -0.14 | -0.06 | 0.95 | 0.37 | Fam26e |
| 10544338 | -0.42 | 0.21 | 0.94 | 0.42 | Prss3 |
| 10586718 | 0.01 | -0.20 | 0.94 | 0.32 | Unidentified |
| 10421269 | 0.17 | 0.07 | 0.94 | 0.55 | Sorbs3 |
| 10444332 | 0.22 | 0.28 | 0.93 | 0.31 | BC051142 |
| 10508800 | 0.48 | 0.41 | 0.93 | 0.11 | Gm3579 |
| 10573865 | 0.48 | 0.41 | 0.93 | 0.11 | Gm3579 |
| 10341961 | 0.44 | -0.25 | 0.92 | 0.06 | Unidentified |
| 10360270 | 0.01 | -0.18 | 0.92 | 0.37 | Atp1a2 |
| 10419261 | 0.51 | -0.15 | 0.92 | 0.37 | Bmp4 |
| 10601303 | 0.02 | -0.27 | 0.92 | 0.40 | Chic1 |
| 10543779 | 0.50 | 0.11 | 0.91 | 0.18 | mir-29 |
| 10571601 | 0.08 | -0.16 | 0.91 | 0.32 | Pdlim3 |
| 10356601 | 0.32 | -0.04 | 0.91 | 0.40 | Per2 |
| 10367532 | 0.20 | 0.51 | 0.91 | 0.24 | Tespa1 |
| 10468898 | -0.02 | 0.16 | 0.90 | -0.07 | Lax1 |
| 10563753 | 0.00 | 0.06 | 0.90 | 0.03 | Mrgprx2 |
| 10431393 | 0.13 | -0.02 | 0.90 | -0.04 | Mapk12 |
| 10551852 | 0.09 | -0.02 | 0.89 | 0.48 | Clip3 |
| 10424695 | -0.06 | 0.01 | 0.89 | 0.41 | Gpihbp1 |
| 10339100 | 0.26 | -0.12 | 0.88 | 0.39 | Unidentified |
| 10442786 | 0.20 | -0.07 | 0.87 | -0.01 | Tpsb2 |
| 10338518 | 0.22 | -0.06 | 0.87 | 0.08 | Unidentified |
| 10538979 | 0.09 | 0.21 | 0.87 | 0.16 | Cd8b1 |
| 10373325 | 0.51 | 0.45 | 0.86 | 0.12 | Gpr182 |
| 10468292 | 0.16 | -0.01 | 0.86 | 0.35 | D930027P08Rik |
| 10374998 | -0.03 | -0.35 | 0.86 | -0.13 | Gpr75 |
| 10564163 | 0.02 | -0.04 | 0.85 | 0.03 | Unidentified |
| 10564167 | 0.02 | -0.04 | 0.85 | 0.03 | Unidentified |
| 10564171 | 0.02 | -0.04 | 0.85 | 0.03 | Unidentified |
| 10564173 | 0.02 | -0.04 | 0.85 | 0.03 | Unidentified |
| 10564175 | 0.02 | -0.04 | 0.85 | 0.03 | Unidentified |
| 10564179 | 0.02 | -0.04 | 0.85 | 0.03 | Unidentified |
| 10564181 | 0.02 | -0.04 | 0.85 | 0.03 | Unidentified |
| 10564185 | 0.02 | -0.04 | 0.85 | 0.03 | Unidentified |
| 10564187 | 0.02 | -0.04 | 0.85 | 0.03 | Unidentified |
| 10564189 | 0.02 | -0.04 | 0.85 | 0.03 | Unidentified |
| 10564191 | 0.02 | -0.04 | 0.85 | 0.03 | Unidentified |
| 10564193 | 0.02 | -0.04 | 0.85 | 0.03 | Unidentified |
| 10564195 | 0.02 | -0.04 | 0.85 | 0.03 | Unidentified |
| 10564197 | 0.02 | -0.04 | 0.85 | 0.03 | Unidentified |
| 10564199 | 0.02 | -0.04 | 0.85 | 0.03 | Unidentified |
| 10564201 | 0.02 | -0.04 | 0.85 | 0.03 | Snord116 |
| 10564205 | 0.02 | -0.04 | 0.85 | 0.03 | Snord116 |
| 10564207 | 0.02 | -0.04 | 0.85 | 0.03 | Snord116 |
| 10445268 | 0.00 | 0.13 | 0.85 | 0.47 | Adgrf5 |
| 10435787 | 0.20 | 0.09 | 0.85 | 0.54 | Unidentified |
| 10512480 | -0.15 | 0.13 | 0.84 | 0.40 | Sit1 |
| 10382136 | 0.21 | 0.03 | 0.83 | 0.48 | 1810010H24Rik |
| 10478474 | -0.12 | -0.15 | 0.83 | 0.32 | Rbpjl |
| 10564161 | 0.02 | -0.04 | 0.83 | 0.04 | Unidentified |
| 10565819 | 0.17 | 0.47 | 0.83 | -0.06 | Slco2b1 |
| 10368556 | 0.00 | -0.24 | 0.82 | 0.21 | Hey2 |
| 10450482 | -0.06 | -0.24 | 0.82 | 0.39 | Unidentified |
| 10583870 | -0.07 | -0.08 | 0.82 | 0.22 | Bmper |
| 10341302 | 0.14 | 0.18 | 0.81 | 0.12 | Unidentified |
| 10368240 | 0.06 | -0.09 | 0.81 | -0.03 | Tcf21 |
| 10351880 | 0.38 | 0.22 | 0.80 | -0.13 | Unidentified |
| 10501048 | 0.27 | 0.42 | 0.80 | 0.14 | 2010016I18Rik |
| 10583207 | 0.20 | 0.12 | 0.80 | 0.13 | Maml2 |
| 10371627 | 0.15 | 0.02 | 0.80 | 0.44 | Mybpc1 |
| 10593878 | 0.11 | 0.04 | 0.80 | 0.42 | Snx33 |
| 10607619 | -0.17 | -0.26 | 0.79 | 0.20 | Cdkl5 |
| 10583203 | 0.14 | 0.07 | 0.79 | -0.07 | Phxr4 |
| 10455098 | -0.15 | -0.10 | 0.79 | 0.24 | Pcdhb14 |
| 10360684 | 0.01 | -0.24 | 0.79 | 0.23 | Ephx1 |
| 10564183 | 0.05 | -0.04 | 0.78 | 0.01 | Unidentified |
| 10594110 | 0.06 | -0.05 | 0.78 | 0.44 | Neo1 |
| 10494817 | -0.02 | -0.14 | 0.78 | 0.18 | Ngf |
| 10457091 | -0.01 | -0.18 | 0.78 | 0.18 | Neto1 |
| 10343259 | 0.18 | 0.25 | 0.78 | 0.34 | Unidentified |
| 10546430 | 0.31 | 0.15 | 0.77 | 0.38 | Adamts9 |
| 10338986 | 0.17 | 0.17 | 0.77 | 0.15 | Unidentified |
| 10466676 | -0.05 | -0.26 | 0.77 | 0.30 | 1110059E24Rik |
| 10529979 | -0.04 | -0.06 | 0.77 | 0.36 | Ppargc1a |
| 10482968 | -0.02 | -0.10 | 0.76 | 0.16 | Pla2r1 |
| 10412805 | -0.09 | 0.00 | 0.76 | 0.18 | Unidentified |
| 10371784 | 0.36 | -0.10 | 0.76 | 0.05 | Nr1h4 |
| 10586722 | 0.12 | -0.14 | 0.76 | 0.28 | F830001A07Rik |
| 10543781 | 0.48 | -0.03 | 0.76 | 0.35 | mir-29 |
| 10376956 | -0.01 | -0.14 | 0.76 | 0.08 | Hs3st3a1 |
| 10343882 | 0.18 | -0.18 | 0.76 | -0.03 | Unidentified |
| 10515242 | -0.03 | -0.08 | 0.76 | 0.35 | Nsun4 |
| 10468294 | 0.11 | -0.08 | 0.76 | 0.01 | Calhm2 |
| 10342437 | 0.05 | -0.32 | 0.75 | -0.24 | Unidentified |
| 10343730 | 0.72 | -0.46 | 0.75 | -0.33 | Unidentified |
| 10486112 | 0.18 | 0.27 | 0.75 | 0.34 | Bmf |
| 10343121 | 0.44 | -0.02 | 0.75 | 0.40 | Unidentified |
| 10340314 | 0.24 | -0.06 | 0.74 | 0.29 | Unidentified |
| 10443949 | -0.13 | 0.03 | 0.74 | 0.31 | Adamts10 |
| 10422493 | 0.04 | 0.42 | 0.74 | -0.40 | Gpr18 |
| 10343238 | -0.09 | 0.13 | 0.74 | 0.02 | Unidentified |
| 10564159 | 0.05 | -0.02 | 0.74 | 0.07 | Unidentified |
| 10502552 | 0.27 | -0.05 | 0.74 | -0.11 | Clca3a1 |
| 10340353 | 0.04 | 0.46 | 0.73 | 0.08 | Unidentified |
| 10538993 | -0.07 | 0.11 | 0.73 | -0.05 | Cd8a |
| 10371332 | -0.04 | -0.16 | 0.73 | 0.25 | Aldh1l2 |
| 10379996 | 0.15 | -0.21 | 0.73 | 0.00 | mir-130 |
| 10361887 | 0.24 | -0.11 | 0.72 | 0.41 | Perp |
| 10585286 | -0.07 | -0.17 | 0.72 | 0.08 | Arhgap20 |
| 10368806 | 0.30 | 0.11 | 0.72 | 0.42 | Smpd2 |
| 10578504 | -0.02 | -0.29 | 0.72 | 0.19 | 1700029J07Rik |
| 10462484 | -0.06 | -0.13 | 0.72 | 0.06 | A1cf |
| 10517003 | -0.11 | -0.07 | 0.72 | 0.01 | Unidentified |
| 10338807 | 0.06 | 0.02 | 0.72 | 0.06 | Unidentified |
| 10477450 | -0.22 | -0.04 | 0.72 | 0.03 | Bpifa2 |
| 10455078 | -0.04 | -0.13 | 0.72 | -0.09 | Pcdhb8 |
| 10342054 | -0.19 | 0.04 | 0.72 | 0.36 | Unidentified |
| 10448676 | -0.03 | -0.12 | 0.72 | -0.01 | Slc9a3r2 |
| 10399505 | 0.19 | 0.21 | 0.72 | 0.18 | Greb1 |
| 10340211 | 0.33 | 0.16 | 0.72 | 0.20 | Unidentified |
| 10588876 | -0.01 | -0.19 | 0.71 | -0.05 | Nicn1 |
| 10524460 | 0.11 | 0.06 | 0.71 | 0.24 | Acacb |
| 10425601 | 0.11 | 0.11 | 0.71 | 0.40 | Tef |
| 10427454 | 0.10 | 0.16 | 0.71 | 0.37 | Unidentified |
| 10414738 | -0.12 | 0.11 | 0.71 | 0.25 | Trav9d-1 |
| 10414934 | -0.12 | 0.11 | 0.71 | 0.25 | Trav9d-1 |
| 10338182 | 0.35 | -0.24 | 0.71 | -0.12 | Unidentified |
| 10344057 | 0.35 | 0.44 | 0.71 | 0.11 | Unidentified |
| 10434229 | 0.03 | 0.27 | 0.71 | 0.06 | Cldn5 |
| 10568332 | -0.05 | -0.12 | 0.70 | -0.10 | Prss8 |
| 10344066 | -0.09 | 0.15 | 0.70 | -0.32 | Unidentified |
| 10372110 | 0.04 | 0.12 | 0.70 | 0.10 | Phxr2 |
| 10344013 | 0.43 | -0.21 | 0.70 | 0.09 | Unidentified |
| 10385323 | -0.23 | 0.07 | 0.69 | -0.15 | mir-146 |
| 10570516 | -0.02 | -0.11 | 0.69 | 0.23 | Kbtbd11 |
| 10561748 | 0.05 | 0.02 | 0.69 | 0.20 | Catsperg1 |
| 10494023 | 0.19 | 0.03 | 0.69 | 0.22 | Rorc |
| 10439651 | 0.05 | 0.04 | 0.69 | -0.17 | Cd200 |
| 10340525 | 0.19 | -0.08 | 0.69 | 0.24 | Unidentified |
| 10405380 | 0.02 | 0.17 | 0.69 | 0.08 | Fgfr4 |
| 10400155 | 0.05 | -0.17 | 0.69 | 0.19 | Unidentified |
| 10342440 | -0.01 | -0.05 | 0.68 | 0.38 | Unidentified |
| 10499529 | 0.17 | 0.15 | 0.68 | 0.12 | Slc50a1 |
| 10552532 | 0.07 | -0.09 | 0.68 | -0.21 | Klk4 |
| 10589654 | 0.24 | 0.14 | 0.68 | 0.38 | Als2cl |
| 10487906 | 0.11 | 0.04 | 0.68 | 0.26 | Slc23a2 |
| 10375019 | -0.03 | -0.16 | 0.68 | 0.16 | Nsg2 |
| 10341224 | 0.13 | -0.11 | 0.68 | 0.13 | Unidentified |
| 10362861 | -0.03 | 0.08 | 0.68 | 0.24 | Scml4 |
| 10351353 | -0.05 | 0.35 | 0.68 | 0.25 | Cd247 |
| 10344118 | -0.15 | 0.15 | 0.68 | 0.20 | Unidentified |
| 10481103 | -0.10 | -0.10 | 0.68 | 0.22 | Lcn4 |
| 10586079 | 0.06 | -0.02 | 0.68 | 0.33 | Itga11 |
| 10342370 | 0.11 | 0.22 | 0.68 | -0.11 | Unidentified |
| 10340618 | 0.25 | 0.19 | 0.68 | 0.19 | Unidentified |
| 10343169 | 0.12 | 0.13 | 0.68 | 0.41 | Unidentified |
| 10421293 | -0.09 | -0.09 | 0.68 | 0.21 | Ppp3cc |
| 10595211 | 0.24 | 0.20 | 0.67 | 0.33 | Col12a1 |
| 10341404 | 0.12 | -0.09 | 0.67 | -0.07 | Unidentified |
| 10437272 | 0.09 | -0.05 | 0.67 | 0.32 | Nlrc3 |
| 10343858 | -0.48 | 0.26 | 0.67 | 0.00 | Unidentified |
| 10342149 | 0.06 | -0.06 | 0.67 | 0.17 | Unidentified |
| 10502052 | 0.25 | 0.01 | 0.67 | 0.42 | Alpk1 |
| 10565811 | 0.00 | 0.08 | 0.66 | 0.13 | Snord15b |
| 10538273 | -0.02 | -0.02 | 0.66 | 0.10 | BC022713 |
| 10598101 | 0.03 | 0.04 | 0.66 | -0.01 | Maml2 |
| 10342452 | 0.28 | -0.14 | 0.66 | -0.17 | Unidentified |
| 10600210 | 0.17 | 0.14 | 0.66 | 0.43 | Slc6a8 |
| 10416956 | 0.21 | 0.09 | 0.66 | 0.42 | mir-19 |
| 10569344 | 0.65 | 0.06 | 0.66 | 0.08 | Igf2 |
| 10571214 | 0.13 | -0.03 | 0.66 | -0.16 | Rnf122 |
| 10451679 | 0.14 | 0.09 | 0.66 | 0.14 | Daam2 |
| 10442495 | 0.02 | -0.05 | 0.66 | 0.24 | Pkd1 |
| 10535956 | 0.20 | 0.06 | 0.66 | 0.40 | Stard13 |
| 10414929 | 0.03 | 0.32 | 0.66 | 0.32 | Trav7d-4 |
| 10416023 | 0.08 | 0.28 | 0.65 | 0.33 | Scara5 |
| 10604956 | 0.02 | -0.03 | 0.65 | -0.06 | mir-452 |
| 10339901 | 0.20 | -0.01 | 0.65 | 0.27 | Unidentified |
| 10563760 | 0.01 | -0.19 | 0.65 | -0.19 | Mrgprb2 |
| 10591263 | -0.03 | -0.11 | 0.65 | 0.23 | Fbxl12 |
| 10484371 | 0.11 | 0.00 | 0.65 | 0.31 | Calcrl |
| 10339254 | 0.14 | 0.03 | 0.64 | 0.30 | Unidentified |
| 10362350 | -0.23 | 0.53 | 0.64 | 0.15 | Themis |
| 10446713 | 0.21 | 0.30 | 0.64 | 0.06 | Unidentified |
| 10492971 | 0.05 | 0.28 | 0.64 | 0.09 | Fcrl1 |
| 10439583 | 0.23 | -0.02 | 0.64 | 0.10 | Sidt1 |
| 10461777 | 0.11 | 0.06 | 0.64 | 0.34 | Olfr1444 |
| 10378549 | 0.05 | -0.14 | 0.63 | 0.10 | Rtn4rl1 |
| 10341930 | 0.02 | -0.16 | 0.63 | 0.03 | Unidentified |
| 10461384 | -0.20 | 0.05 | 0.63 | -0.01 | Unidentified |
| 10340302 | 0.18 | -0.02 | 0.63 | 0.25 | Unidentified |
| 10389047 | 0.02 | 0.00 | 0.63 | 0.32 | Asic2 |
| 10385635 | 0.22 | -0.13 | 0.63 | 0.28 | Zfp354c |
| 10413733 | 0.05 | 0.01 | 0.63 | 0.27 | Sema3g |
| 10344415 | 0.54 | -0.28 | 0.63 | 0.38 | Unidentified |
| 10502565 | 0.15 | -0.03 | 0.63 | 0.26 | Clca3a2 |
| 10338317 | 0.21 | -0.03 | 0.62 | 0.36 | Unidentified |
| 10385747 | 0.15 | 0.13 | 0.62 | 0.25 | Jade2 |
| 10340456 | 0.11 | 0.04 | 0.62 | 0.39 | Unidentified |
| 10548884 | 0.10 | 0.14 | 0.62 | 0.39 | Erp27 |
| 10569719 | 0.04 | 0.11 | 0.62 | 0.36 | A430078G23Rik |
| 10373542 | 0.01 | 0.22 | 0.62 | 0.20 | Dgka |
| 10542335 | 0.30 | 0.11 | 0.62 | 0.35 | Gprc5a |
| 10369264 | 0.11 | 0.01 | 0.61 | 0.40 | Oit3 |
| 10454807 | 0.12 | 0.01 | 0.61 | 0.35 | Snora74a |
| 10341297 | 0.19 | 0.26 | 0.61 | 0.19 | Unidentified |
| 10359713 | 0.11 | 0.13 | 0.61 | 0.24 | Sft2d2 |
| 10339155 | 0.09 | 0.02 | 0.61 | 0.23 | Unidentified |
| 10341294 | 0.06 | 0.27 | 0.60 | 0.25 | Unidentified |
| 10345089 | 0.20 | 0.03 | 0.60 | 0.02 | mir-30 |
| 10593918 | 0.04 | 0.13 | 0.60 | 0.28 | Ppcdc |
| 10360235 | 0.39 | -0.04 | 0.60 | -0.09 | Casq1 |
| 10515396 | 0.09 | 0.00 | 0.60 | 0.10 | Unidentified |
| 10340214 | 0.17 | 0.14 | 0.60 | 0.11 | Unidentified |
| 10528287 | 0.14 | -0.14 | 0.59 | 0.08 | Unidentified |
| 10436662 | 0.16 | 0.33 | 0.59 | 0.13 | Unidentified |
| 10589368 | 0.07 | 0.03 | 0.59 | 0.13 | Plxnb1 |
| 10499748 | 0.07 | 0.25 | 0.59 | 0.28 | Rps27 |
| 10357870 | 0.23 | -0.02 | 0.59 | 0.02 | Prelp |
| 10339852 | 0.35 | -0.14 | 0.59 | 0.21 | Unidentified |
| 10562649 | -0.03 | 0.02 | 0.59 | 0.11 | Unidentified |
| 10397145 | 0.33 | 0.03 | 0.59 | 0.12 | Acot2 |
| 10343702 | 0.10 | 0.18 | 0.59 | 0.22 | Unidentified |
| 10414714 | 0.14 | 0.10 | 0.59 | 0.23 | Unidentified |
| 10366746 | 0.16 | 0.05 | 0.58 | 0.37 | Lrig3 |
| 10341015 | 0.53 | -0.07 | 0.58 | 0.14 | Unidentified |
| 10371506 | 0.05 | 0.16 | 0.58 | 0.27 | Stab2 |
| 10339640 | 0.14 | 0.06 | 0.58 | 0.34 | Unidentified |
| 10463482 | -0.02 | 0.17 | 0.57 | 0.21 | Tlx1 |
| 10344537 | 0.06 | 0.15 | 0.57 | 0.32 | Unidentified |
| 10597575 | 0.09 | 0.16 | 0.57 | 0.27 | Plcd1 |
| 10343549 | 0.17 | 0.16 | 0.57 | 0.32 | Unidentified |
| 10446235 | 0.07 | 0.08 | 0.57 | 0.16 | Trip10 |
| 10346224 | 0.18 | 0.11 | 0.57 | 0.13 | Nemp2 |
| 10545458 | 0.14 | 0.04 | 0.56 | 0.24 | Tcf7l1 |
| 10484207 | 0.21 | 0.10 | 0.56 | 0.32 | Ccdc141 |
| 10344061 | 0.17 | 0.05 | 0.55 | 0.31 | Unidentified |

**Supplemental Table 9.** List of genes in cluster 20

|  | **Log ratio** | | | |  |
| --- | --- | --- | --- | --- | --- |
| **Probe Set ID** | **A.SW_Brain** | **SJL_Brain** | **A.SW_Spleen** | **SJL_Spleen** | **Symbol** |
| 10404264 | -3.57 | -3.25 | 0.19 | -0.23 | Prl |
| 10392135 | -2.00 | -1.83 | -0.05 | -0.17 | Gh |
| 10341506 | -1.77 | -1.03 | 0.15 | 0.62 | Unidentified |
| 10503947 | -1.58 | -1.20 | -0.04 | -0.14 | Cga |
| 10341208 | -1.33 | -0.94 | -0.26 | 0.08 | Unidentified |
| 10464905 | -1.25 | -1.30 | -0.25 | 0.64 | Npas4 |
| 10341469 | -1.20 | -0.87 | -0.27 | 0.97 | Unidentified |
| 10338264 | -1.17 | -0.92 | 0.05 | 0.73 | Unidentified |
| 10339664 | -1.15 | -2.11 | 0.10 | 0.31 | Unidentified |
| 10408557 | -1.09 | -1.04 | 0.17 | 0.96 | Serpinb1a |
| 10467529 | -1.06 | -0.72 | -0.10 | -0.08 | Opalin |
| 10338915 | -1.06 | -0.64 | 0.15 | 0.37 | Unidentified |
| 10343164 | -1.05 | -1.19 | 0.15 | -0.13 | Unidentified |
| 10344437 | -1.05 | -0.75 | 0.32 | 1.70 | Unidentified |
| 10342236 | -1.04 | -0.73 | 0.23 | 0.95 | Unidentified |
| 10339359 | -1.03 | -0.62 | 0.83 | 0.76 | Unidentified |
| 10344154 | -0.99 | -0.73 | 0.68 | 0.51 | Unidentified |
| 10362422 | -0.97 | -0.78 | -0.53 | 0.26 | Trdn |
| 10340723 | -0.97 | -0.87 | -0.18 | 0.10 | Unidentified |
| 10338669 | -0.96 | -1.21 | 0.00 | 0.30 | Unidentified |
| 10343464 | -0.96 | -0.63 | 0.37 | 0.66 | Unidentified |
| 10343703 | -0.96 | -0.58 | 0.12 | 0.36 | Unidentified |
| 10342678 | -0.95 | -1.04 | -0.29 | -0.04 | Unidentified |
| 10436476 | -0.93 | -0.74 | 0.08 | -0.20 | Pou1f1 |
| 10477536 | -0.92 | -1.84 | 0.33 | -0.25 | Bpifb9b |
| 10338386 | -0.88 | -0.60 | 0.47 | 0.42 | Unidentified |
| 10339758 | -0.87 | -0.71 | 0.07 | 0.26 | Unidentified |
| 10340787 | -0.86 | -0.86 | -0.25 | 0.13 | Unidentified |
| 10341088 | -0.83 | -0.78 | -0.40 | 0.64 | Unidentified |
| 10338795 | -0.77 | -1.26 | 0.27 | -0.04 | Unidentified |
| 10340735 | -0.77 | -1.13 | -0.17 | 0.53 | Unidentified |
| 10341882 | -0.75 | -1.08 | 0.66 | 0.84 | Unidentified |
| 10605482 | -0.74 | -1.80 | 0.11 | -0.12 | Gm14744 |
| 10342342 | -0.73 | -1.11 | -0.39 | 0.05 | Unidentified |
| 10438751 | -0.72 | -0.62 | 0.21 | 0.65 | Unidentified |
| 10429491 | -0.67 | -1.27 | 0.05 | -0.17 | Arc |
| 10343485 | -0.67 | -0.67 | -0.27 | 0.33 | Unidentified |
| 10338165 | -0.66 | -0.93 | 0.15 | 0.72 | Unidentified |
| 10517587 | -0.65 | -0.58 | 0.54 | 0.24 | Alpl |
| 10338579 | -0.65 | -0.67 | 0.40 | 0.31 | Unidentified |
| 10342203 | -0.64 | -0.72 | -0.45 | 0.40 | Unidentified |
| 10344548 | -0.63 | -0.94 | -0.11 | 0.76 | Unidentified |
| 10341498 | -0.63 | -0.86 | -0.29 | 0.15 | Unidentified |
| 10339884 | -0.62 | -0.46 | 0.39 | 0.44 | Unidentified |
| 10342235 | -0.59 | -1.17 | -0.08 | 0.24 | Unidentified |
| 10600568 | -0.58 | -1.86 | -0.05 | -0.15 | 5430402E10Rik |
| 10342707 | -0.58 | -0.60 | 0.19 | 0.25 | Unidentified |
| 10339260 | -0.58 | -0.89 | 0.43 | 0.01 | Unidentified |
| 10340722 | -0.56 | -0.62 | 0.02 | 0.19 | Unidentified |
| 10555935 | -0.55 | -0.80 | 0.14 | -0.03 | Cckbr |
| 10343108 | -0.54 | -0.89 | -0.19 | 0.54 | Unidentified |
| 10344579 | -0.54 | -0.62 | 0.44 | 0.47 | Unidentified |
| 10342343 | -0.53 | -0.68 | -0.29 | 0.21 | Unidentified |
| 10470175 | -0.53 | -1.52 | 0.10 | -0.28 | Obp2a |
| 10358533 | -0.53 | -1.32 | 0.46 | 0.50 | Hmcn1 |
| 10605488 | -0.52 | -1.40 | 0.02 | -0.23 | Obp1a |
| 10485357 | -0.51 | -0.97 | 0.80 | 0.91 | Unidentified |
| 10490302 | -0.50 | -0.70 | 0.10 | 0.50 | Zfp931 |
| 10343215 | -0.49 | -1.30 | -0.24 | 0.46 | Unidentified |
| 10342758 | -0.48 | -1.06 | -0.07 | 0.34 | Unidentified |
| 10358565 | -0.48 | -1.23 | 0.31 | 0.61 | Hmcn1 |
| 10358561 | -0.47 | -1.02 | 0.29 | 0.47 | Hmcn1 |
| 10342946 | -0.47 | -0.78 | -0.13 | 0.51 | Unidentified |
| 10358662 | -0.46 | -0.81 | 0.46 | 0.44 | Hmcn1 |
| 10351131 | -0.46 | -1.06 | 0.12 | -0.10 | Myoc |
| 10344177 | -0.46 | -0.83 | -0.19 | 0.38 | Unidentified |
| 10403911 | -0.45 | -1.11 | 0.11 | -0.15 | Gpx6 |
| 10454782 | -0.45 | -0.74 | 0.12 | 0.10 | Egr1 |
| 10358535 | -0.45 | -1.18 | 0.40 | 0.47 | Hmcn1 |
| 10340232 | -0.45 | -0.95 | -0.16 | 0.03 | Unidentified |
| 10427035 | -0.45 | -0.97 | 0.36 | 0.04 | Nr4a1 |
| 10342348 | -0.44 | -0.69 | -0.10 | 0.24 | Unidentified |
| 10340243 | -0.44 | -0.81 | 0.45 | 0.64 | Unidentified |
| 10338596 | -0.44 | -0.68 | -0.13 | 0.53 | Unidentified |
| 10340395 | -0.44 | -0.90 | 0.07 | -0.12 | Unidentified |
| 10342970 | -0.44 | -0.82 | 0.51 | 0.94 | Unidentified |
| 10340940 | -0.43 | -0.54 | 0.26 | 0.41 | Unidentified |
| 10358577 | -0.42 | -0.93 | 0.55 | 0.54 | Hmcn1 |
| 10358664 | -0.42 | -0.98 | 0.37 | 0.68 | Hmcn1 |
| 10551273 | -0.42 | -1.53 | 0.05 | -0.01 | Cyp2g1 |
| 10358575 | -0.41 | -0.74 | 0.52 | 0.64 | Hmcn1 |
| 10358587 | -0.41 | -1.02 | 0.38 | 0.81 | Hmcn1 |
| 10491993 | -0.41 | -1.11 | 0.11 | 0.11 | Stoml3 |
| 10341484 | -0.41 | -1.09 | -0.26 | 0.39 | Unidentified |
| 10342812 | -0.39 | -0.71 | 0.33 | 0.62 | Unidentified |
| 10340088 | -0.39 | -0.83 | 0.29 | 0.82 | Unidentified |
| 10358531 | -0.38 | -0.90 | 0.35 | 0.19 | Hmcn1 |
| 10358583 | -0.38 | -0.90 | 0.48 | 0.62 | Hmcn1 |
| 10340195 | -0.37 | -1.49 | -0.12 | 0.82 | Unidentified |
| 10358541 | -0.37 | -0.99 | 0.25 | 0.53 | Hmcn1 |
| 10343459 | -0.37 | -0.67 | 0.00 | 0.33 | Unidentified |
| 10358579 | -0.37 | -0.78 | 0.42 | 0.54 | Hmcn1 |
| 10358563 | -0.36 | -0.97 | 0.46 | 0.51 | Hmcn1 |
| 10358545 | -0.36 | -0.98 | 0.32 | 0.67 | Hmcn1 |
| 10477475 | -0.36 | -0.95 | 0.02 | -0.07 | Bpifa1 |
| 10358658 | -0.36 | -0.90 | 0.41 | 0.54 | Hmcn1 |
| 10358654 | -0.36 | -0.77 | 0.57 | 0.43 | Hmcn1 |
| 10358549 | -0.35 | -0.97 | 0.51 | 0.52 | Hmcn1 |
| 10358631 | -0.35 | -0.89 | 0.41 | 0.48 | Hmcn1 |
| 10398442 | -0.35 | -1.07 | 0.24 | 0.09 | Mirg |
| 10522902 | -0.35 | -1.56 | 0.07 | -0.17 | BC051076 |
| 10342617 | -0.35 | -0.81 | -0.11 | 0.36 | Unidentified |
| 10358670 | -0.35 | -1.07 | 0.47 | 0.50 | Hmcn1 |
| 10358581 | -0.35 | -0.95 | 0.52 | 0.50 | Hmcn1 |
| 10358539 | -0.34 | -1.00 | 0.41 | 0.48 | Hmcn1 |
| 10358652 | -0.34 | -0.85 | 0.59 | 0.61 | Hmcn1 |
| 10358633 | -0.34 | -1.12 | 0.52 | 0.61 | Hmcn1 |
| 10358656 | -0.34 | -0.99 | 0.46 | 0.48 | Hmcn1 |
| 10358666 | -0.34 | -0.84 | 0.51 | 0.44 | Hmcn1 |
| 10358615 | -0.33 | -0.74 | 0.53 | 0.52 | Hmcn1 |
| 10338451 | -0.33 | -1.38 | 0.11 | 0.10 | Unidentified |
| 10358553 | -0.32 | -0.70 | 0.37 | 0.57 | Hmcn1 |
| 10477528 | -0.32 | -1.17 | 0.26 | -0.19 | Bpifb9a |
| 10472923 | -0.32 | -0.77 | -0.11 | 0.48 | Ak4 |
| 10343139 | -0.32 | -0.80 | -0.25 | 0.42 | Unidentified |
| 10358527 | -0.32 | -0.90 | 0.42 | 0.48 | Hmcn1 |
| 10340252 | -0.31 | -0.70 | 0.00 | 0.31 | Unidentified |
| 10358621 | -0.31 | -0.69 | 0.53 | 0.44 | Hmcn1 |
| 10358637 | -0.31 | -0.86 | 0.57 | 0.42 | Hmcn1 |
| 10358593 | -0.31 | -0.71 | 0.58 | 0.53 | Hmcn1 |
| 10342713 | -0.31 | -0.89 | -0.11 | 0.16 | Unidentified |
| 10340894 | -0.31 | -0.85 | -0.10 | 0.20 | Unidentified |
| 10423049 | -0.30 | -0.94 | 0.92 | 0.70 | PRLR |
| 10358567 | -0.30 | -0.86 | 0.42 | 0.55 | Hmcn1 |
| 10358529 | -0.29 | -0.99 | 0.47 | 0.41 | Hmcn1 |
| 10358623 | -0.29 | -0.86 | 0.47 | 0.56 | Hmcn1 |
| 10358648 | -0.29 | -0.88 | 0.67 | 0.75 | Hmcn1 |
| 10339024 | -0.29 | -0.78 | 0.21 | 0.55 | Unidentified |
| 10358627 | -0.28 | -0.74 | 0.39 | 0.62 | Hmcn1 |
| 10551293 | -0.28 | -1.19 | 0.52 | 0.16 | CYP2F1 |
| 10340091 | -0.28 | -0.74 | -0.09 | 0.30 | Unidentified |
| 10339859 | -0.27 | -0.64 | -0.01 | 0.39 | Unidentified |
| 10358650 | -0.27 | -0.75 | 0.58 | 0.47 | Hmcn1 |
| 10341259 | -0.27 | -0.93 | 0.40 | 0.44 | Unidentified |
| 10358547 | -0.27 | -0.89 | 0.40 | 0.54 | Hmcn1 |
| 10340708 | -0.27 | -1.37 | 0.27 | 0.75 | Unidentified |
| 10358525 | -0.27 | -1.06 | 0.59 | 0.88 | Hmcn1 |
| 10358595 | -0.24 | -0.95 | 0.44 | 0.58 | Hmcn1 |
| 10350337 | -0.24 | -0.71 | 0.06 | 0.30 | A130050O07Rik |
| 10343452 | -0.23 | -1.52 | 0.32 | 0.28 | Unidentified |
| 10358555 | -0.23 | -0.70 | 0.56 | 0.52 | Hmcn1 |
| 10358515 | -0.23 | -0.88 | 0.79 | 0.79 | Hmcn1 |
| 10338699 | -0.23 | -1.94 | -0.38 | 2.00 | Unidentified |
| 10358543 | -0.23 | -0.78 | 0.29 | 0.52 | Hmcn1 |
| 10339835 | -0.23 | -1.51 | -0.36 | -0.12 | Unidentified |
| 10340771 | -0.23 | -0.86 | 0.27 | 0.08 | Unidentified |
| 10343936 | -0.22 | -0.86 | -0.12 | 0.32 | Unidentified |
| 10338692 | -0.22 | -0.73 | 0.07 | 0.32 | Unidentified |
| 10358660 | -0.22 | -1.12 | 0.50 | 0.70 | Hmcn1 |
| 10551226 | -0.21 | -1.33 | -0.03 | -0.10 | CYP2A6 |
| 10339476 | -0.21 | -0.90 | 0.61 | 0.57 | Unidentified |
| 10338097 | -0.21 | -1.45 | 0.65 | 0.93 | Unidentified |
| 10358571 | -0.21 | -0.68 | 0.44 | 0.56 | Hmcn1 |
| 10358668 | -0.21 | -0.99 | 0.51 | 0.58 | Hmcn1 |
| 10341425 | -0.18 | -1.06 | -0.02 | -0.06 | Unidentified |
| 10339583 | -0.18 | -0.97 | 0.01 | 0.61 | Unidentified |
| 10343943 | -0.18 | -0.87 | 0.10 | 0.29 | Unidentified |
| 10358619 | -0.18 | -0.71 | 0.55 | 0.52 | Hmcn1 |
| 10338781 | -0.18 | -0.82 | -0.14 | 0.77 | Unidentified |
| 10341589 | -0.17 | -0.87 | 0.07 | 0.60 | Unidentified |
| 10339918 | -0.15 | -1.19 | 0.29 | 0.50 | Unidentified |
| 10600576 | -0.14 | -1.77 | -0.03 | -0.02 | Gm14743 |
| 10340623 | -0.13 | -0.83 | 0.16 | 0.37 | Unidentified |
| 10338343 | -0.13 | -1.23 | -0.02 | 0.31 | Unidentified |
| 10338729 | -0.12 | -0.71 | 0.34 | 0.46 | Unidentified |
| 10338855 | -0.11 | -0.82 | -0.15 | 0.60 | Unidentified |
| 10342956 | -0.10 | -0.77 | 0.02 | 0.50 | Unidentified |
| 10341406 | -0.10 | -0.92 | 0.58 | 0.46 | Unidentified |
| 10343582 | -0.10 | -1.66 | 0.26 | 0.19 | Unidentified |
| 10341042 | -0.08 | -0.74 | 0.07 | 0.48 | Unidentified |
| 10342441 | -0.08 | -0.80 | 0.37 | 0.62 | Unidentified |
| 10339677 | -0.07 | -0.96 | 0.37 | 0.19 | Unidentified |
| 10469577 | -0.06 | -1.01 | 0.56 | 0.15 | Gm16495 |
| 10339208 | -0.06 | -0.88 | 0.09 | 0.72 | Unidentified |
| 10341870 | 0.00 | -0.90 | 0.40 | 0.56 | Unidentified |
| 10340868 | 0.00 | -1.18 | 0.12 | 0.66 | Unidentified |
| 10340415 | 0.00 | -0.88 | 0.27 | 0.89 | Unidentified |
| 10358597 | 0.01 | -0.88 | 0.41 | 0.84 | Hmcn1 |
| 10341756 | 0.03 | -0.92 | 0.48 | 0.85 | Unidentified |
| 10344482 | 0.15 | -1.30 | 1.27 | 0.71 | Unidentified |
| 10338130 | 0.28 | -1.09 | 0.21 | 0.70 | Unidentified |
| 10339757 | 0.30 | -1.22 | 0.20 | 1.37 | Unidentified |

**Supplemental Table 10.** List of genes in cluster 22

|  | **Log ratio** | | | |  |
| --- | --- | --- | --- | --- | --- |
| **Probe Set ID** | **A.SW_Brain** | **SJL_Brain** | **A.SW_Spleen** | **SJL_Spleen** | **Symbol** |
| 10544383 | -0.07 | -0.05 | -3.96 | -4.77 | Kel |
| 10509002 | 0.02 | -0.20 | -3.92 | -4.76 | Rhd |
| 10573054 | -0.27 | 0.05 | -3.84 | -4.82 | Gypa |
| 10545096 | -0.14 | -0.54 | -3.77 | -2.76 | Mageb16 |
| 10351905 | 0.05 | 0.03 | -3.73 | -3.50 | Spta1 |
| 10497122 | 0.19 | 0.08 | -3.70 | -0.99 | Depdc1a |
| 10339519 | 0.39 | -0.30 | -3.67 | -2.88 | Unidentified |
| 10512757 | 0.00 | 0.09 | -3.65 | -3.81 | Hemgn |
| 10534389 | 0.04 | -0.02 | -3.65 | -4.44 | Cldn13 |
| 10445192 | 0.05 | -0.05 | -3.63 | -4.02 | Rhag |
| 10423971 | -0.15 | -0.13 | -3.60 | -3.51 | Pkhd1l1 |
| 10445046 | -0.03 | -0.09 | -3.59 | -3.62 | Trim10 |
| 10385248 | 0.37 | 0.19 | -3.58 | -1.58 | Hmmr |
| 10563883 | 0.18 | -0.01 | -3.54 | -0.93 | Depdc1a |
| 10486664 | -0.19 | 0.03 | -3.51 | -4.15 | Epb42 |
| 10420877 | 0.04 | -0.04 | -3.47 | -1.64 | Esco2 |
| 10501802 | -0.22 | -0.53 | -3.47 | -3.99 | Tmem56 |
| 10462796 | 0.07 | 0.23 | -3.46 | -1.29 | Kif11 |
| 10605542 | -0.17 | -0.48 | -3.44 | -2.47 | Mageb16 |
| 10400483 | 0.14 | -0.27 | -3.40 | -3.19 | Slc25a21 |
| 10608710 | -0.20 | -0.20 | -3.39 | -4.81 | Unidentified |
| 10593198 | -0.14 | -0.02 | -3.38 | -4.23 | Nxpe2 |
| 10359890 | 0.09 | 0.16 | -3.34 | -1.05 | Nuf2 |
| 10570894 | -0.31 | -0.19 | -3.32 | -3.77 | Ank1 |
| 10340874 | -0.07 | 0.20 | -3.29 | -2.44 | Unidentified |
| 10521731 | 0.05 | 0.00 | -3.29 | -1.06 | Ncapg |
| 10401068 | -0.11 | -0.28 | -3.28 | -2.52 | Sptb |
| 10578690 | -0.09 | 0.20 | -3.28 | -1.06 | Neil3 |
| 10379026 | 0.03 | -0.20 | -3.28 | -2.96 | mir-144 |
| 10339167 | 0.24 | -0.28 | -3.26 | -1.16 | Unidentified |
| 10366446 | 0.25 | 0.12 | -3.24 | -3.60 | Tspan8 |
| 10499062 | -0.27 | -0.29 | -3.24 | -3.56 | Fhdc1 |
| 10414315 | -0.32 | 0.11 | -3.21 | -1.02 | Cdkn3 |
| 10487480 | 0.19 | 0.58 | -3.21 | -1.32 | Bub1 |
| 10344460 | 0.16 | -0.02 | -3.20 | -2.51 | Unidentified |
| 10400589 | 0.20 | 0.26 | -3.19 | -1.09 | Mis18bp1 |
| 10340845 | -0.64 | -0.46 | -3.18 | -1.47 | Unidentified |
| 10338143 | -0.45 | -0.81 | -3.18 | -2.39 | Unidentified |
| 10496204 | 0.25 | 0.16 | -3.18 | -1.03 | Cenpe |
| 10562637 | 0.12 | 0.36 | -3.18 | -1.28 | Ccnb1 |
| 10515836 | 0.19 | 0.44 | -3.15 | -1.25 | Ccnb1 |
| 10474984 | 0.24 | 0.65 | -3.14 | -1.66 | Nusap1 |
| 10554445 | 0.11 | 0.33 | -3.12 | -1.53 | Prc1 |
| 10404063 | 0.09 | -0.12 | -3.12 | -1.45 | Hist1h2ab |
| 10390707 | -0.02 | 0.51 | -3.11 | -1.15 | Top2a |
| 10344001 | -0.29 | 0.07 | -3.11 | -1.46 | Unidentified |
| 10391649 | -0.10 | -0.05 | -3.11 | -4.23 | Slc4a1 |
| 10515848 | 0.08 | 0.05 | -3.10 | -3.27 | Ermap |
| 10473349 | -0.12 | -0.13 | -3.10 | -3.08 | Ypel4 |
| 10338428 | 0.05 | 0.15 | -3.09 | -2.15 | Unidentified |
| 10360985 | -0.12 | 0.05 | -3.09 | -1.36 | Cenpf |
| 10339958 | -0.27 | -0.28 | -3.08 | -1.11 | Unidentified |
| 10411739 | 0.15 | 0.37 | -3.08 | -1.31 | Ccnb1 |
| 10591781 | 0.07 | 0.18 | -3.05 | -1.33 | Anln |
| 10503264 | -0.24 | -0.17 | -3.05 | -1.80 | Ccne2 |
| 10341811 | 0.26 | 0.71 | -3.05 | -2.77 | Unidentified |
| 10350392 | 0.11 | 0.22 | -3.04 | -1.31 | Aspm |
| 10350297 | -0.06 | -0.03 | -3.04 | -1.78 | Kif14 |
| 10563780 | -0.05 | 0.18 | -3.04 | -1.57 | E2f8 |
| 10504692 | -0.05 | -0.09 | -3.04 | -3.10 | Tmod1 |
| 10399710 | 0.26 | 1.00 | -3.03 | -2.93 | Rsad2 |
| 10406968 | -0.02 | 0.18 | -3.03 | -1.55 | Cenpk |
| 10451670 | -0.19 | 0.02 | -3.03 | -3.33 | Tspo2 |
| 10568714 | 0.31 | 0.95 | -3.02 | -1.41 | Mki67 |
| 10343964 | 0.37 | -0.77 | -3.02 | -2.39 | Unidentified |
| 10342665 | 0.02 | 0.16 | -3.01 | -2.61 | Unidentified |
| 10369815 | 0.00 | 0.36 | -3.01 | -1.08 | Cdk1 |
| 10394978 | 0.11 | 0.49 | -2.99 | -1.80 | Rrm2 |
| 10344100 | -0.02 | 0.57 | -2.98 | -2.28 | Unidentified |
| 10340679 | 0.26 | -0.57 | -2.95 | -2.49 | Unidentified |
| 10338454 | -0.57 | 0.11 | -2.95 | -2.35 | Unidentified |
| 10594774 | 0.00 | 0.22 | -2.95 | -1.24 | Ccnb2 |
| 10376434 | -0.03 | -0.04 | -2.94 | -3.00 | Btnl10 |
| 10507112 | 0.03 | 0.10 | -2.93 | -0.93 | Stil |
| 10497520 | 0.24 | 0.02 | -2.92 | -1.28 | Ect2 |
| 10342791 | -0.14 | -0.36 | -2.92 | -2.45 | Unidentified |
| 10573457 | -0.01 | -0.18 | -2.91 | -3.12 | Klf1 |
| 10474875 | -0.01 | 0.56 | -2.91 | -1.27 | Casc5 |
| 10342017 | 0.08 | -0.10 | -2.90 | -2.31 | Unidentified |
| 10340943 | 0.06 | -0.14 | -2.90 | -2.55 | Unidentified |
| 10371591 | 0.15 | 0.10 | -2.66 | -1.18 | Parpbp |
| 10382998 | 0.03 | 0.16 | -2.65 | -1.26 | Birc5 |
| 10338377 | 0.11 | 0.24 | -2.65 | -2.63 | Unidentified |
| 10339273 | 0.31 | -0.17 | -2.65 | -1.52 | Unidentified |
| 10339567 | 0.20 | 0.28 | -2.64 | -2.38 | Unidentified |
| 10421877 | -0.14 | 0.15 | -2.64 | -1.30 | Diaph3 |
| 10404069 | 0.11 | 0.05 | -2.64 | -1.73 | Hist1h1a |
| 10342241 | 0.09 | 0.33 | -2.64 | -2.40 | Unidentified |
| 10384373 | 0.10 | -0.05 | -2.63 | -1.27 | Fignl1 |
| 10343957 | -0.23 | 0.36 | -2.62 | -2.36 | Unidentified |
| 10338617 | -0.31 | -0.54 | -2.61 | -2.29 | Unidentified |
| 10339087 | -0.34 | -0.58 | -2.61 | -2.35 | Unidentified |
| 10338896 | 0.29 | -0.03 | -2.60 | -2.13 | Unidentified |
| 10342237 | 0.09 | -0.17 | -2.59 | -2.12 | Unidentified |
| 10409190 | -0.14 | 0.00 | -2.58 | -1.14 | Cenpp |
| 10342410 | 0.17 | -0.40 | -2.58 | -2.29 | Unidentified |
| 10567108 | -0.08 | -0.12 | -2.58 | -2.40 | Sox6 |
| 10341386 | 0.45 | 0.01 | -2.57 | -2.48 | Unidentified |
| 10341388 | 0.23 | 0.04 | -2.57 | -2.59 | Unidentified |
| 10598507 | -0.18 | 0.44 | -2.56 | -2.90 | Slc38a5 |
| 10490923 | -0.51 | -0.58 | -2.55 | -3.49 | Car2 |
| 10376396 | -0.01 | 0.13 | -2.55 | -1.55 | Trim58 |
| 10343453 | -0.18 | -0.44 | -2.55 | -1.99 | Unidentified |
| 10341692 | -0.11 | 0.26 | -2.54 | -2.48 | Unidentified |
| 10399087 | -0.03 | -0.17 | -2.54 | -1.21 | Ncapg2 |
| 10339230 | -0.13 | -0.17 | -2.54 | -2.55 | Unidentified |
| 10341878 | -0.42 | 0.34 | -2.54 | -1.27 | Unidentified |
| 10519578 | 0.02 | -0.09 | -2.53 | -1.70 | Abcb4 |
| 10339594 | 0.33 | -0.34 | -2.53 | -2.17 | Unidentified |
| 10539669 | -0.09 | -0.10 | -2.53 | -2.51 | Add2 |
| 10582626 | -0.17 | -0.15 | -2.52 | -2.54 | Abcb10 |
| 10509168 | -0.07 | 0.02 | -2.52 | -1.68 | E2f2 |
| 10389300 | -0.01 | -0.08 | -2.51 | -2.72 | Dhrs11 |
| 10344233 | 0.26 | 0.14 | -2.51 | -2.17 | Unidentified |
| 10341667 | 0.40 | -0.12 | -2.51 | -2.36 | Unidentified |
| 10342380 | 0.03 | -0.37 | -2.51 | -2.36 | Unidentified |
| 10339645 | -0.71 | -0.08 | -2.51 | -2.06 | Unidentified |
| 10493382 | -0.03 | -0.05 | -2.49 | -2.48 | Pklr |
| 10536908 | -0.10 | -0.06 | -2.48 | -2.72 | Tspan33 |
| 10341292 | -0.13 | -0.48 | -2.48 | -1.53 | Unidentified |
| 10339089 | -0.06 | -0.10 | -2.48 | -2.70 | Unidentified |
| 10385893 | 0.58 | 0.52 | -2.47 | -1.60 | Slc22a4 |
| 10501402 | 0.00 | -0.11 | -2.46 | -1.35 | Gpsm2 |
| 10342251 | 0.11 | -0.02 | -2.46 | -1.56 | Unidentified |
| 10394990 | 0.00 | 0.15 | -2.46 | -1.84 | Mboat2 |
| 10562563 | -0.14 | -0.14 | -2.46 | -2.10 | Ccne1 |
| 10341384 | -0.26 | -0.29 | -2.45 | -2.03 | Unidentified |
| 10545086 | -0.30 | -0.29 | -2.45 | -1.85 | Snca |
| 10341823 | -0.07 | -0.40 | -2.45 | -2.18 | Unidentified |
| 10339296 | 0.06 | -0.28 | -2.44 | -2.42 | Unidentified |
| 10343937 | 0.30 | -0.46 | -2.43 | -1.78 | Unidentified |
| 10342981 | -0.07 | -0.62 | -2.42 | -2.17 | Unidentified |
| 10340688 | 0.08 | -0.50 | -2.42 | -2.04 | Unidentified |
| 10343840 | -0.34 | -0.10 | -2.41 | -2.00 | Unidentified |
| 10343538 | 0.11 | 0.01 | -2.41 | -2.28 | Unidentified |
| 10376813 | -0.16 | -0.07 | -2.41 | -2.02 | Specc1 |
| 10596166 | 0.00 | 0.01 | -2.41 | -2.92 | 1300017J02Rik |
| 10344372 | -0.53 | -0.31 | -2.39 | -1.89 | Unidentified |
| 10343768 | -0.08 | -0.97 | -2.39 | -2.69 | Unidentified |
| 10528915 | -0.17 | -0.16 | -2.39 | -1.44 | Tyms |
| 10481304 | 0.01 | 0.16 | -2.38 | -1.46 | Gfi1b |
| 10339152 | -0.24 | 0.02 | -2.38 | -1.80 | Unidentified |
| 10343106 | -0.07 | 0.18 | -2.38 | -2.16 | Unidentified |
| 10342331 | -0.52 | -0.15 | -2.37 | -1.90 | Unidentified |
| 10344016 | -0.13 | -0.33 | -2.37 | -1.99 | Unidentified |
| 10538459 | 0.14 | 0.14 | -2.36 | -2.79 | Aqp1 |
| 10338772 | -0.22 | 0.06 | -2.36 | -2.32 | Unidentified |
| 10602372 | -0.27 | -0.34 | -2.35 | -2.90 | Alas2 |
| 10603417 | 0.03 | -0.09 | -2.35 | -1.32 | Gata1 |
| 10343851 | 0.03 | 0.46 | -2.35 | -2.03 | Unidentified |
| 10404061 | -0.34 | -0.20 | -2.34 | -1.58 | Hist1h2bb |
| 10340741 | 0.28 | 0.36 | -2.34 | -1.61 | Unidentified |
| 10340029 | -0.16 | -0.17 | -2.34 | -2.07 | Unidentified |
| 10592772 | -0.16 | -0.25 | -2.33 | -2.58 | Abcg4 |
| 10339563 | 0.03 | 0.11 | -2.32 | -2.32 | Unidentified |
| 10340116 | 0.00 | -0.14 | -2.32 | -2.01 | Unidentified |
| 10522655 | -0.28 | -0.52 | -2.32 | -1.46 | C530008M17Rik |
| 10343283 | 0.10 | 0.11 | -2.30 | -1.87 | Unidentified |
| 10598586 | -0.24 | -0.33 | -2.30 | -1.77 | Xk |
| 10375055 | 0.07 | -0.07 | -2.28 | -1.79 | Hbq1b |
| 10339224 | -0.10 | 0.11 | -2.09 | -2.22 | Unidentified |
| 10339174 | 0.12 | -0.17 | -2.08 | -2.01 | Unidentified |
| 10343514 | -0.15 | 0.18 | -2.07 | -1.97 | Unidentified |
| 10339372 | -0.20 | -0.09 | -2.07 | -2.00 | Unidentified |
| 10515385 | -0.12 | -0.13 | -2.06 | -1.94 | Urod |
| 10339875 | -0.15 | -0.29 | -2.06 | -1.76 | Unidentified |
| 10338686 | -0.29 | -0.27 | -2.06 | -1.83 | Unidentified |
| 10339381 | -0.10 | -0.28 | -2.06 | -1.91 | Unidentified |
| 10344572 | -0.09 | -0.16 | -2.06 | -1.82 | Unidentified |
| 10339740 | 0.09 | -0.12 | -2.05 | -2.05 | Unidentified |
| 10339747 | -0.09 | -0.01 | -2.05 | -1.65 | Unidentified |
| 10339885 | 0.03 | -0.19 | -2.05 | -2.17 | Unidentified |
| 10398085 | -0.13 | -0.01 | -2.05 | -1.88 | Glrx5 |
| 10541049 | -0.04 | -0.12 | -2.04 | -1.77 | March8 |
| 10338234 | 0.15 | -0.17 | -2.04 | -1.67 | Unidentified |
| 10338503 | -0.20 | 0.13 | -2.04 | -1.73 | Unidentified |
| 10339146 | 0.09 | 0.14 | -2.02 | -1.77 | Unidentified |
| 10341495 | 0.07 | -0.13 | -2.02 | -1.69 | Unidentified |
| 10582545 | 2.34 | 0.05 | -2.02 | -4.12 | Mela |
| 10539080 | -0.03 | -0.04 | -2.02 | -1.81 | St3gal5 |
| 10558811 | -0.19 | -0.21 | -2.02 | -2.23 | Ptdss2 |
| 10343818 | -0.03 | -0.20 | -2.02 | -1.82 | Unidentified |
| 10343345 | 0.03 | -0.19 | -2.01 | -1.98 | Unidentified |
| 10357698 | -0.22 | -0.25 | -2.00 | -2.28 | Tmcc2 |
| 10457780 | -0.05 | -0.14 | -2.00 | -1.85 | Garem |
| 10338649 | 0.10 | -0.79 | -2.00 | -1.90 | Unidentified |
| 10344422 | -0.05 | -0.08 | -1.98 | -1.94 | Unidentified |
| 10401149 | -0.04 | -0.06 | -1.98 | -1.80 | Plek2 |
| 10357249 | 0.16 | 0.11 | -1.97 | -1.92 | Steap3 |
| 10340720 | -0.09 | -0.05 | -1.97 | -2.05 | Unidentified |
| 10568638 | -0.21 | -0.32 | -1.97 | -1.81 | Uros |
| 10436392 | -0.14 | -0.18 | -1.97 | -2.87 | Cpox |
| 10344286 | 0.55 | 0.55 | -1.97 | -1.86 | Unidentified |
| 10338760 | -0.04 | -0.05 | -1.97 | -1.96 | Unidentified |
| 10342031 | -0.20 | -0.50 | -1.96 | -1.58 | Unidentified |
| 10338235 | -0.05 | -0.50 | -1.96 | -1.70 | Unidentified |
| 10581340 | 0.02 | -0.09 | -1.96 | -1.82 | Ranbp10 |
| 10342215 | -0.04 | -0.24 | -1.95 | -1.91 | Unidentified |
| 10343129 | 0.28 | 0.20 | -1.95 | -1.73 | Unidentified |
| 10341062 | -0.17 | -0.19 | -1.94 | -2.06 | Unidentified |
| 10343328 | -0.06 | -0.30 | -1.94 | -1.98 | Unidentified |
| 10421456 | -0.05 | -0.15 | -1.94 | -2.07 | Xpo7 |
| 10554240 | 0.05 | 0.70 | -1.92 | -2.19 | Isg20 |
| 10338506 | 0.13 | -0.14 | -1.91 | -1.67 | Unidentified |
| 10339249 | 0.08 | -0.06 | -1.91 | -1.96 | Unidentified |
| 10407955 | -0.15 | -0.28 | -1.90 | -1.81 | Epdr1 |
| 10495035 | 0.28 | -0.03 | -1.89 | -1.68 | Slc16a1 |
| 10573483 | -0.18 | -0.11 | -1.88 | -1.86 | Prdx2 |
| 10594825 | -0.03 | -0.09 | -1.87 | -1.76 | Aqp9 |
| 10377319 | -0.24 | -0.23 | -1.86 | -1.68 | Myh10 |
| 10340920 | -0.03 | -0.08 | -1.86 | -1.99 | Unidentified |
| 10342786 | -0.01 | -0.74 | -1.86 | -2.07 | Unidentified |
| 10472933 | -0.15 | -0.38 | -1.86 | -1.65 | Scrn3 |
| 10341552 | -0.22 | -0.10 | -1.85 | -1.72 | Unidentified |
| 10341538 | -0.11 | 0.21 | -1.84 | -1.95 | Unidentified |
| 10343868 | 0.38 | -0.28 | -1.83 | -2.08 | Unidentified |
| 10507500 | -0.27 | -0.30 | -1.83 | -2.70 | Slc6a9 |
| 10513608 | -0.12 | -0.14 | -1.83 | -2.16 | Alad |
| 10356999 | -0.11 | -0.09 | -1.82 | -2.22 | Prdx2 |
| 10341023 | 0.22 | -0.01 | -1.81 | -2.05 | Unidentified |
| 10338577 | 0.31 | 0.00 | -1.80 | -2.44 | Unidentified |
| 10368720 | 0.13 | 0.23 | -1.80 | -1.85 | Slc16a10 |
| 10449163 | -0.31 | -0.21 | -1.79 | -1.87 | Pigq |
| 10338101 | 0.44 | 0.16 | -1.79 | -2.05 | Unidentified |
| 10339823 | -0.03 | -0.14 | -1.79 | -1.86 | Unidentified |
| 10341847 | -0.18 | -0.05 | -1.77 | -1.95 | Unidentified |
| 10588707 | 0.00 | -0.11 | -1.76 | -1.88 | Ifrd2 |
| 10421172 | -0.25 | 0.00 | -1.74 | -2.08 | Slc25a37 |
| 10339308 | -0.08 | 0.03 | -1.71 | -1.90 | Unidentified |
| 10344117 | 0.31 | 0.14 | -1.70 | -1.80 | Unidentified |
| 10344207 | 0.26 | -0.07 | -1.70 | -2.47 | Unidentified |
| 10341260 | 0.68 | 0.00 | -1.68 | -2.13 | Unidentified |
| 10597960 | 0.79 | 0.51 | -1.68 | -2.08 | Slc6a20a |
| 10339904 | 0.53 | 0.32 | -1.66 | -1.84 | Unidentified |
| 10344481 | -0.09 | 0.40 | -1.63 | -1.88 | Unidentified |
| 10563852 | -0.20 | -0.28 | -1.63 | -1.91 | Nipa1 |
| 10551347 | 0.09 | 0.34 | -1.59 | -2.04 | Blvrb |
| 10587871 | -0.10 | -0.14 | -1.49 | -2.03 | Paqr9 (mPRε) |
| 10582549 | 0.73 | -0.04 | -1.44 | -2.58 | Mela |
| 10490913 | -0.06 | -0.66 | -1.09 | -2.29 | Car3 |

**Supplemental Table 11.** List of genes in cluster 23

|  | **Log ratio** | | | |  |
| --- | --- | --- | --- | --- | --- |
| **Probe Set ID** | **A.SW_Brain** | **SJL_Brain** | **A.SW_Spleen** | **SJL_Spleen** | **Symbol** |
| 10576757 | 0.03 | 0.07 | -0.41 | -2.00 | Fcer2a |
| 10535904 | -0.46 | -0.50 | -0.81 | -1.75 | Hsph1 |
| 10450926 | -0.06 | 0.12 | -0.11 | -1.70 | Crisp3 |
| 10341668 | -0.18 | -0.31 | -0.13 | -1.69 | Unidentified |
| 10495562 | 0.02 | -0.14 | -0.69 | -1.59 | Lrrc39 |
| 10387505 | 0.17 | 0.11 | -0.76 | -1.43 | Atp1b2 |
| 10552743 | -0.01 | 0.03 | -0.47 | -1.41 | Il4i1 |
| 10561055 | 0.37 | 0.12 | -0.29 | -1.37 | Ceacam2 |
| 10458992 | -0.42 | -0.33 | -0.87 | -1.31 | C330018D20Rik |
| 10498018 | -0.32 | -0.75 | -0.89 | -1.24 | Pcdh18 |
| 10441422 | -0.10 | -0.23 | -0.77 | -1.20 | Zdhhc14 |
| 10442643 | -0.17 | -0.30 | -0.74 | -1.20 | Nme3 |
| 10364444 | 0.02 | 0.12 | -0.57 | -1.16 | Madcam1 |
| 10350742 | 0.08 | 0.44 | -0.31 | -1.13 | Rnasel |
| 10389797 | -0.18 | -0.35 | -0.59 | -1.13 | Stxbp4 |
| 10389795 | -0.13 | -0.45 | -0.85 | -1.12 | Stxbp4 |
| 10373515 | 0.14 | 0.03 | -0.80 | -1.12 | Suox |
| 10440099 | 0.01 | -0.02 | -0.66 | -1.11 | St3gal6 |
| 10569646 | -0.02 | 0.12 | -0.68 | -1.10 | Ccnd1 |
| 10473444 | -0.17 | -0.30 | -0.87 | -1.10 | Aplnr |
| 10393936 | 0.12 | -0.69 | -0.76 | -1.08 | Cbr2 |
| 10375463 | -0.09 | 0.35 | -0.58 | -1.08 | Havcr1 |
| 10343269 | -0.74 | 0.41 | -0.17 | -1.08 | Unidentified |
| 10555460 | -0.15 | -0.12 | -0.95 | -1.06 | Stard10 |
| 10340240 | 0.01 | -0.54 | -0.48 | -1.05 | Unidentified |
| 10347564 | -0.17 | -0.37 | -0.93 | -1.04 | Dnajb2 |
| 10447521 | -0.40 | -0.62 | -0.84 | -1.04 | Tfb1m |
| 10504690 | -0.10 | -0.13 | -0.83 | -1.04 | Unidentified |
| 10339860 | 0.08 | -0.53 | -0.41 | -1.03 | Unidentified |
| 10340700 | 0.03 | 0.27 | -0.98 | -1.03 | Unidentified |
| 10463486 | -0.04 | -0.09 | -1.03 | -1.03 | Btrc |
| 10548875 | -0.15 | 0.01 | -0.96 | -1.02 | Art4 |
| 10424140 | 0.09 | -0.11 | -0.75 | -1.00 | Col14a1 |
| 10585428 | -0.22 | -0.12 | -1.05 | -0.99 | Dnaja4 |
| 10429520 | 0.14 | 0.41 | -0.37 | -0.99 | Ly6d |
| 10566254 | -0.48 | -0.59 | -0.73 | -0.98 | Hbb-b1 |
| 10566767 | -0.05 | -0.07 | -0.52 | -0.97 | St5 |
| 10374727 | 0.02 | -0.17 | -0.32 | -0.97 | Bcl11a |
| 10496971 | 0.07 | -0.01 | -0.60 | -0.96 | Asb17 |
| 10368356 | -0.09 | -0.15 | -1.08 | -0.96 | Akap7 |
| 10566258 | -0.53 | -0.48 | -0.74 | -0.95 | Hbb-b1 |
| 10438530 | 0.04 | -0.08 | -0.86 | -0.95 | Clcn2 |
| 10594053 | -0.06 | 0.13 | -0.51 | -0.95 | Pml |
| 10389590 | -0.07 | -0.18 | -0.98 | -0.95 | Gdpd1 |
| 10549647 | -0.07 | -0.04 | -0.71 | -0.94 | Ncr1 |
| 10580349 | 0.08 | -0.17 | -0.74 | -0.94 | Mylk3 |
| 10338916 | -0.51 | -0.38 | -0.57 | -0.94 | Unidentified |
| 10424607 | -0.14 | -0.06 | -0.33 | -0.94 | Ptp4a3 |
| 10343861 | 0.37 | -0.09 | -0.53 | -0.93 | Unidentified |
| 10421917 | -0.10 | -0.08 | -1.11 | -0.93 | Pabpc4 |
| 10354732 | -0.16 | -0.31 | -0.96 | -0.93 | Hspd1 |
| 10545672 | -0.07 | 0.05 | -0.82 | -0.93 | Mthfd2 |
| 10506603 | -0.07 | -0.08 | -0.91 | -0.93 | Ssbp3 |
| 10372130 | 0.01 | -0.27 | -1.10 | -0.93 | 4930430F08Rik |
| 10596815 | -0.15 | -0.14 | -0.85 | -0.93 | Rnf123 |
| 10409424 | -0.09 | -0.08 | -0.85 | -0.92 | Mxd3 |
| 10389929 | -0.44 | -0.28 | -1.02 | -0.92 | Cacna1g |
| 10369932 | -0.19 | -0.29 | -0.56 | -0.92 | Susd2 |
| 10512226 | 0.00 | -0.09 | -0.96 | -0.92 | Dcaf12 |
| 10491883 | -0.03 | -0.02 | -1.09 | -0.91 | Acp1 |
| 10375062 | 0.25 | 0.08 | -0.83 | -0.91 | Hbq1a |
| 10342889 | 0.53 | 0.63 | -0.98 | -0.91 | Unidentified |
| 10481474 | -0.02 | -0.07 | -0.70 | -0.91 | Crat |
| 10423363 | 0.07 | -0.09 | -0.72 | -0.91 | Ank |
| 10368534 | -0.12 | -0.22 | -1.08 | -0.91 | Ncoa7 |
| 10341380 | 0.04 | -0.41 | -0.43 | -0.90 | Unidentified |
| 10409978 | -0.15 | -0.25 | -0.99 | -0.90 | Zfp808 |
| 10460253 | 0.17 | -0.09 | -0.96 | -0.89 | Aldh3b3 |
| 10432573 | 0.07 | 0.02 | -0.89 | -0.89 | Slc11a2 |
| 10539435 | -0.01 | -0.08 | -0.97 | -0.89 | Bola3 |
| 10535841 | 0.05 | -0.01 | -0.29 | -0.89 | Slc46a3 |
| 10386909 | -0.25 | -0.38 | -1.03 | -0.89 | Cenpv |
| 10344592 | 0.01 | -0.71 | -0.89 | -0.88 | Unidentified |
| 10412211 | 0.07 | -0.12 | -0.56 | -0.88 | Gzma |
| 10449280 | -0.35 | -0.23 | -0.50 | -0.87 | Gm5226 |
| 10583529 | 0.15 | 0.12 | -0.94 | -0.87 | Icam4 |
| 10450675 | 0.02 | 0.02 | -0.75 | -0.87 | H2-T24 |
| 10403361 | -0.04 | -0.12 | -1.02 | -0.87 | Pitrm1 |
| 10341199 | 0.37 | -0.05 | -0.31 | -0.87 | Unidentified |
| 10435697 | -0.06 | -0.28 | -0.71 | -0.87 | Popdc2 |
| 10600797 | -0.12 | -0.38 | -0.89 | -0.57 | Apoo-ps |
| 10579609 | -0.12 | -0.32 | -0.69 | -0.57 | Fcho1 |
| 10444957 | 0.04 | -0.13 | -0.40 | -0.56 | Ppp1r10 |
| 10575534 | -0.14 | -0.20 | -1.13 | -0.56 | St3gal2 |
| 10590648 | -0.17 | -0.18 | -0.82 | -0.56 | Unidentified |
| 10592618 | -0.02 | -0.15 | -0.92 | -0.56 | Tbcel |
| 10339258 | 0.18 | -0.71 | -1.06 | -0.56 | Unidentified |
| 10386344 | -0.02 | 0.01 | -0.63 | -0.56 | Iba57 |
| 10368888 | -0.04 | 0.05 | -0.91 | -0.56 | Foxo3 |
| 10512451 | 0.09 | 0.14 | -1.13 | -0.56 | Fam214b |
| 10473343 | -0.07 | -0.07 | -0.67 | -0.56 | Med19 |
| 10511446 | -0.07 | -0.13 | -0.39 | -0.56 | Asph |
| 10607747 | 0.12 | -0.07 | -1.06 | -0.56 | Siah1b |
| 10346303 | -0.09 | -0.15 | -0.60 | -0.56 | Hspe1 |
| 10499366 | -0.01 | 0.13 | -0.82 | -0.56 | Pmf1 |
| 10481111 | -0.16 | -0.22 | -0.60 | -0.56 | Unidentified |
| 10477004 | -0.06 | -0.13 | -0.82 | -0.56 | Unidentified |
| 10481380 | 0.00 | 0.00 | -0.45 | -0.55 | 2600006K01Rik |
| 10371888 | 0.20 | 0.43 | -1.02 | -0.55 | Tmpo |
| 10341962 | -0.16 | -0.28 | -0.90 | -0.55 | Unidentified |
| 10476314 | 0.01 | 0.03 | -0.72 | -0.55 | Prnp |
| 10372082 | 0.12 | -0.03 | -1.25 | -0.55 | Nudt4 |
| 10503123 | -0.26 | -0.44 | -0.91 | -0.55 | Ubxn2b |
| 10443221 | 0.00 | 0.07 | -0.70 | -0.55 | Uhrf1bp1 |
| 10565250 | -0.17 | -0.31 | -0.75 | -0.55 | Mesdc1 |
| 10461093 | -0.10 | 0.00 | -0.77 | -0.55 | Pla2g16 |
| 10407022 | 0.07 | -0.44 | -0.73 | -0.55 | EG238836 |
| 10445867 | -0.09 | -0.15 | -0.55 | -0.55 | Plcl2 |
| 10381250 | -0.08 | -0.24 | -0.46 | -0.55 | Tubg1 |
| 10368845 | 0.01 | -0.15 | -0.72 | -0.55 | Cep57l1 |
| 10380773 | -0.16 | -0.18 | -0.93 | -0.55 | Arhgap23 |
| 10579047 | 0.01 | 0.29 | -1.10 | -0.55 | Lzts1 |
| 10492590 | -0.14 | -0.17 | -0.92 | -0.54 | Ppm1l |
| 10416273 | -0.05 | -0.12 | -0.65 | -0.54 | Phyhip |
| 10535413 | -0.07 | 0.04 | -0.84 | -0.54 | Unidentified |
| 10513622 | 0.16 | 0.10 | -0.78 | -0.54 | Pole3 |
| 10547492 | -0.12 | -0.21 | -0.69 | -0.54 | Ccdc77 |
| 10533090 | 0.16 | 0.12 | -0.85 | -0.54 | Rfc5 |
| 10339077 | 0.29 | -0.29 | -0.63 | -0.54 | Unidentified |
| 10340557 | 0.16 | 0.04 | -0.68 | -0.54 | Unidentified |
| 10593205 | -0.13 | -0.20 | -0.62 | -0.53 | Rexo2 |
| 10541642 | 0.01 | -0.08 | -0.77 | -0.53 | Vmn2r26 |
| 10379998 | -0.29 | -0.28 | -0.78 | -0.53 | Trim37 |
| 10339957 | 0.14 | 0.03 | -0.49 | -0.53 | Unidentified |
| 10471337 | -0.22 | -0.40 | -0.64 | -0.53 | Pomt1 |
| 10494395 | -0.01 | 0.16 | -1.04 | -0.53 | Hist2h2aa1 |
| 10600485 | -0.11 | -0.39 | -0.84 | -0.53 | Dkc1 |
| 10535647 | -0.20 | -0.10 | -0.59 | -0.53 | Atp5j2 |
| 10526217 | -0.20 | -0.22 | -0.61 | -0.53 | Rfc2 |
| 10577623 | 0.04 | -0.16 | -0.58 | -0.53 | Gins4 |
| 10426544 | -0.02 | -0.12 | -0.49 | -0.53 | Slc48a1 |
| 10592058 | -0.05 | -0.09 | -0.78 | -0.53 | Tuba1b |
| 10489542 | 0.16 | -0.29 | -0.74 | -0.53 | Wfdc3 |
| 10512489 | 0.09 | 0.00 | -0.87 | -0.52 | Arhgef39 |
| 10473650 | 0.01 | -0.07 | -0.43 | -0.52 | Nup160 |
| 10488482 | -0.06 | -0.23 | -0.44 | -0.52 | Acss1 |
| 10343721 | -0.01 | -0.15 | -0.56 | -0.52 | Unidentified |
| 10430778 | 0.06 | -0.04 | -0.66 | -0.52 | Phf5a |
| 10454984 | -0.10 | -0.21 | -0.44 | -0.52 | Wdr55 |
| 10465895 | -0.05 | -0.14 | -0.58 | -0.52 | Fads2 |
| 10342583 | 0.52 | -0.10 | -0.57 | -0.52 | Unidentified |
| 10529215 | -0.03 | -0.02 | -0.62 | -0.52 | Unidentified |
| 10529142 | 0.11 | -0.06 | -0.70 | -0.52 | Ppm1g |
| 10443108 | 0.06 | -0.18 | -0.73 | -0.52 | Syngap1 |
| 10582190 | 0.03 | 0.03 | -1.01 | -0.52 | Gins2 |
| 10508711 | 0.00 | -0.30 | -0.44 | -0.52 | Taf12 |
| 10547633 | -0.06 | -0.06 | -0.54 | -0.52 | Gdf3 |
| 10357856 | -0.16 | -0.40 | -0.72 | -0.52 | Unidentified |
| 10377541 | -0.14 | -0.13 | -0.55 | -0.52 | Naa38 |
| 10438328 | -0.25 | -0.19 | -0.66 | -0.51 | Tango2 |
| 10601471 | 0.11 | -0.22 | -0.43 | -0.51 | Gm5393 |
| 10436561 | 0.02 | 0.08 | -0.77 | -0.51 | Usp25 |
| 10342556 | 0.17 | -0.56 | -0.91 | -0.51 | Unidentified |
| 10379646 | -0.11 | 0.02 | -1.18 | -0.51 | Slfn3 |
| 10430997 | -0.05 | 0.18 | -0.70 | -0.51 | Pacsin2 |
| 10541279 | 0.01 | -0.21 | -0.83 | -0.51 | Bcl2l13 |
| 10366073 | -0.23 | -0.33 | -1.16 | -0.51 | Cep290 |
| 10342041 | -0.04 | -0.13 | -0.46 | -0.51 | Unidentified |
| 10479833 | 0.15 | 0.08 | -1.24 | -0.51 | Optn |
| 10591369 | -0.06 | -0.09 | -1.08 | -0.51 | Dnmt1 |
| 10411722 | -0.15 | -0.08 | -0.63 | -0.33 | Mrps36 |
| 10515943 | -0.01 | 0.05 | -0.87 | -0.33 | Ctps |
| 10453825 | -0.02 | -0.16 | -0.56 | -0.33 | Snrpd1 |
| 10508759 | 0.07 | 0.09 | -0.77 | -0.33 | Rpa2 |
| 10342294 | 0.02 | -0.23 | -0.58 | -0.33 | Unidentified |
| 10473118 | -0.06 | -0.10 | -0.93 | -0.33 | Ube2e3 |
| 10449242 | -0.19 | -0.30 | -0.91 | -0.32 | Snrpg |
| 10472994 | -0.17 | -0.13 | -0.77 | -0.32 | Mtx2 |
| 10353533 | -0.11 | -0.22 | -0.85 | -0.32 | Smap1 |
| 10464572 | -0.06 | -0.05 | -0.73 | -0.32 | Ndufv1 |
| 10468489 | 0.04 | 0.09 | -1.13 | -0.32 | Xpnpep1 |
| 10349571 | 0.15 | -0.05 | -1.15 | -0.31 | Fcamr |
| 10392440 | -0.14 | 0.02 | -0.80 | -0.31 | Slc16a6 |
| 10536369 | -0.01 | -0.17 | -0.64 | -0.31 | C1galt1 |
| 10365574 | -0.02 | -0.17 | -0.74 | -0.31 | Pmch |
| 10363163 | -0.03 | -0.27 | -0.90 | -0.31 | Asf1a |
| 10447880 | -0.27 | -0.15 | -0.65 | -0.31 | Mrpl18 |
| 10588482 | 0.06 | 0.03 | -0.85 | -0.31 | Poc1a |
| 10572813 | -0.10 | -0.18 | -0.89 | -0.31 | Usmg5 |
| 10496015 | -0.05 | -0.17 | -0.74 | -0.30 | Pla2g12a |
| 10424221 | -0.05 | -0.34 | -0.97 | -0.30 | Tbc1d31 |
| 10462752 | 0.03 | -0.18 | -0.66 | -0.30 | Btaf1 |
| 10491036 | -0.01 | -0.04 | -0.76 | -0.30 | Nudt21 |
| 10444595 | 0.04 | -0.10 | -0.70 | -0.30 | Lsm2 |
| 10388234 | -0.11 | 0.03 | -1.02 | -0.30 | Gsg2 |
| 10389524 | 0.00 | -0.19 | -0.64 | -0.30 | Unidentified |
| 10553533 | -0.25 | -0.25 | -0.94 | -0.30 | Gm6181 |
| 10582981 | 0.12 | -0.04 | -1.33 | -0.29 | Tfdp1 |
| 10415791 | 0.10 | -0.05 | -0.84 | -0.29 | Rnaseh2b |
| 10421555 | 0.30 | -0.16 | -0.86 | -0.29 | Mir687 |
| 10587746 | -0.08 | -0.15 | -0.74 | -0.29 | Tmem41b |
| 10489078 | 0.13 | 0.10 | -1.10 | -0.29 | Dsn1 |
| 10339980 | -0.13 | 0.28 | -0.90 | -0.29 | Unidentified |
| 10548176 | -0.07 | -0.09 | -0.72 | -0.29 | Rhno1 |
| 10441797 | -0.01 | -0.08 | -0.72 | -0.29 | Tcp1 |
| 10475625 | 0.01 | 0.12 | -0.77 | -0.29 | Eid1 |
| 10496379 | -0.01 | 0.06 | -1.02 | -0.29 | H2afz |
| 10540999 | 0.00 | 0.04 | -0.93 | -0.29 | H2afz |
| 10341229 | 0.26 | -0.49 | -0.74 | -0.28 | Unidentified |
| 10375559 | 0.04 | 0.20 | -1.28 | -0.28 | Unidentified |
| 10348004 | 0.01 | -0.04 | -0.75 | -0.28 | Psmd1 |
| 10448459 | -0.04 | -0.10 | -0.83 | -0.28 | Tbc1d24 |
| 10580722 | -0.03 | -0.15 | -0.72 | -0.28 | Nudt21 |
| 10530641 | -0.22 | -0.36 | -1.30 | -0.28 | Usp46 |
| 10396952 | -0.17 | -0.26 | -0.77 | -0.28 | Ttc9 |
| 10416155 | -0.20 | -0.17 | -0.88 | -0.28 | Kctd9 |
| 10492558 | 0.23 | 0.50 | -0.97 | -0.27 | Smc4 |
| 10395612 | -0.03 | -0.28 | -0.80 | -0.27 | G2e3 |
| 10414113 | 0.03 | 0.11 | -0.95 | -0.27 | Wapl |
| 10560304 | -0.18 | -0.17 | -0.74 | -0.27 | Calm3 |
| 10604630 | 0.14 | 0.22 | -0.97 | -0.26 | Mospd1 |
| 10405189 | 0.27 | -0.05 | -1.26 | -0.26 | Unidentified |
| 10340968 | -0.25 | 0.03 | -1.13 | -0.26 | Unidentified |
| 10344137 | 0.01 | -0.05 | -0.79 | -0.26 | Unidentified |
| 10341925 | 0.41 | 0.11 | -0.94 | -0.26 | Unidentified |
| 10428561 | 0.00 | 0.00 | -0.84 | -0.26 | Rad21 |
| 10604528 | 0.09 | 0.04 | -0.96 | -0.26 | Mbnl3 |
| 10447417 | -0.07 | -0.21 | -0.87 | -0.26 | Msh6 |
| 10528170 | -0.16 | -0.15 | -0.77 | -0.25 | Cycs |
| 10410560 | 0.07 | 0.17 | -1.13 | -0.25 | Trip13 |
| 10453867 | 0.09 | -0.05 | -1.12 | -0.25 | Rbbp8 |
| 10512136 | -0.28 | -0.29 | -0.83 | -0.24 | Bag1 |
| 10588091 | -0.09 | -0.12 | -1.02 | -0.23 | Cep70 |
| 10352194 | -0.14 | -0.08 | -0.88 | -0.23 | Cdc42bpa |
| 10457020 | -0.19 | -0.13 | -0.99 | -0.22 | Unidentified |
| 10340239 | -0.95 | 0.07 | -1.19 | -0.22 | Unidentified |
| 10338519 | 0.02 | 0.06 | -0.96 | -0.22 | Unidentified |
| 10499483 | -0.45 | -0.35 | -0.93 | -0.22 | Fdps |
| 10582388 | 0.04 | 0.29 | -1.31 | -0.21 | Unidentified |
| 10360720 | 0.11 | 0.20 | -1.06 | -0.21 | Wdr26 |
| 10352267 | 0.04 | -0.29 | -1.04 | -0.21 | Lin9 |
| 10340753 | -0.27 | -0.14 | -0.91 | -0.21 | Unidentified |
| 10574378 | -0.22 | -0.26 | -0.94 | -0.20 | Gins3 |
| 10574427 | -0.15 | 0.11 | -1.09 | -0.20 | Impdh2 |
| 10404038 | -0.09 | -0.19 | -1.16 | -0.19 | Hist1h3d |
| 10368199 | -0.10 | -0.08 | -1.14 | -0.19 | Myb |
| 10546024 | 0.03 | 0.01 | -1.32 | -0.18 | Prokr1 |
| 10343615 | -0.71 | -0.60 | -1.37 | -0.17 | Unidentified |
| 10570373 | 0.05 | -0.07 | -1.18 | -0.16 | Tfdp1 |
| 10606436 | -0.03 | -0.15 | -1.22 | -0.16 | Hmgn5 |

**Supplemental Table 12.** List of genes in cluster 25

|  | **Log ratio** | | | |  |
| --- | --- | --- | --- | --- | --- |
| **Probe Set ID** | **A.SW_Brain** | **SJL_Brain** | **A.SW_Spleen** | **SJL_Spleen** | **Symbol** |
| 10481627 | 4.99 | 6.15 | -1.93 | 1.75 | Lcn2 |
| 10523359 | 2.85 | 5.47 | 0.24 | 0.01 | Cxcl13 |
| 10379727 | 2.04 | 4.85 | 0.51 | 1.70 | Wfdc17 |
| 10372648 | 2.79 | 4.60 | -0.19 | 0.14 | Lyz2 |
| 10456005 | 1.84 | 4.60 | 0.14 | -0.15 | Cd74 |
| 10501020 | 0.97 | 4.45 | -0.86 | 3.71 | Chil3 |
| 10563597 | 1.95 | 4.40 | 0.32 | 2.01 | Saa3 |
| 10389231 | 2.28 | 4.38 | -0.16 | -0.07 | Ccl3 |
| 10545235 | 1.05 | 4.34 | -0.35 | -0.12 | Igkv8-28 |
| 10403031 | 0.29 | 4.26 | 0.24 | 0.23 | Ighv1-55 |
| 10363070 | 2.79 | 4.26 | 0.46 | 1.91 | Lilr4b |
| 10603551 | 2.18 | 4.22 | 0.13 | 0.62 | Cybb |
| 10455970 | 1.55 | 4.13 | -0.61 | -0.74 | BC023105 |
| 10450154 | 1.26 | 4.07 | 0.29 | -0.14 | H2-Aa |
| 10461614 | 2.24 | 4.07 | 0.13 | 0.61 | Ms4a6c |
| 10444298 | 0.95 | 3.88 | 0.26 | -0.15 | H2-Eb1 |
| 10598976 | 2.01 | 3.87 | 0.17 | 0.27 | Timp1 |
| 10444291 | 0.89 | 3.72 | 0.23 | -0.21 | H2-Ab1 |
| 10389207 | 0.40 | 3.58 | -0.48 | -0.44 | Ccl5 |
| 10461721 | 1.58 | 3.57 | 0.10 | 0.30 | Mpeg1 |
| 10416837 | 0.81 | 3.57 | 0.04 | -0.06 | Acod1 |
| 10429560 | 0.06 | 3.53 | 0.09 | -0.50 | Ly6i |
| 10398075 | 2.83 | 3.53 | 2.22 | 3.25 | Serpina3n |
| 10466210 | 1.80 | 3.51 | 0.16 | 1.23 | Ms4a6d |
| 10567580 | 1.83 | 3.45 | 0.21 | 0.70 | Igsf6 |
| 10455961 | 0.73 | 3.43 | -0.37 | -0.63 | Iigp1 |
| 10608681 | 0.74 | 3.43 | -0.29 | -0.52 | Unidentified |
| 10545190 | 1.34 | 3.38 | -0.10 | -0.20 | Unidentified |
| 10360382 | 1.01 | 3.38 | 0.33 | 0.65 | Ifi204 |
| 10466127 | 0.46 | 3.36 | -0.07 | -0.26 | AW112010 |
| 10538187 | 4.09 | 3.33 | 0.43 | 2.87 | Gpnmb |
| 10363082 | 2.33 | 3.32 | 0.22 | 0.98 | Lilrb4a |
| 10450075 | 1.18 | 3.31 | 0.26 | 0.00 | H2-K1 |
| 10547664 | 1.12 | 3.30 | -0.49 | 1.83 | Clec4e |
| 10455954 | 1.01 | 3.27 | 0.19 | 0.08 | Gm4951 |
| 10545198 | 1.52 | 3.27 | -0.06 | -0.20 | Igkv4-59 |
| 10548375 | 2.01 | 3.24 | -0.10 | 0.45 | Clec7a (dectin-1) |
| 10487588 | 1.05 | 3.21 | -0.71 | 0.80 | Il1a |
| 10606016 | 0.90 | 3.18 | 0.39 | -0.40 | Il2rg |
| 10349968 | 1.14 | 3.15 | -1.14 | 2.03 | Chil1 |
| 10375608 | 0.96 | 3.14 | 0.20 | 0.14 | Scgb3a1 |
| 10496592 | 0.66 | 3.08 | -0.15 | -0.69 | Gbp2 |
| 10389222 | 1.69 | 3.06 | 0.34 | 1.16 | Ccl6 |
| 10444824 | 0.53 | 3.04 | 0.71 | 0.12 | H2-Q6 |
| 10545247 | 1.54 | 3.03 | -0.29 | 0.16 | Igkv6-15 |
| 10360377 | 1.03 | 3.03 | 0.02 | 0.85 | AI607873 |
| 10494271 | 1.28 | 3.03 | 0.17 | 0.06 | Ctss |
| 10385500 | 0.73 | 3.00 | -0.25 | -0.54 | Irgm1 |
| 10461587 | 1.83 | 2.98 | 1.37 | 2.96 | Unidentified |
| 10462390 | 0.65 | 2.97 | 0.21 | -0.25 | Cd274 |
| 10452316 | 3.03 | 2.95 | 0.42 | 0.18 | C3 |
| 10541614 | 1.07 | 2.94 | 0.28 | 1.28 | Clec4d |
| 10425066 | 1.37 | 2.89 | 0.07 | 0.89 | Csf2rb |
| 10517165 | 1.12 | 2.89 | 0.17 | -0.21 | Cd52 |
| 10351509 | 0.78 | 2.88 | -0.13 | 0.52 | Fcgr4 |
| 10517513 | 1.76 | 2.88 | 0.09 | 0.53 | C1qc |
| 10476945 | 1.36 | 2.84 | 0.12 | 0.75 | Cst7 |
| 10538871 | 0.83 | 2.83 | 0.02 | -0.06 | Igkv2-137 |
| 10420114 | 1.67 | 2.79 | -0.13 | -0.23 | Tgm1 |
| 10405587 | 1.19 | 2.76 | 0.39 | 0.69 | Tgfbi |
| 10523156 | 1.04 | 2.75 | 0.41 | 0.38 | Cxcl2 |
| 10444780 | 0.91 | 2.75 | 0.33 | 0.08 | H2-D1 |
| 10545184 | 1.44 | 2.74 | -0.14 | -0.23 | Unidentified |
| 10545196 | 1.24 | 2.72 | 0.02 | -0.23 | Igkc |
| 10451953 | 1.14 | 2.70 | 1.13 | 1.75 | Lrg1 |
| 10360028 | 1.28 | 2.66 | 0.45 | 0.11 | Fcgr2b |
| 10521667 | 1.03 | 2.66 | -0.28 | 0.03 | Bst1 |
| 10444830 | 1.02 | 2.63 | 0.36 | 0.02 | H2-Q7 |
| 10601385 | 1.58 | 2.61 | 0.39 | 1.02 | Tlr13 |
| 10458382 | 1.02 | 2.58 | 0.09 | 1.55 | Cd14 |
| 10360070 | 1.38 | 2.57 | -0.19 | 0.53 | Fcer1g |
| 10364262 | 1.08 | 2.55 | 0.02 | 0.40 | Itgb2 |
| 10547740 | 0.84 | 2.54 | 0.39 | 0.78 | C1s1 |
| 10517508 | 1.36 | 2.52 | 0.20 | 0.48 | C1qb |
| 10461622 | 0.89 | 2.52 | 0.32 | 0.19 | Ms4a6b |
| 10501063 | 1.46 | 2.51 | 0.17 | 0.00 | Cd53 |
| 10545187 | 1.46 | 2.49 | -0.14 | -0.31 | Gm1502 |
| 10547657 | 2.08 | 2.49 | 0.15 | 1.32 | C3ar1 |
| 10484463 | 1.45 | 2.47 | 0.59 | 0.54 | Serping1 |
| 10582303 | 1.43 | 2.44 | 0.15 | 0.23 | Cyba |
| 10517517 | 1.14 | 2.43 | 0.50 | 0.86 | C1qa |
| 10347291 | 1.13 | 2.42 | -0.06 | 1.28 | Cxcr2 |
| 10554789 | 1.04 | 2.42 | 0.04 | 0.21 | Ctsc |
| 10511779 | 3.88 | 2.40 | 0.18 | 1.88 | Atp6v0d2 |
| 10526410 | 2.12 | 2.39 | 0.70 | 0.09 | Hspb1 |
| 10408928 | 2.10 | 2.38 | 0.67 | 0.15 | Hspb1 |
| 10387536 | 1.70 | 2.38 | 0.05 | 0.18 | Cd68 |
| 10422760 | 0.98 | 2.36 | 0.19 | 0.63 | Fyb |
| 10430302 | 1.26 | 2.36 | -0.15 | 0.32 | Csf2rb2 |
| 10473384 | 1.90 | 2.33 | -0.32 | -0.46 | Slc43a3 |
| 10559467 | 1.44 | 2.31 | 0.03 | 0.31 | Pira11 |
| 10427628 | 1.41 | 2.31 | 1.43 | 1.28 | Il7r |
| 10583669 | 1.21 | 2.31 | 0.36 | 0.35 | AB124611 |
| 10578264 | 1.94 | 2.29 | 0.19 | 1.19 | Msr1 |
| 10369615 | 1.44 | 2.22 | 0.03 | 0.24 | Srgn |
| 10351679 | 1.45 | 2.14 | 0.36 | 0.61 | Cd84 |
| 10607870 | 1.56 | 2.10 | 0.37 | 0.73 | Tlr7 |
| 10547621 | 1.89 | 2.06 | 0.70 | 0.83 | Apobec1 |
| 10414360 | 1.90 | 2.06 | 0.06 | 0.98 | Lgals3 |
| 10538921 | 2.15 | 2.03 | -0.06 | 0.04 | Igkv1-110 |
| 10545231 | 2.22 | 1.97 | -0.11 | -0.02 | Igkv6-32 |
| 10538880 | 1.95 | 1.95 | 0.09 | 0.13 | Unidentified |
| 10523717 | 2.20 | 1.93 | 0.18 | 0.56 | Spp1 |
| 10451932 | 2.03 | 1.90 | 0.53 | 0.27 | Plin4 |

**Supplemental Table 13.** List of genes in cluster 28

|  | **Log ratio** | | | |  |
| --- | --- | --- | --- | --- | --- |
| **Probe Set ID** | **A.SW_Brain** | **SJL_Brain** | **A.SW_Spleen** | **SJL_Spleen** | **Symbol** |
| 10344438 | 0.70 | -1.69 | -0.59 | 0.81 | Unidentified |
| 10342092 | 0.69 | -1.31 | -0.19 | 0.52 | Unidentified |
| 10342003 | 1.22 | -1.31 | -0.27 | -0.48 | Unidentified |
| 10362424 | 0.69 | -1.30 | 0.40 | -0.39 | Unidentified |
| 10339105 | 0.14 | -1.17 | -0.56 | 0.07 | Unidentified |
| 10542223 | 0.21 | -1.14 | -0.02 | -0.02 | 5430401F13Rik |
| 10343011 | -0.05 | -1.13 | -0.39 | 0.16 | Unidentified |
| 10343455 | 0.58 | -1.10 | -0.16 | 0.64 | Unidentified |
| 10343776 | 0.43 | -1.10 | 0.27 | 0.66 | Unidentified |
| 10531078 | 0.05 | -1.10 | 0.19 | -0.07 | Ugt2a1 |
| 10398406 | 0.04 | -1.09 | -0.09 | -0.09 | mir-368 |
| 10342020 | 0.28 | -1.04 | 0.24 | 0.01 | Unidentified |
| 10341702 | 0.71 | -1.04 | -0.18 | -0.07 | Unidentified |
| 10583887 | 0.03 | -1.03 | 0.05 | -0.13 | Npsr1 |
| 10470200 | 0.16 | -1.03 | -0.05 | -0.23 | Lcn11 |
| 10344337 | 1.05 | -1.02 | -0.63 | 0.52 | Unidentified |
| 10593981 | 0.00 | -1.01 | 0.18 | 0.00 | Cyp1a2 |
| 10477406 | 0.03 | -1.01 | 0.13 | -0.14 | Bpifb3 |
| 10343522 | 0.21 | -1.00 | -0.03 | 0.17 | Unidentified |
| 10341935 | 0.18 | -1.00 | 0.07 | 0.52 | Unidentified |
| 10362440 | 0.22 | -1.00 | -0.12 | 0.08 | Unidentified |
| 10340133 | 0.49 | -0.99 | 0.51 | 0.40 | Unidentified |
| 10343047 | 0.15 | -0.98 | -0.13 | 0.42 | Unidentified |
| 10499189 | 0.12 | -0.98 | 0.18 | -0.28 | Fcrls |
| 10600057 | 0.05 | -0.98 | -0.03 | -0.11 | Cnga2 |
| 10344164 | -0.02 | -0.97 | -0.23 | -0.01 | Unidentified |
| 10342795 | 0.07 | -0.95 | -0.10 | 0.17 | Unidentified |
| 10531111 | 0.12 | -0.94 | 0.15 | -0.14 | SULT1E1 |
| 10339219 | 0.52 | -0.93 | -0.30 | 0.84 | Unidentified |
| 10343676 | 0.25 | -0.93 | -0.18 | -0.09 | Unidentified |
| 10340062 | 0.11 | -0.93 | -0.52 | 0.47 | Unidentified |
| 10341567 | 0.05 | -0.91 | -0.02 | 0.30 | Unidentified |
| 10342631 | 0.27 | -0.89 | 0.09 | -0.35 | Unidentified |
| 10343889 | 0.24 | -0.89 | 0.27 | 0.13 | Unidentified |
| 10341382 | 0.02 | -0.88 | 0.07 | 0.21 | Unidentified |
| 10341672 | 0.69 | -0.88 | -0.51 | 0.13 | Unidentified |
| 10341250 | -0.01 | -0.87 | -0.06 | -0.18 | Unidentified |
| 10343600 | 0.51 | -0.86 | -0.78 | 0.59 | Unidentified |
| 10340648 | 0.18 | -0.85 | -0.08 | 0.14 | Unidentified |
| 10562314 | 0.35 | -0.84 | -0.21 | -0.12 | Unidentified |
| 10341486 | -0.22 | -0.84 | -0.29 | 0.18 | Unidentified |
| 10341065 | 1.18 | -0.84 | -0.57 | -0.39 | Unidentified |
| 10339799 | -0.08 | -0.84 | -0.31 | 0.40 | Unidentified |
| 10342361 | 0.16 | -0.83 | -0.36 | -0.08 | Unidentified |
| 10551282 | 0.02 | -0.83 | 0.07 | 0.00 | Unidentified |
| 10340081 | 0.19 | -0.82 | 0.36 | 0.31 | Unidentified |
| 10342213 | 0.15 | -0.82 | 0.26 | 0.00 | Unidentified |
| 10339268 | 0.38 | -0.82 | -0.07 | 0.49 | Unidentified |
| 10343595 | -0.05 | -0.80 | -0.26 | 0.18 | Unidentified |
| 10344805 | -0.09 | -0.80 | -0.27 | 0.02 | Cspp1 |
| 10340426 | 0.04 | -0.79 | -0.11 | 0.05 | Unidentified |
| 10440141 | 0.08 | -0.79 | 0.14 | -0.06 | Olfr177 |
| 10340334 | 0.35 | -0.79 | 0.05 | 0.57 | Unidentified |
| 10482772 | -0.22 | -0.79 | -0.17 | 0.25 | Nr4a2 |
| 10338677 | 0.05 | -0.78 | -0.55 | 0.13 | Unidentified |
| 10344267 | 0.18 | -0.78 | -0.42 | 0.61 | Unidentified |
| 10343995 | 0.54 | -0.78 | -0.44 | 0.09 | Unidentified |
| 10339687 | 0.06 | -0.77 | 0.05 | -0.18 | Unidentified |
| 10343607 | -0.24 | -0.77 | -0.42 | 0.38 | Unidentified |
| 10338336 | 0.27 | -0.77 | -0.01 | 0.11 | Unidentified |
| 10522904 | -0.06 | -0.76 | 0.02 | -0.02 | 2310003L06Rik |
| 10344376 | 0.00 | -0.76 | -0.11 | 0.12 | Unidentified |
| 10339900 | 0.04 | -0.76 | 0.03 | 0.53 | Unidentified |
| 10543428 | 0.07 | -0.75 | 0.01 | -0.10 | Iqub |
| 10341006 | 0.15 | -0.75 | -0.13 | 0.35 | Unidentified |
| 10343799 | 0.01 | -0.74 | -0.47 | 0.40 | Unidentified |
| 10341676 | 0.00 | -0.74 | 0.08 | 0.08 | Unidentified |
| 10344146 | 0.39 | -0.74 | -0.86 | 0.10 | Unidentified |
| 10339017 | 0.27 | -0.73 | 0.16 | 0.60 | Unidentified |
| 10341391 | 0.18 | -0.73 | 0.23 | 0.13 | Unidentified |
| 10339048 | -0.08 | -0.72 | -0.12 | 0.29 | Unidentified |
| 10443704 | 0.02 | -0.72 | 0.02 | -0.04 | Umodl1 |
| 10339425 | 0.17 | -0.72 | -0.08 | -0.02 | Unidentified |
| 10340493 | 0.16 | -0.72 | 0.21 | 0.57 | Unidentified |
| 10343115 | 0.11 | -0.72 | 0.10 | 0.16 | Unidentified |
| 10343896 | -0.02 | -0.72 | 0.09 | 0.36 | Unidentified |
| 10606257 | -0.25 | -0.72 | -0.16 | 0.20 | mir-384 |
| 10341994 | 0.54 | -0.72 | -0.42 | -0.07 | Unidentified |
| 10344222 | 0.18 | -0.71 | -0.43 | 0.09 | Unidentified |
| 10338183 | -0.05 | -0.71 | -0.08 | 0.39 | Unidentified |
| 10484777 | 0.02 | -0.70 | -0.05 | -0.06 | Olfr1216 |
| 10484832 | 0.27 | -0.70 | 0.03 | -0.07 | Olfr1246 |
| 10342960 | 0.22 | -0.69 | -0.24 | 0.14 | Unidentified |
| 10342317 | 0.34 | -0.69 | 0.17 | 0.10 | Unidentified |
| 10338797 | 0.16 | -0.68 | -0.28 | 0.40 | Unidentified |
| 10343759 | 0.19 | -0.68 | -0.18 | 0.47 | Unidentified |
| 10341742 | -0.19 | -0.67 | 0.02 | 0.30 | Unidentified |
| 10422946 | 0.16 | -0.67 | 0.06 | -0.06 | Ranbp3l |
| 10341058 | -0.06 | -0.66 | -0.37 | 0.04 | Unidentified |
| 10339298 | -0.09 | -0.66 | -0.28 | 0.47 | Unidentified |
| 10343083 | 0.14 | -0.66 | 0.04 | 0.35 | Unidentified |
| 10341137 | 0.03 | -0.66 | 0.17 | 0.41 | Unidentified |
| 10343330 | 0.27 | -0.65 | 0.19 | 0.67 | Unidentified |
| 10343316 | 0.34 | -0.65 | -0.05 | -0.36 | Unidentified |
| 10343190 | 0.10 | -0.65 | -0.12 | -0.04 | Unidentified |
| 10340313 | 0.10 | -0.65 | -0.40 | 0.08 | Unidentified |
| 10342823 | 0.15 | -0.65 | -0.48 | 0.33 | Unidentified |
| 10484662 | 0.07 | -0.65 | -0.14 | -0.08 | Olfr1110 |
| 10341661 | 0.18 | -0.65 | -0.07 | 0.68 | Unidentified |
| 10342105 | 0.01 | -0.65 | 0.16 | 0.18 | Unidentified |
| 10343470 | -0.19 | -0.64 | -0.38 | 0.40 | Unidentified |
| 10343869 | 0.14 | -0.64 | -0.06 | -0.06 | Unidentified |
| 10339226 | -0.06 | -0.64 | -0.04 | 0.31 | Unidentified |
| 10343591 | 0.15 | -0.64 | 0.05 | 0.11 | Unidentified |
| 10343369 | 0.31 | -0.64 | -0.16 | 0.08 | Unidentified |
| 10373846 | 0.01 | -0.64 | 0.05 | -0.07 | Sec14l3 |
| 10356403 | 0.19 | -0.64 | 0.18 | -0.17 | Kcnj13 |
| 10340463 | 0.16 | -0.63 | -0.26 | 0.51 | Unidentified |
| 10338261 | 0.44 | -0.63 | -0.72 | -0.12 | Unidentified |
| 10339498 | -0.06 | -0.63 | -0.13 | 0.37 | Unidentified |
| 10355259 | -0.04 | -0.63 | -0.11 | 0.22 | Myl1 |
| 10344445 | 0.32 | -0.62 | -0.19 | 0.11 | Unidentified |
| 10477423 | 0.01 | -0.62 | -0.06 | -0.13 | Bpifb4 |
| 10342978 | 0.12 | -0.62 | -0.42 | 0.30 | Unidentified |
| 10342987 | 0.24 | -0.62 | 0.14 | 0.47 | Unidentified |
| 10340718 | 0.26 | -0.62 | -0.32 | 0.50 | Unidentified |
| 10339916 | -0.14 | -0.61 | -0.01 | 0.28 | Unidentified |
| 10339633 | 0.31 | -0.61 | -0.14 | 0.49 | Unidentified |
| 10339496 | 0.10 | -0.61 | -0.05 | 0.46 | Unidentified |
| 10339410 | 0.26 | -0.61 | -0.45 | 0.20 | Unidentified |
| 10338456 | 0.07 | -0.61 | 0.17 | 0.15 | Unidentified |
| 10341556 | -0.27 | -0.61 | -0.15 | 0.36 | Unidentified |
| 10338266 | 0.47 | -0.60 | -0.04 | 0.12 | Unidentified |
| 10341809 | 0.36 | -0.60 | -0.05 | 0.52 | Unidentified |
| 10338328 | 0.92 | -0.60 | -0.50 | 0.42 | Unidentified |
| 10343173 | 0.27 | -0.60 | -0.58 | 0.21 | Unidentified |
| 10339605 | 0.38 | -0.60 | -0.05 | 0.55 | Unidentified |
| 10362418 | 0.14 | -0.60 | -0.08 | 0.03 | Unidentified |
| 10344542 | 0.20 | -0.59 | 0.27 | 0.09 | Unidentified |
| 10341267 | 0.22 | -0.59 | -0.09 | 0.13 | Unidentified |
| 10340945 | -0.13 | -0.59 | 0.02 | 0.23 | Unidentified |
| 10342820 | -0.16 | -0.59 | -0.13 | 0.11 | Unidentified |
| 10463643 | -0.08 | -0.59 | -0.46 | 0.12 | Unidentified |
| 10339883 | 0.19 | -0.59 | 0.01 | 0.03 | Unidentified |
| 10341368 | 0.11 | -0.59 | -0.33 | 0.14 | Unidentified |
| 10344540 | 0.21 | -0.58 | -0.22 | 0.11 | Unidentified |
| 10341781 | -0.03 | -0.58 | 0.00 | 0.36 | Unidentified |
| 10341517 | 0.08 | -0.58 | -0.06 | 0.05 | Unidentified |
| 10338401 | 0.08 | -0.58 | 0.06 | 0.29 | Unidentified |
| 10341960 | 0.07 | -0.58 | -0.12 | 0.39 | Unidentified |
| 10343236 | 0.04 | -0.57 | -0.41 | 0.04 | Unidentified |
| 10342966 | 0.24 | -0.57 | -0.15 | -0.21 | Unidentified |
| 10342395 | 0.25 | -0.57 | -0.37 | -0.20 | Unidentified |
| 10339592 | 0.17 | -0.57 | -0.14 | -0.05 | Unidentified |
| 10341819 | 0.22 | -0.57 | -0.51 | 0.55 | Unidentified |
| 10342852 | 0.51 | -0.56 | 0.07 | 0.54 | Unidentified |
| 10342145 | -0.03 | -0.56 | -0.47 | 0.16 | Unidentified |
| 10341417 | 0.04 | -0.56 | -0.45 | 0.10 | Unidentified |
| 10407797 | -0.02 | -0.56 | -0.05 | -0.03 | Prl2c3 |
| 10419060 | -0.08 | -0.56 | 0.01 | 0.09 | Unidentified |
| 10566624 | -0.03 | -0.55 | 0.02 | 0.19 | Unidentified |
| 10339458 | 0.23 | -0.55 | 0.14 | 0.23 | Unidentified |
| 10343719 | -0.08 | -0.55 | 0.07 | 0.17 | Unidentified |
| 10342055 | 0.57 | -0.55 | -0.30 | 0.19 | Unidentified |
| 10342861 | 1.02 | -0.55 | 0.18 | 0.83 | Unidentified |
| 10608682 | -0.13 | -0.55 | -0.20 | 0.06 | Unidentified |
| 10343314 | 0.35 | -0.55 | 0.17 | 0.23 | Unidentified |
| 10341685 | -0.05 | -0.54 | 0.04 | 0.13 | Unidentified |
| 10342936 | 0.53 | -0.54 | -0.07 | 0.19 | Unidentified |
| 10343355 | 0.04 | -0.54 | 0.08 | -0.03 | Unidentified |
| 10467385 | 0.35 | -0.53 | -0.39 | 0.27 | Cyp2c68 |
| 10485716 | 0.12 | -0.53 | -0.05 | -0.09 | Unidentified |
| 10564231 | -0.06 | -0.53 | -0.11 | 0.01 | Unidentified |
| 10338194 | 0.28 | -0.53 | 0.17 | 0.20 | Unidentified |
| 10344580 | 0.05 | -0.53 | 0.06 | 0.00 | Unidentified |
| 10343020 | -0.02 | -0.53 | -0.09 | -0.01 | Unidentified |
| 10350377 | -0.06 | -0.53 | -0.04 | 0.23 | Zbtb41 |
| 10451761 | 0.13 | -0.53 | -0.02 | 0.17 | Tbc1d5 |
| 10398440 | 0.12 | -0.53 | -0.05 | 0.10 | mir-154 |
| 10342468 | 0.54 | -0.52 | -0.23 | 0.38 | Unidentified |
| 10338548 | -0.01 | -0.52 | -0.19 | 0.46 | Unidentified |
| 10560164 | 0.06 | -0.52 | -0.14 | -0.14 | Obox6 |
| 10342810 | 0.08 | -0.52 | -0.42 | 0.14 | Unidentified |
| 10338583 | 0.41 | -0.52 | 0.23 | 0.39 | Unidentified |
| 10405063 | 0.47 | -0.52 | -0.25 | 0.53 | Ogn |
| 10374012 | -0.14 | -0.52 | -0.05 | 0.24 | Rasl10a |
| 10340692 | 0.44 | -0.51 | 0.09 | -0.12 | Unidentified |
| 10357656 | -0.01 | -0.51 | -0.29 | 0.21 | Unidentified |
| 10457959 | -0.11 | -0.51 | -0.06 | 0.10 | Sft2d3 |
| 10338591 | 0.27 | -0.50 | 0.05 | -0.10 | Unidentified |
| 10338175 | 0.22 | -0.50 | 0.26 | 0.32 | Unidentified |
| 10554351 | 0.25 | -0.50 | 0.18 | -0.01 | Wdr93 |
| 10362448 | 0.38 | -0.50 | -0.07 | -0.08 | Unidentified |
| 10338246 | 0.24 | -0.50 | 0.15 | 0.06 | Unidentified |
| 10344064 | 0.46 | -0.50 | -0.19 | 0.38 | Unidentified |
| 10342776 | 0.28 | -0.50 | -0.24 | -0.06 | Unidentified |
| 10585984 | 0.06 | -0.50 | -0.18 | 0.19 | Unidentified |
| 10528909 | 0.33 | -0.50 | -0.03 | 0.15 | Unidentified |
| 10342002 | 0.22 | -0.50 | 0.13 | 0.57 | Unidentified |
| 10341203 | 0.13 | -0.50 | -0.35 | 0.17 | Unidentified |
| 10342413 | 0.17 | -0.50 | -0.27 | 0.07 | Unidentified |
| 10440340 | 0.12 | -0.50 | -0.09 | -0.08 | Unidentified |
| 10339529 | -0.12 | -0.49 | -0.19 | 0.13 | Unidentified |
| 10343697 | -0.08 | -0.49 | -0.12 | 0.19 | Unidentified |
| 10550818 | 0.00 | -0.49 | -0.10 | -0.01 | Vmn1r174 |
| 10545569 | 0.05 | -0.49 | -0.16 | -0.08 | Reg3g |
| 10341082 | 0.32 | -0.49 | 0.13 | 0.60 | Unidentified |
| 10340485 | -0.19 | -0.49 | -0.07 | 0.28 | Unidentified |
| 10341014 | 0.10 | -0.48 | 0.14 | 0.10 | Unidentified |
| 10438169 | 0.00 | -0.48 | 0.06 | 0.30 | Unidentified |
| 10338985 | -0.11 | -0.48 | -0.05 | 0.22 | Unidentified |
| 10341803 | 0.08 | -0.48 | -0.31 | 0.11 | Unidentified |
| 10343755 | 0.17 | -0.48 | 0.06 | 0.27 | Unidentified |
| 10485794 | 0.14 | -0.48 | 0.15 | -0.10 | Olfr1305 |
| 10412826 | 0.16 | -0.48 | -0.01 | -0.14 | Unidentified |
| 10339762 | 0.44 | -0.48 | -0.37 | 0.31 | Unidentified |
| 10537290 | 0.08 | -0.48 | -0.47 | 0.05 | Unidentified |
| 10342683 | 0.13 | -0.47 | 0.21 | 0.38 | Unidentified |
| 10339610 | 0.15 | -0.47 | -0.05 | -0.04 | Unidentified |
| 10339691 | -0.13 | -0.47 | -0.20 | 0.11 | Unidentified |
| 10344042 | 0.10 | -0.47 | 0.03 | -0.08 | Unidentified |
| 10440158 | 0.33 | -0.47 | 0.12 | -0.03 | Olfr191 |
| 10344274 | 0.63 | -0.47 | 0.30 | 0.07 | Unidentified |
| 10341067 | -0.05 | -0.47 | -0.13 | 0.42 | Unidentified |
| 10344789 | -0.11 | -0.47 | -0.20 | 0.08 | Cspp1 |
| 10343510 | 0.73 | -0.47 | -0.18 | 0.55 | Unidentified |
| 10504912 | 0.13 | -0.47 | 0.21 | 0.32 | Unidentified |
| 10566686 | -0.01 | -0.46 | -0.25 | 0.03 | Olfr490 |
| 10341370 | 0.08 | -0.46 | 0.13 | 0.18 | Unidentified |
| 10343430 | 0.37 | -0.46 | 0.12 | 0.12 | Unidentified |
| 10340974 | 0.06 | -0.46 | -0.26 | 0.28 | Unidentified |
| 10344246 | 0.17 | -0.46 | 0.13 | -0.08 | Unidentified |
| 10344390 | 0.30 | -0.46 | -0.26 | -0.17 | Unidentified |
| 10342279 | -0.05 | -0.46 | -0.32 | 0.19 | Unidentified |
| 10342737 | -0.01 | -0.45 | -0.23 | 0.14 | Unidentified |
| 10592404 | 0.43 | -0.45 | 0.32 | -0.16 | Olfr948 |
| 10367481 | 0.16 | -0.45 | -0.01 | 0.11 | Olfr776 |
| 10338898 | 0.05 | -0.45 | -0.20 | 0.26 | Unidentified |
| 10342711 | 0.03 | -0.45 | 0.08 | 0.21 | Unidentified |
| 10547793 | 0.36 | -0.45 | 0.24 | 0.01 | Unidentified |
| 10398408 | 0.00 | -0.45 | -0.31 | 0.07 | mir-368 |
| 10340504 | 0.06 | -0.45 | 0.05 | -0.01 | Unidentified |
| 10341755 | 0.14 | -0.45 | -0.23 | 0.20 | Unidentified |
| 10582582 | -0.04 | -0.45 | -0.11 | 0.05 | Unidentified |
| 10582584 | -0.04 | -0.45 | -0.11 | 0.05 | Unidentified |
| 10549171 | -0.14 | -0.44 | -0.23 | 0.17 | C2cd5 |
| 10351998 | 0.06 | -0.44 | -0.09 | -0.08 | Unidentified |
| 10485786 | 0.32 | -0.44 | 0.18 | -0.12 | Olfr1298 |
| 10410362 | -0.03 | -0.44 | 0.04 | 0.11 | Zfp738 |
| 10343675 | 0.30 | -0.44 | 0.04 | 0.62 | Unidentified |
| 10343036 | 0.05 | -0.44 | -0.05 | 0.10 | Unidentified |
| 10343573 | 0.59 | -0.44 | -0.33 | 0.31 | Unidentified |
| 10442262 | -0.05 | -0.44 | -0.35 | 0.07 | Zfp758 |
| 10340224 | 0.00 | -0.44 | -0.25 | 0.11 | Unidentified |
| 10340895 | 0.08 | -0.44 | -0.19 | 0.05 | Unidentified |
| 10430679 | 0.03 | -0.44 | -0.20 | -0.01 | Unidentified |
| 10343699 | 0.53 | -0.43 | -0.41 | -0.15 | Unidentified |
| 10560812 | 0.14 | -0.43 | -0.08 | -0.07 | Unidentified |
| 10340054 | 0.28 | -0.43 | 0.06 | 0.03 | Unidentified |
| 10340213 | -0.03 | -0.43 | 0.01 | 0.14 | Unidentified |
| 10362428 | 0.54 | -0.43 | -0.27 | 0.12 | Unidentified |
| 10566620 | 0.18 | -0.43 | 0.04 | -0.06 | Olfr711 |
| 10343674 | 0.12 | -0.43 | 0.11 | 0.31 | Unidentified |
| 10340556 | 0.40 | -0.43 | 0.14 | 0.02 | Unidentified |
| 10338879 | 0.30 | -0.43 | -0.28 | 0.21 | Unidentified |
| 10340026 | 0.21 | -0.43 | -0.09 | 0.46 | Unidentified |
| 10339948 | 0.28 | -0.43 | 0.23 | 0.06 | Unidentified |
| 10435494 | 0.51 | -0.43 | -0.42 | -0.20 | Unidentified |
| 10554727 | 0.13 | -0.42 | 0.06 | -0.03 | Olfr297 |
| 10339575 | 0.07 | -0.42 | -0.30 | 0.05 | Unidentified |
| 10342150 | 0.30 | -0.42 | -0.18 | 0.15 | Unidentified |
| 10484616 | 0.34 | -0.42 | 0.13 | 0.19 | Olfr1082 |
| 10375227 | 0.06 | -0.42 | 0.12 | 0.09 | Unidentified |
| 10362160 | 0.20 | -0.42 | -0.24 | 0.10 | Unidentified |
| 10340859 | 0.24 | -0.42 | -0.10 | -0.08 | Unidentified |
| 10340034 | 0.00 | -0.42 | -0.26 | 0.24 | Unidentified |
| 10343894 | 0.17 | -0.42 | 0.05 | -0.07 | Unidentified |
| 10340826 | 0.11 | -0.41 | 0.00 | 0.23 | Unidentified |
| 10438784 | 0.20 | -0.41 | 0.01 | -0.14 | Gmnc |
| 10338368 | 0.10 | -0.41 | -0.07 | 0.11 | Unidentified |
| 10544497 | 0.13 | -0.41 | -0.19 | -0.03 | Unidentified |
| 10345875 | 0.32 | -0.41 | -0.17 | 0.03 | Unidentified |
| 10343068 | 0.47 | -0.41 | 0.24 | 0.56 | Unidentified |
| 10340228 | -0.08 | -0.41 | -0.32 | 0.15 | Unidentified |
| 10339752 | 0.06 | -0.41 | -0.27 | 0.27 | Unidentified |
| 10457485 | 0.23 | -0.41 | 0.10 | 0.04 | mir-133 |
| 10567506 | 0.25 | -0.41 | -0.01 | 0.06 | Unidentified |
| 10338612 | 0.10 | -0.41 | 0.07 | 0.13 | Unidentified |
| 10343249 | 0.00 | -0.41 | -0.11 | 0.16 | Unidentified |
| 10342807 | 0.12 | -0.41 | 0.01 | 0.11 | Unidentified |
| 10342540 | 0.16 | -0.40 | 0.11 | -0.04 | Unidentified |
| 10537801 | 0.05 | -0.40 | -0.10 | 0.00 | Olfr449 |
| 10595626 | 0.00 | -0.40 | -0.15 | 0.18 | Unidentified |
| 10342591 | 0.18 | -0.40 | -0.17 | 0.49 | Unidentified |
| 10339547 | 0.38 | -0.40 | 0.21 | 0.24 | Unidentified |
| 10362729 | 0.17 | -0.40 | 0.02 | -0.09 | Unidentified |
| 10461832 | 0.28 | -0.39 | 0.04 | 0.00 | Olfr1496 |
| 10338153 | 0.32 | -0.39 | 0.09 | 0.18 | Unidentified |
| 10548653 | 0.22 | -0.39 | -0.03 | 0.05 | Prpmp5 |
| 10340562 | 0.21 | -0.39 | -0.20 | 0.01 | Unidentified |
| 10349791 | 0.10 | -0.39 | -0.02 | 0.18 | Unidentified |
| 10414468 | 0.04 | -0.39 | -0.13 | 0.16 | Olfr727 |
| 10343632 | 0.28 | -0.38 | 0.06 | 0.16 | Unidentified |
| 10340803 | 0.18 | -0.38 | -0.13 | 0.42 | Unidentified |
| 10342582 | 0.37 | -0.38 | -0.21 | -0.10 | Unidentified |
| 10587688 | -0.01 | -0.38 | -0.17 | 0.18 | Unidentified |
| 10595620 | -0.01 | -0.38 | -0.17 | 0.18 | Unidentified |
| 10340375 | 0.29 | -0.38 | -0.10 | 0.22 | Unidentified |
| 10341716 | 0.31 | -0.38 | -0.22 | 0.22 | Unidentified |
| 10549797 | 0.13 | -0.38 | -0.01 | -0.06 | Vmn1r62 |
| 10505643 | 0.11 | -0.38 | -0.08 | -0.03 | Ccdc171 |
| 10401841 | 0.07 | -0.38 | -0.14 | 0.21 | Dio2 |
| 10516466 | 0.05 | -0.38 | -0.10 | 0.06 | Zmym1 |
| 10338570 | 0.12 | -0.38 | -0.09 | 0.40 | Unidentified |
| 10413136 | 0.10 | -0.37 | -0.12 | 0.20 | Unidentified |
| 10341940 | -0.05 | -0.37 | -0.11 | 0.22 | Unidentified |
| 10591203 | 0.17 | -0.37 | -0.01 | -0.05 | Olfr860 |
| 10339631 | 0.34 | -0.37 | 0.07 | 0.06 | Unidentified |
| 10344599 | 0.43 | -0.37 | 0.02 | -0.09 | Unidentified |
| 10342453 | 0.10 | -0.37 | -0.26 | 0.22 | Unidentified |
| 10439282 | 0.10 | -0.37 | -0.02 | 0.27 | Csta1 |
| 10338502 | 0.19 | -0.37 | -0.07 | 0.24 | Unidentified |
| 10484636 | 0.20 | -0.37 | -0.17 | 0.21 | Olfr1097 |
| 10341990 | 0.27 | -0.37 | -0.03 | 0.30 | Unidentified |
| 10338439 | 0.31 | -0.36 | -0.20 | -0.08 | Unidentified |
| 10368711 | 0.23 | -0.36 | 0.00 | -0.10 | Unidentified |
| 10343712 | 0.35 | -0.36 | -0.21 | 0.48 | Unidentified |
| 10338230 | 0.59 | -0.35 | -0.61 | 0.28 | Unidentified |
| 10338825 | 0.31 | -0.35 | 0.15 | -0.07 | Unidentified |
| 10342028 | 0.24 | -0.35 | -0.28 | 0.03 | Unidentified |
| 10343610 | 0.25 | -0.35 | -0.18 | -0.17 | Unidentified |
| 10344576 | 0.19 | -0.35 | -0.03 | 0.10 | Unidentified |
| 10601461 | 0.15 | -0.35 | -0.03 | -0.03 | Cylc1 |
| 10341374 | 0.47 | -0.35 | -0.50 | 0.23 | Unidentified |
| 10339037 | 0.04 | -0.35 | -0.14 | 0.15 | Unidentified |
| 10595622 | 0.02 | -0.34 | -0.13 | 0.21 | Unidentified |
| 10595636 | 0.02 | -0.34 | -0.13 | 0.21 | Unidentified |
| 10362454 | 0.11 | -0.34 | -0.03 | 0.06 | Unidentified |
| 10342564 | 0.25 | -0.34 | -0.16 | 0.32 | Unidentified |
| 10344412 | 0.26 | -0.34 | 0.08 | 0.11 | Unidentified |
| 10344809 | 0.06 | -0.34 | -0.05 | 0.16 | Cspp1 |
| 10342794 | 0.44 | -0.34 | -0.23 | 0.51 | Unidentified |
| 10339562 | 0.84 | -0.34 | -0.20 | 0.60 | Unidentified |
| 10590821 | 0.16 | -0.34 | 0.00 | 0.15 | 9230110C19Rik |
| 10341611 | 0.31 | -0.34 | 0.07 | -0.14 | Unidentified |
| 10340241 | 0.12 | -0.34 | -0.24 | 0.15 | Unidentified |
| 10455310 | 0.18 | -0.33 | -0.14 | 0.01 | Unidentified |
| 10343667 | 0.44 | -0.33 | -0.28 | 0.12 | Unidentified |
| 10415804 | 0.07 | -0.33 | -0.16 | 0.15 | Unidentified |
| 10566680 | 0.16 | -0.33 | -0.11 | 0.06 | Olfr486 |
| 10344568 | 0.28 | -0.33 | 0.12 | 0.43 | Unidentified |
| 10572083 | 0.34 | -0.33 | 0.23 | 0.29 | Unidentified |
| 10343066 | 0.28 | -0.32 | -0.16 | -0.14 | Unidentified |
| 10340414 | 0.09 | -0.32 | -0.05 | 0.16 | Unidentified |
| 10344504 | 0.25 | -0.32 | -0.10 | 0.11 | Unidentified |
| 10484716 | 0.31 | -0.32 | 0.17 | 0.03 | Olfr1164 |
| 10473592 | 0.23 | -0.31 | -0.21 | -0.03 | Olfr1183 |
| 10340255 | 0.44 | -0.31 | -0.34 | 0.39 | Unidentified |
| 10341177 | 0.46 | -0.31 | -0.35 | 0.34 | Unidentified |
| 10344183 | 0.38 | -0.30 | 0.02 | 0.26 | Unidentified |
| 10338309 | 0.54 | -0.30 | -0.42 | 0.42 | Unidentified |
| 10338568 | 0.31 | -0.30 | 0.13 | 0.17 | Unidentified |
| 10466298 | 0.26 | -0.30 | -0.01 | -0.05 | Olfr1436 |
| 10514388 | 0.31 | -0.29 | -0.23 | 0.08 | Unidentified |
| 10340991 | 0.26 | -0.29 | -0.17 | 0.03 | Unidentified |
| 10339083 | 0.41 | -0.29 | -0.41 | 0.13 | Unidentified |
| 10341119 | 0.32 | -0.29 | 0.16 | 0.18 | Unidentified |
| 10567500 | 0.21 | -0.28 | 0.02 | 0.06 | Unidentified |
| 10343105 | 0.35 | -0.28 | -0.02 | 0.16 | Unidentified |
| 10342122 | 0.30 | -0.28 | -0.05 | 0.42 | Unidentified |
| 10341884 | 0.20 | -0.27 | -0.25 | 0.25 | Unidentified |
| 10341278 | 0.25 | -0.27 | -0.12 | 0.10 | Unidentified |
| 10344378 | 0.26 | -0.27 | -0.11 | 0.01 | Unidentified |
| 10342094 | 0.26 | -0.26 | -0.10 | 0.41 | Unidentified |
| 10343895 | 0.30 | -0.26 | -0.17 | 0.10 | Unidentified |
| 10340922 | 0.33 | -0.26 | -0.01 | 0.28 | Unidentified |
| 10343230 | 0.27 | -0.25 | -0.23 | 0.27 | Unidentified |
| 10596403 | 0.26 | -0.20 | -0.20 | 0.22 | Col6a5 |

**Supplemental Table 14.** List of genes in cluster 29

|  | **Log ratio** | | | |  |
| --- | --- | --- | --- | --- | --- |
| **Probe Set ID** | **A.SW_Brain** | **SJL_Brain** | **A.SW_Spleen** | **SJL_Spleen** | **Symbol** |
| 10590494 | 0.04 | 0.30 | -2.92 | -0.77 | Kif15 |
| 10500559 | -0.08 | -0.24 | -2.90 | -0.21 | Hsd3b6 |
| 10480432 | 0.02 | 0.00 | -2.83 | -0.74 | Mastl |
| 10501963 | -0.87 | -1.04 | -2.74 | 0.10 | Ugt8a |
| 10420426 | 0.02 | 0.20 | -2.74 | -0.67 | Ska3 |
| 10436106 | 0.06 | 0.12 | -2.72 | -0.62 | C330027C09Rik |
| 10516246 | -0.08 | 0.05 | -2.67 | -0.87 | Cdca8 |
| 10346365 | 0.01 | 0.17 | -2.66 | -0.50 | Sgol2a |
| 10389606 | 0.06 | 0.22 | -2.65 | -0.57 | Prr11 |
| 10454709 | 0.09 | 0.15 | -2.61 | -0.96 | Kif20a |
| 10568150 | 0.00 | 0.03 | -2.61 | -1.06 | Kif22 |
| 10487577 | 0.01 | 0.15 | -2.58 | -0.77 | Ckap2l |
| 10557156 | 0.13 | 0.17 | -2.56 | -1.04 | Plk1 |
| 10483401 | -0.15 | 0.42 | -2.55 | -0.81 | Spc25 |
| 10576883 | -0.19 | 0.08 | -2.53 | -0.72 | Shcbp1 |
| 10487340 | 0.19 | 0.43 | -2.50 | -1.13 | Ncaph |
| 10587508 | -0.03 | 0.09 | -2.50 | -0.94 | Ttk |
| 10592201 | -0.05 | -0.19 | -2.48 | -1.09 | Chek1 |
| 10419323 | -0.08 | 0.16 | -2.47 | -1.16 | Dlgap5 |
| 10554325 | -0.06 | 0.11 | -2.45 | -0.80 | Ticrr |
| 10352767 | -0.19 | 0.21 | -2.45 | -0.83 | Nek2 |
| 10405185 | -0.09 | -0.19 | -2.44 | -0.92 | Cks2 |
| 10433096 | 0.08 | 0.07 | -2.43 | -0.37 | Nfe2 |
| 10353004 | -0.08 | -0.14 | -2.43 | -0.94 | Cks2 |
| 10377405 | 0.07 | 0.35 | -2.42 | -0.95 | Aurkb |
| 10577508 | 0.00 | -0.07 | -2.42 | -1.04 | Ckap2 |
| 10474769 | 0.03 | 0.21 | -2.41 | -0.79 | Bub1b |
| 10586448 | 0.03 | 0.50 | -2.39 | -0.63 | Pclaf |
| 10515431 | 0.15 | 0.43 | -2.38 | -0.73 | Kif2c |
| 10458195 | -0.20 | -0.04 | -2.37 | -0.60 | Cdc25c |
| 10490104 | 0.06 | 0.04 | -2.36 | -1.19 | Aurka |
| 10446074 | 0.10 | 0.17 | -2.34 | -1.28 | Uhrf1 |
| 10544106 | -0.01 | -0.26 | -2.33 | -0.90 | Unidentified |
| 10460738 | 0.01 | 0.16 | -2.32 | -1.09 | Cdca5 |
| 10424779 | -0.12 | -0.10 | -2.30 | -0.90 | Cks2 |
| 10601705 | 0.13 | 0.15 | -2.29 | -0.89 | Cenpi |
| 10504957 | 0.09 | -0.17 | -2.29 | -1.03 | Smc2 |
| 10544982 | -0.03 | -0.07 | -2.28 | -1.03 | Nt5c3 |
| 10391461 | -0.01 | 0.03 | -2.27 | -1.19 | Brca1 |
| 10416037 | 0.05 | -0.05 | -2.26 | -1.28 | Pbk |
| 10399908 | -0.17 | -0.41 | -2.25 | 0.25 | Prkar2b |
| 10485963 | 0.42 | 0.45 | -2.25 | -0.21 | Arhgap11a |
| 10474229 | 0.08 | 0.29 | -2.24 | -0.36 | Cd59a |
| 10467637 | -0.16 | 0.27 | -2.23 | -0.32 | Arhgap19 |
| 10530783 | 0.04 | -0.05 | -2.22 | -0.94 | A730089K16Rik |
| 10575733 | 0.07 | -0.04 | -2.22 | -0.91 | Cenpn |
| 10594251 | 0.10 | 0.35 | -2.22 | -0.66 | Kif23 |
| 10341025 | 0.18 | -0.36 | -2.22 | -0.59 | Unidentified |
| 10403943 | -0.06 | 0.17 | -2.22 | -1.17 | Hist1h2bm |
| 10399391 | -0.08 | -0.18 | -2.21 | -1.25 | Gen1 |
| 10399314 | 0.02 | 0.00 | -2.21 | -0.13 | Mfsd2b |
| 10540738 | 0.02 | 0.00 | -2.19 | -1.21 | Fancd2 |
| 10410919 | 0.21 | 0.07 | -2.18 | -0.72 | Unidentified |
| 10513818 | -0.36 | -0.37 | -2.17 | -0.53 | Stmn1 |
| 10571870 | 0.10 | 0.61 | -2.17 | 0.00 | Hmgb2 |
| 10510172 | 0.14 | 0.62 | -2.16 | 0.01 | Hmgb2 |
| 10524790 | -0.10 | -0.12 | -2.15 | -0.84 | Cit |
| 10524266 | 0.13 | 0.38 | -2.15 | -0.95 | Chek2 |
| 10478572 | 0.17 | 0.46 | -2.15 | -1.44 | Ube2c |
| 10564978 | 0.14 | 0.28 | -2.15 | -1.09 | Blm |
| 10474902 | -0.06 | -0.04 | -2.14 | -1.17 | Rad51 |
| 10542079 | 0.10 | 0.16 | -2.14 | -0.66 | Foxm1 |
| 10478355 | 0.07 | 0.03 | -2.14 | -1.05 | Mybl2 |
| 10526559 | -0.16 | -0.18 | -2.14 | -0.03 | Ache |
| 10512236 | 0.16 | 0.02 | -2.14 | -0.94 | Kif24 |
| 10495763 | 0.06 | -0.01 | -2.14 | -1.01 | Gclm |
| 10600765 | -0.17 | -0.25 | -2.14 | -0.94 | Pcyt1b |
| 10425755 | 0.09 | -0.01 | -2.13 | -1.25 | Unidentified |
| 10474223 | 0.00 | -0.18 | -2.13 | -1.15 | Cd59b |
| 10518350 | 0.11 | 0.54 | -2.13 | -0.02 | Hmgb2 |
| 10508986 | -0.37 | -0.38 | -2.13 | -0.53 | Stmn1 |
| 10508151 | -0.01 | 0.00 | -2.13 | -0.95 | Clspn |
| 10494889 | 0.10 | -0.01 | -2.12 | -0.56 | Dennd2c |
| 10459576 | -0.47 | -0.24 | -2.11 | -1.24 | Cep76 |
| 10361995 | 0.18 | 0.10 | -2.11 | -0.57 | Mtfr2 |
| 10466779 | 0.01 | -0.18 | -2.11 | -0.57 | Pip5k1b |
| 10548086 | 0.00 | 0.05 | -2.10 | -0.79 | Rad51ap1 |
| 10515257 | -0.07 | -0.01 | -2.10 | -1.10 | Rad54l |
| 10518352 | 0.17 | 0.65 | -2.10 | 0.01 | Hmgb2 |
| 10555695 | 0.01 | 0.06 | -2.10 | -1.40 | Rrm1 |
| 10547943 | -0.02 | -0.09 | -2.09 | -1.06 | Ncapd2 |
| 10379363 | 0.18 | 0.22 | -2.09 | -0.76 | Atad5 |
| 10473356 | 0.40 | 1.18 | -2.09 | -1.51 | Ube2l6 |
| 10541729 | 0.15 | 0.15 | -2.08 | -0.82 | Cdca3 |
| 10548699 | -0.09 | 0.20 | -2.08 | -1.39 | Unidentified |
| 10466659 | -0.08 | -0.16 | -2.07 | -0.21 | Gda |
| 10361375 | 0.17 | 0.06 | -2.07 | -1.23 | Fbxo5 |
| 10408200 | -0.03 | 0.00 | -2.06 | -0.59 | Hist1h4f |
| 10573261 | 0.27 | 0.44 | -2.06 | -1.37 | Asf1b |
| 10491805 | -0.07 | -0.02 | -2.05 | -0.62 | Plk4 |
| 10416655 | -0.17 | -0.21 | -2.05 | -0.67 | Kbtbd6 |
| 10565570 | 0.04 | 0.24 | -2.03 | -1.05 | Ddias |
| 10344092 | 0.42 | 0.36 | -2.02 | -0.74 | Unidentified |
| 10450519 | -0.05 | 0.08 | -2.02 | -1.41 | Tcf19 |
| 10590909 | 0.24 | 0.24 | -2.01 | -1.51 | Endod1 |
| 10339038 | 0.08 | 0.09 | -1.98 | -1.49 | Unidentified |
| 10385052 | 0.07 | -0.16 | -1.98 | -0.80 | Ranbp17 |
| 10474825 | 0.07 | 0.23 | -1.98 | -0.72 | Knstrn |
| 10502071 | -0.14 | 0.14 | -1.97 | -0.46 | 5730508B09Rik |
| 10550102 | 0.03 | -0.05 | -1.97 | -1.10 | Lig1 |
| 10499639 | 0.25 | 0.26 | -1.97 | -0.84 | Cks1b |
| 10586907 | 0.03 | 0.00 | -1.96 | -1.41 | Mns1 |
| 10367076 | -0.02 | -0.28 | -1.96 | -1.12 | Prim1 |
| 10523923 | -0.23 | -0.11 | -1.96 | -0.66 | Ccdc18 |
| 10527801 | 0.06 | 0.07 | -1.96 | -1.10 | Brca2 |
| 10461979 | 0.43 | 0.04 | -1.95 | -1.33 | Aldh1a1 |
| 10437040 | 0.37 | 0.40 | -1.95 | -0.91 | Chaf1b |
| 10546163 | 0.00 | -0.01 | -1.94 | -1.34 | Mcm2 |
| 10407081 | 0.01 | -0.30 | -1.93 | -0.32 | Depdc1b |
| 10389816 | -0.02 | -0.24 | -1.93 | -0.75 | Tom1l1 |
| 10563338 | 0.13 | 0.35 | -1.92 | -1.35 | Ppp1r15a |
| 10474998 | -0.05 | 0.07 | -1.91 | -1.31 | Unidentified |
| 10507131 | -0.09 | -0.08 | -1.91 | -1.34 | Tal1 |
| 10395142 | 0.04 | -0.15 | -1.91 | -1.55 | Sh3yl1 |
| 10476301 | 0.22 | 0.35 | -1.91 | -1.15 | Smox |
| 10366277 | -0.01 | -0.06 | -1.91 | -0.62 | E2f7 |
| 10340905 | -0.59 | 0.01 | -1.90 | -0.03 | Unidentified |
| 10356457 | -0.35 | -0.40 | -1.90 | -1.56 | Dnajb3 |
| 10515090 | 0.16 | 0.21 | -1.89 | -0.88 | Cdkn2c |
| 10526630 | 0.22 | 0.03 | -1.89 | -1.50 | Tfr2 |
| 10421029 | -0.05 | -0.02 | -1.88 | -0.57 | Cdca2 |
| 10383819 | 0.00 | -0.16 | -1.88 | -1.35 | Sec14l2 |
| 10515744 | -0.03 | 0.13 | -1.88 | -0.74 | Cdc20 |
| 10343004 | 0.31 | 0.32 | -1.87 | -1.47 | Unidentified |
| 10342557 | -0.26 | 0.18 | -1.87 | -0.79 | Unidentified |
| 10521090 | 0.19 | 0.32 | -1.86 | -0.38 | Tacc3 |
| 10586184 | -0.17 | 0.00 | -1.86 | -0.63 | Tipin |
| 10343265 | -0.12 | -0.07 | -1.86 | -1.29 | Unidentified |
| 10432511 | -0.03 | 0.03 | -1.86 | -0.33 | Racgap1 |
| 10380489 | -0.01 | -0.05 | -1.86 | -0.70 | Samd14 |
| 10584710 | -0.05 | -0.21 | -1.85 | -0.74 | H2afx |
| 10520521 | 0.13 | 0.26 | -1.84 | -0.94 | Cenpa |
| 10393320 | 0.01 | -0.13 | -1.83 | -1.32 | Ube2o |
| 10479811 | -0.01 | 0.18 | -1.83 | -1.03 | Mcm10 |
| 10408197 | -0.05 | 0.20 | -1.82 | -1.12 | Hist1h2bh |
| 10452860 | 0.00 | -0.25 | -1.82 | -1.04 | Memo1 |
| 10603087 | -0.06 | -0.11 | -1.82 | -1.63 | Pir |
| 10408616 | -0.11 | -0.21 | -1.81 | -1.29 | Slc22a23 |
| 10352954 | 0.08 | 0.18 | -1.81 | -0.79 | Hmgb3 |
| 10459552 | 0.09 | 0.01 | -1.80 | -1.46 | Spire1 |
| 10605674 | -0.01 | -0.21 | -1.80 | -0.97 | Pola1 |
| 10586252 | -0.13 | -0.13 | -1.80 | -0.72 | Dennd4a |
| 10533213 | 0.05 | 0.50 | -1.80 | -1.19 | Oas3 |
| 10581538 | 0.19 | -0.07 | -1.79 | -1.39 | Nqo1 |
| 10393431 | -0.03 | 0.15 | -1.79 | -1.36 | Tk1 |
| 10534041 | 0.06 | 0.09 | -1.79 | -1.29 | Stx2 |
| 10594426 | 0.09 | 0.12 | -1.78 | -0.89 | Zwilch |
| 10504470 | 0.00 | -0.05 | -1.78 | -0.73 | Melk |
| 10552311 | 0.31 | -0.08 | -1.78 | -1.10 | Unidentified |
| 10495945 | -0.03 | -0.10 | -1.78 | -0.37 | Zgrf1 |
| 10394770 | 0.06 | 0.31 | -1.78 | -1.01 | Odc1 |
| 10582809 | 0.05 | 0.08 | -1.78 | -1.49 | Tk1 |
| 10350838 | 0.04 | 0.11 | -1.78 | -0.46 | Pclaf |
| 10582295 | 0.34 | 0.41 | -1.78 | -1.23 | Unidentified |
| 10352048 | -0.02 | 0.13 | -1.77 | -0.91 | Exo1 |
| 10524169 | 0.23 | 0.28 | -1.77 | -0.73 | Pole |
| 10361748 | -0.09 | -0.05 | -1.77 | -1.20 | Fbxo30 |
| 10571680 | 0.03 | -0.04 | -1.77 | -0.12 | Cenpu |
| 10507653 | -0.04 | 0.22 | -1.77 | -0.88 | Unidentified |
| 10498620 | -0.46 | -0.65 | -1.76 | -1.56 | Trim59 |
| 10554281 | 0.21 | 0.10 | -1.75 | -0.81 | Fanci |
| 10358457 | -0.29 | -0.09 | -1.75 | -1.72 | Bex4 |
| 10435581 | -0.02 | -0.01 | -1.75 | -1.04 | Polq |
| 10403413 | -0.74 | -0.80 | -1.75 | -0.64 | Idi1 |
| 10389395 | 0.02 | 0.18 | -1.74 | -0.36 | Brip1 |
| 10427166 | 0.01 | 0.06 | -1.74 | -1.09 | Espl1 |
| 10507137 | 0.09 | 0.04 | -1.74 | -1.13 | Pdzk1ip1 |
| 10494407 | -0.16 | -0.06 | -1.74 | -0.80 | Hist2h2bb |
| 10355742 | -0.15 | -0.15 | -1.74 | -1.41 | Abcb6 |
| 10438378 | 0.07 | 0.02 | -1.74 | -0.79 | Cdc45 |
| 10419296 | 0.12 | 0.16 | -1.73 | -1.02 | Wdhd1 |
| 10340529 | 0.07 | -0.16 | -1.73 | -0.80 | Unidentified |
| 10357590 | 0.03 | -0.09 | -1.73 | -1.12 | Dyrk3 |
| 10515156 | -0.04 | -0.17 | -1.72 | -1.46 | Minpp1 |
| 10497503 | 0.03 | -0.25 | -1.72 | -1.05 | Kpna2 |
| 10497343 | 0.00 | -0.05 | -1.72 | -1.10 | Unidentified |
| 10338163 | -0.02 | -0.23 | -1.72 | -1.69 | Unidentified |
| 10534974 | 0.18 | 0.14 | -1.72 | -1.11 | Mcm7 |
| 10354897 | -0.04 | -0.12 | -1.72 | -1.25 | Trak2 |
| 10576090 | -0.10 | -0.02 | -1.71 | -1.11 | Zfpm1 |
| 10606532 | -0.03 | -0.02 | -1.71 | -1.24 | Acp1 |
| 10538640 | 0.35 | 0.04 | -1.71 | -1.36 | Abcg2 |
| 10488785 | 0.08 | -0.14 | -1.70 | -0.94 | E2f1 |
| 10528077 | -0.03 | 0.66 | -1.70 | -0.35 | Dbf4 |
| 10341349 | -0.28 | 0.14 | -1.69 | -1.26 | Unidentified |
| 10408225 | -0.24 | -0.17 | -1.68 | -0.63 | Hist1h4c |
| 10398665 | 0.63 | 1.48 | -1.68 | -0.97 | Tnfaip2 |
| 10355327 | 0.04 | 0.03 | -1.68 | -0.51 | Bard1 |
| 10412559 | -0.12 | -0.05 | -1.68 | -1.17 | Slbp |
| 10482030 | 0.29 | 0.17 | -1.67 | -1.05 | Stom |
| 10425749 | 0.03 | -0.12 | -1.67 | -1.01 | Fam109b |
| 10343564 | 0.14 | -0.09 | -1.67 | -1.28 | Unidentified |
| 10465861 | -0.08 | -0.03 | -1.66 | -0.46 | Incenp |
| 10538832 | 0.05 | 0.11 | -1.66 | -0.66 | Mad2l1 |
| 10586248 | 0.01 | -0.07 | -1.66 | -0.84 | Dennd4a |
| 10376459 | -0.01 | 0.22 | -1.65 | -0.82 | Unidentified |
| 10527229 | -0.10 | -0.15 | -1.65 | -1.61 | Fam220a |
| 10453512 | 0.05 | -0.26 | -1.65 | -0.99 | Kpna2 |
| 10503617 | -0.03 | 0.19 | -1.65 | -0.53 | Mms22l |
| 10458028 | 0.07 | 0.15 | -1.64 | -1.51 | Gypc |
| 10572906 | 0.11 | 0.07 | -1.64 | -0.98 | Mcm5 |
| 10440002 | -0.21 | -0.05 | -1.64 | -0.46 | Adgrg7 |
| 10544501 | -0.07 | -0.08 | -1.64 | -0.83 | Ezh2 |
| 10600017 | 0.15 | 0.19 | -1.63 | -0.68 | Hmgb3 |
| 10557199 | 0.02 | -0.01 | -1.63 | -1.01 | Acp1 |
| 10379034 | -0.26 | -0.43 | -1.62 | -1.60 | Tlcd1 |
| 10570278 | 0.07 | 0.06 | -1.62 | -1.41 | Unidentified |
| 10585068 | -0.02 | 0.10 | -1.62 | -1.43 | Nxpe4 |
| 10346576 | -0.10 | -0.39 | -1.62 | -1.10 | Stradb |
| 10406581 | 0.13 | -0.02 | -1.62 | -0.66 | Dhfr |
| 10403948 | -0.16 | 0.24 | -1.61 | -0.66 | Hist1h2br |
| 10573451 | 0.02 | 0.10 | -1.61 | -1.05 | Syce2 |
| 10428672 | 0.20 | -0.25 | -1.61 | -0.93 | Dscc1 |
| 10375002 | -0.03 | -0.12 | -1.61 | -1.15 | Cpeb4 |
| 10466624 | 0.11 | -0.27 | -1.61 | -0.27 | Aldh1a7 |
| 10465912 | 0.16 | 0.15 | -1.61 | -0.90 | Fen1 |
| 10586244 | -0.05 | -0.01 | -1.61 | -0.73 | Dennd4a |
| 10490955 | 0.08 | -0.17 | -1.60 | -1.32 | Mtfr1 |
| 10482762 | -0.74 | -0.83 | -1.60 | -0.63 | Idi1 |
| 10601850 | -0.21 | -0.15 | -1.60 | -1.18 | Bex4 |
| 10568266 | -0.05 | -0.09 | -1.60 | -0.39 | Ccdc189 |
| 10385325 | -0.05 | 0.14 | -1.60 | -0.58 | Pttg1 |
| 10371159 | -0.06 | -0.13 | -1.60 | -1.23 | Fzr1 |
| 10353010 | -0.04 | -0.17 | -1.60 | -0.68 | Mybl1 |
| 10403945 | -0.30 | -0.28 | -1.60 | -0.65 | Hist1h4j |
| 10459755 | -0.05 | -0.01 | -1.60 | -0.61 | Ska1 |
| 10456140 | -0.21 | -0.11 | -1.60 | -1.84 | Sh3tc2 |
| 10500327 | -0.41 | -0.55 | -1.59 | -0.76 | Hist2h3c2 |
| 10476989 | 0.14 | 0.43 | -1.59 | -0.82 | Gins1 |
| 10430195 | -0.07 | 0.01 | -1.59 | -1.45 | Apol8 |
| 10396068 | 0.01 | -0.07 | -1.58 | -1.06 | Lrr1 |
| 10437945 | 0.22 | 0.16 | -1.58 | -0.90 | Mcm4 |
| 10446027 | 0.01 | 0.06 | -1.58 | -0.81 | Chaf1a |
| 10466798 | -0.13 | -0.39 | -1.58 | -0.67 | Unidentified |
| 10577449 | -0.03 | -0.18 | -1.58 | -1.47 | Atp7b |
| 10452709 | 0.12 | 0.27 | -1.58 | -0.61 | Ndc80 |
| 10430825 | -0.02 | -0.04 | -1.58 | -0.88 | Cenpm |
| 10529299 | -0.08 | 0.05 | -1.58 | -1.09 | Slbp |
| 10453715 | 0.06 | -0.01 | -1.58 | -0.66 | Unidentified |
| 10461408 | 0.28 | 0.41 | -1.58 | -1.86 | Rab3il1 |
| 10361191 | 0.04 | -0.03 | -1.58 | -0.90 | Syt14 |
| 10338991 | -0.12 | -0.10 | -1.57 | -1.49 | Unidentified |
| 10358399 | -0.27 | -0.12 | -1.57 | -0.60 | Rgs13 |
| 10395039 | -0.10 | 0.16 | -1.57 | -1.43 | Cmpk2 |
| 10535103 | 0.05 | 0.35 | -1.57 | -1.44 | Micall2 |
| 10403978 | -0.15 | 0.24 | -1.56 | -0.66 | Hist1h2br |
| 10392284 | 0.03 | -0.27 | -1.56 | -0.95 | Kpna2 |
| 10462343 | 0.25 | 0.38 | -1.56 | -1.39 | Unidentified |
| 10366645 | 0.00 | -0.07 | -1.56 | -0.09 | 1700006J14Rik |
| 10343402 | -0.02 | -0.13 | -1.56 | -1.76 | Unidentified |
| 10408210 | -0.14 | 0.19 | -1.56 | -0.65 | Hist1h2br |
| 10363575 | 0.18 | 0.39 | -1.56 | -0.33 | Dna2 |
| 10403934 | -0.16 | -0.08 | -1.55 | -1.32 | Isca1 |
| 10607116 | -0.10 | 0.08 | -1.55 | -1.33 | Ammecr1 |
| 10351047 | 0.05 | 0.04 | -1.55 | -0.79 | Cenpl |
| 10371987 | -0.06 | 0.09 | -1.55 | -1.09 | Metap2 |
| 10400649 | 0.07 | 0.16 | -1.54 | -0.79 | Pole2 |
| 10357436 | 0.20 | 0.17 | -1.54 | -0.82 | Mcm6 |
| 10364916 | 0.03 | -0.03 | -1.54 | -1.60 | Sppl2b |
| 10343913 | 0.06 | -0.23 | -1.54 | -1.10 | Unidentified |
| 10525473 | 0.09 | -0.07 | -1.54 | -1.88 | Tmem120b |
| 10362896 | 0.68 | 0.64 | -1.54 | -1.28 | Cd24a |
| 10495935 | 0.20 | 0.05 | -1.54 | -0.40 | Zgrf1 |
| 10586250 | -0.04 | 0.02 | -1.54 | -0.61 | Dennd4a |
| 10543067 | 0.07 | -0.14 | -1.54 | -1.16 | Asns |
| 10493137 | 0.18 | 0.11 | -1.53 | -0.42 | Iqgap3 |
| 10339733 | 0.16 | 0.17 | -1.53 | -0.42 | Unidentified |
| 10542665 | -0.08 | -0.14 | -1.53 | -0.48 | Cmas |
| 10343638 | -0.04 | -0.11 | -1.52 | -1.50 | Unidentified |
| 10431035 | -0.01 | -0.28 | -1.52 | -1.24 | Ttll12 |
| 10409799 | -0.16 | -0.06 | -1.51 | -1.30 | Isca1 |
| 10570556 | -0.04 | -0.12 | -1.51 | -0.90 | Mcph1 |
| 10437590 | 0.36 | 0.38 | -1.51 | -1.06 | Carhsp1 |
| 10390746 | -0.11 | 0.17 | -1.50 | -0.28 | Unidentified |
| 10374400 | -0.10 | -0.33 | -1.50 | -0.69 | Fbxo48 |
| 10586254 | 0.01 | -0.07 | -1.50 | -0.76 | Dennd4a |
| 10434191 | -0.08 | -0.09 | -1.50 | -1.54 | Txnrd2 |
| 10448743 | -0.19 | -0.21 | -1.50 | -1.08 | Fahd1 |
| 10353420 | 0.10 | 0.24 | -1.50 | -0.90 | Mcm3 |
| 10532241 | -0.02 | -0.20 | -1.49 | -1.63 | Slc26a1 |
| 10350024 | -0.03 | -0.20 | -1.49 | -1.00 | Klhl12 |
| 10472916 | -0.06 | -0.20 | -1.49 | -0.32 | Cdca7 |
| 10408070 | -0.16 | 0.23 | -1.49 | -0.61 | Hist1h2br |
| 10422962 | 0.08 | -0.01 | -1.49 | -0.79 | Nadk2 |
| 10428763 | -0.19 | -0.02 | -1.49 | -0.76 | Atad2 |
| 10500333 | -0.07 | -0.06 | -1.49 | -0.90 | Hist2h4 |
| 10470775 | -0.19 | 0.10 | -1.49 | -1.10 | Cercam |
| 10480087 | -0.02 | -0.27 | -1.48 | -1.01 | Unidentified |
| 10480628 | -0.15 | -0.25 | -1.48 | -0.88 | Tubb4b |
| 10462973 | -0.04 | -0.25 | -1.48 | -0.14 | Hells |
| 10521136 | -0.12 | -0.24 | -1.48 | -0.50 | Nsd2 |
| 10487930 | 0.15 | 0.28 | -1.48 | -0.88 | Pcna |
| 10342397 | -0.07 | -0.12 | -1.48 | -1.59 | Unidentified |
| 10382692 | -0.03 | 0.07 | -1.48 | -0.89 | Smim5 |
| 10409031 | 0.10 | 0.09 | -1.48 | -0.64 | Dek |
| 10461391 | 0.15 | 0.29 | -1.47 | -0.90 | Pcna |
| 10606071 | 0.16 | 0.10 | -1.47 | -0.70 | Ercc6l |
| 10348618 | -0.11 | -0.17 | -1.47 | -0.99 | Asb1 |
| 10403980 | -0.17 | 0.24 | -1.47 | -0.63 | Hist1h2br |
| 10339749 | -0.02 | -0.11 | -1.47 | -1.36 | Unidentified |
| 10450904 | -0.16 | -0.25 | -1.46 | -0.59 | Scoc |
| 10400057 | 0.06 | 0.11 | -1.46 | -0.87 | Arl4a |
| 10588007 | 0.09 | -0.08 | -1.46 | -0.86 | Tfdp2 |
| 10380566 | -0.27 | -0.20 | -1.46 | -0.58 | Phospho1 |
| 10545130 | 0.11 | 0.18 | -1.45 | -0.55 | Gadd45a |
| 10404033 | -0.13 | -0.08 | -1.45 | -0.19 | Hist1h1d |
| 10450025 | -0.04 | -0.04 | -1.45 | -1.10 | March2 |
| 10544186 | -0.03 | -0.01 | -1.45 | -0.85 | Mkrn1 |
| 10515072 | -0.09 | -0.16 | -1.45 | -0.32 | Rnf11 |
| 10496796 | -0.01 | -0.10 | -1.44 | -0.60 | Ssx2ip |
| 10497337 | 0.09 | 0.04 | -1.44 | -1.58 | Car1 |
| 10362974 | -0.07 | -0.26 | -1.44 | -0.90 | Hace1 |
| 10522661 | -0.20 | -0.18 | -1.44 | -0.48 | C530008M17Rik |
| 10442616 | -0.10 | -0.09 | -1.44 | -1.38 | Hagh |
| 10401852 | 0.13 | 0.12 | -1.44 | -0.26 | Cep128 |
| 10410092 | -0.09 | -0.02 | -1.43 | -0.87 | Zfp367 |
| 10426669 | -0.10 | 0.04 | -1.43 | -0.50 | Troap |
| 10452648 | 0.38 | 0.83 | -1.43 | -0.30 | Emilin2 |
| 10455813 | -0.11 | 0.11 | -1.42 | -0.41 | Lmnb1 |
| 10408321 | -0.19 | -0.05 | -1.42 | -0.72 | Gmnn |
| 10525236 | -0.17 | -0.12 | -1.42 | -0.74 | Gm15800 |
| 10387743 | 1.04 | 0.45 | -1.42 | -1.39 | Slc2a4 |
| 10512065 | 0.04 | 0.41 | -1.42 | -0.77 | Unidentified |
| 10428310 | -0.01 | 0.15 | -1.42 | -0.87 | Azin1 |
| 10451225 | -0.01 | -0.26 | -1.42 | -0.47 | Polh |
| 10488655 | 0.23 | 0.31 | -1.42 | -0.65 | Bcl2l1 |
| 10586246 | -0.03 | 0.04 | -1.42 | -0.73 | Dennd4a |
| 10572605 | 0.07 | 0.12 | -1.42 | -1.05 | Ankle1 |
| 10500610 | 0.48 | 1.17 | -1.41 | -1.30 | Fam46c |
| 10341949 | 0.00 | -0.75 | -1.41 | -0.49 | Unidentified |
| 10397966 | -0.17 | -0.55 | -1.40 | -1.40 | Otub2 |
| 10341036 | 0.29 | -0.11 | -1.40 | -1.28 | Unidentified |
| 10503315 | -0.11 | -0.14 | -1.40 | -0.60 | Rad54b |
| 10448004 | 0.01 | -0.09 | -1.40 | -0.78 | Phf10 |
| 10570472 | -0.04 | 0.05 | -1.40 | -1.06 | Cln8 |
| 10419082 | 0.08 | -0.03 | -1.40 | -1.59 | Fam213a |
| 10400748 | 0.01 | -0.14 | -1.39 | -0.49 | Cdkl1 |
| 10566810 | -0.17 | -0.27 | -1.39 | -1.29 | Nrip3 |
| 10587873 | -0.14 | -0.28 | -1.38 | -0.78 | Unidentified |
| 10519488 | -0.15 | -0.23 | -1.38 | -0.88 | Tubb4b |
| 10408212 | -0.41 | -0.08 | -1.37 | -0.42 | Hist1h1e |
| 10350590 | -0.05 | 0.29 | -1.37 | -0.70 | Unidentified |
| 10531146 | -0.12 | -0.17 | -1.37 | -0.81 | Mkrn1-ps1 |
| 10353733 | -0.02 | -0.23 | -1.37 | -0.56 | Prim2 |
| 10435057 | 0.01 | 0.00 | -1.37 | -0.81 | Pcyt1a |
| 10548585 | 0.06 | -0.03 | -1.37 | -1.06 | Ybx3 |
| 10462632 | 0.28 | 0.10 | -1.37 | -0.35 | Kif20b |
| 10338674 | -0.11 | -0.11 | -1.37 | -1.31 | Unidentified |
| 10532133 | -0.08 | -0.23 | -1.36 | -0.82 | Evi5 |
| 10352709 | 0.07 | 0.06 | -1.36 | -0.72 | Nsl1 |
| 10338647 | 0.30 | -0.12 | -1.36 | -0.60 | Unidentified |
| 10476252 | -0.04 | -0.03 | -1.36 | -1.29 | Cdc25b |
| 10404036 | -0.18 | -0.04 | -1.36 | -0.37 | Hist1h2bg |
| 10408477 | -0.16 | -0.10 | -1.35 | -0.45 | E2f3 |
| 10375031 | -0.07 | -0.05 | -1.35 | -0.92 | Snrnp25 |
| 10465379 | -0.17 | -0.25 | -1.35 | -1.33 | Snx15 |
| 10502823 | 0.07 | 0.02 | -1.35 | -0.45 | Dnajb4 |
| 10527252 | 0.00 | 0.04 | -1.35 | -1.00 | Eif2ak1 |
| 10571657 | 0.02 | 0.18 | -1.34 | -0.39 | Acsl1 |
| 10389339 | -0.10 | -0.16 | -1.34 | -0.92 | Usp32 |
| 10600500 | -0.11 | -0.08 | -1.34 | -1.61 | Fam220a |
| 10489343 | -0.02 | 0.04 | -1.34 | -0.47 | Gm11451 |
| 10497752 | 0.22 | 0.25 | -1.34 | -1.03 | Carhsp1 |
| 10379989 | -0.11 | -0.25 | -1.34 | -0.86 | Ska2 |
| 10590245 | 0.07 | 0.00 | -1.34 | -1.37 | Slc25a38 |
| 10419288 | 0.18 | 0.02 | -1.34 | -0.95 | Gch1 |
| 10445702 | -0.17 | -0.08 | -1.34 | -0.79 | Usp49 |
| 10548857 | 0.00 | -0.10 | -1.33 | -0.72 | Hist4h4 |
| 10458398 | -0.03 | -0.08 | -1.33 | -0.68 | Hars |
| 10525210 | -0.13 | -0.08 | -1.33 | -0.88 | Gm15800 |
| 10458983 | -0.05 | 0.06 | -1.33 | -1.26 | March3 |
| 10515086 | -0.05 | 0.08 | -1.32 | -0.51 | Unidentified |
| 10508454 | 0.04 | 0.00 | -1.32 | -1.20 | Bsdc1 |
| 10413710 | 0.91 | 0.92 | -1.32 | -0.87 | Nt5dc2 |
| 10403959 | -0.17 | 0.16 | -1.32 | -0.44 | Hist1h2bq |
| 10408087 | -0.17 | 0.16 | -1.32 | -0.44 | Hist1h2bq |
| 10366983 | -0.18 | -0.32 | -1.32 | -0.93 | Nemp1 |
| 10495574 | -0.03 | -0.29 | -1.31 | -0.59 | Sass6 |
| 10474437 | 0.13 | -0.27 | -1.31 | -0.78 | Ccdc34 |
| 10459866 | 0.77 | 0.69 | -1.31 | -1.06 | Slc14a1 |
| 10460400 | -0.08 | -0.10 | -1.31 | -0.79 | Pcx |
| 10538617 | -0.04 | -0.20 | -1.31 | -0.57 | Lancl2 |
| 10504730 | -0.07 | 0.09 | -1.31 | -0.54 | Anp32b |
| 10586242 | -0.01 | -0.02 | -1.31 | -0.73 | Dennd4a |
| 10488522 | 0.00 | -0.08 | -1.31 | -0.82 | Ninl |
| 10600502 | -0.10 | -0.13 | -1.30 | -1.53 | Fam220a |
| 10340287 | -0.06 | 0.02 | -1.30 | -1.48 | Unidentified |
| 10594582 | 0.15 | -0.14 | -1.30 | -1.39 | Snx22 |
| 10408077 | -0.01 | 0.21 | -1.29 | -0.60 | Hist1h2ak |
| 10376021 | -0.15 | -0.26 | -1.29 | -1.18 | Sept8 |
| 10576140 | 0.25 | 0.26 | -1.29 | -0.59 | Cdt1 |
| 10390050 | 0.20 | 0.19 | -1.28 | -0.84 | Eme1 |
| 10521337 | -0.13 | -0.15 | -1.28 | -0.98 | Rgs12 |
| 10452151 | 0.16 | 0.00 | -1.27 | -1.31 | Rfx2 |
| 10550877 | 0.06 | 0.43 | -1.27 | -1.05 | Kcnn4 |
| 10525195 | -0.13 | -0.17 | -1.27 | -0.98 | Gm15800 |
| 10356780 | -0.05 | -0.16 | -1.27 | -0.80 | Pask |
| 10553935 | -0.14 | -0.30 | -1.27 | -0.80 | Tarsl2 |
| 10381345 | -0.03 | -0.05 | -1.27 | -0.92 | Psme3 |
| 10447023 | -0.04 | 0.27 | -1.27 | -1.12 | Unidentified |
| 10345791 | 0.17 | -0.13 | -1.26 | -1.49 | Il1rl1 |
| 10391669 | -0.15 | -0.02 | -1.26 | -1.19 | Slc25a39 |
| 10408202 | 0.01 | 0.31 | -1.26 | -0.49 | Hist2h3b |
| 10404024 | -0.19 | -0.21 | -1.26 | -0.65 | Hist1h4h |
| 10569280 | -0.05 | -0.18 | -1.26 | -0.75 | Dusp8 |
| 10386236 | -0.10 | -0.05 | -1.26 | -0.82 | Hist3h2bb-ps |
| 10494402 | 0.00 | 0.34 | -1.25 | -0.47 | Hist2h3c1 |
| 10357833 | -0.15 | -0.30 | -1.25 | -0.93 | Atp2b4 |
| 10403076 | 0.18 | 0.29 | -1.25 | -0.83 | Unidentified |
| 10408083 | 0.01 | 0.30 | -1.25 | -0.48 | Hist2h3b |
| 10383532 | -0.09 | -0.14 | -1.25 | -0.75 | Narf |
| 10541307 | 0.48 | 1.38 | -1.25 | -1.66 | Usp18 |
| 10408266 | 0.16 | -0.02 | -1.25 | -0.89 | Hist1h2ba |
| 10493086 | -0.01 | -0.06 | -1.25 | -0.67 | Hdgf |
| 10352548 | -0.19 | -0.58 | -1.24 | -1.04 | Slc30a10 |
| 10358754 | 0.14 | 0.10 | -1.24 | -0.95 | Unidentified |
| 10355401 | 0.05 | -0.02 | -1.24 | -0.97 | Acp1 |
| 10382973 | 0.04 | -0.01 | -1.24 | -1.30 | 6030468B19Rik |
| 10548761 | -0.01 | 0.15 | -1.24 | -1.18 | Hebp1 |
| 10463716 | -0.08 | -0.21 | -1.24 | -1.17 | Cnnm2 |
| 10498827 | 0.28 | 0.36 | -1.24 | -0.70 | Fnip2 |
| 10370046 | 0.04 | 0.19 | -1.23 | -1.32 | Gm867 |
| 10534889 | 0.03 | 0.11 | -1.23 | -1.09 | Agfg2 |
| 10528385 | -0.05 | -0.11 | -1.22 | -1.40 | Reln |
| 10580210 | -0.10 | -0.18 | -1.22 | -1.31 | Rad23a |
| 10585586 | -0.06 | -0.12 | -1.22 | -0.73 | Ube2s |
| 10516823 | 0.02 | -0.04 | -1.22 | -0.83 | Epb41 |
| 10586491 | -0.10 | -0.04 | -1.21 | -1.03 | Dapk2 |
| 10475610 | -0.12 | -0.18 | -1.21 | -0.77 | Dut |
| 10380514 | 0.01 | -0.01 | -1.21 | -1.28 | Fam117a |
| 10478962 | -0.06 | -0.06 | -1.21 | -1.15 | Fam210b |
| 10408085 | -0.01 | 0.20 | -1.20 | -0.63 | Hist1h2an |
| 10511258 | -0.10 | -0.19 | -1.20 | -1.65 | Fam132a |
| 10552681 | -0.41 | -0.45 | -1.20 | -1.44 | Josd2 |
| 10479765 | -0.12 | -0.29 | -1.20 | -0.90 | Suv39h2 |
| 10339336 | -0.22 | 0.07 | -1.19 | -1.38 | Unidentified |
| 10602090 | 0.28 | 0.33 | -1.19 | -1.00 | Atg4a |
| 10368886 | -0.03 | 0.12 | -1.19 | -0.71 | Foxo3 |
| 10430993 | 0.04 | -0.13 | -1.19 | -0.76 | 1700001L05Rik |
| 10605370 | 0.16 | 0.04 | -1.19 | -0.77 | Mpp1 |
| 10595407 | -0.03 | -0.11 | -1.18 | -0.88 | Ibtk |
| 10570321 | 0.03 | -0.13 | -1.18 | -0.86 | Cul4a |
| 10479041 | 0.15 | 0.28 | -1.18 | -0.83 | Rbm38 |
| 10401359 | -0.22 | -0.43 | -1.18 | -1.14 | Dpf3 |
| 10462504 | -0.06 | -0.05 | -1.18 | -0.90 | Minpp1 |
| 10425078 | -0.05 | -0.05 | -1.18 | -0.86 | Mpst |
| 10396610 | -0.05 | -0.18 | -1.17 | -0.90 | Mthfd1 |
| 10462791 | -0.11 | -0.03 | -1.17 | -0.90 | March5 |
| 10588028 | 0.05 | 0.01 | -1.16 | -0.78 | Nmnat3 |
| 10391540 | -0.06 | -0.37 | -1.16 | -1.19 | Mpp2 |
| 10588899 | 0.30 | 0.45 | -1.16 | -1.05 | Gpx1 |
| 10500802 | 0.39 | 0.38 | -1.14 | -0.99 | Atg4a |
| 10556302 | 0.48 | 0.70 | -1.14 | -0.75 | Ampd3 |
| 10532248 | 0.00 | -0.16 | -1.14 | -1.14 | Rnf212 |
| 10405733 | -0.16 | -0.27 | -1.14 | -1.08 | Zfp808 |
| 10419465 | -0.19 | -0.13 | -1.13 | -1.48 | Ccnb1ip1 |
| 10376096 | -0.20 | -0.20 | -1.13 | -1.09 | Acsl6 |
| 10507851 | -0.10 | -0.05 | -1.13 | -0.95 | Pabpc4 |
| 10608716 | 0.02 | -0.05 | -1.12 | -1.45 | Unidentified |
| 10488575 | 0.11 | 0.15 | -1.12 | -1.02 | Psmf1 |
| 10574682 | -0.08 | -0.03 | -1.12 | -1.09 | E2f4 |
| 10572097 | 0.03 | -0.12 | -1.11 | -1.05 | Sh2d4a |
| 10376455 | -0.01 | -0.05 | -1.11 | -1.05 | Hist3h2a |
| 10443421 | 0.03 | -0.15 | -1.11 | -1.07 | Brpf3 |
| 10340887 | -0.60 | 0.29 | -1.10 | -2.03 | Unidentified |
| 10360090 | -0.12 | -0.30 | -1.10 | -1.23 | Ppox |
| 10433088 | -0.04 | -0.18 | -1.10 | -1.09 | Cbx5 |
| 10587266 | 0.10 | 0.00 | -1.08 | -1.07 | Gclc |
| 10512747 | -0.04 | -0.09 | -1.07 | -1.10 | Trmo |
| 10361906 | -0.13 | -0.14 | -1.05 | -1.20 | Il22ra2 |
| 10382802 | 0.18 | 0.23 | -1.05 | -1.08 | Sphk1 |
| 10474201 | 0.01 | 0.06 | -1.05 | -1.26 | Lmo2 |
| 10465395 | 0.07 | -0.06 | -1.03 | -1.53 | Ppp2r5b |
| 10460251 | -0.43 | 0.01 | -1.03 | -1.23 | Aldh3b3 |
| 10597996 | 0.09 | 0.14 | -0.95 | -1.55 | Xcr1 |
| 10519060 | 0.14 | 0.77 | -0.92 | -1.13 | Tnfrsf14 |
| 10339813 | 0.24 | 0.54 | -0.79 | -2.10 | Unidentified |

**Supplemental Table 15.** List of genes in cluster 35

|  | **Log ratio** | | | |  |
| --- | --- | --- | --- | --- | --- |
| **Probe Set ID** | **A.SW_Brain** | **SJL_Brain** | **A.SW_Spleen** | **SJL_Spleen** | **Symbol** |
| 10569020 | 0.40 | 0.99 | -0.69 | 2.80 | Ifitm6 |
| 10581605 | 0.12 | 0.99 | -0.77 | 2.77 | Hp |
| 10466224 | 0.09 | 0.15 | -1.70 | 2.73 | Ms4a3 |
| 10505438 | 0.09 | 0.38 | -0.51 | 2.59 | Orm1 |
| 10356886 | -0.08 | -0.39 | -1.16 | 2.59 | Slco4c1 |
| 10466606 | 1.07 | 1.10 | -1.23 | 2.36 | Anxa1 |
| 10597098 | -0.04 | 0.13 | -2.97 | 2.31 | Camp |
| 10420261 | 0.00 | 0.12 | -2.56 | 2.28 | Ctsg |
| 10493831 | 0.24 | 1.20 | -2.40 | 2.27 | S100a8 |
| 10374248 | -0.07 | -0.33 | -1.06 | 2.25 | Abca13 |
| 10364529 | -0.04 | 0.03 | -1.75 | 2.23 | Prtn3 |
| 10560886 | 0.05 | 0.21 | -1.40 | 2.17 | Cd177 |
| 10499861 | 0.29 | 1.26 | -2.05 | 2.16 | S100a9 |
| 10361507 | -0.18 | -0.38 | -0.42 | 2.12 | Unidentified |
| 10563712 | 0.33 | 0.53 | -0.92 | 2.10 | Mrgpra2b |
| 10563715 | 0.33 | 0.48 | -0.94 | 2.01 | Mrgpra2b |
| 10376332 | 0.00 | 0.15 | -0.92 | 2.00 | 4930438A08Rik |
| 10580382 | -0.25 | -0.40 | -0.53 | 1.96 | Neto2 |
| 10341004 | -0.65 | -0.31 | -0.71 | 1.93 | Unidentified |
| 10537509 | 0.02 | 0.12 | -0.94 | 1.89 | Mgam |
| 10444016 | 0.04 | 0.14 | -0.55 | 1.89 | Pram1 |
| 10445753 | 0.03 | 0.48 | -1.04 | 1.89 | Trem3 |
| 10379636 | 0.15 | 1.00 | -0.92 | 1.85 | Slfn4 |
| 10380174 | -0.03 | 0.35 | -2.73 | 1.82 | Mpo |
| 10589703 | 0.10 | 0.41 | -3.37 | 1.79 | Ltf |
| 10364535 | -0.04 | 0.23 | -2.39 | 1.70 | Elane |
| 10550509 | 0.34 | 1.14 | -0.79 | 1.69 | Pglyrp1 |
| 10478633 | -0.05 | 0.49 | -0.89 | 1.68 | Mmp9 |
| 10574276 | 0.17 | 0.19 | -0.89 | 1.66 | Adgrg3 |
| 10400844 | 0.18 | 0.66 | -0.47 | 1.66 | Pygl |
| 10351224 | 0.18 | -0.07 | -1.33 | 1.64 | F5 |
| 10541599 | 0.01 | 0.01 | -0.45 | 1.63 | Clec4b2 |
| 10429580 | 0.11 | 1.26 | -0.86 | 1.62 | Ly6a |
| 10542172 | 0.18 | -0.01 | -0.85 | 1.55 | Clec1b |
| 10416689 | 0.01 | 0.04 | -1.72 | 1.54 | Olfm4 |
| 10421970 | -0.23 | -0.68 | -1.41 | 1.53 | Unidentified |
| 10387821 | -0.07 | 0.03 | -1.24 | 1.51 | Alox12 |
| 10448278 | 0.15 | 0.42 | -0.83 | 1.50 | Mmp25 |
| 10529824 | 0.17 | 0.01 | -0.47 | 1.47 | Prom1 |
| 10351206 | 0.44 | 1.71 | -1.28 | 1.47 | Selp |
| 10538706 | -0.10 | -0.15 | -1.93 | 1.46 | Mmrn1 |
| 10481262 | -0.11 | 0.00 | -1.25 | 1.45 | Fcnb |
| 10343814 | -1.36 | -1.54 | -1.58 | 1.44 | Unidentified |
| 10391697 | -0.04 | 0.20 | -1.02 | 1.38 | Itga2b |
| 10472350 | 0.02 | -0.24 | -0.51 | 1.38 | Gca |
| 10421517 | 0.12 | 0.47 | -0.58 | 1.37 | Cysltr2 |
| 10450412 | 0.18 | -0.22 | -1.21 | 1.32 | G6b |
| 10438358 | -0.20 | -0.18 | -0.82 | 1.32 | Sept5 |
| 10589535 | -0.11 | 1.80 | -2.64 | 1.30 | Ngp |
| 10541587 | 0.36 | 0.64 | -0.47 | 1.29 | Clec4a2 |
| 10586433 | 0.11 | 0.00 | -0.88 | 1.28 | Rbpms2 |
| 10424683 | -0.07 | 0.47 | -3.37 | 1.28 | Ly6g |
| 10479154 | 0.03 | -0.02 | -1.60 | 1.28 | Tubb1 |
| 10495794 | -0.44 | -0.24 | -1.07 | 1.27 | Pde5a |
| 10381809 | -0.07 | 0.02 | -0.98 | 1.26 | Itgb3 |
| 10338935 | 0.00 | 0.23 | -0.98 | 1.26 | Unidentified |
| 10548385 | 0.43 | 0.84 | -1.26 | 1.25 | Olr1 |
| 10566943 | -0.06 | -0.08 | -0.63 | 1.23 | Mrvi1 |
| 10403743 | -0.18 | 0.39 | -0.62 | 1.23 | Inhba |
| 10459620 | -0.12 | -0.29 | -1.60 | 1.23 | Rab27b |
| 10340069 | 0.48 | -0.31 | -0.67 | 1.22 | Unidentified |
| 10547056 | -0.01 | 0.03 | -0.93 | 1.22 | Tmem40 |
| 10358421 | 0.46 | 0.35 | -1.29 | 1.20 | Rgs18 |
| 10467115 | 0.00 | 0.00 | -0.76 | 1.19 | Ankrd22 |
| 10542164 | 0.69 | 1.47 | -1.27 | 1.19 | Clec12a |
| 10523134 | 0.40 | -0.03 | -1.29 | 1.18 | Pf4 |
| 10475567 | -0.13 | -0.01 | -1.13 | 1.18 | Slc24a5 |
| 10378240 | -0.04 | 0.27 | -0.81 | 1.18 | P2rx1 |
| 10583163 | -0.25 | -0.38 | -1.36 | 1.17 | Trpc6 |
| 10515755 | -0.02 | -0.08 | -1.26 | 1.17 | Mpl |
| 10433101 | 0.41 | 0.65 | -0.47 | 1.16 | Gpr84 |
| 10438907 | -0.07 | -0.01 | -1.06 | 1.16 | Gp5 |
| 10377924 | 0.04 | -0.01 | -1.11 | 1.14 | Gp1ba |
| 10428376 | 0.25 | 0.06 | -1.05 | 1.14 | Angpt1 |
| 10378816 | -0.04 | -0.11 | -1.21 | 1.12 | Slc6a4 |
| 10541910 | 0.66 | 1.31 | -1.17 | 1.12 | Vwf |
| 10451646 | 0.06 | 0.12 | -0.53 | 1.11 | A530064D06Rik |
| 10421950 | -0.31 | -0.32 | -1.01 | 1.08 | Dach1 |
| 10438639 | 0.00 | 0.00 | -0.87 | 1.07 | Dgkg |
| 10548030 | 0.63 | 0.72 | -0.69 | 1.07 | Cd9 |
| 10406736 | 0.05 | 0.07 | -1.20 | 1.04 | F2rl2 |
| 10363962 | -0.17 | -0.29 | -1.27 | 1.02 | Gnaz |
| 10379685 | -0.11 | 0.02 | -0.62 | 1.02 | 1700020L24Rik |
| 10521757 | -0.01 | -0.06 | -1.09 | 1.02 | Unidentified |
| 10438891 | 0.07 | 0.01 | -1.40 | 1.00 | Gm1968 |
| 10342857 | -0.10 | -0.10 | -1.01 | 0.99 | Unidentified |
| 10341366 | -0.18 | 0.30 | -1.27 | 0.99 | Unidentified |
| 10545245 | 0.20 | 0.18 | -1.39 | 0.91 | Igkv8-16 |
| 10349157 | 0.09 | 0.22 | -1.01 | 0.91 | Serpinb2 |
| 10407511 | -0.11 | 0.06 | -1.11 | 0.91 | Unidentified |
| 10492330 | 0.11 | -0.10 | -1.20 | 0.89 | P2ry1 |
| 10341518 | 0.02 | -0.34 | -1.02 | 0.89 | Unidentified |
| 10489463 | 0.40 | 0.45 | -0.74 | 0.88 | Slpi |
| 10513869 | 0.09 | 0.08 | -0.93 | 0.87 | Megf9 |
| 10451547 | -0.07 | 0.10 | -1.23 | 0.85 | Gm16494 |
| 10349166 | 0.16 | 0.04 | -1.96 | 0.84 | Serpinb10 |
| 10539818 | -0.02 | -0.18 | -1.49 | 0.82 | Gp9 |
| 10514275 | 0.48 | 0.60 | -0.80 | 0.81 | Hacd4 |
| 10343985 | -0.18 | -0.31 | -1.12 | 0.80 | Unidentified |
| 10413059 | 0.12 | -0.04 | -0.96 | 0.79 | Vcl |
| 10341578 | 0.21 | -0.26 | -1.00 | 0.79 | Unidentified |
| 10404059 | -0.10 | 0.88 | -0.86 | 0.78 | Hist1h1c |
| 10341771 | -0.50 | -0.94 | -1.29 | 0.77 | Unidentified |
| 10591773 | -0.09 | 0.03 | -1.13 | 0.72 | Hmgn2 |
| 10403108 | -0.12 | 0.04 | -1.11 | 0.71 | Hmgn2 |
| 10579659 | -0.11 | 0.02 | -1.11 | 0.70 | Hmgn2 |
| 10517141 | -0.12 | 0.03 | -1.10 | 0.68 | Hmgn2 |
| 10343241 | -0.11 | -0.06 | -1.02 | 0.68 | Unidentified |
| 10420254 | 0.04 | 0.00 | -1.27 | 0.60 | Mcpt8 |
| 10340246 | -0.18 | -0.93 | -1.42 | 0.60 | Unidentified |
| 10342166 | -0.47 | 1.38 | -1.06 | 0.57 | Unidentified |
| 10526564 | -0.08 | -0.14 | -1.63 | 0.50 | Ufsp1 |
| 10592061 | 0.05 | 0.02 | -1.28 | 0.50 | Kcnj5 |
| 10425852 | 0.04 | 0.20 | -1.65 | 0.47 | Parvb |

**Supplemental Table 16.** Top and bottom 100 genes listed in factor loading for PC1 of brain PCA

| **Gene ID** | **Factor loading** | **Gene symbol** | **Gene name** |
| --- | --- | --- | --- |
| 10481627 | 0.09144541 | *Lcn2* | lipocalin 2 |
| 10523359 | 0.07658287 | *Cxcl13* | chemokine (C-X-C motif) ligand 13 |
| 10379727 | 0.07086484 | *Wfdc17* | WAP four-disulfide core domain 17 |
| 10456005 | 0.07020096 | *Cd74* | CD74 antigen (invariant polypeptide of major histocompatibility complex, class II antigen-associated) |
| 10563597 | 0.06976034 | *Saa3* | serum amyloid A 3 |
| 10501020 | 0.06570622 | *Chil3* | chitinase-like 3 |
| 10545235 | 0.06530789 | *Igkv8-28* | immunoglobulin kappa variable 8-28 |
| 10403031 | 0.06252016 | *Ighv1-55* | immunoglobulin heavy variable 1-55 |
| 10603551 | 0.0618363 | *Cybb* | cytochrome b-245, beta polypeptide |
| 10450154 | 0.06168648 | *H2-Aa* | histocompatibility 2, class II antigen A, alpha |
| 10461614 | 0.06105505 | *Ms4a6c* | membrane-spanning 4-domains, subfamily A, member 6C |
| 10455970 | 0.06057554 | *BC023105* | interferon-inducible GTPase-like [Mus musculus] |
| 10363070 | 0.06022403 | *Gp49a* | glycoprotein 49 A |
| 10389231 | 0.05927723 | *Ccl3* | chemokine (C-C motif) ligand 3 |
| 10598976 | 0.05807999 | *Timp1* | tissue inhibitor of metalloproteinase 1 |
| 10444298 | 0.0574205 | *H2-Eb1* | histocompatibility 2, class II antigen E beta |
| 10452316 | 0.05706301 | *C3* | complement component 3 |
| 10372648 | 0.05688917 | *Lyz2* | lysozyme 2 |
| 10416837 | 0.05566243 | *Irg1* | immunoresponsive gene 1 |
| 10429560 | 0.05433952 | *Ly6i* | lymphocyte antigen 6 complex, locus I |
| 10389207 | 0.05408186 | *Ccl5* | chemokine (C-C motif) ligand 5 |
| 10444291 | 0.05320995 | *H2-Ab1* | histocompatibility 2, class II antigen A, beta 1 |
| 10363082 | 0.05214165 | *Lilrb4* | leukocyte immunoglobulin-like receptor, subfamily B, member 4 |
| 10547664 | 0.05209053 | *Clec4e* | C-type lectin domain family 4, member e |
| 10455961 | 0.05198574 | *Iigp1* | interferon inducible GTPase 1 |
| 10531407 | 0.05110502 | *Cxcl9* | chemokine (C-X-C motif) ligand 9 |
| 10608681 | 0.05095739 | Unidentified | Unmapped full-length transcript |
| 10398075 | 0.05087674 | *Serpina3n* | serine (or cysteine) peptidase inhibitor, clade A, member 3N |
| 10466210 | 0.0507553 | *Ms4a6d* | membrane-spanning 4-domains, subfamily A, member 6D |
| 10567580 | 0.04990691 | *Igsf6* | immunoglobulin superfamily, member 6 |
| 10545198 | 0.04958497 | *Igkv4-59* | immunoglobulin kappa variable 4-59 |
| 10375608 | 0.04924839 | *Scgb3a1* | secretoglobin, family 3A, member 1 |
| 10360382 | 0.04917767 | *Ifi204* | interferon activated gene 204 |
| 10450075 | 0.04909518 | *H2-K1* | histocompatibility 2, K1, K region |
| 10455954 | 0.04889664 | *Gm4951* | predicted gene 4951 |
| 10545190 | 0.04835753 | *Igkv4-69* | immunoglobulin kappa variable 4-69 |
| 10389222 | 0.04789478 | *Ccl6* | chemokine (C-C motif) ligand 6 |
| 10496592 | 0.04771558 | *Gbp2* | guanylate binding protein 2 |
| 10606016 | 0.04742427 | *Il2rg* | interleukin 2 receptor, gamma chain |
| 10466127 | 0.04724158 | *AW112010* | expressed sequence AW112010 |
| 10349968 | 0.04681194 | *Chil1* | chitinase-like 1 |
| 10538187 | 0.04602073 | *Gpnmb* | glycoprotein (transmembrane) nmb |
| 10375121 | 0.04598108 | *Gm10462* | predicted gene 10462 |
| 10444824 | 0.04523983 | *H2-Q6* | histocompatibility 2, Q region locus 6 |
| 10548375 | 0.04477914 | *Clec7a* | C-type lectin domain family 7, member a |
| 10545247 | 0.04477313 | *Igkv6-15* | immunoglobulin kappa variable 6-15 |
| 10360377 | 0.04416054 | *AI607873* | expressed sequence AI607873 |
| 10375123 | 0.04407064 | *Gm10462* | predicted gene 10462 |
| 10425066 | 0.04383602 | *Csf2rb* | colony stimulating factor 2 receptor, beta, low-affinity (granulocyte-macrophage) |
| 10420114 | 0.0437672 | *Tgm1* | transglutaminase 1, K polypeptide |
| 10385500 | 0.04368628 | *Irgm1* | immunity-related GTPase family M member 1 |
| 10461587 | 0.04368502 | *Ms4a4a* | membrane-spanning 4-domains, subfamily A, member 4A |
| 10541614 | 0.04347014 | *Clec4d* | C-type lectin domain family 4, member d |
| 10405587 | 0.04333338 | *Tgfbi* | transforming growth factor, beta induced |
| 10523156 | 0.04315106 | *Cxcl2* | chemokine (C-X-C motif) ligand 2 |
| 10476945 | 0.04282127 | *Cst7* | cystatin F (leukocystatin) |
| 10526410 | 0.04224808 | *Hspb1* | heat shock protein 1 |
| 10538871 | 0.04198979 | *Igkv2-137* | immunoglobulin kappa chain variable 2-137 |
| 10408928 | 0.0416694 | *Hspb1* | heat shock protein 1 |
| 10351509 | 0.04149926 | *Fcgr4* | Fc receptor, IgG, low affinity IV |
| 10545196 | 0.0414641 | *Igkc* | immunoglobulin kappa constant |
| 10462390 | 0.04137137 | *Cd274* | CD274 antigen (B7-H1/PD-L1) |
| 10545184 | 0.04125156 | *Igkv4-74* | immunoglobulin kappa variable 4-74 |
| 10521667 | 0.04096324 | *Bst1* | bone marrow stromal cell antigen 1 |
| 10444780 | 0.04080693 | *H2-D1* | histocompatibility 2, D region locus 1 |
| 10487588 | 0.04079974 | *Il1a* | interleukin 1 alpha |
| 10511779 | 0.0406017 | *Atp6v0d2* | ATPase, H+ transporting, lysosomal V0 subunit D2 |
| 10517165 | 0.04034464 | *Cd52* | CD52 antigen (B7/CLS1) |
| 10451953 | 0.03987596 | *Lrg1* | leucine-rich alpha-2-glycoprotein 1 |
| 10444830 | 0.0396172 | *H2-Q7* | histocompatibility 2, Q region locus 7 |
| 10531994 | 0.03929947 | *Gbp6* | guanylate binding protein 6 |
| 10517513 | 0.0391423 | *C1qc* | complement component 1, q subcomponent, C chain |
| 10484463 | 0.0390012 | *Serping1* | serine (or cysteine) peptidase inhibitor, clade G, member 1 |
| 10360028 | 0.03890225 | *Fcgr2b* | Fc receptor, IgG, low affinity Iib |
| 10385518 | 0.03866313 | *Tgtp1* | T cell specific GTPase 1 |
| 10461721 | 0.03864644 | *Mpeg1* | macrophage expressed gene 1 |
| 10385533 | 0.03861305 | *Tgtp1* | T cell specific GTPase 1 |
| 10458382 | 0.03845302 | *Cd14* | CD14 antigen |
| 10545187 | 0.03835943 | *Igkv4-70* | immunoglobulin kappa chain variable 4-70 |
| 10349648 | 0.03831325 | *Ctse* | cathepsin E |
| 10578264 | 0.03827811 | *Msr1* | macrophage scavenger receptor 1 |
| 10408693 | 0.03806782 | *F13a1* | coagulation factor XIII, A1 subunit |
| 10554789 | 0.03778697 | *Ctsc* | cathepsin C |
| 10531415 | 0.0376659 | *Cxcl10* | chemokine (C-X-C motif) ligand 10 (IP-10) |
| 10547740 | 0.03755243 | *C1s1* | complement component 1, s subcomponent 1 |
| 10469786 | 0.03751927 | *Il1f9* | interleukin 1 family, member 9 |
| 10583100 | 0.0373017 | *Mmp8* | matrix metallopeptidase 8 |
| 10403015 | 0.03703454 | *Ighv1-22* | immunoglobulin heavy variable 1-22 |
| 10494271 | 0.0369459 | *Ctss* | cathepsin S |
| 10574098 | 0.03688683 | *Nlrc5* | NLR family, CARD domain containing 5 |
| 10582303 | 0.03682595 | *Cyba* | cytochrome b-245, alpha polypeptide |
| 10376326 | 0.03681167 | *Igtp* | interferon gamma induced GTPase |
| 10347291 | 0.03679469 | *Cxcr2* | chemokine (C-X-C motif) receptor 2 (CD128/IL8RA) |
| 10478048 | 0.03655047 | *Lbp* | lipopolysaccharide binding protein |
| 10358879 | 0.03640882 | *Npl* | N-acetylneuraminate pyruvate lyase |
| 10487597 | 0.03634421 | *Il1b* | interleukin 1 beta |
| 10534667 | 0.03616654 | *Serpine1* | serine (or cysteine) peptidase inhibitor, clade E, member 1 |
| 10364262 | 0.03612694 | *Itgb2* | integrin beta 2 (Cd18) |
| 10360406 | 0.03608386 | *Ifi205* | interferon activated gene 205 |
| 10473384 | 0.03594406 | *Slc43a3* | solute carrier family 43, member 3 |
| 10482814 | -0.011457223 | *Acvr1c* | activin A receptor, type IC |
| 10351430 | -0.011475261 | *Rxrg* | retinoid X receptor gamma |
| 10607484 | -0.0114994 | *Ptchd1* | patched domain containing 1 |
| 10401320 | -0.011531112 | *Adam4* | a disintegrin and metallopeptidase domain 4 |
| 10590438 | -0.011596102 | *Fam198a* | family with sequence similarity 198, member A |
| 10482762 | -0.011629824 | *Idi1* | isopentenyl-diphosphate delta isomerase |
| 10436658 | -0.011658155 | *7120432I05Rik* | RIKEN cDNA 7120432I05 gene |
| 10373680 | -0.01169593 | *Neurod4* | neurogenic differentiation 4 |
| 10595668 | -0.011868625 | *Ankrd34c* | ankyrin repeat domain 34C |
| 10442037 | -0.011884864 | *Zfp97* | zinc finger protein 97 |
| 10541191 | -0.011913981 | *Rad52* | RAD52 homolog, DNA repair protein |
| 10485745 | -0.011954329 | *Ano3* | anoctamin 3 |
| 10517587 | -0.011973508 | *Alpl* | alkaline phosphatase, liver/bone/kidney |
| 10479176 | -0.011980455 | *Gm6710* | predicted gene 6710 |
| 10584578 | -0.011984817 | *Hspa8* | heat shock protein 8 |
| 10374777 | -0.012001333 | *Efemp1* | epidermal growth factor-containing fibulin-like extracellular matrix protein 1 |
| 10362422 | -0.012014505 | *Trdn* | triadin |
| 10395389 | -0.012059392 | *Sostdc1* | sclerostin domain containing 1 |
| 10604375 | -0.012178288 | *Apln* | apelin |
| 10440329 | -0.012267747 | *9330155M09Rik* | RIKEN cDNA 9330155M09 gene |
| 10429491 | -0.01228189 | *Arc* | activity regulated cytoskeletal-associated protein |
| 10351551 | -0.012286358 | *Adamts4* | a disintegrin-like and metallopeptidase (reprolysin type) with thrombospondin type 1 motif, 4 |
| 10606257 | -0.012298561 | *Mir384* | microRNA 384 |
| 10423230 | -0.012359561 | *Cdh9* | cadherin 9 |
| 10536405 | -0.01236625 | *Nxph1* | neurexophilin 1 |
| 10571599 | -0.012409984 |  | Mus musculus strain C57BL/6J chromosome 8, GRCm38.p4 C57BL/6J |
| 10447688 | -0.012498421 | *4930506C21Rik* | RIKEN cDNA 4930506C21 gene |
| 10490302 | -0.012525144 | *Zfp931* | zinc finger protein 931 |
| 10412500 | -0.012527837 | *Gm8237* | predicted gene 8237 |
| 10461115 | -0.012557023 | *Slc22a8* | solute carrier family 22 (organic anion transporter), member 8 |
| 10462442 | -0.012618362 | *Il33* | interleukin 33 |
| 10469613 | -0.012625453 | *Thnsl1* | threonine synthase-like 1 (bacterial) |
| 10510239 | -0.012773894 | *Gm16503* | predicted gene 16503 |
| 10512354 | -0.012778399 |  | Mus musculus predicted gene 2163 (Gm2163), misc_RNA |
| 10563858 | -0.012832523 | *Gabrg3* | gamma-aminobutyric acid (GABA) A receptor, subunit gamma 3 |
| 10566258 | -0.012852084 | *Hbb-b1* | hemoglobin, beta adult major chain |
| 10474373 | -0.012852815 | *Kcna4* | potassium voltage-gated channel, shaker-related subfamily, member 4 |
| 10549265 | -0.012939943 | *Lmntd1* | lamin tail domain containing 1 |
| 10398362 | -0.012991022 | *AF357355* | snoRNA AF357355 |
| 10563094 | -0.013045937 | *Fcgrt* | Fc receptor, IgG, alpha chain transporter |
| 10485357 | -0.0130647 | *Gm10800* | predicted gene 10800 |
| 10501963 | -0.013124467 | *Ugt8a* | UDP galactosyltransferase 8A |
| 10498620 | -0.013222146 | *Trim59* | tripartite motif-containing 59 |
| 10560818 | -0.013241027 | *Zfp111* | zinc finger protein 111 |
| 10467529 | -0.013275153 | *Opalin* | oligodendrocytic myelin paranodal and inner loop protein |
| 10410644 | -0.013288097 | *Zfp72* | zinc finger protein 72 |
| 10381798 | -0.01332155 | *Myl4* | myosin, light polypeptide 4 |
| 10393642 | -0.013368816 | *Eif4a3* | eukaryotic translation initiation factor 4A3 |
| 10373692 | -0.013657703 | *Vmn2r87* | vomeronasal 2, receptor 87 |
| 10446713 | -0.013723267 | *Snord53* | small nucleolar RNA, C/D box 53 |
| 10571752 | -0.013735272 | *Dctd* | dCMP deaminase |
| 10398356 | -0.013931132 | *Gm24899* | predicted gene, 24899 |
| 10499189 | -0.013958867 | *Fcrls* | Fc receptor-like S, scavenger receptor |
| 10469573 | -0.014017744 | *Gm25859* | predicted gene, 25859 |
| 10459602 | -0.014064694 | *Ptpn2* | protein tyrosine phosphatase, non-receptor type 2 |
| 10555935 | -0.014253635 | *Cckbr* | cholecystokinin B receptor |
| 10515335 | -0.014376005 | *C530005A16Rik* | RIKEN cDNA C530005A16 gene |
| 10479228 | -0.014541239 | *Etohi1* | ethanol induced 1 |
| 10373684 | -0.014606457 | *Vmn2r85* | vomeronasal 2, receptor 85 |
| 10598794 | -0.014679986 |  | desmoglein4 [Mus musculus] |
| 10566254 | -0.014715872 | *Hbb-b1* | hemoglobin, beta adult major chain |
| 10584580 | -0.014943703 | *Hspa8* | heat shock protein 8 |
| 10528287 | -0.015184919 | *Rpl31-ps21* | ribosomal protein L31, pseudogene 21 |
| 10375058 | -0.015309303 | *Hba-a2* | hemoglobin alpha, adult chain 2 |
| 10375051 | -0.015327746 | *Hba-a1* | hemoglobin alpha, adult chain 1 |
| 10579049 | -0.01573291 | *Gm10033* | predicted gene 10033 |
| 10407467 | -0.015779948 | *Akr1e1* | aldo-keto reductase family 1, member E1 |
| 10353102 | -0.015784947 | *Cpa6* | carboxypeptidase A6 |
| 10469577 | -0.015810973 |  | Mus musculus strain C57BL/6J chromosome 2, GRCm38.p4 C57BL/6J |
| 10479185 | -0.015836466 |  | mCG145033, partial [Mus musculus] |
| 10514713 | -0.015957402 | *Wdr78* | WD repeat domain 78 |
| 10531944 | -0.016099124 | *Gm10047* | predicted gene 10047 |
| 10408557 | -0.016207217 | *Serpinb1a* | serine (or cysteine) peptidase inhibitor, clade B, member 1a |
| 10362440 | -0.016470308 | *Trdn* | triadin |
| 10559818 | -0.016544876 |  | Mus musculus retrotransposon ETnII-beta, complete sequence |
| 10490289 | -0.016671494 |  | zinc finger protein family member [Mus musculus] |
| 10579052 | -0.016848788 | *Gm10033* | predicted gene 10033 |
| 10436476 | -0.016983641 | *Pou1f1* | POU domain, class 1, transcription factor 1 |
| 10490276 | -0.017007142 | *Gm14432* | predicted gene 14432 |
| 10448312 | -0.017842565 | *Cldn9* | claudin 9 |
| 10457038 | -0.017969358 |  | Mus musculus zinc finger protein 236 (Zfp236), transcript variant X10, mRNA |
| 10464905 | -0.018135722 | *Npas4* | neuronal PAS domain protein 4 |
| 10392522 | -0.019261352 | *Abca8a* | ATP-binding cassette, sub-family A (ABC1), member 8a |
| 10573865 | -0.019723049 | *Gm3579* | predicted gene 3579 |
| 10375559 | -0.020531495 |  | Mus musculus ETn-related retroelement Young MusD 5, partial sequence |
| 10514491 | -0.020847702 | *Cyp2j12* | cytochrome P450, family 2, subfamily j, polypeptide 12 |
| 10504203 | -0.022417312 | *4930578G10Rik* | RIKEN cDNA 4930578G10 gene |
| 10572739 | -0.022453466 |  | pol protein, partial [Mus musculus] |
| 10512350 | -0.02252675 | *4933409K07Rik* | RIKEN cDNA 4933409K07 gene |
| 10400948 | -0.023033459 | *4930447C04Rik* | RIKEN cDNA 4930447C04 gene |
| 10479192 | -0.023918623 | *Gm14434* | predicted gene 14434 |
| 10479195 | -0.023918623 | *Gm14305* | predicted gene 14305 |
| 10535559 | -0.024146816 | *Baiap2l1* | BAI1-associated protein 2-like 1 |
| 10352104 | -0.024172838 | *Gm16432* | predicted gene 16432 |
| 10503947 | -0.026675593 | *Cga* | glycoprotein hormones, alpha subunit |
| 10568731 | -0.027938476 |  | Mus musculus retrotransposon ETnII-beta, complete sequence |
| 10399588 | -0.029575724 | *Zfp125* | zinc finger protein 125 |
| 10392135 | -0.034520409 | *Gh* | growth hormone |
| 10421524 | -0.03495152 |  | Mus musculus LTR retrotransposon DNA, MusD element Dac2J insertion |
| 10404264 | -0.059951369 | *Prl* | prolactin |

**Supplemental Table 17.** Top and bottom 100 genes listed in factor loading for PC2 of brain PCA

| **Gene ID** | **Factor loading** | **Gene** |  |
| --- | --- | --- | --- |
| 10504203 | 0.075205662 | 4930578G10Rik | RIKEN cDNA 4930578G10 gene |
| 10582845 | 0.06930994 |  | TPA_exp: pro protein, partial [Mus musculus] |
| 10508800 | 0.062305524 | Gm3579 | predicted gene 3579 |
| 10568731 | 0.059271538 |  | Fas antigen - mouse (fragment) |
| 10538187 | 0.058323706 | Gpnmb | glycoprotein (transmembrane) nmb |
| 10504201 | 0.056483363 | 4933409K07Rik | RIKEN cDNA 4933409K07 gene |
| 10481627 | 0.0563081 | Lcn2 | lipocalin 2 |
| 10551998 | 0.05282181 |  | envelope protein precursor [Polytropic murine leukemia virus] |
| 10372648 | 0.050955273 | Lyz2 | lysozyme 2 |
| 10559818 | 0.049630019 |  | Fas antigen - mouse (fragment) |
| 10399588 | 0.04910495 | Zfp125 | zinc finger protein 125 |
| 10461721 | 0.044353252 | Mpeg1 | macrophage expressed gene 1 |
| 10535559 | 0.043168359 | Baiap2l1 | BAI1-associated protein 2-like 1 |
| 10446713 | 0.042888968 | Snord53 | small nucleolar RNA, C/D box 53 |
| 10511779 | 0.040542672 | Atp6v0d2 | ATPase, H+ transporting, lysosomal V0 subunit D2 |
| 10400948 | 0.039980536 | 4930447C04Rik | RIKEN cDNA 4930447C04 gene |
| 10389231 | 0.039965126 | Ccl3 | chemokine (C-C motif) ligand 3 |
| 10457038 | 0.039781189 |  | Mus musculus zinc finger protein 236 (Zfp236), transcript variant X10, mRNA |
| 10572739 | 0.039237598 |  | TPA_exp: pol protein, partial [Mus musculus] |
| 10363070 | 0.038137944 | Lilr4b | leukocyte immunoglobulin-like receptor, subfamily B, member 4B |
| 10582545 | 0.038056434 | Mela | melanoma antigen |
| 10512332 | 0.037078501 |  | unnamed protein product [Mus musculus] |
| 10450694 | 0.036413924 | H2-T22 | histocompatibility 2, T region locus 22 |
| 10523717 | 0.035815203 | Spp1 | secreted phosphoprotein 1 |
| 10531944 | 0.035781963 | Gm10047 | predicted gene 10047 |
| 10398075 | 0.035652736 | Serpina3n | serine (or cysteine) peptidase inhibitor, clade A, member 3N |
| 10547657 | 0.035311383 | C3ar1 | complement component 3a receptor 1 |
| 10523359 | 0.035279936 | Cxcl13 | chemokine (C-X-C motif) ligand 13 |
| 10353102 | 0.035204259 | Cpa6 | carboxypeptidase A6 |
| 10563094 | 0.034666325 | Fcgrt | Fc receptor, IgG, alpha chain transporter |
| 10479198 | 0.034591863 | Gm14434 | predicted gene 14434 |
| 10490273 | 0.034591863 | Gm14305 | predicted gene 14305 |
| 10352104 | 0.034033216 | Gm16432 | predicted gene 16432 |
| 10541191 | 0.033602788 | Rad52 | RAD52 homolog, DNA repair protein |
| 10496077 | 0.031878896 | Etnppl | ethanolamine phosphate phospholyase |
| 10528287 | 0.03178668 | Rpl31-ps21 | ribosomal protein L31, pseudogene 21 |
| 10538921 | 0.029514578 | Igkv1-110 | immunoglobulin kappa variable 1-110 |
| 10490248 | 0.029125276 |  | mCG145033, partial [Mus musculus] |
| 10393642 | 0.02834049 | Eif4a3 | eukaryotic translation initiation factor 4A3 |
| 10584458 | 0.028231127 | Olfr920 | olfactory receptor 920 |
| 10399581 | 0.027756427 | 3110053B16Rik | RIKEN cDNA 3110053B16 gene |
| 10548375 | 0.027720906 | Clec7a | C-type lectin domain family 7, member a (dectin-1) |
| 10563858 | 0.027430665 | Gabrg3 | gamma-aminobutyric acid (GABA) A receptor, subunit gamma 3 |
| 10387536 | 0.027331862 | Cd68 | CD68 antigen |
| 10494271 | 0.027281263 | Ctss | cathepsin S |
| 10598794 | 0.02693037 |  | desmoglein4 [Mus musculus] |
| 10448312 | 0.02664207 | Cldn9 | claudin 9 |
| 10510239 | 0.026274138 | Gm16503 | predicted gene 16503 |
| 10517513 | 0.025852542 | C1qc | complement component 1, q subcomponent, C chain |
| 10601385 | 0.025832761 | Tlr13 | toll-like receptor 13 |
| 10404606 | 0.025762751 | Ly86 | lymphocyte antigen 86 |
| 10441933 | 0.025567588 | Ermard | ER membrane associated RNA degradation |
| 10517508 | 0.025166945 | C1qb | complement component 1, q subcomponent, beta polypeptide |
| 10411082 | 0.024880604 | Thbs4 | thrombospondin 4 |
| 10479203 | 0.024742277 | Gm14432 | predicted gene 14432 |
| 10490289 | 0.024585856 | Gm14411 | predicted gene 14411 |
| 10562709 | 0.024516919 | Cd33 | CD33 antigen |
| 10441952 | 0.024490126 | Ermard | ER membrane associated RNA degradation |
| 10509122 | 0.024322852 | Cnr2 | cannabinoid receptor 2 (macrophage) |
| 10402195 | 0.02414464 | Tc2n | tandem C2 domains, nuclear |
| 10545231 | 0.02382402 | Igkv6-32 | immunoglobulin kappa variable 6-32 |
| 10487588 | 0.0238098 | Il1a | interleukin 1 alpha |
| 10356461 | 0.023792856 | Hjurp | Holliday junction recognition protein |
| 10502042 | 0.02363651 | Alpk1 | alpha-kinase 1 |
| 10455080 | 0.023635648 | Pcdhb9 | protocadherin beta 9 |
| 10384458 | 0.023263868 | Plek | pleckstrin |
| 10510221 | 0.022967916 | Gm3579 | predicted gene 3579 |
| 10491846 | 0.022733656 | Gm22043 | predicted gene, 22043 |
| 10379727 | 0.022431249 | Wfdc17 | WAP four-disulfide core domain 17 |
| 10587799 | 0.022409258 | Plscr2 | phospholipid scramblase 2 |
| 10551883 | 0.022082499 | Tyrobp | TYRO protein tyrosine kinase binding protein |
| 10567580 | 0.021934028 | Igsf6 | immunoglobulin superfamily, member 6 |
| 10461587 | 0.02175018 | Ms4a4a | membrane-spanning 4-domains, subfamily A, member 4A |
| 10514491 | 0.021721929 | Cyp2j12 | cytochrome P450, family 2, subfamily j, polypeptide 12 |
| 10350742 | 0.021704239 | Rnasel | ribonuclease L (2', 5'-oligoisoadenylate synthetase-dependent) |
| 10502196 | 0.021590953 | Rpl34 | ribosomal protein L34 |
| 10531126 | 0.021455921 | Jchain | immunoglobulin joining chain |
| 10399584 | 0.021200486 | G730007D18Rik | RIKEN cDNA G730007D18 gene |
| 10587383 | 0.021172936 | Cd109 | CD109 antigen |
| 10469322 | 0.021068553 | Vim | vimentin |
| 10545220 | 0.020890216 | Igkv12-44 | immunoglobulin kappa variable 12-44 |
| 10448016 | 0.020733462 | Tcte3 | t-complex-associated testis expressed 3 |
| 10603551 | 0.020668357 | Cybb | cytochrome b-245, beta polypeptide |
| 10363082 | 0.020519287 | Lilrb4a | leukocyte immunoglobulin-like receptor, subfamily B, member 4A |
| 10398348 | 0.02042611 | Mir136 | microRNA 136 |
| 10403034 | 0.020406994 | Ighv8-8 | immunoglobulin heavy variable 8-8 |
| 10466210 | 0.019739061 | Ms4a6d | membrane-spanning 4-domains, subfamily A, member 6D |
| 10445781 | 0.01973381 | Trem2 | triggering receptor expressed on myeloid cells 2 |
| 10448023 | 0.019662503 | Tcte3 | t-complex-associated testis expressed 3 |
| 10473384 | 0.019487993 | Slc43a3 | solute carrier family 43, member 3 |
| 10428336 | 0.019469249 | Gm25381 | predicted gene, 25381 |
| 10501063 | 0.019402232 | Cd53 | CD53 antigen |
| 10414360 | 0.019399048 | Lgals3 | lectin, galactose binding, soluble 3 |
| 10446282 | 0.019361218 | Adgre1 | adhesion G protein-coupled receptor E1 |
| 10517517 | 0.019306444 | C1qa | complement component 1, q subcomponent, alpha polypeptide |
| 10538880 | 0.019298382 | Igkv1-117 | immunoglobulin kappa variable 1-117 |
| 10403063 | 0.019264631 | Ighv8-12 | immunoglobulin heavy variable V8-12 |
| 10586484 | 0.019187384 | Fam96a | family with sequence similarity 96, member A |
| 10527441 | 0.019130315 | Arpc1b | actin related protein 2/3 complex, subunit 1B |
| 10427471 | 0.01908515 | Osmr | oncostatin M receptor |
| 10512315 | -0.016332971 | Ccl27a | chemokine (C-C motif) ligand 27A |
| 10576749 | -0.016384569 | Pcp2 | Purkinje cell protein 2 (L7) |
| 10589994 | -0.016538956 | Eomes | eomesodermin |
| 10417452 | -0.016625853 | 4930555G01Rik | RIKEN cDNA 4930555G01 gene |
| 10417446 | -0.016625853 | D830030K20Rik | RIKEN cDNA D830030K20 gene |
| 10506296 | -0.016873783 |  | Mus musculus strain C57BL/6J chromosome 4, GRCm38.p4 C57BL/6J |
| 10373846 | -0.016927633 | Sec14l3 | SEC14-like lipid binding 3 |
| 10423496 | -0.017168146 | Gm24487 | predicted gene, 24487 |
| 10583887 | -0.01718431 | Npsr1 | neuropeptide S receptor 1 |
| 10501963 | -0.01729458 | Ugt8a | UDP galactosyltransferase 8A |
| 10379731 | -0.017407029 | Wfdc18 | WAP four-disulfide core domain 18 |
| 10351504 | -0.017532163 | AI506816 | expressed sequence AI506816 |
| 10451828 | -0.017539808 | Sult1c1 | sulfotransferase family, cytosolic, 1C, member 1 |
| 10461909 | -0.017718423 | Nmrk1 | nicotinamide riboside kinase 1 |
| 10427035 | -0.018052124 | Nr4a1 | nuclear receptor subfamily 4, group A, member 1 |
| 10380411 | -0.018167191 | Mrpl27 | mitochondrial ribosomal protein L27 |
| 10376579 | -0.018583229 | Drc3 | dynein regulatory complex subunit 3 |
| 10505982 | -0.018604965 | Fggy | FGGY carbohydrate kinase domain containing |
| 10358879 | -0.018659581 | Npl | N-acetylneuraminate pyruvate lyase |
| 10511429 | -0.018691614 | Car8 | carbonic anhydrase 8 |
| 10346485 | -0.018732054 | Aox2 | aldehyde oxidase 2 |
| 10522904 | -0.018783302 | 2310003L06Rik | RIKEN cDNA 2310003L06 gene |
| 10351131 | -0.018915289 | Myoc | myocilin |
| 10504865 | -0.019011632 | Invs | inversin |
| 10510254 | -0.019028843 | Fv1 | Friend virus susceptibility 1 |
| 10412488 | -0.01905893 | Gm3468 | predicted gene 3468 |
| 10567879 | -0.019081913 | Atp2a1 | ATPase, Ca++ transporting, cardiac muscle, fast twitch 1 |
| 10479550 | -0.019214798 | Ppdpf | pancreatic progenitor cell differentiation and proliferation factor |
| 10493235 | -0.019305773 | Paqr6 | progestin and adipoQ receptor family member VI |
| 10395356 | -0.019546261 | Agr3 | anterior gradient 3 |
| 10363735 | -0.019598754 | Egr2 | early growth response 2 |
| 10504838 | -0.019651478 | Nr4a3 | nuclear receptor subfamily 4, group A, member 3 |
| 10398406 | -0.019862208 | Mir376b | microRNA 376b |
| 10477406 | -0.019880948 | Bpifb3 | BPI fold containing family B, member 3 |
| 10513514 | -0.020014206 | Mup5 | major urinary protein 5 |
| 10464905 | -0.020148018 | Npas4 | neuronal PAS domain protein 4 |
| 10565705 | -0.020518075 | Omp | olfactory marker protein |
| 10468877 | -0.020650423 |  | envelope protein [Mus musculus] |
| 10600057 | -0.020687318 | Cnga2 | cyclic nucleotide gated channel alpha 2 |
| 10361234 | -0.021145718 | Hsd11b1 | hydroxysteroid 11-beta dehydrogenase 1 |
| 10417371 | -0.021326796 |  | Mus musculus predicted gene 3696 (Gm3696), mRNA |
| 10402390 | -0.021595302 | Serpina1b | serine (or cysteine) preptidase inhibitor, clade A, member 1B |
| 10379677 | -0.02190141 | Rasl10b | RAS-like, family 10, member B |
| 10505998 | -0.021948324 | Fggy | FGGY carbohydrate kinase domain containing |
| 10453553 | -0.021955861 | 2610044O15Rik8 | RIKEN cDNA 2610044O15 gene |
| 10416897 | -0.022263891 | Gm22290 | predicted gene, 22290 |
| 10493816 | -0.022364293 | S100a5 | S100 calcium binding protein A5 |
| 10470200 | -0.022434772 | Lcn11 | lipocalin 11 |
| 10470186 | -0.023266386 | Obp2b | odorant binding protein 2B |
| 10400326 | -0.023832415 | Eapp | E2F-associated phosphoprotein |
| 10429491 | -0.02384186 | Arc | activity regulated cytoskeletal-associated protein |
| 10358541 | -0.024004593 | Hmcn1 | hemicentin 1 |
| 10545569 | -0.02411421 | Reg3g | regenerating islet-derived 3 gamma |
| 10478048 | -0.024492715 | Lbp | lipopolysaccharide binding protein |
| 10542223 | -0.024644475 | 5430401F13Rik | RIKEN cDNA 5430401F13 gene |
| 10593981 | -0.024755935 | Cyp1a2 | cytochrome P450, family 1, subfamily a, polypeptide 2 |
| 10447100 | -0.025860024 | Morn2 | MORN repeat containing 2 |
| 10477423 | -0.025996144 | Bpifb4 | BPI fold containing family B, member 4 |
| 10404264 | -0.026401895 | Prl | prolactin |
| 10498584 | -0.026685798 | Rarres1 | retinoic acid receptor responder (tazarotene induced) 1 |
| 10447108 | -0.026867666 | Arhgef33 | Rho guanine nucleotide exchange factor (GEF) 33 |
| 10358533 | -0.027289689 | Hmcn1 | hemicentin 1 |
| 10531100 | -0.027703069 | Sult1d1 | sulfotransferase family 1D, member 1 |
| 10394819 | -0.028001207 | Gm40847 | predicted gene, 40847 |
| 10393936 | -0.029556872 | Cbr2 | carbonyl reductase 2 |
| 10440340 | -0.030932186 |  | Mus musculus strain C57BL/6J chromosome 16, GRCm38.p4 C57BL/6J |
| 10551293 | -0.031152767 | Cyp2f2 | cytochrome P450, family 2, subfamily f, polypeptide 2 |
| 10415081 | -0.031353212 | Gm41154 | predicted gene, 41154 |
| 10586076 | -0.031353212 | Gm40525 | predicted gene, 40525 |
| 10520121 | -0.031353212 |  | envelope protein [Mus musculus] |
| 10578405 | -0.031353212 | Gm40498 | predicted gene, 40498 |
| 10608668 | -0.032963105 |  | Unmapped full-length transcript |
| 10398442 | -0.033721855 | Mir410 | microRNA 410 |
| 10513412 | -0.034062884 | Mup4 | major urinary protein 4 |
| 10504123 | -0.034793609 | Gm2163 | predicted gene 2163 |
| 10504169 | -0.035068026 | 4933409K07Rik | RIKEN cDNA 4933409K07 gene |
| 10504178 | -0.035600337 | Gm3893 | predicted gene 3893 |
| 10524098 | -0.036084872 | Zfp605 | zinc finger protein 605 |
| 10437646 | -0.036436146 | Tekt5 | tektin 5 |
| 10601588 | -0.036605953 | Cldn34c1 | claudin 34C1 |
| 10522902 | -0.036628966 | BC051076 | cDNA sequence BC051076 |
| 10552888 | -0.036869138 | Slc6a16 | solute carrier family 6, member 16 |
| 10403911 | -0.037218853 | Gpx6 | glutathione peroxidase 6 |
| 10551282 | -0.037956982 | Cyp2a5 | cytochrome P450, family 2, subfamily a, polypeptide 5 |
| 10531078 | -0.038286644 | Ugt2a1 | UDP glucuronosyltransferase 2 family, polypeptide A1 |
| 10477528 | -0.038357775 | Bpifb9a | BPI fold containing family B, member 9A |
| 10347218 | -0.042432308 | Gm25360 | predicted gene, 25360 |
| 10558694 | -0.042475043 | Scgb1c1 | secretoglobin, family 1C, member 1 |
| 10477475 | -0.045612339 | Bpifa1 | BPI fold containing family A, member 1 |
| 10491993 | -0.046014174 | Stoml3 | stomatin (Epb7.2)-like 3 |
| 10557058 | -0.047037601 | Polr3e | polymerase (RNA) III (DNA directed) polypeptide E |
| 10551226 | -0.048874983 | Cyp2a4 | cytochrome P450, family 2, subfamily a, polypeptide 4 |
| 10605488 | -0.052475061 | Obp1a | odorant binding protein IA |
| 10470175 | -0.053139572 | Obp2a | odorant binding protein 2A |
| 10600576 | -0.055985192 | Gm14743 | predicted gene 14743 |
| 10600568 | -0.056886876 | 5430402E10Rik | RIKEN cDNA 5430402E10 gene |
| 10605482 | -0.06002632 | Gm14744 | predicted gene 14744 |
| 10551273 | -0.077333102 | Cyp2g1 | cytochrome P450, family 2, subfamily g, polypeptide 1 |
| 10477536 | -0.087993738 | Bpifb9b | BPI fold containing family B, member 9B |
| 10375121 | -0.093463612 | Gm10462 | predicted gene 10462 |

**Supplemental Table 18.** Top and bottom 100 genes listed in factor loading for PC1 of spleen PCA

| **Gene ID** | **Factor loading** | **Gene symbol** | **Gene name** |
| --- | --- | --- | --- |
| 10435497 | 0.044162 | Stfa2l1 | stefin A2 like 1 |
| 10558159 | 0.043589 | Dmbt1 | deleted in malignant brain tumors 1 |
| 10544333 | 0.041419 | Try5 | trypsin 5 |
| 10543017 | 0.040876 | Pdk4 | pyruvate dehydrogenase kinase, isoenzyme 4 |
| 10537638 | 0.039354 | Try10 | trypsin 10 |
| 10464313 | 0.038711 | Pnliprp1 | pancreatic lipase related protein 1 |
| 10537014 | 0.038656 | Cpa2 | carboxypeptidase A2, pancreatic |
| 10464328 | 0.038289 | Pnliprp2 | pancreatic lipase-related protein 2 |
| 10398075 | 0.038132 | Serpina3n | serine (or cysteine) peptidase inhibitor, clade A, member 3N |
| 10567366 | 0.036976 | Gp2 | glycoprotein 2 (zymogen granule membrane) |
| 10517573 | 0.036211 | Cela3b | chymotrypsin-like elastase family, member 3B |
| 10497463 | 0.03339 | Cpb1 | carboxypeptidase B1 (tissue) |
| 10464298 | 0.032574 | Pnlip | pancreatic lipase |
| 10402435 | 0.032045 | Serpina3c | serine (or cysteine) peptidase inhibitor, clade A, member 3C |
| 10439292 | 0.031598 | BC100530 | cDNA sequence BC100530 |
| 10543762 | 0.031276 | Tsga13 | testis specific gene A13 |
| 10544326 | 0.030935 | 2210010C04Rik | RIKEN cDNA 2210010C04 gene |
| 10458894 | 0.030917 | Lox | lysyl oxidase |
| 10481278 | 0.030722 | Cel | carboxyl ester lipase |
| 10552546 | 0.030695 | Klk1 | kallikrein 1 |
| 10501494 | 0.030658 | Amy2a5 | amylase 2a5 |
| 10568165 | 0.030219 | Zg16 | zymogen granule protein 16 |
| 10539200 | 0.02985 | Reg1 | regenerating islet-derived 1 |
| 10461587 | 0.029324 | Ms4a4a | membrane-spanning 4-domains, subfamily A, member 4A |
| 10345752 | 0.0292 | Il1r2 | interleukin 1 receptor, type II |
| 10518059 | 0.02896 | Ctrc | chymotrypsin C (caldecrin) |
| 10501544 | 0.028793 | Amy2a4 | amylase 2a4 |
| 10518050 | 0.02833 | Cela2a | chymotrypsin-like elastase family, member 2A |
| 10458704 | 0.028303 | Spink1 | serine peptidase inhibitor, Kazal type 1 |
| 10506301 | 0.028284 | Lepr | leptin receptor |
| 10537051 | 0.028229 | Cpa1 | carboxypeptidase A1, pancreatic |
| 10581882 | 0.028118 | Ctrb1 | chymotrypsinogen B1 |
| 10524698 | 0.027984 | Pla2g1b | phospholipase A2, group IB, pancreas |
| 10432652 | 0.027974 | Cela1 | chymotrypsin-like elastase family, member 1 |
| 10581355 | 0.027892 | Ctrl | chymotrypsin-like |
| 10537627 | 0.027836 | Prss2 | protease, serine 2 |
| 10551531 | 0.027609 | Sycn | syncollin |
| 10419578 | 0.026809 | Ndrg2 | N-myc downstream regulated gene 2 |
| 10539194 | 0.026719 | Reg2 | regenerating islet-derived 2 |
| 10510574 | 0.026663 | Errfi1 | ERBB receptor feedback inhibitor 1 |
| 10568502 | 0.026226 | Cuzd1 | CUB and zona pellucida-like domains 1 |
| 10449467 | 0.026212 | Clps | colipase, pancreatic |
| 10449452 | 0.025931 | Fkbp5 | FK506 binding protein 5 |
| 10512279 | 0.025826 | Cntfr | ciliary neurotrophic factor receptor |
| 10598041 | 0.025676 | mt-Tk | mitochondrially encoded tRNA lysine |
| 10419563 | 0.025625 | Rnase1 | ribonuclease, RNase A family, 1 (pancreatic) |
| 10359582 | 0.025356 | Fmo2 | flavin containing monooxygenase 2 |
| 10439296 | 0.025128 | Stfa2 | stefin A2 |
| 10369290 | 0.024873 | Ddit4 | DNA-damage-inducible transcript 4 |
| 10593225 | 0.024651 | Zbtb16 | zinc finger and BTB domain containing 16 |
| 10392522 | 0.024617 | Abca8a | ATP-binding cassette, sub-family A (ABC1), member 8a |
| 10538890 | 0.024446 |  | vSAG(M12), partial [Mouse mammary tumor virus] |
| 10482528 | 0.024351 | Neb | nebulin |
| 10376201 | 0.024036 | Gpx3 | glutathione peroxidase 3 |
| 10373740 | 0.023975 | Pik3ip1 | phosphoinositide-3-kinase interacting protein 1 |
| 10537645 | 0.023814 | Gm5409 | predicted pseudogene 5409 |
| 10537634 | 0.023346 | Try4 | trypsin 4 |
| 10545561 | 0.023063 | Reg3d | regenerating islet-derived 3 delta |
| 10559796 | 0.022942 | Peg3 | paternally expressed 3 |
| 10602896 | 0.022896 | Adgrg2 | adhesion G protein-coupled receptor G2 |
| 10427436 | 0.022689 | C7 | complement component 7 |
| 10522503 | 0.022647 | Pdgfra | platelet derived growth factor receptor, alpha polypeptide |
| 10401931 | 0.022353 | Gm40902 | predicted gene, 40902 |
| 10401937 | 0.022353 | BC005685 | cDNA sequence BC005685 |
| 10414192 | 0.022294 | Mat1a | methionine adenosyltransferase I, alpha |
| 10537650 | 0.021932 | Prss1 | protease, serine 1 (trypsin 1) |
| 10567995 | 0.021777 | Nupr1 | nuclear protein transcription regulator 1 |
| 10606989 | 0.021769 | Tsc22d3 | TSC22 domain family, member 3 |
| 10501020 | 0.021702 | Chil3 | chitinase-like 3 |
| 10552618 | 0.021665 | Klk1b5 | kallikrein 1-related peptidase b5 |
| 10552587 | 0.021252 | Klk1b21 | kallikrein 1-related peptidase b21 |
| 10501555 | 0.021129 | Amy1 | amylase 1, salivary |
| 10538892 | 0.021088 | LOC105243827 | uncharacterized LOC105243827 |
| 10498741 | 0.02105 | Serpini2 | serine (or cysteine) peptidase inhibitor, clade I, member 2 |
| 10504753 | 0.020853 |  | uncharacterized protein LOC105247125 [Mus musculus] |
| 10517568 | 0.020816 | Cela3a | chymotrypsin-like elastase family, member 3A |
| 10462091 | 0.020697 | Klf9 | Kruppel-like factor 9 |
| 10578136 | 0.020597 |  | Mus musculus strain C57BL/6J chromosome 8, GRCm38.p4 C57BL/6J |
| 10449661 | 0.020586 | Tff2 | trefoil factor 2 (spasmolytic protein 1) |
| 10538187 | 0.020468 | Gpnmb | glycoprotein (transmembrane) nmb |
| 10430851 | 0.020398 | Cyp2d22 | cytochrome P450, family 2, subfamily d, polypeptide 22 |
| 10451953 | 0.020373 | Lrg1 | leucine-rich alpha-2-glycoprotein 1 |
| 10504761 | 0.020365 | LOC105247125 | uncharacterized LOC105247125 |
| 10345065 | 0.020196 | Gsta3 | glutathione S-transferase, alpha 3 |
| 10452815 | 0.020145 | Xdh | xanthine dehydrogenase |
| 10439009 | 0.020142 | Apod | apolipoprotein D |
| 10509063 | 0.019909 | Il22ra1 | interleukin 22 receptor, alpha 1 |
| 10538901 | 0.019508 | BC005685 | cDNA sequence BC005685 |
| 10449245 | 0.019494 | Pdia2 | protein disulfide isomerase associated 2 |
| 10377439 | 0.019249 | Per1 | period circadian clock 1 |
| 10568001 | 0.018928 | Sult1a1 | sulfotransferase family 1A, phenol-preferring, member 1 |
| 10571865 | 0.018798 | Scrg1 | scrapie responsive gene 1 |
| 10537561 | 0.018667 | 1810009J06Rik | RIKEN cDNA 1810009J06 gene |
| 10433885 | 0.018609 | Cebpd | CCAAT/enhancer binding protein (C/EBP), delta |
| 10358339 | 0.018564 | Cfh | complement component factor h |
| 10427628 | 0.018541 | Il7r | interleukin 7 receptor |
| 10504203 | 0.018473 | 4930578G10Rik | RIKEN cDNA 4930578G10 gene |
| 10341419 | 0.01844 |  | Intronic normalization control (Negative Control) |
| 10604175 | 0.018407 | Tmem255a | transmembrane protein 255A |
| 10494643 | 0.018257 | Hmgcs2 | 3-hydroxy-3-methylglutaryl-Coenzyme A synthase 2 |
| 10425037 | -0.02911 | Apol10a | apolipoprotein L 10A |
| 10497520 | -0.02914 | Ect2 | ect2 oncogene |
| 10400589 | -0.02914 | Mis18bp1 | MIS18 binding protein 1 |
| 10414315 | -0.02918 | Cdkn3 | cyclin-dependent kinase inhibitor 3 |
| 10480891 | -0.02929 | Ubac1 | ubiquitin associated domain containing 1 |
| 10357698 | -0.0293 | Tmcc2 | transmembrane and coiled-coil domains 2 |
| 10594774 | -0.02942 | Ccnb2 | cyclin B2 |
| 10550131 | -0.02954 | Pla2g4c | phospholipase A2, group IVC (cytosolic, calcium-independent) |
| 10506822 | -0.02964 | Orc1 | origin recognition complex, subunit 1 |
| 10591781 | -0.02964 | Anln | anillin, actin binding protein |
| 10521731 | -0.02971 | Ncapg | non-SMC condensin I complex, subunit G |
| 10509168 | -0.02972 | E2f2 | E2F transcription factor 2 |
| 10497831 | -0.02972 | Ccna2 | cyclin A2 |
| 10391811 | -0.02976 | Kif18b | kinesin family member 18B |
| 10554240 | -0.02983 | Isg20 | interferon-stimulated protein |
| 10350392 | -0.02988 | Aspm | asp (abnormal spindle)-like, microcephaly associated (Drosophila) |
| 10459391 | -0.0299 | Fech | ferrochelatase |
| 10390707 | -0.02995 | Top2a | topoisomerase (DNA) II alpha |
| 10578690 | -0.03003 | Neil3 | nei like 3 (E. coli) |
| 10359861 | -0.03006 | Mgst3 | microsomal glutathione S-transferase 3 |
| 10404069 | -0.03023 | Hist1h1a | histone cluster 1, H1a |
| 10359890 | -0.03032 | Nuf2 | NUF2, NDC80 kinetochore complex component |
| 10507500 | -0.03034 | Slc6a9 | solute carrier family 6 (neurotransmitter transporter, glycine), member 9 |
| 10350335 | -0.03055 | Hmbs | hydroxymethylbilane synthase |
| 10562637 | -0.03056 | Ccnb1 | cyclin B1 |
| 10563883 | -0.03096 | Depdc1a | DEP domain containing 1a |
| 10364784 | -0.03099 | Reep6 | receptor accessory protein 6 |
| 10487480 | -0.03109 | Bub1 | BUB1, mitotic checkpoint serine/threonine kinase |
| 10406968 | -0.03109 | Cenpk | centromere protein K |
| 10360985 | -0.03111 | Cenpf | centromere protein F |
| 10376813 | -0.03126 | Specc1 | sperm antigen with calponin homology and coiled-coil domains 1 |
| 10562563 | -0.0313 | Ccne1 | cyclin E1 |
| 10568714 | -0.03133 | Mki67 | antigen identified by monoclonal antibody Ki 67 |
| 10563780 | -0.03164 | E2f8 | E2F transcription factor 8 |
| 10537179 | -0.03187 | Bpgm | 2,3-bisphosphoglycerate mutase |
| 10545086 | -0.03189 | Snca | synuclein, alpha |
| 10554445 | -0.03197 | Prc1 | protein regulator of cytokinesis 1 |
| 10436392 | -0.0322 | Cpox | coproporphyrinogen oxidase |
| 10367024 | -0.03221 | Tac2 | tachykinin 2 |
| 10404063 | -0.03256 | Hist1h2ab | histone cluster 1, H2ab |
| 10497122 | -0.03257 | Depdc1a | DEP domain containing 1a |
| 10394978 | -0.03266 | Rrm2 | ribonucleotide reductase M2 |
| 10383556 | -0.03269 | Fn3krp | fructosamine 3 kinase related protein |
| 10350297 | -0.03292 | Kif14 | kinesin family member 14 |
| 10462796 | -0.03298 | Kif11 | kinesin family member 11 |
| 10503264 | -0.03306 | Ccne2 | cyclin E2 |
| 10592772 | -0.03347 | Abcg4 | ATP-binding cassette, sub-family G (WHITE), member 4 |
| 10474984 | -0.03365 | Nusap1 | nucleolar and spindle associated protein 1 |
| 10493382 | -0.034 | Pklr | pyruvate kinase liver and red blood cell |
| 10567108 | -0.03449 | Sox6 | SRY (sex determining region Y)-box 6 |
| 10582626 | -0.0346 | Abcb10 | ATP-binding cassette, sub-family B (MDR/TAP), member 10 |
| 10539669 | -0.03469 | Add2 | adducin 2 (beta) |
| 10582545 | -0.03484 | Mela | melanoma antigen |
| 10574572 | -0.03492 | Ces2g | carboxylesterase 2G |
| 10538459 | -0.03508 | Aqp1 | aquaporin 1 |
| 10385248 | -0.03522 | Hmmr | hyaluronan mediated motility receptor (RHAMM) |
| 10384956 | -0.03549 | Chac2 | ChaC, cation transport regulator 2 |
| 10420877 | -0.03568 | Esco2 | establishment of sister chromatid cohesion N-acetyltransferase 2 |
| 10435075 | -0.03601 | Tfrc | transferrin receptor |
| 10536908 | -0.03613 | Tspan33 | tetraspanin 33 |
| 10596166 | -0.03616 | 1300017J02Rik | RIKEN cDNA 1300017J02 gene |
| 10389300 | -0.03618 | Dhrs11 | dehydrogenase/reductase (SDR family) member 11 |
| 10598507 | -0.03742 | Slc38a5 | solute carrier family 38, member 5 |
| 10421418 | -0.03753 | Dmtn | dematin actin binding protein |
| 10383564 | -0.03772 | Fn3k | fructosamine 3 kinase |
| 10592816 | -0.03814 | Hmbs | hydroxymethylbilane synthase |
| 10602372 | -0.0387 | Alas2 | aminolevulinic acid synthase 2, erythroid |
| 10385826 | -0.03904 | Sowaha | sosondowah ankyrin repeat domain family member A |
| 10567564 | -0.03987 | Cdr2 | cerebellar degeneration-related 2 |
| 10401068 | -0.04079 | Sptb | spectrin beta, erythrocytic |
| 10376434 | -0.04099 | Btnl10 | butyrophilin-like 10 |
| 10605542 | -0.04102 | Mageb16 | melanoma antigen family B, 16 |
| 10473367 | -0.0413 | Slc43a1 | solute carrier family 43, member 1 |
| 10573457 | -0.04168 | Klf1 | Kruppel-like factor 1 (erythroid) |
| 10490923 | -0.04184 | Car2 | carbonic anhydrase 2 |
| 10504692 | -0.04212 | Tmod1 | tropomodulin 1 |
| 10473349 | -0.04253 | Ypel4 | yippee-like 4 (Drosophila) |
| 10399710 | -0.04341 | Rsad2 | radical S-adenosyl methionine domain containing 2 |
| 10451670 | -0.04355 | Tspo2 | translocator protein 2 |
| 10515848 | -0.04453 | Ermap | erythroblast membrane-associated protein |
| 10379026 | -0.04522 | Mir144 | microRNA 144 |
| 10545096 | -0.04567 | Mageb16 | melanoma antigen family B, 16 |
| 10400483 | -0.04587 | Slc25a21 | solute carrier family 25 (mitochondrial oxodicarboxylate carrier), member 21 |
| 10499062 | -0.04686 | Fhdc1 | FH2 domain containing 1 |
| 10366446 | -0.04724 | Tspan8 | tetraspanin 8 |
| 10423971 | -0.04885 | Pkhd1l1 | polycystic kidney and hepatic disease 1-like 1 |
| 10570894 | -0.04934 | Ank1 | ankyrin 1, erythroid |
| 10445046 | -0.05029 | Trim10 | tripartite motif-containing 10 |
| 10501802 | -0.05174 | Tmem56 | transmembrane protein 56 |
| 10351905 | -0.05211 | Spta1 | spectrin alpha, erythrocytic 1 |
| 10512757 | -0.05217 | Hemgn | hemogen |
| 10391649 | -0.05278 | Slc4a1 | solute carrier family 4 (anion exchanger), member 1 |
| 10593198 | -0.05285 | Nxpe2 | neurexophilin and PC-esterase domain family, member 2 |
| 10486664 | -0.05369 | Epb42 | erythrocyte membrane protein band 4.2 |
| 10445192 | -0.0538 | Rhag | Rhesus blood group-associated A glycoprotein |
| 10534389 | -0.05622 | Cldn13 | claudin 13 |
| 10608710 | -0.05935 |  | Unmapped full-length transcript |
| 10509002 | -0.06069 | Rhd | Rh blood group, D antigen |
| 10544383 | -0.06134 | Kel | Kell blood group |
| 10573054 | -0.06193 | Gypa | glycophorin A |

**Supplemental Table 19.** Top and bottom 100 genes listed in factor loading for PC2 of spleen PCA

| **Gene ID** | **Factor loading** | **Gene symbol** | **Gene name** | |
| --- | --- | --- | --- | --- |
| 10502552 | 0.08926487 | Clca3a1 | chloride channel accessory 3A1 |  |
| 10421524 | 0.075483785 |  | Mus musculus LTR retrotransposon DNA, MusD element Dac2J insertion |  |
| 10604961 | 0.073990234 | Gabra3 | gamma-aminobutyric acid (GABA) A receptor, subunit alpha 3 |  |
| 10502565 | 0.069961791 | Clca3a2 | chloride channel accessory 3A2 |  |
| 10399588 | 0.060376742 | Zfp125 | zinc finger protein 125 |  |
| 10502801 | 0.05939054 | Ifi44l | interferon-induced protein 44 like |  |
| 10528287 | 0.058346554 | Rpl31-ps21 | ribosomal protein L31, pseudogene 21 |  |
| 10538878 | 0.055147279 | Igkv11-125 | immunoglobulin kappa variable 11-125 |  |
| 10600069 | 0.050174988 | Gabrq | gamma-aminobutyric acid (GABA) A receptor, subunit theta |  |
| 10435745 | 0.049864746 | Gm16498 | predicted gene 16498 |  |
| 10420247 | 0.047883684 | Mcpt4 | mast cell protease 4 |  |
| 10490913 | 0.046689797 | Car3 | carbonic anhydrase 3 |  |
| 10435743 | 0.044508878 | Gm8609 | predicted gene 8609 |  |
| 10589632 | 0.043260675 | Prss45 | protease, serine 45 |  |
| 10358928 | 0.042272404 | Cacna1e | calcium channel, voltage-dependent, R type, alpha 1E subunit |  |
| 10504203 | 0.042188135 | 4930578G10Rik | RIKEN cDNA 4930578G10 gene |  |
| 10497451 | 0.041042356 | Cpa3 | carboxypeptidase A3, mast cell |  |
| 10479192 | 0.040747766 | Gm14434 | predicted gene 14434 |  |
| 10479195 | 0.040747766 | Gm14305 | predicted gene 14305 |  |
| 10448312 | 0.040605941 | Cldn9 | claudin 9 |  |
| 10388086 | 0.03997054 | Nlrp1c-ps | NLR family, pyrin domain containing 1C, pseudogene |  |
| 10403006 | 0.038789996 | Ighv3-5 | immunoglobulin heavy variable 3-5 |  |
| 10450694 | 0.037122279 | H2-T22 | histocompatibility 2, T region locus 22 |  |
| 10477600 | 0.036831197 | a | nonagouti |  |
| 10492774 | 0.036767084 | Dchs2 | dachsous 2 (Drosophila) |  |
| 10512350 | 0.035298557 | 4933409K07Rik | RIKEN cDNA 4933409K07 gene |  |
| 10414953 | 0.035220218 | Trav15-1-dv6-1 | T cell receptor alpha variable 15-1-DV6-1 |  |
| 10543967 | 0.034628445 | Dgki | diacylglycerol kinase, iota |  |
| 10502791 | 0.033834145 | Ifi44 | interferon-induced protein 44 |  |
| 10446713 | 0.033624548 | Snord53 | small nucleolar RNA, C/D box 53 |  |
| 10402195 | 0.031773656 | Tc2n | tandem C2 domains, nuclear |  |
| 10604944 | 0.031733688 | Gabre | gamma-aminobutyric acid (GABA) A receptor, subunit epsilon |  |
| 10548535 | 0.03154118 | Klra3 | killer cell lectin-like receptor, subfamily A, member 3 |  |
| 10392834 | 0.031300492 | Cd300ld5 | CD300 molecule like family member D5 |  |
| 10392839 | 0.031254455 | Cd300e | CD300E molecule |  |
| 10598794 | 0.030874815 |  | Mus musculus LTR retrotransposon DNA, MusD element Dac2J insertion |  |
| 10541191 | 0.030698258 | Rad52 | RAD52 homolog, DNA repair protein |  |
| 10352110 | 0.030525097 | Gm16432 | predicted gene 16432 |  |
| 10563749 | 0.029641795 | Mrgprb1 | MAS-related GPR, member B1 |  |
| 10568731 | 0.02926966 |  | Mus musculus retrotransposon ETnII-beta |  |
| 10388065 | 0.02909045 | Nlrp1b | NLR family, pyrin domain containing 1B |  |
| 10439321 | 0.028875933 | Slc15a2 | solute carrier family 15 (H+/peptide transporter), member 2 |  |
| 10445758 | 0.027286963 | Treml4 | triggering receptor expressed on myeloid cells-like 4 |  |
| 10351477 | 0.027022064 | Sh2d1b1 | SH2 domain containing 1B1 |  |
| 10572739 | 0.026993948 |  | Mus musculus chromosome 8, clone RP23-314A15 |  |
| 10582545 | 0.026679143 | Mela | melanoma antigen |  |
| 10527878 | 0.026155172 | V1rg10 | vomeronasal 1 receptor, G10 |  |
| 10399584 | 0.026011009 | G730007D18Rik | RIKEN cDNA G730007D18 gene |  |
| 10502042 | 0.02587068 | Alpk1 | alpha-kinase 1 |  |
| 10469577 | 0.025612606 |  | Mus musculus chromosome 2, clone RPCI-22-207E02 strain 129/Sv |  |
| 10548396 | 0.025495455 | Klrc3 | killer cell lectin-like receptor subfamily C, member 3 |  |
| 10559818 | 0.025164497 |  | Mus musculus retrotransposon ETnII-beta |  |
| 10549990 | 0.024376104 | V1rg10 | vomeronasal 1 receptor, G10 |  |
| 10500559 | 0.024097998 | Hsd3b6 | hydroxy-delta-5-steroid dehydrogenase, 3 beta- and steroid delta-isomerase 6 |  |
| 10565819 | 0.023775813 | Slco2b1 | solute carrier organic anion transporter family, member 2b1 |  |
| 10355310 | 0.023595971 | Gm8840 | predicted gene 8840 |  |
| 10601569 | 0.023537558 | Pcdh11x | protocadherin 11 X-linked |  |
| 10475910 | 0.023532755 | Gm23172 | predicted gene, 23172 |  |
| 10548525 | 0.023503782 | Klra10 | killer cell lectin-like receptor subfamily A, member 10 |  |
| 10546762 | 0.023356602 | Il5ra | interleukin 5 receptor, alpha |  |
| 10442115 | 0.023311726 | Vmn2r96 | vomeronasal 2, receptor 96 |  |
| 10590628 | 0.023249128 | Ccr3 | chemokine (C-C motif) receptor 3 |  |
| 10604954 | 0.023136836 | Mir224 | microRNA 224 |  |
| 10563094 | 0.022855622 | Fcgrt | Fc receptor, IgG, alpha chain transporter |  |
| 10548422 | 0.022793878 | Klri2 | killer cell lectin-like receptor family I member 2 |  |
| 10548345 | 0.022698162 | Klrk1 | killer cell lectin-like receptor subfamily K, member 1 |  |
| 10472860 | 0.022582134 | Rapgef4 | Rap guanine nucleotide exchange factor (GEF) 4 |  |
| 10448916 | 0.022224558 | Tpsab1 | tryptase alpha/beta 1 |  |
| 10382316 | 0.022085625 | Kcnj16 | potassium inwardly-rectifying channel, subfamily J, member 16 |  |
| 10563760 | 0.021909901 | Mrgprb2 | MAS-related GPR, member B2 |  |
| 10389134 | 0.021893395 | Slfn9 | schlafen 9 |  |
| 10542140 | 0.02189117 | Klrb1f | killer cell lectin-like receptor subfamily B member 1F |  |
| 10539931 | 0.021835361 | Gm1965 | predicted gene 1965 |  |
| 10442786 | 0.021789435 | Tpsb2 | tryptase beta 2 |  |
| 10403034 | 0.021672198 | Ighv8-8 | immunoglobulin heavy variable 8-8 |  |
| 10506360 | 0.021494173 | Sgip1 | SH3-domain GRB2-like (endophilin) interacting protein 1 |  |
| 10596428 | 0.021282361 | Col6a4 | collagen, type VI, alpha 4 |  |
| 10457091 | 0.021274488 | Neto1 | neuropilin (NRP) and tolloid (TLL)-like 1 |  |
| 10352104 | 0.021266646 | Gm16432 | predicted gene 16432 |  |
| 10549647 | 0.021122905 | Ncr1 | natural cytotoxicity triggering receptor 1 |  |
| 10393642 | 0.020978938 | Eif4a3 | eukaryotic translation initiation factor 4A3 |  |
| 10368240 | 0.020905057 | Tcf21 | transcription factor 21 |  |
| 10414470 | 0.02062464 | Tlr11 | toll-like receptor 11 |  |
| 10457038 | 0.020618088 |  | Mus musculus chromosome 18, clone RP23-321L15 |  |
| 10374998 | 0.020596557 | Gpr75 | G protein-coupled receptor 75 |  |
| 10521600 | 0.02057941 |  | Mus musculus MLV-related proviral Env polyprotein (LOC108169043) |  |
| 10362111 | 0.02057941 | Gm40611 | predicted gene, 40611 |  |
| 10402981 | 0.020567071 | Ighe | Immunoglobulin heavy constant epsilon |  |
| 10510047 | 0.020515376 | Tmem51os1 | Tmem51 opposite strand 1 |  |
| 10403063 | 0.02037089 | Ighv8-12 | immunoglobulin heavy variable V8-12 |  |
| 10469573 | 0.020362748 | Gm25859 | predicted gene, 25859 |  |
| 10533720 | 0.020125081 | Hcar2 | hydroxycarboxylic acid receptor 2 |  |
| 10545224 | 0.019989445 | Igkv12-40 | immunoglobulin kappa chain variable 12-40 |  |
| 10498978 | 0.019984795 | Lrat | lecithin-retinol acyltransferase (phosphatidylcholine-retinol-O-acyltransferase) |  |
| 10549635 | 0.019842892 | Lilra5 | leukocyte immunoglobulin-like receptor, subfamily A (with TM domain), member 5 |  |
| 10584458 | 0.019712546 | Olfr920 | olfactory receptor 920 |  |
| 10548513 | 0.01960164 | Klra9 | killer cell lectin-like receptor subfamily A, member 9 |  |
| 10403816 | 0.019596335 | Tcrg-V4 | T cell receptor gamma, variable 4 |  |
| 10479176 | 0.019407342 | Gm6710 | predicted gene 6710 |  |
| 10572949 | 0.019371733 | Nr3c2 | nuclear receptor subfamily 3, group C, member 2 |  |
| 10376332 | -0.021295179 | 4930438A08Rik | RIKEN cDNA 4930438A08 gene |  |
| 10517573 | -0.021314851 | Cela3b | chymotrypsin-like elastase family, member 3B |  |
| 10566350 | -0.02151204 | Trim30b | tripartite motif-containing 30B |  |
| 10349166 | -0.021711554 | Serpinb10 | serine (or cysteine) peptidase inhibitor, clade B (ovalbumin), member 10 |  |
| 10563597 | -0.021945003 | Saa3 | serum amyloid A 3 |  |
| 10429968 | -0.021975626 |  | Mus musculus chromosome 15, clone RP23-415D19 |  |
| 10581355 | -0.022050815 | Ctrl | chymotrypsin-like |  |
| 10500982 | -0.022074661 | I830077J02Rik | RIKEN cDNA I830077J02 gene |  |
| 10505438 | -0.022573054 | Orm1 | orosomucoid 1 |  |
| 10351206 | -0.022579067 | Selp | selectin, platelet |  |
| 10464298 | -0.022712895 | Pnlip | pancreatic lipase |  |
| 10552888 | -0.022821344 | Slc6a16 | solute carrier family 6, member 16 |  |
| 10449467 | -0.022918193 | Clps | colipase, pancreatic |  |
| 10523134 | -0.02324767 | Pf4 | platelet factor 4 |  |
| 10510167 | -0.023448923 | Cdv3 | carnitine deficiency-associated gene expressed in ventricle 3 |  |
| 10574276 | -0.023475926 | Adgrg3 | adhesion G protein-coupled receptor G3 |  |
| 10550509 | -0.023479269 | Pglyrp1 | peptidoglycan recognition protein 1 |  |
| 10544273 | -0.024001727 | Clec5a | C-type lectin domain family 5, member a |  |
| 10537509 | -0.024071253 | Mgam | maltase-glucoamylase |  |
| 10481262 | -0.024127001 | Fcnb | ficolin B |  |
| 10538706 | -0.024274727 | Mmrn1 | multimerin 1 |  |
| 10478633 | -0.024291733 | Mmp9 | matrix metallopeptidase 9 |  |
| 10466606 | -0.024301982 | Anxa1 | annexin A1 |  |
| 10566580 | -0.024470869 | Gm4759 | predicted gene 4759 |  |
| 10439292 | -0.024852312 | BC100530 | cDNA sequence BC100530 |  |
| 10537638 | -0.024868853 | Try10 | trypsin 10 |  |
| 10481278 | -0.025044187 | Cel | carboxyl ester lipase |  |
| 10406736 | -0.025069035 | F2rl2 | coagulation factor II (thrombin) receptor-like 2 |  |
| 10396831 | -0.025238889 | Arg2 | arginase type II |  |
| 10356886 | -0.025255517 | Slco4c1 | solute carrier organic anion transporter family, member 4C1 |  |
| 10581605 | -0.025732377 | Hp | haptoglobin |  |
| 10537014 | -0.025794889 | Cpa2 | carboxypeptidase A2, pancreatic |  |
| 10523977 | -0.025876219 | Gm10419 | predicted gene 10419 |  |
| 10436100 | -0.02588697 | Retnlg | resistin like gamma |  |
| 10510201 | -0.0265949 | Rex2 | reduced expression 2 |  |
| 10539200 | -0.026847716 | Reg1 | regenerating islet-derived 1 |  |
| 10537051 | -0.026904787 | Cpa1 | carboxypeptidase A1, pancreatic |  |
| 10571865 | -0.027275541 | Scrg1 | scrapie responsive gene 1 |  |
| 10408693 | -0.027652758 | F13a1 | coagulation factor XIII, A1 subunit |  |
| 10544326 | -0.027880978 | 2210010C04Rik | RIKEN cDNA 2210010C04 gene |  |
| 10374248 | -0.028011123 | Abca13 | ATP-binding cassette, sub-family A (ABC1), member 13 |  |
| 10473399 | -0.028299698 | Prg2 | proteoglycan 2, bone marrow |  |
| 10569020 | -0.028430841 | Ifitm6 | interferon induced transmembrane protein 6 |  |
| 10492964 | -0.028526876 | Cd5l | CD5 antigen-like |  |
| 10358879 | -0.028987432 | Npl | N-acetylneuraminate pyruvate lyase |  |
| 10566585 | -0.029018407 | Gm1966 | predicted gene 1966 |  |
| 10587655 | -0.029689665 | Zfp949 | zinc finger protein 949 |  |
| 10389717 | -0.029705526 |  | Mus musculus endogenous virus NZB virus XMV-3 envelope protein gene |  |
| 10563712 | -0.030109404 | Mrgpra2b | MAS-related GPR, member A2B |  |
| 10501500 | -0.030340971 | Amy2a5 | amylase 2a5 |  |
| 10497463 | -0.030526217 | Cpb1 | carboxypeptidase B1 (tissue) |  |
| 10499861 | -0.03054334 | S100a9 | S100 calcium binding protein A9 (calgranulin B) |  |
| 10410931 | -0.030755878 | Vcan | versican |  |
| 10478048 | -0.031148945 | Lbp | lipopolysaccharide binding protein |  |
| 10557058 | -0.031149388 | Polr3e | polymerase (RNA) III (DNA directed) polypeptide E |  |
| 10501544 | -0.031223369 | Amy2a4 | amylase 2a4 |  |
| 10587350 | -0.031563419 | Ddx43 | DEAD (Asp-Glu-Ala-Asp) box polypeptide 43 |  |
| 10433172 | -0.0315701 | Glycam1 | glycosylation dependent cell adhesion molecule 1 |  |
| 10566366 | -0.031730852 | Trim30d | tripartite motif-containing 30D |  |
| 10589535 | -0.031861553 | Ngp | neutrophilic granule protein |  |
| 10481627 | -0.032122737 | Lcn2 | lipocalin 2 |  |
| 10466224 | -0.0321285 | Ms4a3 | membrane-spanning 4-domains, subfamily A, member 3 |  |
| 10524698 | -0.032221492 | Pla2g1b | phospholipase A2, group IB, pancreas |  |
| 10403021 | -0.032623393 | Ighv1-43 | immunoglobulin heavy variable V1-43 |  |
| 10560886 | -0.032718126 | Cd177 | CD177 antigen |  |
| 10501494 | -0.032830067 | Amy2a5 | amylase 2a5 |  |
| 10510191 | -0.032832279 | Zfp600 | zinc finger protein 600 |  |
| 10392808 | -0.033290021 | Cd300ld | CD300 molecule like family member d |  |
| 10597098 | -0.03350291 | Camp | cathelicidin antimicrobial peptide |  |
| 10403069 | -0.033857636 | Ighv1-74 | immunoglobulin heavy variable V1-74 |  |
| 10364529 | -0.034799577 | Prtn3 | proteinase 3 |  |
| 10384974 | -0.035227372 | Il9r | interleukin 9 receptor |  |
| 10468877 | -0.03581213 |  | Mus musculus endogenous virus NZB virus XMV-3 envelope protein gene |  |
| 10367744 | -0.036092429 |  | Mus musculus mobilized endogenous polytropic provirus clone 5 truncated gag-pol polyprotein (gag) gene |  |
| 10547153 | -0.036109828 | Alox5 | arachidonate 5-lipoxygenase |  |
| 10493831 | -0.03625608 | S100a8 | S100 calcium binding protein A8 (calgranulin A) |  |
| 10429520 | -0.036957094 | Ly6d | lymphocyte antigen 6 complex, locus D |  |
| 10518059 | -0.037605533 | Ctrc | chymotrypsin C (caldecrin) |  |
| 10364535 | -0.03783231 | Elane | elastase, neutrophil expressed |  |
| 10357381 | -0.039132631 | Map3k19 | mitogen-activated protein kinase kinase kinase 19 |  |
| 10402399 | -0.0394466 | Serpina1a | serine (or cysteine) peptidase inhibitor, clade A, member 1A |  |
| 10380174 | -0.039559431 | Mpo | myeloperoxidase |  |
| 10589703 | -0.039898301 | Ltf | lactotransferrin |  |
| 10430186 | -0.040093782 | Apol7c | apolipoprotein L 7c |  |
| 10402409 | -0.040868825 | Serpina1e | serine (or cysteine) peptidase inhibitor, clade A, member 1E |  |
| 10420261 | -0.041207874 | Ctsg | cathepsin G |  |
| 10351873 | -0.042624015 | Ifi209 | interferon activated gene 209 |  |
| 10349968 | -0.046750591 | Chil1 | chitinase-like 1 |  |
| 10403011 | -0.047909511 | Ighv13-2 | immunoglobulin heavy variable 13-2 |  |
| 10518358 | -0.051816774 | Zfp979 | zinc finger protein 979 |  |
| 10415081 | -0.054987949 | Gm41154 | predicted gene, 41154 |  |
| 10586076 | -0.054987949 | Gm40525 | predicted gene, 40525 |  |
| 10520121 | -0.054987949 |  | Mus musculus endogenous virus NZB virus XMV-3 envelope protein gene |  |
| 10578405 | -0.054987949 | Gm40498 | predicted gene, 40498 |  |
| 10402390 | -0.0561168 | Serpina1b | serine (or cysteine) preptidase inhibitor, clade A, member 1B |  |
| 10501020 | -0.059589751 | Chil3 | chitinase-like 3 |  |
| 10360370 | -0.06177087 | Ifi214 | interferon activated gene 214 |  |
| 10375121 | -0.07278036 | Gm10462 | predicted gene 10462 |  |
| 10429580 | -0.080973339 | I830127L07Rik | RIKEN cDNA I830127L07 gene |  |
| 10543333 | -0.086992855 | Aass | aminoadipate-semialdehyde synthase |  |

**Supplemental Table 20.** The list of spleen surrogate marker candidates by pattern matching analysis between brain PC1 values and spleen microarray data

|  | **Spleen microarray signal values** | | | | | |  | **Spleen microarray signal values** | | | | | |  |  |  |
| --- | --- | --- | --- | --- | --- | --- | --- | --- | --- | --- | --- | --- | --- | --- | --- | --- |
|  | **Control A.SW** | | | **A.SW with EAE** | | | **A.SW** | **Control SJL/J** | | | **SJL/J with EAE** | | | **SJL/J** |  |  |
| **Probe Set ID** | **1** | **2** | **3** | **1** | **2** | **3** | **log ratio** | **1** | **2** | **3** | **1** | **2** | **3** | **log ratio** | ***r*** | **Symbol** |
| 10602896 | 7.5 | 7.7 | 7.7 | 8.5 | 9.7 | 8.4 | 1.2 | 8.3 | 8.3 | 8.4 | 10.1 | 10.0 | 10.7 | 1.9 | 0.94 | Adgrg2 |
| 10451953 | 10.3 | 9.7 | 9.7 | 10.8 | 11.7 | 10.6 | 1.1 | 10.5 | 10.0 | 10.2 | 11.6 | 12.0 | 12.3 | 1.7 | 0.93 | Lrg1 |
| 10373740 | 8.7 | 8.9 | 8.7 | 10.1 | 10.9 | 10.0 | 1.5 | 9.4 | 9.3 | 9.3 | 11.2 | 10.7 | 11.6 | 1.8 | 0.93 | Pik3ip1 |
| 10377439 | 9.4 | 9.4 | 9.6 | 10.6 | 11.0 | 10.4 | 1.2 | 9.6 | 9.5 | 9.8 | 11.1 | 10.9 | 11.6 | 1.6 | 0.90 | Per1 |
| 10512279 | 8.8 | 8.8 | 9.0 | 10.2 | 11.3 | 9.8 | 1.6 | 9.0 | 8.9 | 9.0 | 11.2 | 10.9 | 11.1 | 2.1 | 0.90 | Cntfr |
| 10401931 | 7.9 | 8.2 | 7.8 | 9.6 | 9.5 | 8.9 | 1.4 | 8.0 | 8.0 | 8.1 | 10.3 | 10.2 | 9.4 | 1.9 | 0.89 | Unidentified |
| 10401937 | 7.9 | 8.2 | 7.8 | 9.6 | 9.5 | 8.9 | 1.4 | 8.0 | 8.0 | 8.1 | 10.3 | 10.2 | 9.4 | 1.9 | 0.89 | BC005685 |
| 10435497 | 7.4 | 6.9 | 6.9 | 8.2 | 10.0 | 7.1 | 1.4 | 6.5 | 6.3 | 5.7 | 10.9 | 10.2 | 12.1 | 4.9 | 0.89 | Stfa2l1 |
| 10417561 | 7.8 | 7.8 | 8.1 | 8.7 | 9.3 | 8.8 | 1.0 | 8.1 | 7.8 | 8.0 | 9.3 | 9.1 | 9.1 | 1.2 | 0.88 | Fam107a |
| 10452815 | 9.9 | 9.9 | 9.7 | 10.6 | 11.4 | 10.7 | 1.1 | 9.7 | 9.4 | 9.4 | 11.3 | 11.3 | 11.6 | 1.9 | 0.88 | Xdh |
| 10449452 | 9.1 | 9.2 | 9.1 | 10.6 | 11.6 | 10.3 | 1.7 | 9.2 | 9.2 | 9.3 | 11.3 | 11.2 | 11.3 | 2.0 | 0.88 | Fkbp5 |
| 10430851 | 9.0 | 9.3 | 9.4 | 10.3 | 11.3 | 10.2 | 1.3 | 9.4 | 9.6 | 9.6 | 11.0 | 10.8 | 11.3 | 1.5 | 0.88 | Cyp2d22 |
| 10427436 | 7.2 | 7.2 | 7.1 | 8.9 | 8.9 | 8.5 | 1.6 | 7.6 | 7.1 | 7.1 | 8.9 | 9.4 | 9.0 | 1.8 | 0.87 | C7 |
| 10398075 | 9.4 | 10.0 | 9.3 | 11.5 | 12.8 | 11.0 | 2.2 | 9.2 | 8.8 | 9.7 | 12.3 | 12.4 | 12.7 | 3.2 | 0.87 | Serpina3n |
| 10593225 | 8.8 | 8.8 | 8.6 | 10.0 | 10.9 | 10.2 | 1.6 | 8.6 | 8.4 | 8.9 | 10.5 | 10.6 | 10.8 | 2.0 | 0.86 | Zbtb16 |
| 10606989 | 9.6 | 9.9 | 9.7 | 11.2 | 11.3 | 10.9 | 1.4 | 9.5 | 9.5 | 9.7 | 11.6 | 11.4 | 11.3 | 1.8 | 0.86 | Tsc22d3 |
| 10458894 | 7.2 | 7.3 | 7.4 | 8.5 | 10.1 | 7.6 | 1.4 | 7.2 | 7.4 | 7.2 | 9.8 | 9.2 | 11.1 | 2.8 | 0.86 | Lox |
| 10518059 | 8.5 | 7.8 | 8.5 | 10.7 | 9.8 | 8.9 | 1.5 | 10.0 | 9.2 | 11.2 | 12.9 | 11.7 | 12.5 | 2.2 | 0.86 | Ctrc |
| 10538892 | 8.3 | 8.4 | 8.1 | 9.8 | 9.7 | 9.0 | 1.2 | 7.8 | 8.2 | 8.3 | 10.4 | 10.0 | 9.5 | 1.8 | 0.85 | LOC641050 |
| 10359582 | 7.4 | 7.2 | 7.5 | 9.0 | 9.9 | 7.9 | 1.6 | 7.4 | 8.0 | 7.6 | 9.9 | 9.7 | 9.1 | 1.9 | 0.85 | Fmo2 |
| 10504755 | 8.8 | 9.0 | 8.9 | 10.4 | 10.1 | 9.8 | 1.2 | 8.9 | 8.8 | 9.3 | 10.7 | 10.4 | 10.1 | 1.4 | 0.85 | BC057193 |
| 10543017 | 8.1 | 8.5 | 8.4 | 11.4 | 12.1 | 10.8 | 3.1 | 8.6 | 8.8 | 9.0 | 11.7 | 11.5 | 11.7 | 2.8 | 0.84 | Pdk4 |
| 10494978 | 10.7 | 10.9 | 10.5 | 11.8 | 12.2 | 11.5 | 1.1 | 10.5 | 10.5 | 10.6 | 12.2 | 11.9 | 12.0 | 1.5 | 0.83 | Ptpn22 |
| 10510574 | 10.0 | 10.4 | 10.3 | 12.1 | 12.4 | 12.2 | 1.9 | 10.2 | 10.1 | 10.3 | 12.3 | 12.2 | 12.4 | 2.1 | 0.82 | Errfi1 |
| 10369290 | 9.2 | 9.3 | 9.7 | 11.0 | 11.2 | 11.3 | 1.7 | 9.4 | 9.0 | 9.4 | 11.4 | 11.0 | 11.5 | 2.1 | 0.81 | Ddit4 |
| 10482528 | 7.6 | 7.8 | 7.5 | 9.1 | 10.0 | 9.0 | 1.8 | 7.7 | 7.4 | 7.7 | 9.6 | 9.3 | 9.3 | 1.8 | 0.81 | Neb |
| 10522503 | 9.8 | 9.8 | 9.8 | 11.1 | 11.9 | 11.0 | 1.6 | 9.6 | 9.7 | 9.7 | 11.5 | 11.3 | 11.4 | 1.7 | 0.80 | Pdgfra |
| 10568001 | 7.8 | 8.2 | 8.4 | 9.6 | 9.5 | 9.6 | 1.4 | 8.2 | 8.0 | 8.3 | 10.1 | 9.7 | 9.2 | 1.5 | 0.80 | Sult1a1 |
| 10483353 | 8.9 | 9.1 | 8.7 | 10.2 | 10.5 | 9.8 | 1.2 | 8.7 | 9.0 | 8.8 | 10.3 | 10.1 | 10.1 | 1.4 | 0.80 | Scn7a |
| 10362896 | 14.2 | 14.1 | 14.0 | 12.7 | 11.9 | 13.1 | -1.5 | 13.7 | 13.9 | 13.8 | 12.3 | 12.7 | 12.6 | -1.3 | -0.80 | Cd24a |
| 10344460 | 10.3 | 10.3 | 9.6 | 7.1 | 6.1 | 7.4 | -3.2 | 9.6 | 9.7 | 9.1 | 6.8 | 7.2 | 6.9 | -2.5 | -0.80 | Unidentified |
| 10339181 | 9.2 | 8.7 | 8.8 | 6.3 | 6.1 | 5.7 | -2.9 | 8.4 | 8.8 | 8.2 | 6.1 | 6.4 | 5.8 | -2.4 | -0.80 | Unidentified |
| 10582626 | 11.0 | 10.6 | 11.0 | 8.3 | 7.9 | 8.9 | -2.5 | 11.0 | 11.1 | 10.7 | 8.4 | 8.5 | 8.3 | -2.5 | -0.80 | Abcb10 |
| 10408616 | 11.1 | 10.8 | 11.1 | 9.2 | 9.0 | 9.3 | -1.8 | 10.7 | 10.7 | 10.3 | 9.3 | 9.3 | 9.1 | -1.3 | -0.80 | Slc22a23 |
| 10451670 | 10.8 | 10.7 | 10.9 | 7.5 | 7.1 | 8.7 | -3.0 | 11.1 | 11.3 | 10.8 | 7.6 | 7.9 | 7.7 | -3.3 | -0.80 | Tspo2 |
| 10602372 | 13.1 | 13.0 | 13.1 | 10.3 | 9.1 | 12.7 | -2.4 | 13.0 | 12.9 | 12.6 | 8.8 | 9.8 | 11.2 | -2.9 | -0.80 | Alas2 |
| 10340162 | 9.1 | 8.7 | 9.1 | 6.1 | 6.0 | 6.5 | -2.8 | 8.2 | 8.5 | 7.4 | 5.9 | 6.1 | 6.6 | -1.8 | -0.80 | Unidentified |
| 10586907 | 9.4 | 8.9 | 9.3 | 7.2 | 7.2 | 7.2 | -2.0 | 8.6 | 8.8 | 8.6 | 7.3 | 7.4 | 7.1 | -1.4 | -0.80 | Mns1 |
| 10343265 | 9.8 | 9.6 | 9.5 | 7.5 | 7.6 | 8.2 | -1.9 | 9.2 | 9.1 | 8.7 | 7.8 | 7.7 | 7.7 | -1.3 | -0.80 | Unidentified |
| 10462343 | 9.6 | 9.4 | 9.6 | 8.0 | 7.9 | 8.1 | -1.6 | 9.4 | 9.5 | 9.3 | 8.1 | 8.1 | 7.9 | -1.4 | -0.80 | Gm9895 |
| 10376096 | 8.5 | 8.4 | 8.5 | 7.2 | 7.3 | 7.6 | -1.1 | 8.3 | 8.5 | 8.4 | 7.3 | 7.5 | 7.2 | -1.1 | -0.81 | Acsl6 |
| 10585068 | 8.8 | 8.6 | 8.7 | 7.0 | 6.8 | 7.4 | -1.6 | 8.5 | 8.7 | 8.4 | 7.0 | 7.1 | 7.1 | -1.4 | -0.81 | Nxpe4 |
| 10515156 | 11.4 | 11.1 | 11.1 | 9.6 | 9.3 | 9.5 | -1.7 | 10.8 | 11.2 | 10.8 | 9.4 | 9.8 | 9.2 | -1.5 | -0.81 | Minpp1 |
| 10375055 | 10.7 | 10.7 | 11.0 | 8.2 | 8.1 | 9.2 | -2.3 | 10.3 | 10.5 | 10.1 | 8.6 | 8.4 | 8.6 | -1.8 | -0.81 | Hbq1b |
| 10537179 | 11.5 | 11.2 | 11.4 | 8.7 | 8.1 | 10.9 | -2.1 | 10.7 | 10.8 | 10.6 | 7.9 | 8.3 | 9.6 | -2.2 | -0.81 | Bpgm |
| 10476252 | 11.6 | 11.4 | 11.7 | 10.0 | 10.3 | 10.4 | -1.4 | 11.5 | 11.5 | 11.4 | 10.1 | 10.1 | 10.4 | -1.3 | -0.81 | Cdc25b |
| 10341300 | 11.9 | 11.5 | 11.8 | 9.3 | 9.2 | 9.9 | -2.3 | 11.7 | 11.7 | 11.2 | 9.4 | 9.5 | 9.5 | -2.0 | -0.81 | Unidentified |
| 10495243 | 10.3 | 10.2 | 10.6 | 8.0 | 8.1 | 8.4 | -2.2 | 9.9 | 10.4 | 9.9 | 8.1 | 8.4 | 8.2 | -1.8 | -0.81 | Gstm5 |
| 10398085 | 12.2 | 12.0 | 12.3 | 9.9 | 9.8 | 10.7 | -2.0 | 12.0 | 12.0 | 11.9 | 9.9 | 10.1 | 10.3 | -1.9 | -0.81 | Glrx5 |
| 10534041 | 11.4 | 11.2 | 11.4 | 9.5 | 9.3 | 9.8 | -1.8 | 10.9 | 11.1 | 10.6 | 9.5 | 9.8 | 9.4 | -1.3 | -0.81 | Stx2 |
| 10495035 | 11.8 | 11.7 | 12.0 | 9.8 | 9.8 | 10.3 | -1.9 | 11.7 | 11.8 | 11.5 | 9.8 | 10.0 | 10.1 | -1.7 | -0.81 | Slc16a1 |
| 10459552 | 10.9 | 10.6 | 10.8 | 8.7 | 9.2 | 9.1 | -1.8 | 10.4 | 10.7 | 10.2 | 8.8 | 9.0 | 9.1 | -1.5 | -0.81 | Spire1 |
| 10474223 | 9.4 | 9.3 | 9.3 | 7.5 | 6.9 | 7.2 | -2.1 | 8.1 | 8.7 | 8.1 | 7.0 | 7.2 | 7.1 | -1.2 | -0.81 | Cd59b |
| 10600500 | 12.5 | 12.3 | 12.5 | 10.6 | 10.4 | 12.2 | -1.3 | 12.4 | 12.3 | 12.2 | 10.3 | 10.6 | 11.1 | -1.6 | -0.81 | Fam220a |
| 10338896 | 9.1 | 8.8 | 9.4 | 6.4 | 6.4 | 6.8 | -2.6 | 8.9 | 8.6 | 8.2 | 6.8 | 6.6 | 5.9 | -2.1 | -0.81 | Unidentified |
| 10376326 | 10.8 | 10.4 | 10.5 | 9.5 | 9.1 | 10.0 | -1.0 | 10.6 | 10.2 | 10.2 | 9.4 | 9.5 | 9.0 | -1.0 | -0.81 | Igtp |
| 10407955 | 10.1 | 10.0 | 10.1 | 8.0 | 7.9 | 8.6 | -1.9 | 9.9 | 10.2 | 9.6 | 8.2 | 8.3 | 7.8 | -1.8 | -0.81 | Epdr1 |
| 10352548 | 9.2 | 8.9 | 9.3 | 7.9 | 7.9 | 7.8 | -1.2 | 8.9 | 9.0 | 8.8 | 7.9 | 7.7 | 8.1 | -1.0 | -0.81 | Slc30a10 |
| 10480891 | 11.8 | 11.6 | 11.9 | 9.3 | 9.2 | 10.1 | -2.3 | 11.3 | 11.7 | 11.4 | 9.4 | 9.6 | 9.5 | -2.0 | -0.81 | Ubac1 |
| 10341292 | 9.2 | 8.9 | 8.5 | 6.4 | 6.4 | 6.4 | -2.5 | 7.3 | 8.1 | 7.7 | 5.8 | 6.5 | 6.3 | -1.5 | -0.82 | Unidentified |
| 10339632 | 9.5 | 9.1 | 9.0 | 7.0 | 7.1 | 7.0 | -2.1 | 8.7 | 8.6 | 7.9 | 6.8 | 6.8 | 7.1 | -1.5 | -0.82 | Unidentified |
| 10342017 | 11.9 | 11.7 | 11.7 | 9.1 | 8.2 | 9.2 | -2.9 | 11.4 | 11.5 | 10.7 | 8.7 | 9.0 | 9.0 | -2.3 | -0.82 | Unidentified |
| 10382973 | 9.4 | 9.6 | 9.9 | 8.4 | 8.1 | 8.7 | -1.2 | 9.6 | 9.8 | 9.5 | 8.3 | 8.4 | 8.4 | -1.3 | -0.82 | 6030468B19Rik |
| 10399710 | 12.8 | 12.3 | 12.4 | 9.1 | 8.0 | 11.3 | -3.0 | 11.9 | 11.9 | 11.6 | 8.0 | 8.9 | 9.8 | -2.9 | -0.82 | Rsad2 |
| 10357249 | 10.9 | 10.7 | 11.0 | 8.9 | 8.7 | 9.1 | -2.0 | 10.7 | 11.0 | 10.7 | 8.7 | 9.2 | 8.7 | -1.9 | -0.82 | Steap3 |
| 10570278 | 9.0 | 8.5 | 8.4 | 7.0 | 6.7 | 7.4 | -1.6 | 8.3 | 8.4 | 8.3 | 6.6 | 7.0 | 7.1 | -1.4 | -0.82 | Unidentified |
| 10339519 | 9.8 | 9.3 | 9.1 | 5.9 | 5.2 | 6.1 | -3.7 | 8.6 | 9.0 | 8.0 | 5.5 | 6.1 | 5.4 | -2.9 | -0.82 | Unidentified |
| 10515385 | 11.9 | 11.8 | 12.1 | 9.7 | 9.7 | 10.3 | -2.1 | 11.9 | 11.9 | 11.6 | 9.9 | 9.9 | 9.7 | -1.9 | -0.82 | Urod |
| 10594582 | 9.5 | 9.5 | 9.7 | 8.4 | 7.8 | 8.6 | -1.3 | 9.7 | 9.8 | 9.4 | 8.2 | 8.3 | 8.2 | -1.4 | -0.82 | Snx22 |
| 10459866 | 12.8 | 12.7 | 12.9 | 11.4 | 11.3 | 11.8 | -1.3 | 12.5 | 12.6 | 12.3 | 11.4 | 11.6 | 11.2 | -1.1 | -0.82 | Slc14a1 |
| 10465395 | 9.8 | 9.7 | 9.8 | 8.8 | 8.6 | 8.8 | -1.0 | 10.1 | 10.2 | 9.9 | 8.6 | 8.7 | 8.2 | -1.5 | -0.82 | Ppp2r5b |
| 10400483 | 11.4 | 10.8 | 11.1 | 7.8 | 6.9 | 8.4 | -3.4 | 10.6 | 11.0 | 10.4 | 7.5 | 8.2 | 6.8 | -3.2 | -0.82 | Slc25a21 |
| 10435075 | 12.8 | 12.3 | 12.5 | 9.7 | 9.4 | 10.1 | -2.8 | 12.2 | 12.4 | 12.0 | 9.6 | 10.0 | 9.4 | -2.5 | -0.82 | Tfrc |
| 10356457 | 8.9 | 7.8 | 8.3 | 6.3 | 6.5 | 6.5 | -1.9 | 7.7 | 8.1 | 7.6 | 5.8 | 6.3 | 6.6 | -1.6 | -0.82 | Dnajb3 |
| 10602090 | 11.6 | 11.3 | 11.5 | 9.9 | 10.8 | 10.2 | -1.2 | 11.0 | 11.2 | 10.7 | 9.8 | 9.9 | 10.1 | -1.0 | -0.82 | Atg4a |
| 10343957 | 12.0 | 11.5 | 11.7 | 9.2 | 8.9 | 9.3 | -2.6 | 11.5 | 11.6 | 11.2 | 9.0 | 9.1 | 9.2 | -2.4 | -0.82 | Unidentified |
| 10343937 | 11.4 | 11.0 | 11.2 | 9.0 | 8.5 | 8.9 | -2.4 | 10.6 | 10.8 | 10.3 | 8.6 | 8.9 | 8.9 | -1.8 | -0.82 | Unidentified |
| 10577449 | 9.1 | 9.0 | 9.3 | 7.7 | 7.4 | 7.6 | -1.6 | 9.0 | 9.1 | 8.9 | 7.4 | 7.7 | 7.5 | -1.5 | -0.82 | Atp7b |
| 10373515 | 9.7 | 9.7 | 10.0 | 9.0 | 8.9 | 9.0 | -0.8 | 9.9 | 10.1 | 9.8 | 8.9 | 9.0 | 8.5 | -1.1 | -0.83 | Suox |
| 10500610 | 13.3 | 13.0 | 13.1 | 11.5 | 11.0 | 12.7 | -1.4 | 12.7 | 12.7 | 12.5 | 11.1 | 11.1 | 11.6 | -1.3 | -0.83 | Fam46c |
| 10338686 | 10.2 | 10.2 | 10.6 | 8.4 | 8.0 | 8.4 | -2.1 | 10.4 | 10.1 | 9.6 | 8.0 | 8.2 | 8.4 | -1.8 | -0.83 | Unidentified |
| 10431035 | 10.7 | 10.3 | 10.7 | 9.3 | 8.8 | 9.1 | -1.5 | 10.2 | 10.4 | 10.2 | 9.0 | 9.2 | 8.9 | -1.2 | -0.83 | Ttll12 |
| 10490955 | 11.2 | 11.0 | 11.5 | 9.8 | 9.4 | 9.7 | -1.6 | 10.9 | 11.2 | 10.7 | 9.6 | 9.6 | 9.6 | -1.3 | -0.83 | Mtfr1 |
| 10340874 | 10.0 | 9.8 | 8.8 | 7.0 | 5.8 | 6.0 | -3.3 | 8.2 | 9.1 | 7.4 | 5.5 | 6.5 | 5.4 | -2.4 | -0.83 | Unidentified |
| 10532248 | 8.0 | 7.5 | 8.0 | 6.8 | 6.6 | 6.6 | -1.1 | 7.6 | 7.8 | 7.7 | 6.6 | 6.4 | 6.8 | -1.1 | -0.83 | Rnf212 |
| 10377319 | 10.4 | 9.9 | 10.3 | 8.3 | 8.3 | 8.4 | -1.9 | 9.8 | 10.2 | 9.9 | 8.0 | 8.5 | 8.3 | -1.7 | -0.83 | Myh10 |
| 10558811 | 11.6 | 11.4 | 11.9 | 9.5 | 9.4 | 10.0 | -2.0 | 11.8 | 11.8 | 11.5 | 9.5 | 9.6 | 9.3 | -2.2 | -0.83 | Ptdss2 |
| 10452151 | 10.1 | 9.8 | 10.2 | 8.7 | 8.7 | 8.9 | -1.3 | 10.1 | 10.1 | 9.8 | 8.7 | 8.7 | 8.7 | -1.3 | -0.83 | Rfx2 |
| 10493382 | 10.8 | 10.7 | 11.1 | 8.4 | 8.0 | 8.7 | -2.5 | 10.6 | 10.9 | 10.6 | 8.2 | 8.7 | 7.8 | -2.5 | -0.83 | Pklr |
| 10587266 | 12.0 | 11.9 | 12.1 | 10.9 | 11.0 | 10.9 | -1.1 | 12.0 | 12.0 | 11.8 | 10.8 | 10.8 | 11.0 | -1.1 | -0.83 | Gclc |
| 10339087 | 11.0 | 10.6 | 11.0 | 8.2 | 7.9 | 8.7 | -2.6 | 10.6 | 10.7 | 10.2 | 8.4 | 7.9 | 8.2 | -2.4 | -0.83 | Unidentified |
| 10340741 | 11.5 | 11.2 | 11.4 | 8.9 | 8.9 | 9.3 | -2.3 | 10.6 | 10.8 | 10.2 | 8.7 | 8.8 | 9.3 | -1.6 | -0.83 | Unidentified |
| 10384956 | 12.0 | 11.5 | 11.7 | 9.0 | 8.6 | 9.3 | -2.8 | 11.3 | 11.5 | 10.9 | 8.9 | 9.2 | 8.5 | -2.4 | -0.83 | Chac2 |
| 10338506 | 10.9 | 10.8 | 11.1 | 9.0 | 8.6 | 9.4 | -1.9 | 10.8 | 10.7 | 10.3 | 9.0 | 8.9 | 8.9 | -1.7 | -0.83 | Unidentified |
| 10592816 | 12.9 | 12.7 | 13.0 | 10.1 | 9.4 | 11.0 | -2.7 | 12.8 | 12.8 | 12.5 | 9.7 | 10.3 | 9.9 | -2.7 | -0.83 | Hmbs |
| 10364916 | 11.3 | 11.2 | 11.6 | 9.8 | 9.6 | 10.1 | -1.5 | 11.4 | 11.3 | 11.2 | 9.8 | 9.9 | 9.4 | -1.6 | -0.83 | Sppl2b |
| 10389300 | 12.6 | 12.3 | 12.8 | 10.0 | 9.4 | 10.8 | -2.5 | 12.7 | 12.6 | 12.3 | 9.5 | 9.9 | 10.1 | -2.7 | -0.83 | Dhrs11 |
| 10360344 | 10.1 | 9.9 | 10.3 | 7.8 | 7.9 | 8.2 | -2.1 | 10.1 | 10.1 | 9.6 | 7.8 | 7.8 | 7.9 | -2.1 | -0.83 | Ackr1 |
| 10401149 | 9.1 | 8.7 | 9.2 | 6.9 | 6.9 | 7.1 | -2.0 | 8.6 | 9.0 | 8.5 | 7.1 | 6.8 | 6.8 | -1.8 | -0.83 | Plek2 |
| 10338234 | 10.1 | 10.0 | 9.4 | 7.9 | 7.8 | 7.8 | -2.0 | 8.9 | 10.0 | 8.8 | 7.3 | 7.9 | 7.4 | -1.7 | -0.83 | Unidentified |
| 10341349 | 9.9 | 9.4 | 9.9 | 7.9 | 8.0 | 8.2 | -1.7 | 9.2 | 9.3 | 9.0 | 7.8 | 7.7 | 8.3 | -1.3 | -0.84 | Unidentified |
| 10442616 | 11.1 | 10.9 | 11.2 | 9.4 | 9.5 | 9.9 | -1.4 | 11.0 | 11.0 | 10.7 | 9.5 | 9.5 | 9.6 | -1.4 | -0.84 | Hagh |
| 10344180 | 13.1 | 13.0 | 13.2 | 10.8 | 10.7 | 11.2 | -2.2 | 12.9 | 12.9 | 12.6 | 10.8 | 10.8 | 10.8 | -2.0 | -0.84 | Unidentified |
| 10343964 | 9.0 | 8.9 | 8.2 | 5.7 | 5.1 | 6.1 | -3.0 | 7.6 | 8.3 | 7.3 | 4.6 | 5.8 | 5.7 | -2.4 | -0.84 | Unidentified |
| 10550131 | 9.6 | 9.4 | 9.7 | 7.4 | 7.0 | 7.5 | -2.3 | 9.3 | 9.4 | 9.1 | 7.1 | 7.5 | 6.9 | -2.1 | -0.84 | Pla2g4c |
| 10590245 | 11.5 | 11.4 | 11.7 | 10.2 | 10.0 | 10.3 | -1.3 | 11.6 | 11.6 | 11.2 | 10.1 | 10.1 | 10.0 | -1.4 | -0.84 | Slc25a38 |
| 10343538 | 12.0 | 11.8 | 12.3 | 9.3 | 9.5 | 10.1 | -2.4 | 12.0 | 11.8 | 11.3 | 9.2 | 9.6 | 9.5 | -2.3 | -0.84 | Unidentified |
| 10355742 | 10.1 | 9.8 | 10.3 | 8.2 | 8.4 | 8.4 | -1.7 | 9.7 | 9.8 | 9.5 | 8.2 | 8.2 | 8.3 | -1.4 | -0.84 | Abcb6 |
| 10340943 | 12.4 | 12.2 | 12.4 | 9.7 | 8.8 | 9.8 | -2.9 | 12.2 | 12.0 | 11.3 | 9.2 | 9.5 | 9.3 | -2.5 | -0.84 | Unidentified |
| 10359861 | 10.3 | 10.1 | 10.9 | 8.0 | 7.9 | 8.7 | -2.2 | 10.2 | 10.3 | 10.2 | 8.0 | 8.1 | 8.0 | -2.2 | -0.84 | Mgst3 |
| 10395142 | 9.0 | 8.6 | 8.8 | 6.8 | 6.8 | 7.0 | -1.9 | 8.2 | 8.6 | 8.0 | 6.8 | 6.9 | 6.5 | -1.5 | -0.84 | Sh3yl1 |
| 10339152 | 9.4 | 9.1 | 9.4 | 6.8 | 7.0 | 7.0 | -2.4 | 8.5 | 8.6 | 7.7 | 6.0 | 5.8 | 7.5 | -1.8 | -0.84 | Unidentified |
| 10397966 | 9.6 | 9.5 | 9.6 | 8.0 | 8.4 | 8.2 | -1.4 | 9.4 | 9.6 | 9.3 | 8.1 | 8.2 | 7.8 | -1.4 | -0.84 | Otub2 |
| 10596166 | 10.5 | 10.4 | 10.8 | 8.0 | 7.8 | 8.6 | -2.4 | 10.9 | 10.9 | 10.5 | 7.9 | 8.0 | 7.7 | -2.9 | -0.84 | 1300017J02Rik |
| 10425755 | 9.9 | 9.4 | 9.9 | 7.6 | 7.3 | 7.9 | -2.1 | 8.3 | 8.6 | 8.3 | 7.2 | 7.4 | 6.9 | -1.2 | -0.84 | Unidentified |
| 10588707 | 11.9 | 11.7 | 12.0 | 10.1 | 9.8 | 10.4 | -1.8 | 11.8 | 11.9 | 11.7 | 10.0 | 10.1 | 9.7 | -1.9 | -0.84 | Ifrd2 |
| 10581340 | 12.4 | 12.2 | 12.5 | 10.3 | 10.1 | 10.9 | -2.0 | 12.2 | 12.2 | 11.9 | 10.2 | 10.2 | 10.4 | -1.8 | -0.84 | Ranbp10 |
| 10472933 | 10.8 | 10.5 | 10.7 | 8.9 | 8.7 | 8.8 | -1.9 | 10.3 | 10.6 | 10.2 | 8.7 | 8.8 | 8.6 | -1.7 | -0.84 | Scrn3 |
| 10339038 | 11.5 | 11.1 | 11.1 | 9.1 | 9.2 | 9.5 | -2.0 | 10.7 | 10.8 | 10.1 | 9.0 | 8.9 | 9.2 | -1.5 | -0.84 | Unidentified |
| 10512757 | 12.1 | 11.9 | 12.1 | 8.4 | 7.1 | 9.6 | -3.7 | 11.9 | 11.9 | 11.6 | 7.9 | 8.1 | 7.9 | -3.8 | -0.84 | Hemgn |
| 10391540 | 8.9 | 8.9 | 9.1 | 7.7 | 7.9 | 7.9 | -1.2 | 9.0 | 9.0 | 8.6 | 7.7 | 7.6 | 7.8 | -1.2 | -0.84 | Mpp2 |
| 10574682 | 11.6 | 11.5 | 11.8 | 10.6 | 10.2 | 10.7 | -1.1 | 11.5 | 11.6 | 11.4 | 10.4 | 10.5 | 10.4 | -1.1 | -0.84 | E2f4 |
| 10535103 | 10.1 | 9.9 | 10.3 | 8.5 | 8.4 | 8.7 | -1.6 | 9.8 | 10.1 | 9.7 | 8.2 | 8.3 | 8.8 | -1.4 | -0.84 | Micall2 |
| 10341036 | 10.1 | 9.9 | 10.1 | 8.5 | 8.5 | 8.9 | -1.4 | 9.9 | 9.9 | 9.4 | 8.4 | 8.6 | 8.4 | -1.3 | -0.84 | Unidentified |
| 10395039 | 10.1 | 9.9 | 10.1 | 8.6 | 8.1 | 8.7 | -1.6 | 9.8 | 9.8 | 9.6 | 8.2 | 8.7 | 8.1 | -1.4 | -0.84 | Cmpk2 |
| 10538640 | 10.5 | 10.1 | 10.3 | 8.5 | 8.7 | 8.6 | -1.7 | 9.7 | 10.0 | 9.6 | 8.5 | 8.4 | 8.4 | -1.4 | -0.84 | Abcg2 |
| 10343004 | 12.2 | 11.9 | 12.0 | 10.1 | 10.3 | 10.1 | -1.9 | 11.6 | 11.6 | 11.1 | 10.0 | 9.8 | 10.2 | -1.5 | -0.85 | Unidentified |
| 10344100 | 10.0 | 9.9 | 10.0 | 6.8 | 6.9 | 7.2 | -3.0 | 9.2 | 9.4 | 8.6 | 6.6 | 6.9 | 6.9 | -2.3 | -0.85 | Unidentified |
| 10342791 | 10.2 | 9.6 | 9.2 | 6.7 | 6.5 | 7.1 | -2.9 | 8.9 | 9.4 | 8.2 | 6.6 | 6.3 | 6.3 | -2.4 | -0.85 | Unidentified |
| 10341865 | 10.8 | 10.6 | 10.6 | 8.5 | 8.4 | 8.3 | -2.2 | 10.0 | 10.3 | 9.7 | 8.0 | 8.2 | 8.5 | -1.8 | -0.85 | Unidentified |
| 10527229 | 10.8 | 10.4 | 10.4 | 8.5 | 8.1 | 10.0 | -1.7 | 10.0 | 10.0 | 9.7 | 7.9 | 8.2 | 8.7 | -1.6 | -0.85 | Fam220a |
| 10458028 | 11.8 | 11.5 | 11.8 | 10.0 | 9.9 | 10.3 | -1.6 | 11.5 | 11.6 | 11.3 | 9.8 | 10.1 | 9.9 | -1.5 | -0.85 | Gypc |
| 10338235 | 8.9 | 9.3 | 9.0 | 6.9 | 6.9 | 7.6 | -2.0 | 8.6 | 9.0 | 8.1 | 7.1 | 6.6 | 7.0 | -1.7 | -0.85 | Unidentified |
| 10567564 | 12.2 | 12.0 | 12.2 | 9.3 | 8.4 | 10.7 | -2.7 | 12.1 | 12.0 | 11.6 | 8.3 | 9.1 | 9.5 | -2.9 | -0.85 | Cdr2 |
| 10341384 | 11.1 | 10.9 | 11.1 | 8.6 | 8.3 | 8.9 | -2.5 | 10.6 | 10.6 | 10.1 | 8.4 | 8.4 | 8.4 | -2.0 | -0.85 | Unidentified |
| 10350335 | 12.4 | 12.2 | 12.7 | 10.2 | 10.0 | 10.7 | -2.1 | 12.5 | 12.4 | 12.1 | 10.1 | 10.2 | 9.9 | -2.3 | -0.85 | Hmbs |
| 10338503 | 7.7 | 7.2 | 6.7 | 5.4 | 4.7 | 5.4 | -2.0 | 6.6 | 6.9 | 5.7 | 5.0 | 4.6 | 4.5 | -1.7 | -0.85 | Unidentified |
| 10570472 | 10.4 | 10.1 | 10.6 | 8.8 | 8.9 | 9.2 | -1.4 | 10.0 | 10.0 | 9.7 | 8.7 | 8.8 | 8.9 | -1.1 | -0.85 | Cln8 |
| 10449163 | 12.4 | 12.2 | 12.5 | 10.7 | 10.0 | 11.0 | -1.8 | 12.3 | 12.4 | 12.1 | 10.4 | 10.5 | 10.1 | -1.9 | -0.85 | Pigq |
| 10376021 | 10.9 | 10.5 | 10.8 | 9.4 | 9.3 | 9.7 | -1.3 | 10.4 | 10.6 | 10.4 | 9.4 | 9.4 | 9.1 | -1.2 | -0.85 | Sept8 |
| 10567108 | 11.1 | 10.8 | 11.3 | 8.3 | 8.2 | 9.0 | -2.6 | 10.8 | 10.8 | 10.4 | 8.1 | 8.4 | 8.3 | -2.4 | -0.85 | Sox6 |
| 10539080 | 11.5 | 11.3 | 11.5 | 9.1 | 9.1 | 10.0 | -2.0 | 11.1 | 11.1 | 10.7 | 9.1 | 9.3 | 9.0 | -1.8 | -0.85 | St3gal5 |
| 10554240 | 13.2 | 13.0 | 13.2 | 11.2 | 10.2 | 12.3 | -1.9 | 13.0 | 13.0 | 12.7 | 10.5 | 11.0 | 10.7 | -2.2 | -0.85 | Isg20 |
| 10339740 | 13.3 | 13.2 | 13.3 | 11.0 | 10.8 | 11.8 | -2.1 | 13.2 | 13.2 | 12.6 | 10.9 | 10.9 | 11.1 | -2.1 | -0.85 | Unidentified |
| 10339747 | 10.3 | 10.1 | 10.3 | 7.9 | 8.1 | 8.6 | -2.1 | 9.6 | 9.7 | 9.3 | 7.7 | 8.0 | 8.0 | -1.6 | -0.85 | Unidentified |
| 10376434 | 10.5 | 10.3 | 10.8 | 7.4 | 7.1 | 8.3 | -2.9 | 10.4 | 10.5 | 10.0 | 7.4 | 7.4 | 7.1 | -3.0 | -0.85 | Btnl10 |
| 10448743 | 9.7 | 9.4 | 9.6 | 7.9 | 8.0 | 8.2 | -1.5 | 8.9 | 9.1 | 8.8 | 7.7 | 8.0 | 7.9 | -1.1 | -0.85 | Fahd1 |
| 10383564 | 10.6 | 10.4 | 10.8 | 7.7 | 7.8 | 8.4 | -2.7 | 10.6 | 10.7 | 10.2 | 7.7 | 7.5 | 7.6 | -2.9 | -0.85 | Fn3k |
| 10338380 | 13.4 | 13.1 | 13.3 | 11.0 | 10.7 | 11.4 | -2.2 | 13.0 | 13.0 | 12.6 | 10.7 | 10.9 | 10.9 | -2.0 | -0.85 | Unidentified |
| 10508454 | 11.9 | 11.8 | 12.1 | 10.5 | 10.5 | 10.9 | -1.3 | 11.8 | 11.8 | 11.4 | 10.4 | 10.6 | 10.5 | -1.2 | -0.85 | Bsdc1 |
| 10566810 | 8.6 | 8.2 | 8.5 | 7.1 | 7.1 | 7.0 | -1.4 | 8.3 | 8.4 | 7.9 | 6.8 | 6.9 | 6.9 | -1.3 | -0.85 | Nrip3 |
| 10541049 | 11.2 | 11.0 | 11.3 | 9.0 | 8.9 | 9.5 | -2.0 | 10.9 | 10.8 | 10.4 | 8.9 | 9.0 | 8.9 | -1.8 | -0.85 | March8 |
| 10343453 | 9.0 | 8.8 | 8.8 | 6.2 | 6.1 | 6.8 | -2.6 | 8.2 | 8.3 | 7.5 | 5.7 | 6.0 | 6.4 | -2.0 | -0.85 | Unidentified |
| 10504692 | 11.3 | 10.9 | 11.4 | 7.6 | 8.0 | 8.8 | -3.0 | 11.0 | 11.1 | 10.4 | 7.6 | 7.9 | 7.7 | -3.1 | -0.85 | Tmod1 |
| 10401359 | 9.2 | 9.2 | 9.2 | 7.9 | 7.9 | 8.2 | -1.2 | 9.1 | 9.1 | 8.8 | 7.8 | 7.8 | 8.0 | -1.1 | -0.85 | Dpf3 |
| 10387743 | 9.5 | 9.4 | 9.7 | 7.9 | 8.1 | 8.4 | -1.4 | 9.4 | 9.4 | 9.2 | 7.8 | 7.8 | 8.1 | -1.4 | -0.86 | Slc2a4 |
| 10342410 | 11.8 | 11.2 | 11.5 | 8.9 | 8.5 | 9.4 | -2.6 | 11.1 | 11.0 | 10.5 | 8.6 | 8.7 | 8.5 | -2.3 | -0.86 | Unidentified |
| 10361906 | 7.9 | 8.5 | 8.0 | 7.3 | 6.3 | 7.7 | -1.1 | 7.6 | 8.3 | 7.6 | 6.6 | 6.6 | 6.6 | -1.2 | -0.86 | Il22ra2 |
| 10339510 | 13.0 | 12.8 | 13.1 | 10.8 | 10.2 | 11.3 | -2.2 | 12.8 | 12.7 | 12.2 | 10.4 | 10.4 | 10.8 | -2.0 | -0.86 | Unidentified |
| 10457780 | 10.3 | 9.9 | 10.3 | 8.3 | 8.1 | 8.2 | -2.0 | 9.8 | 10.0 | 9.7 | 7.9 | 8.1 | 8.0 | -1.9 | -0.86 | Garem |
| 10490913 | 11.7 | 11.8 | 11.4 | 9.9 | 11.0 | 10.7 | -1.1 | 8.8 | 10.2 | 9.6 | 7.4 | 7.1 | 7.3 | -2.3 | -0.86 | Car3 |
| 10421418 | 11.5 | 11.2 | 11.6 | 8.5 | 8.5 | 9.2 | -2.7 | 11.3 | 11.4 | 10.9 | 8.3 | 8.5 | 8.4 | -2.8 | -0.86 | Dmtn |
| 10498620 | 10.7 | 10.3 | 10.4 | 8.9 | 8.6 | 8.7 | -1.8 | 10.1 | 10.3 | 9.9 | 8.4 | 8.5 | 8.8 | -1.6 | -0.86 | Trim59 |
| 10515848 | 12.8 | 12.6 | 12.9 | 9.6 | 8.9 | 10.6 | -3.1 | 12.6 | 12.6 | 12.3 | 9.0 | 9.7 | 9.0 | -3.3 | -0.86 | Ermap |
| 10445046 | 11.4 | 11.1 | 11.4 | 7.5 | 6.9 | 8.8 | -3.6 | 10.9 | 11.1 | 10.5 | 7.0 | 7.4 | 7.3 | -3.6 | -0.86 | Trim10 |
| 10367024 | 10.2 | 10.2 | 10.4 | 7.5 | 7.4 | 7.6 | -2.8 | 8.6 | 9.5 | 8.9 | 7.2 | 7.0 | 7.0 | -1.9 | -0.86 | Tac2 |
| 10343302 | 12.8 | 12.6 | 12.8 | 10.6 | 10.1 | 10.8 | -2.2 | 12.4 | 12.4 | 11.8 | 10.2 | 10.2 | 10.4 | -1.9 | -0.86 | Unidentified |
| 10573483 | 10.5 | 10.5 | 10.6 | 8.5 | 8.2 | 9.3 | -1.9 | 10.3 | 10.4 | 10.0 | 8.3 | 8.6 | 8.2 | -1.9 | -0.86 | Prdx2 |
| 10586491 | 9.5 | 9.5 | 9.8 | 8.5 | 8.1 | 8.5 | -1.2 | 9.3 | 9.4 | 9.1 | 8.4 | 8.3 | 8.2 | -1.0 | -0.86 | Dapk2 |
| 10465379 | 10.8 | 10.6 | 10.7 | 9.4 | 9.1 | 9.4 | -1.4 | 10.6 | 10.6 | 10.2 | 9.2 | 9.3 | 8.9 | -1.3 | -0.86 | Snx15 |
| 10343818 | 13.0 | 12.8 | 13.0 | 10.9 | 10.5 | 11.4 | -2.0 | 12.6 | 12.7 | 12.3 | 10.6 | 10.8 | 10.7 | -1.8 | -0.86 | Unidentified |
| 10341301 | 12.1 | 12.0 | 12.0 | 9.3 | 8.9 | 9.3 | -2.8 | 11.4 | 11.8 | 11.1 | 8.8 | 9.1 | 8.9 | -2.5 | -0.86 | Unidentified |
| 10478962 | 10.5 | 10.3 | 10.5 | 9.2 | 9.1 | 9.3 | -1.2 | 10.3 | 10.4 | 10.0 | 9.1 | 9.1 | 9.1 | -1.1 | -0.86 | Fam210b |
| 10473349 | 11.0 | 10.7 | 11.1 | 7.7 | 7.3 | 8.4 | -3.1 | 10.7 | 10.8 | 10.2 | 7.3 | 7.7 | 7.5 | -3.1 | -0.86 | Ypel4 |
| 10339166 | 10.2 | 10.0 | 10.6 | 7.4 | 7.0 | 8.1 | -2.8 | 9.8 | 9.9 | 9.1 | 7.0 | 7.3 | 7.4 | -2.4 | -0.86 | Unidentified |
| 10339645 | 9.6 | 9.4 | 9.1 | 7.0 | 6.5 | 7.0 | -2.5 | 8.5 | 9.0 | 8.5 | 6.4 | 6.6 | 6.8 | -2.1 | -0.86 | Unidentified |
| 10459391 | 12.6 | 12.2 | 12.3 | 10.0 | 9.7 | 11.1 | -2.1 | 12.1 | 12.2 | 11.7 | 9.5 | 9.8 | 10.3 | -2.1 | -0.86 | Fech |
| 10536908 | 11.8 | 11.6 | 12.0 | 9.1 | 8.8 | 10.1 | -2.5 | 11.8 | 11.8 | 11.4 | 8.8 | 9.0 | 9.1 | -2.7 | -0.86 | Tspan33 |
| 10383556 | 10.9 | 10.7 | 11.1 | 8.7 | 8.4 | 9.0 | -2.2 | 10.9 | 11.0 | 10.6 | 8.3 | 8.7 | 7.7 | -2.6 | -0.86 | Fn3krp |
| 10340029 | 11.6 | 11.5 | 11.4 | 9.4 | 9.1 | 9.0 | -2.3 | 11.0 | 11.3 | 10.7 | 8.9 | 9.0 | 8.8 | -2.1 | -0.86 | Unidentified |
| 10343638 | 7.8 | 7.6 | 7.7 | 6.6 | 6.1 | 5.8 | -1.5 | 7.3 | 7.8 | 7.3 | 5.7 | 6.0 | 6.1 | -1.5 | -0.86 | Unidentified |
| 10539669 | 10.6 | 10.3 | 10.7 | 7.9 | 7.7 | 8.5 | -2.5 | 10.3 | 10.4 | 10.0 | 7.6 | 7.9 | 7.6 | -2.5 | -0.86 | Add2 |
| 10370046 | 7.9 | 7.7 | 8.3 | 6.7 | 6.5 | 7.0 | -1.2 | 8.0 | 8.0 | 7.5 | 6.7 | 6.2 | 6.6 | -1.3 | -0.86 | Gm867 |
| 10364784 | 11.6 | 11.2 | 11.6 | 9.4 | 9.2 | 9.3 | -2.2 | 11.2 | 11.7 | 11.3 | 9.1 | 9.2 | 8.5 | -2.5 | -0.86 | Reep6 |
| 10437590 | 10.8 | 10.7 | 10.9 | 9.2 | 9.4 | 9.4 | -1.5 | 10.1 | 10.3 | 9.9 | 9.0 | 8.9 | 9.2 | -1.1 | -0.86 | Carhsp1 |
| 10592772 | 11.1 | 11.0 | 11.2 | 8.6 | 8.5 | 9.2 | -2.3 | 11.0 | 11.2 | 10.9 | 8.3 | 8.6 | 8.4 | -2.6 | -0.86 | Abcg4 |
| 10344331 | 12.7 | 12.4 | 12.8 | 9.9 | 9.6 | 10.2 | -2.8 | 12.3 | 12.3 | 12.0 | 9.5 | 9.7 | 9.7 | -2.6 | -0.86 | Unidentified |
| 10339174 | 11.1 | 10.9 | 11.0 | 8.8 | 8.7 | 9.2 | -2.1 | 10.9 | 10.8 | 10.2 | 8.4 | 8.7 | 8.8 | -2.0 | -0.86 | Unidentified |
| 10544383 | 12.4 | 12.2 | 12.6 | 8.5 | 6.8 | 9.8 | -4.0 | 12.4 | 12.4 | 12.1 | 7.3 | 8.2 | 7.0 | -4.8 | -0.86 | Kel |
| 10358457 | 7.6 | 7.0 | 7.2 | 5.3 | 5.4 | 5.8 | -1.7 | 6.8 | 7.3 | 6.8 | 5.0 | 5.2 | 5.5 | -1.7 | -0.86 | Bex4 |
| 10423971 | 11.5 | 11.1 | 11.4 | 7.6 | 7.1 | 8.4 | -3.6 | 10.7 | 11.0 | 10.6 | 7.4 | 7.4 | 6.9 | -3.5 | -0.86 | Pkhd1l1 |
| 10341538 | 13.7 | 13.7 | 13.9 | 11.8 | 11.6 | 12.5 | -1.8 | 13.8 | 13.6 | 13.3 | 11.4 | 11.7 | 11.8 | -1.9 | -0.86 | Unidentified |
| 10342031 | 11.0 | 10.7 | 10.9 | 8.8 | 8.4 | 9.4 | -2.0 | 9.3 | 8.9 | 8.1 | 7.4 | 7.2 | 6.9 | -1.6 | -0.86 | Unidentified |
| 10434191 | 10.1 | 9.9 | 10.2 | 8.6 | 8.3 | 8.7 | -1.5 | 9.9 | 10.0 | 9.7 | 8.4 | 8.6 | 7.9 | -1.5 | -0.86 | Txnrd2 |
| 10341667 | 10.6 | 9.9 | 10.1 | 7.6 | 7.7 | 7.9 | -2.5 | 9.8 | 9.9 | 9.3 | 7.1 | 7.5 | 7.4 | -2.4 | -0.86 | Unidentified |
| 10340700 | 10.8 | 10.6 | 10.8 | 9.6 | 9.9 | 9.8 | -1.0 | 10.7 | 10.6 | 10.4 | 9.5 | 9.4 | 9.8 | -1.0 | -0.86 | Unidentified |
| 10385826 | 10.4 | 10.1 | 10.4 | 7.5 | 6.8 | 7.9 | -2.9 | 9.9 | 10.1 | 9.3 | 6.7 | 7.3 | 7.2 | -2.7 | -0.86 | Sowaha |
| 10512747 | 9.1 | 8.9 | 9.5 | 8.0 | 8.0 | 8.4 | -1.1 | 9.1 | 9.1 | 8.9 | 7.9 | 7.9 | 8.0 | -1.1 | -0.87 | Trmo |
| 10342331 | 9.3 | 9.6 | 9.1 | 7.1 | 6.7 | 7.2 | -2.4 | 8.5 | 8.8 | 8.2 | 6.2 | 6.9 | 6.7 | -1.9 | -0.87 | Unidentified |
| 10509002 | 12.0 | 11.8 | 12.1 | 8.0 | 6.6 | 9.4 | -3.9 | 12.0 | 12.0 | 11.7 | 7.0 | 7.7 | 6.8 | -4.8 | -0.87 | Rhd |
| 10598507 | 10.9 | 10.8 | 11.3 | 8.6 | 7.8 | 9.0 | -2.6 | 10.9 | 11.1 | 10.8 | 8.1 | 8.2 | 7.9 | -2.9 | -0.87 | Slc38a5 |
| 10339567 | 10.9 | 10.8 | 10.9 | 8.3 | 8.3 | 8.0 | -2.6 | 10.4 | 10.5 | 9.8 | 7.6 | 7.7 | 8.3 | -2.4 | -0.87 | Unidentified |
| 10608710 | 13.7 | 13.6 | 13.8 | 10.7 | 8.0 | 12.2 | -3.4 | 13.6 | 13.7 | 13.5 | 7.9 | 10.1 | 8.3 | -4.8 | -0.87 | Unidentified |
| 10445192 | 12.8 | 12.4 | 12.7 | 9.2 | 7.8 | 10.1 | -3.6 | 12.3 | 12.4 | 12.1 | 7.9 | 9.0 | 7.8 | -4.0 | -0.87 | Rhag |
| 10341823 | 12.0 | 11.7 | 12.0 | 9.6 | 9.0 | 9.8 | -2.5 | 11.6 | 11.5 | 10.9 | 9.1 | 9.2 | 9.2 | -2.2 | -0.87 | Unidentified |
| 10343564 | 10.0 | 9.9 | 10.3 | 8.7 | 8.2 | 8.3 | -1.7 | 9.6 | 9.4 | 9.1 | 8.0 | 8.3 | 7.8 | -1.3 | -0.87 | Unidentified |
| 10362899 | 11.2 | 11.1 | 11.4 | 8.9 | 8.8 | 9.6 | -2.1 | 11.1 | 10.9 | 10.5 | 8.7 | 8.8 | 8.9 | -2.0 | -0.87 | F930017D23Rik |
| 10344233 | 9.8 | 9.5 | 9.5 | 7.3 | 7.2 | 6.9 | -2.5 | 8.9 | 9.4 | 8.2 | 6.5 | 7.0 | 6.5 | -2.2 | -0.87 | Unidentified |
| 10548761 | 10.3 | 10.5 | 10.8 | 9.4 | 8.9 | 9.6 | -1.2 | 10.0 | 10.5 | 10.0 | 9.1 | 9.2 | 8.6 | -1.2 | -0.87 | Hebp1 |
| 10368720 | 11.7 | 11.3 | 11.6 | 9.8 | 9.3 | 10.0 | -1.8 | 11.3 | 11.3 | 11.0 | 9.5 | 9.6 | 8.9 | -1.9 | -0.87 | Slc16a10 |
| 10341149 | 8.9 | 8.4 | 8.4 | 5.7 | 5.3 | 6.2 | -2.8 | 7.7 | 8.0 | 7.0 | 5.5 | 4.5 | 5.6 | -2.4 | -0.87 | Unidentified |
| 10340116 | 12.1 | 11.7 | 11.8 | 9.5 | 9.2 | 9.9 | -2.3 | 11.3 | 11.5 | 10.8 | 9.0 | 9.2 | 9.4 | -2.0 | -0.87 | Unidentified |
| 10473367 | 12.2 | 12.1 | 12.4 | 9.1 | 9.1 | 9.9 | -2.9 | 12.2 | 12.2 | 11.8 | 8.7 | 9.0 | 8.9 | -3.2 | -0.87 | Slc43a1 |
| 10568638 | 10.5 | 10.1 | 10.5 | 8.5 | 8.0 | 8.6 | -2.0 | 10.0 | 10.0 | 9.7 | 8.1 | 8.2 | 8.0 | -1.8 | -0.87 | Uros |
| 10538459 | 12.1 | 11.9 | 12.4 | 9.6 | 9.5 | 10.2 | -2.4 | 12.2 | 12.2 | 11.9 | 9.2 | 9.5 | 9.2 | -2.8 | -0.87 | Aqp1 |
| 10379026 | 8.2 | 7.3 | 8.0 | 4.6 | 3.4 | 5.7 | -3.3 | 6.9 | 7.5 | 6.0 | 3.9 | 3.9 | 3.8 | -3.0 | -0.87 | mir-144 |
| 10339224 | 12.9 | 12.7 | 13.1 | 10.6 | 10.3 | 11.5 | -2.1 | 12.8 | 12.8 | 12.2 | 10.3 | 10.4 | 10.5 | -2.2 | -0.87 | Unidentified |
| 10344016 | 11.0 | 10.7 | 11.2 | 8.3 | 8.5 | 9.0 | -2.4 | 10.3 | 10.4 | 9.7 | 8.2 | 8.2 | 8.0 | -2.0 | -0.87 | Unidentified |
| 10379034 | 10.9 | 10.5 | 10.9 | 9.0 | 9.3 | 9.0 | -1.6 | 10.5 | 10.5 | 10.2 | 8.7 | 8.7 | 9.0 | -1.6 | -0.87 | Tlcd1 |
| 10463716 | 10.4 | 10.2 | 10.4 | 9.2 | 9.0 | 9.1 | -1.2 | 10.1 | 10.2 | 10.0 | 9.0 | 8.9 | 8.9 | -1.2 | -0.87 | Cnnm2 |
| 10343851 | 9.5 | 9.3 | 9.8 | 6.9 | 7.1 | 7.6 | -2.4 | 8.9 | 9.1 | 8.3 | 6.9 | 6.8 | 6.6 | -2.0 | -0.87 | Unidentified |
| 10597960 | 10.5 | 10.4 | 10.7 | 8.7 | 8.6 | 9.3 | -1.7 | 10.6 | 10.7 | 10.4 | 8.6 | 8.5 | 8.4 | -2.1 | -0.87 | Slc6a20a |
| 10356999 | 13.4 | 13.2 | 13.4 | 11.3 | 10.8 | 12.4 | -1.8 | 13.1 | 13.3 | 13.1 | 10.8 | 11.3 | 10.8 | -2.2 | -0.88 | Prdx2 |
| 10338772 | 12.0 | 11.7 | 11.7 | 9.4 | 9.1 | 9.8 | -2.4 | 11.5 | 11.5 | 11.1 | 8.8 | 9.2 | 9.2 | -2.3 | -0.88 | Unidentified |
| 10339381 | 12.5 | 12.4 | 12.4 | 10.2 | 10.2 | 10.7 | -2.1 | 12.1 | 12.0 | 11.6 | 9.8 | 10.0 | 10.1 | -1.9 | -0.88 | Unidentified |
| 10456140 | 9.8 | 9.6 | 10.0 | 8.1 | 8.0 | 8.4 | -1.6 | 9.7 | 9.9 | 9.6 | 7.8 | 8.1 | 7.7 | -1.8 | -0.88 | Sh3tc2 |
| 10573054 | 12.4 | 12.1 | 12.3 | 8.8 | 6.3 | 10.2 | -3.8 | 12.0 | 12.1 | 11.8 | 6.1 | 7.9 | 7.4 | -4.8 | -0.88 | Gypa |
| 10594825 | 9.7 | 9.4 | 9.6 | 7.6 | 7.5 | 8.0 | -1.9 | 9.2 | 9.2 | 8.9 | 7.1 | 7.7 | 7.3 | -1.8 | -0.88 | Aqp9 |
| 10391649 | 13.6 | 13.5 | 13.8 | 10.6 | 8.7 | 12.2 | -3.1 | 13.5 | 13.5 | 13.4 | 8.2 | 10.0 | 9.5 | -4.2 | -0.88 | Slc4a1 |
| 10338617 | 11.6 | 11.4 | 11.3 | 9.1 | 8.4 | 9.2 | -2.6 | 10.9 | 10.9 | 10.4 | 8.4 | 8.4 | 8.6 | -2.3 | -0.88 | Unidentified |
| 10443421 | 10.2 | 10.0 | 10.3 | 9.1 | 9.0 | 9.1 | -1.1 | 10.0 | 9.9 | 9.9 | 8.9 | 8.9 | 8.7 | -1.1 | -0.88 | Brpf3 |
| 10344221 | 11.7 | 11.2 | 11.5 | 9.1 | 9.0 | 9.6 | -2.2 | 10.7 | 10.9 | 10.3 | 8.5 | 8.7 | 9.2 | -1.9 | -0.88 | Unidentified |
| 10573457 | 11.6 | 11.4 | 11.6 | 8.7 | 8.0 | 9.2 | -2.9 | 11.2 | 11.4 | 11.0 | 7.8 | 8.4 | 8.0 | -3.1 | -0.88 | Klf1 |
| 10421172 | 13.2 | 12.9 | 13.2 | 11.0 | 10.9 | 12.2 | -1.7 | 12.9 | 12.9 | 12.7 | 10.4 | 10.6 | 11.3 | -2.1 | -0.88 | Slc25a37 |
| 10499062 | 11.5 | 11.1 | 11.5 | 8.1 | 7.5 | 8.8 | -3.2 | 11.2 | 11.3 | 10.7 | 7.3 | 7.7 | 7.5 | -3.6 | -0.88 | Fhdc1 |
| 10339230 | 12.4 | 12.2 | 12.2 | 9.7 | 9.3 | 10.2 | -2.5 | 11.9 | 12.1 | 11.5 | 9.1 | 9.5 | 9.3 | -2.5 | -0.88 | Unidentified |
| 10360090 | 11.0 | 11.0 | 11.4 | 10.0 | 10.0 | 10.2 | -1.1 | 11.0 | 11.0 | 10.8 | 9.9 | 9.9 | 9.3 | -1.2 | -0.88 | Ppox |
| 10338143 | 7.4 | 7.8 | 7.5 | 4.5 | 4.4 | 4.3 | -3.2 | 5.5 | 6.8 | 5.2 | 3.9 | 3.0 | 3.4 | -2.4 | -0.88 | Unidentified |
| 10513608 | 13.9 | 13.7 | 13.9 | 12.0 | 11.5 | 12.5 | -1.8 | 13.8 | 13.8 | 13.6 | 11.4 | 11.8 | 11.5 | -2.2 | -0.88 | Alad |
| 10339875 | 11.1 | 10.6 | 11.2 | 8.9 | 8.7 | 9.1 | -2.1 | 10.5 | 10.5 | 9.9 | 8.4 | 8.5 | 8.7 | -1.8 | -0.88 | Unidentified |
| 10338163 | 13.3 | 13.2 | 13.5 | 11.6 | 11.3 | 11.9 | -1.7 | 13.2 | 13.1 | 12.7 | 11.2 | 11.3 | 11.5 | -1.7 | -0.88 | Unidentified |
| 10341388 | 11.9 | 11.9 | 12.1 | 9.2 | 8.9 | 10.0 | -2.6 | 11.7 | 11.7 | 11.0 | 8.6 | 9.0 | 9.1 | -2.6 | -0.88 | Unidentified |
| 10340287 | 14.3 | 14.2 | 14.3 | 12.9 | 12.7 | 13.3 | -1.3 | 14.3 | 14.2 | 14.0 | 12.6 | 12.7 | 12.7 | -1.5 | -0.88 | Unidentified |
| 10570894 | 12.7 | 12.4 | 12.7 | 9.1 | 8.5 | 10.3 | -3.3 | 12.4 | 12.4 | 12.0 | 8.3 | 8.8 | 8.4 | -3.8 | -0.88 | Ank1 |
| 10341386 | 11.3 | 11.1 | 11.4 | 8.8 | 8.4 | 8.8 | -2.6 | 10.8 | 11.0 | 10.4 | 8.1 | 8.0 | 8.6 | -2.5 | -0.88 | Unidentified |
| 10339885 | 13.3 | 13.1 | 13.4 | 11.3 | 10.7 | 11.8 | -2.0 | 13.2 | 13.1 | 12.7 | 10.9 | 10.9 | 10.7 | -2.2 | -0.88 | Unidentified |
| 10341603 | 9.5 | 9.1 | 9.6 | 7.1 | 7.1 | 7.5 | -2.1 | 8.9 | 9.1 | 8.2 | 6.7 | 6.9 | 6.9 | -1.9 | -0.88 | Unidentified |
| 10525473 | 11.1 | 10.8 | 11.2 | 9.6 | 9.3 | 9.6 | -1.5 | 11.0 | 11.1 | 10.9 | 9.3 | 9.2 | 8.9 | -1.9 | -0.88 | Tmem120b |
| 10563852 | 10.3 | 9.9 | 10.4 | 8.6 | 8.5 | 8.7 | -1.6 | 10.2 | 10.2 | 9.8 | 8.1 | 8.4 | 8.0 | -1.9 | -0.88 | Nipa1 |
| 10343840 | 11.2 | 10.7 | 11.0 | 8.4 | 8.4 | 8.8 | -2.4 | 10.1 | 10.3 | 9.8 | 8.0 | 8.0 | 8.2 | -2.0 | -0.88 | Unidentified |
| 10343328 | 10.9 | 10.8 | 11.1 | 8.9 | 8.7 | 9.3 | -1.9 | 10.7 | 10.7 | 10.2 | 8.4 | 8.8 | 8.4 | -2.0 | -0.89 | Unidentified |
| 10338760 | 13.6 | 13.5 | 13.6 | 11.6 | 11.2 | 12.0 | -2.0 | 13.4 | 13.2 | 12.8 | 11.2 | 11.2 | 11.2 | -2.0 | -0.89 | Unidentified |
| 10339804 | 12.1 | 11.7 | 11.7 | 9.4 | 9.5 | 9.9 | -2.3 | 11.3 | 11.7 | 10.9 | 9.1 | 9.0 | 9.1 | -2.2 | -0.89 | Unidentified |
| 10351905 | 12.3 | 11.8 | 12.2 | 8.1 | 7.1 | 9.9 | -3.7 | 10.9 | 11.0 | 10.6 | 6.9 | 7.4 | 7.7 | -3.5 | -0.89 | Spta1 |
| 10338991 | 8.4 | 8.5 | 7.5 | 6.4 | 6.7 | 6.6 | -1.6 | 7.1 | 7.4 | 6.8 | 5.7 | 5.2 | 5.8 | -1.5 | -0.89 | Unidentified |
| 10463486 | 11.3 | 11.2 | 11.4 | 10.3 | 10.2 | 10.4 | -1.0 | 11.1 | 11.2 | 10.9 | 10.1 | 10.2 | 9.9 | -1.0 | -0.89 | Btrc |
| 10342272 | 11.8 | 11.4 | 11.7 | 9.3 | 9.3 | 9.8 | -2.2 | 11.4 | 11.3 | 10.9 | 9.0 | 9.0 | 9.0 | -2.3 | -0.89 | Unidentified |
| 10486664 | 12.2 | 11.9 | 12.2 | 8.3 | 7.6 | 9.8 | -3.5 | 11.9 | 11.9 | 11.4 | 7.4 | 7.9 | 7.5 | -4.1 | -0.89 | Epb42 |
| 10339362 | 11.1 | 10.7 | 10.9 | 8.7 | 8.4 | 8.7 | -2.3 | 10.6 | 10.5 | 9.7 | 8.0 | 7.9 | 8.5 | -2.1 | -0.89 | Unidentified |
| 10419082 | 13.5 | 13.2 | 13.2 | 11.7 | 11.5 | 12.5 | -1.4 | 13.0 | 13.1 | 12.9 | 11.4 | 11.6 | 11.3 | -1.6 | -0.89 | Fam213a |
| 10340051 | 12.7 | 12.4 | 12.5 | 10.3 | 10.1 | 11.0 | -2.1 | 12.2 | 12.3 | 11.6 | 9.8 | 9.9 | 10.1 | -2.1 | -0.89 | Unidentified |
| 10340720 | 13.5 | 13.4 | 13.6 | 11.5 | 11.0 | 12.0 | -2.0 | 13.4 | 13.2 | 12.8 | 11.1 | 11.1 | 11.1 | -2.0 | -0.89 | Unidentified |
| 10344068 | 10.4 | 10.3 | 10.7 | 7.7 | 7.5 | 7.9 | -2.8 | 9.4 | 9.8 | 9.1 | 7.1 | 6.9 | 7.5 | -2.3 | -0.89 | Unidentified |
| 10342215 | 12.0 | 11.6 | 11.9 | 9.8 | 9.8 | 10.1 | -2.0 | 11.5 | 11.5 | 11.1 | 9.3 | 9.6 | 9.5 | -1.9 | -0.89 | Unidentified |
| 10344117 | 13.2 | 12.9 | 13.2 | 11.0 | 11.3 | 11.8 | -1.7 | 12.9 | 12.8 | 12.4 | 10.9 | 10.9 | 11.0 | -1.8 | -0.89 | Unidentified |
| 10343402 | 13.9 | 13.8 | 13.9 | 12.1 | 12.0 | 12.8 | -1.6 | 13.8 | 13.7 | 13.4 | 11.9 | 11.9 | 11.9 | -1.8 | -0.89 | Unidentified |
| 10339749 | 12.6 | 12.6 | 12.9 | 11.3 | 11.1 | 11.4 | -1.5 | 12.5 | 12.5 | 12.1 | 11.0 | 11.1 | 10.9 | -1.4 | -0.89 | Unidentified |
| 10341062 | 13.5 | 13.3 | 13.6 | 11.5 | 11.1 | 12.0 | -1.9 | 13.4 | 13.2 | 12.8 | 10.9 | 11.1 | 11.2 | -2.1 | -0.89 | Unidentified |
| 10344372 | 9.0 | 9.0 | 8.9 | 6.6 | 6.6 | 6.5 | -2.4 | 7.4 | 8.1 | 6.5 | 5.0 | 5.6 | 5.8 | -1.9 | -0.89 | Unidentified |
| 10341616 | 11.5 | 11.4 | 11.6 | 9.2 | 9.0 | 9.5 | -2.3 | 11.0 | 10.8 | 10.5 | 8.7 | 8.6 | 9.0 | -2.0 | -0.89 | Unidentified |
| 10532241 | 9.1 | 9.1 | 9.5 | 7.5 | 7.7 | 7.9 | -1.5 | 8.8 | 9.3 | 8.8 | 7.3 | 7.4 | 7.3 | -1.6 | -0.89 | Slc26a1 |
| 10603087 | 9.4 | 9.1 | 9.5 | 7.4 | 7.4 | 7.8 | -1.8 | 8.7 | 8.9 | 8.5 | 7.1 | 7.1 | 7.1 | -1.6 | -0.89 | Pir |
| 10534389 | 11.9 | 11.8 | 12.3 | 8.5 | 7.2 | 9.3 | -3.6 | 11.9 | 12.0 | 11.5 | 7.3 | 7.7 | 7.0 | -4.4 | -0.89 | Cldn13 |
| 10580210 | 11.5 | 11.3 | 11.6 | 10.4 | 10.0 | 10.4 | -1.2 | 11.4 | 11.4 | 11.1 | 10.0 | 10.1 | 9.8 | -1.3 | -0.89 | Rad23a |
| 10342241 | 11.2 | 11.0 | 11.0 | 8.4 | 8.2 | 8.7 | -2.6 | 10.4 | 10.4 | 9.8 | 7.9 | 8.0 | 7.5 | -2.4 | -0.89 | Unidentified |
| 10338377 | 11.9 | 11.7 | 11.4 | 9.1 | 8.6 | 9.3 | -2.7 | 11.1 | 11.4 | 10.7 | 8.2 | 8.5 | 8.5 | -2.6 | -0.89 | Unidentified |
| 10344286 | 11.1 | 11.0 | 11.2 | 9.0 | 9.2 | 9.3 | -2.0 | 10.7 | 10.8 | 10.3 | 8.6 | 8.6 | 8.9 | -1.9 | -0.89 | Unidentified |
| 10341495 | 11.2 | 11.1 | 11.4 | 9.2 | 9.0 | 9.5 | -2.0 | 10.6 | 10.7 | 10.1 | 8.8 | 8.7 | 8.8 | -1.7 | -0.89 | Unidentified |
| 10342237 | 11.5 | 11.3 | 11.8 | 8.8 | 8.9 | 9.1 | -2.6 | 10.6 | 10.5 | 10.2 | 8.1 | 8.4 | 8.4 | -2.1 | -0.89 | Unidentified |
| 10600502 | 11.0 | 10.6 | 10.7 | 9.2 | 8.8 | 10.4 | -1.3 | 10.3 | 10.3 | 10.0 | 8.6 | 8.6 | 8.9 | -1.5 | -0.89 | Fam220a |
| 10421456 | 13.0 | 12.8 | 13.1 | 11.0 | 10.7 | 11.5 | -1.9 | 12.8 | 12.8 | 12.3 | 10.5 | 10.6 | 10.7 | -2.1 | -0.90 | Xpo7 |
| 10344422 | 13.1 | 12.8 | 13.1 | 10.9 | 10.7 | 11.4 | -2.0 | 12.6 | 12.6 | 12.2 | 10.4 | 10.6 | 10.6 | -1.9 | -0.90 | Unidentified |
| 10339146 | 10.4 | 10.1 | 10.1 | 8.3 | 8.2 | 8.1 | -2.0 | 9.4 | 9.7 | 8.9 | 7.1 | 7.7 | 7.9 | -1.8 | -0.90 | Unidentified |
| 10357698 | 10.3 | 10.2 | 10.5 | 8.3 | 8.0 | 8.7 | -2.0 | 10.2 | 10.4 | 9.9 | 8.0 | 7.9 | 7.7 | -2.3 | -0.90 | Tmcc2 |
| 10342397 | 13.7 | 13.5 | 13.6 | 12.1 | 11.9 | 12.5 | -1.5 | 13.5 | 13.4 | 13.2 | 11.7 | 11.7 | 11.9 | -1.6 | -0.90 | Unidentified |
| 10339296 | 12.5 | 12.2 | 12.4 | 9.9 | 9.7 | 10.2 | -2.4 | 12.0 | 12.0 | 11.4 | 9.2 | 9.5 | 9.5 | -2.4 | -0.90 | Unidentified |
| 10339823 | 13.4 | 13.3 | 13.6 | 11.6 | 11.3 | 12.0 | -1.8 | 13.3 | 13.2 | 12.8 | 11.1 | 11.3 | 11.3 | -1.9 | -0.90 | Unidentified |
| 10338654 | 12.8 | 12.7 | 12.9 | 10.5 | 10.3 | 11.3 | -2.1 | 12.6 | 12.5 | 12.0 | 10.2 | 10.1 | 10.1 | -2.2 | -0.90 | Unidentified |
| 10341811 | 10.8 | 10.3 | 10.1 | 7.7 | 6.9 | 7.6 | -3.0 | 9.2 | 9.5 | 8.7 | 6.8 | 6.6 | 5.8 | -2.8 | -0.90 | Unidentified |
| 10340688 | 10.9 | 10.8 | 11.2 | 8.5 | 8.4 | 8.8 | -2.4 | 10.1 | 10.2 | 9.7 | 7.8 | 7.9 | 8.1 | -2.0 | -0.90 | Unidentified |
| 10344572 | 11.3 | 11.1 | 11.1 | 9.0 | 8.9 | 9.5 | -2.1 | 10.5 | 10.5 | 10.1 | 8.4 | 8.6 | 8.6 | -1.8 | -0.90 | Unidentified |
| 10458983 | 10.0 | 9.9 | 10.0 | 8.7 | 8.4 | 8.8 | -1.3 | 9.6 | 9.8 | 9.4 | 8.3 | 8.2 | 8.5 | -1.3 | -0.90 | March3 |
| 10342380 | 12.0 | 11.7 | 11.9 | 9.3 | 9.0 | 9.7 | -2.5 | 11.2 | 11.2 | 10.8 | 8.6 | 8.8 | 8.7 | -2.4 | -0.90 | Unidentified |
| 10593198 | 13.1 | 12.8 | 12.9 | 9.2 | 8.9 | 10.5 | -3.4 | 12.6 | 12.7 | 12.3 | 8.3 | 8.9 | 7.8 | -4.2 | -0.90 | Nxpe2 |
| 10551347 | 12.5 | 12.6 | 12.8 | 11.1 | 10.6 | 11.4 | -1.6 | 12.6 | 12.6 | 12.3 | 10.5 | 10.7 | 10.0 | -2.0 | -0.90 | Blvrb |
| 10339904 | 10.9 | 10.7 | 11.1 | 9.0 | 9.2 | 9.6 | -1.7 | 10.6 | 10.7 | 10.2 | 8.8 | 8.8 | 8.5 | -1.8 | -0.90 | Unidentified |
| 10339249 | 13.1 | 12.8 | 13.1 | 11.0 | 10.9 | 11.4 | -1.9 | 12.7 | 12.7 | 12.2 | 10.5 | 10.5 | 10.7 | -2.0 | -0.90 | Unidentified |
| 10340920 | 13.3 | 13.1 | 13.4 | 11.3 | 11.0 | 11.9 | -1.9 | 13.0 | 13.0 | 12.7 | 10.8 | 10.8 | 11.0 | -2.0 | -0.90 | Unidentified |
| 10501802 | 11.5 | 11.2 | 11.5 | 8.3 | 7.1 | 8.4 | -3.5 | 10.6 | 11.1 | 10.6 | 6.9 | 7.5 | 5.9 | -4.0 | -0.90 | Tmem56 |
| 10343106 | 10.8 | 10.6 | 10.6 | 8.1 | 8.4 | 8.4 | -2.4 | 9.9 | 9.8 | 9.2 | 7.2 | 7.5 | 7.8 | -2.2 | -0.90 | Unidentified |
| 10341847 | 13.6 | 13.4 | 13.4 | 11.7 | 11.3 | 12.0 | -1.8 | 13.3 | 13.3 | 12.7 | 11.1 | 11.1 | 11.2 | -2.0 | -0.90 | Unidentified |
| 10555460 | 12.0 | 11.9 | 12.1 | 11.0 | 11.3 | 11.0 | -1.0 | 11.8 | 11.9 | 11.7 | 10.7 | 10.7 | 10.9 | -1.1 | -0.91 | Stard10 |
| 10474201 | 12.1 | 12.0 | 12.1 | 11.1 | 10.7 | 11.2 | -1.0 | 12.0 | 12.0 | 11.8 | 10.6 | 10.8 | 10.5 | -1.3 | -0.91 | Lmo2 |
| 10528385 | 9.5 | 9.5 | 9.4 | 8.5 | 7.8 | 8.4 | -1.2 | 9.3 | 9.4 | 9.1 | 7.8 | 8.0 | 7.8 | -1.4 | -0.91 | Reln |
| 10339594 | 11.0 | 10.6 | 10.8 | 8.5 | 7.8 | 8.5 | -2.5 | 9.9 | 9.9 | 9.2 | 7.6 | 7.5 | 7.3 | -2.2 | -0.91 | Unidentified |
| 10541307 | 10.9 | 10.5 | 10.6 | 9.7 | 9.0 | 9.6 | -1.2 | 10.1 | 10.1 | 10.1 | 8.6 | 9.0 | 7.6 | -1.7 | -0.91 | Usp18 |
| 10343283 | 11.3 | 11.0 | 11.6 | 8.9 | 9.0 | 9.1 | -2.3 | 10.1 | 10.0 | 9.6 | 7.9 | 7.8 | 8.4 | -1.9 | -0.91 | Unidentified |
| 10342475 | 11.2 | 10.9 | 11.5 | 8.7 | 8.8 | 9.4 | -2.2 | 9.9 | 10.0 | 9.5 | 7.2 | 8.2 | 8.2 | -1.9 | -0.91 | Unidentified |
| 10342665 | 11.0 | 10.5 | 10.6 | 7.7 | 7.4 | 8.0 | -3.0 | 8.9 | 9.6 | 9.1 | 6.5 | 7.1 | 6.3 | -2.6 | -0.91 | Unidentified |
| 10343514 | 11.5 | 11.2 | 11.7 | 9.2 | 9.4 | 9.6 | -2.1 | 10.9 | 10.9 | 10.2 | 8.5 | 8.5 | 9.1 | -2.0 | -0.91 | Unidentified |
| 10343129 | 8.3 | 8.1 | 8.1 | 6.5 | 6.0 | 6.2 | -1.9 | 6.1 | 7.6 | 6.8 | 4.9 | 5.4 | 5.1 | -1.7 | -0.91 | Unidentified |
| 10345791 | 9.4 | 9.1 | 9.3 | 8.2 | 7.9 | 7.9 | -1.3 | 8.7 | 9.1 | 8.6 | 7.4 | 7.7 | 6.7 | -1.5 | -0.91 | Il1rl1 |
| 10338674 | 13.5 | 13.5 | 13.4 | 12.1 | 11.9 | 12.3 | -1.4 | 12.9 | 13.3 | 12.9 | 11.7 | 11.8 | 11.7 | -1.3 | -0.91 | Unidentified |
| 10343345 | 12.3 | 12.0 | 12.1 | 10.3 | 9.6 | 10.4 | -2.0 | 11.5 | 11.8 | 11.2 | 9.5 | 9.5 | 9.6 | -2.0 | -0.91 | Unidentified |
| 10366446 | 10.3 | 10.1 | 10.4 | 6.8 | 6.5 | 7.7 | -3.2 | 9.6 | 9.9 | 9.4 | 5.7 | 6.1 | 6.3 | -3.6 | -0.91 | Tspan8 |
| 10341472 | 11.4 | 10.9 | 11.0 | 9.2 | 8.6 | 9.1 | -2.2 | 10.4 | 10.7 | 10.0 | 8.3 | 8.4 | 8.1 | -2.1 | -0.91 | Unidentified |
| 10338866 | 10.6 | 10.6 | 10.8 | 8.8 | 8.5 | 8.3 | -2.1 | 9.2 | 9.5 | 8.5 | 7.4 | 7.1 | 7.2 | -1.8 | -0.91 | Unidentified |
| 10548875 | 9.1 | 8.9 | 9.2 | 8.1 | 8.1 | 8.2 | -1.0 | 8.8 | 8.8 | 8.8 | 7.9 | 7.8 | 7.7 | -1.0 | -0.91 | Art4 |
| 10572097 | 9.3 | 9.1 | 9.2 | 8.3 | 7.8 | 8.2 | -1.1 | 8.4 | 8.8 | 8.3 | 7.7 | 7.5 | 7.2 | -1.1 | -0.92 | Sh2d4a |
| 10341552 | 11.4 | 11.2 | 11.4 | 9.5 | 9.3 | 9.7 | -1.8 | 10.8 | 10.5 | 10.2 | 8.7 | 8.7 | 8.9 | -1.7 | -0.92 | Unidentified |
| 10339563 | 11.3 | 10.9 | 10.9 | 8.8 | 8.2 | 9.1 | -2.3 | 10.4 | 10.4 | 9.8 | 7.5 | 8.0 | 8.1 | -2.3 | -0.92 | Unidentified |
| 10342981 | 11.1 | 10.8 | 10.9 | 8.7 | 8.2 | 8.5 | -2.4 | 9.5 | 9.7 | 9.3 | 6.7 | 7.6 | 7.8 | -2.2 | -0.92 | Unidentified |
| 10341023 | 12.3 | 12.1 | 12.3 | 10.3 | 10.3 | 10.7 | -1.8 | 11.9 | 12.0 | 11.4 | 9.8 | 9.8 | 9.5 | -2.0 | -0.92 | Unidentified |
| 10490923 | 13.7 | 13.5 | 13.6 | 10.9 | 10.5 | 11.7 | -2.6 | 13.5 | 13.5 | 13.2 | 9.6 | 10.3 | 9.9 | -3.5 | -0.92 | Car2 |
| 10507500 | 12.4 | 12.2 | 12.7 | 10.9 | 10.4 | 10.7 | -1.8 | 12.6 | 12.5 | 12.3 | 9.9 | 9.6 | 9.8 | -2.7 | -0.93 | Slc6a9 |
| 10552681 | 10.5 | 10.4 | 10.7 | 9.5 | 8.9 | 9.5 | -1.2 | 10.2 | 10.3 | 10.0 | 8.9 | 8.9 | 8.4 | -1.4 | -0.93 | Josd2 |
| 10339089 | 11.9 | 11.6 | 11.8 | 9.1 | 9.0 | 9.7 | -2.5 | 11.2 | 11.2 | 10.6 | 8.1 | 8.3 | 8.5 | -2.7 | -0.93 | Unidentified |
| 10343768 | 10.5 | 10.2 | 10.1 | 8.2 | 7.5 | 7.9 | -2.4 | 9.5 | 9.8 | 8.6 | 5.9 | 7.0 | 6.8 | -2.7 | -0.94 | Unidentified |
| 10344040 | 10.7 | 10.4 | 10.7 | 8.3 | 8.2 | 8.7 | -2.2 | 9.7 | 9.8 | 9.4 | 7.3 | 7.6 | 7.2 | -2.3 | -0.94 | Unidentified |
| 10339308 | 12.0 | 11.7 | 11.8 | 10.1 | 10.2 | 10.1 | -1.7 | 10.5 | 10.8 | 10.0 | 8.5 | 8.5 | 8.7 | -1.9 | -0.94 | Unidentified |
| 10461408 | 11.8 | 11.7 | 11.9 | 10.2 | 10.1 | 10.4 | -1.6 | 11.5 | 11.5 | 11.1 | 9.3 | 9.7 | 9.5 | -1.9 | -0.94 | Rab3il1 |
| 10338101 | 12.5 | 12.4 | 12.8 | 10.6 | 10.7 | 11.1 | -1.8 | 12.1 | 12.0 | 11.5 | 9.7 | 9.8 | 10.0 | -2.0 | -0.94 | Unidentified |
| 10587871 | 11.3 | 10.9 | 11.2 | 9.5 | 9.5 | 10.0 | -1.5 | 10.7 | 10.9 | 10.6 | 8.5 | 8.9 | 8.7 | -2.0 | -0.95 | Paqr9 (mPRε) |

**Supplemental Table 21.** Data mining of peripheral surrogate marker candidates, which were up- or down-regulated in mouse spleens, on the human blood transcriptome database of MS patients

| **Gene**  **symbol** | **Gene name** | **Sample number** | **Gene expression (mean ± SEM)** | | ***P*** | **References** |
| --- | --- | --- | --- | --- | --- | --- |
|  |  |  | **Control** | **MS** |  |  |
| **Up-regulated genes*** | |  |  |  |  |  |
| *Per1* (*MIR6883*) | period circadian clock 1 (microRNA 6883) | 27  (Control: 15, MS: 12) | 44.6 ± 7.3 | 379.2 ± 44.7 | < 0.01 | (1) |
| *FKBP5* | FK506 binding protein 5 | 7  (Control: 4, MS with high serum Sema4A level: 3) | 7740.8 ± 290.9 | 27902.5 ± 5508.1 | < 0.05 | (2) |
| **Down-regulated gene**** | |  |  |  |  |  |
| *SLC16A1* (*MCT1*) | solute carrier family 16 member 1 (monocarboxylate transporter 1) | 27  (Control: 15, MS: 12) | 92.0 ± 3.0 | 35.8 ± 2.6 | < 0.01 | (1) |

*Search keywords for up-regulated marker candidates: "multiple sclerosis"[All Fields] AND "Homo sapiens"[Organism] AND "peripheral blood"[All Fields] AND "disease state"[Flag Information] AND (Adgrg2 OR Lrg1 OR Pik3ip1 OR Per1 OR Cntfr OR BC005685 OR Stfa2l1 OR Fam107a OR Xdh OR Fkbp5 OR Cyp2d22 OR C7 OR Serpina3n OR Zbtb16 OR Tsc22d3 OR Lox OR Ctrc OR LOC641050 OR Fmo2 OR BC057193 OR Pdk4 OR Ptpn22 OR Errfi1 OR Ddit4 OR Neb OR Pdgfra OR Sult1a1 OR Scn7a)

**Search keywords for down-regulated marker candidates: "multiple sclerosis"[All Fields] AND "Homo sapiens"[Organism] AND "peripheral blood"[All Fields] AND "disease state"[Flag Information] AND (Paqr9 OR Rab3il1 OR Josd2 OR Slc6a9 OR Car2 OR Sh2d4a OR Art4 OR Tspan8 OR Il1rl1 OR Usp18 OR Reln OR Lmo2 OR Stard10 OR Tmem56 OR Blvrb OR Nxpe2 OR March3 OR Tmcc2 OR Xpo7 OR Fam220a OR Rad23a OR Cldn13 OR Pir OR Slc26a1 OR Fam213a OR Epb42 OR Btrc OR Spta1 OR Nipa1 OR Tmem120b OR Ank1 OR Alad OR Ppox OR Fhdc1 OR Slc25a37 OR Klf1 OR Brpf3 OR Slc4a1 OR Aqp9 OR Gypa OR Sh3tc2 OR Prdx2 OR Slc6a20a OR Cnnm2 OR Tlcd1 OR mir144 OR Aqp1 OR Uros OR Slc43a1 OR Slc16a10 OR Hebp1 OR F930017D23Rik OR Rhag OR Slc38a5 OR Rhd OR Trmo OR Sowaha OR Txnrd2 OR Pkhd1l1 OR Bex4 OR Kel OR Abcg4 OR Carhsp1 OR Reep6 OR Gm867 OR Add2 OR Fn3krp OR Tspan33 OR Fech OR Ypel4 OR Fam210b OR Snx15 OR Dapk2 OR Prdx2 OR Tac2 OR Trim10 OR Ermap OR Trim59 OR Dmtn OR Car3 OR Garem OR Il22ra2 OR Slc2a4 OR Dpf3 OR Tmod1 OR March8 OR Nrip3 OR Bsdc1 OR Fn3k OR Fahd1 OR Btnl10 OR Isg20 OR St3gal5 OR Sox6 OR Sept8 OR Pigq OR Cln8 OR Hmbs OR Cdr2 OR Gypc OR Abcg2 OR Cmpk2 OR Micall2 OR E2f4 OR Mpp2 OR Hemgn OR Scrn3 OR Ranbp10 OR Ifrd2 OR 1300017J02Rik OR Otub2 OR Sh3yl1 OR Mgst3 OR Abcb6 OR Slc25a38 OR Pla2g4c OR Hagh OR Plek2 OR Ackr1 OR Dhrs11 OR Sppl2b OR Hmbs OR Chac2 OR Gclc OR Pklr OR Rfx2 OR Ptdss2 OR Myh10 OR Rnf212 OR Mtfr1 OR Ttll12 OR Fam46c OR Suox OR Atp7b OR Atg4a OR Dnajb3 OR Tfrc OR Slc25a21 OR Ppp2r5b OR Slc14a1 OR Snx22 OR Urod OR Steap3 OR Rsad2 OR 6030468B19Rik OR Ubac1 OR Slc30a10 OR Epdr1 OR Igtp OR Cd59b OR Spire1 OR Slc16a1 OR Stx2 OR Glrx5 OR Gstm5 OR Cdc25b OR Bpgm OR Hbq1b OR Minpp1 OR Nxpe4 OR Acsl6 OR Gm9895 OR Mns1 OR Alas2 OR Tspo2 OR Slc22a23 OR Abcb10 OR Cd24a)

**References**

1. [Dataset] Kamppinen, A.K., and Saarela, J. (2011). *Expression data from peripheral blood mononuclear cells in multiple sclerosis patients and controls.* Gene Expression Omnibus. Available: https://www.ncbi.nlm.nih.gov/geo/query/acc.cgi?acc=GSE21942.

2. [Dataset] Nakatsuji, Y., Okuno, T., Moriya, M., Sugimoto, T., Kinoshita, M., Takamatsu, H., et al. (2011). *Sema4A, a novel serum marker of multiple sclerosis, implicates Th17 pathology and efficacy of interferon-β.* Gene Expression Omnibus. Available: https://www.ncbi.nlm.nih.gov/geo/query/acc.cgi?acc=GSE26484.
